# Photoinduced Ruthenium-Catalyzed *meta*-C–H Glycosylation

**DOI:** 10.1021/acscatal.5c02183

**Published:** 2025-06-05

**Authors:** Julia Pöhlmann, Binbin Yuan, Rajeshwaran Purushothaman, Jun Wu, Lutz Ackermann

**Affiliations:** † WISCh (Wöhler Research Institute for Sustainable Chemistry), 9375 Georg-August-Universität Göttingen, Tammannstraße 2, 37077 Göttingen, Germany; ‡ DZHK (German Centre for Cardiovascular Research), Potsdamer Straße 58, 10785 Berlin, Germany

**Keywords:** C−H activation, photoinduced *meta*-C-aryl glycosylation, glycosyl bromide, ruthenium catalysis, C-glycosylation

## Abstract

C–H functionalization has surfaced as a powerful tool in molecular synthesis. *meta*-Selective arene C–H activations typically rely on rather costly palladium or rhodium catalysts, elaborate template auxiliaries, or elevated temperatures, and *meta*-glycosylations are scarce. In contrast, we herein report on a visible-light-induced ruthenium-catalyzed *meta*-C–H glycosylation that utilizes a stable ruthenium­(II) catalyst under exceedingly mild conditions at room temperature. This strategy is operative without an exogenous photocatalyst. The versatile ruthenium­(II) catalyst featured high *meta*- and anomeric α-selectivity, employing readily accessible glycosyl bromides.

## Graphical abstract

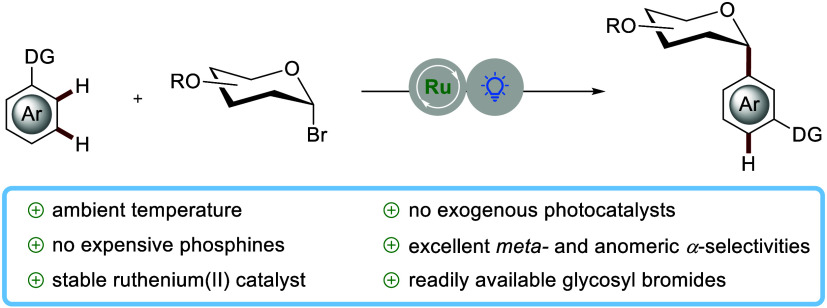

## Introduction

Over the past decade, photoinduced C–H functionalization has emerged as an increasingly viable platform for molecular synthesis.^1^ One key approach involves the synergistic collaboration of a transition metal with a photosensitizer, enabling dual catalytic maneuvers (Scheme 1a).^2^ Sporadically, photosensitizer can be avoided within metallaphotocatalysis.^3^ Here, the direct excitation of the transition metal facilitates both light absorption and strong bond cleavage (Scheme 1a). In this regard, Ackermann and Greaney concurrently reported on ruthenium-catalyzed *ortho*-C–H arylations under visible light without additional photocatalysts.^4^ Despite these undisputable advances, remote *meta*-C–H functionalization *via* photocatalysis remains scarce, with recent elegant findings by Maiti on powerful photoinduced *meta*-C–H functionalization of arenes through engineered template-directed palladium catalysis (Scheme 1b).^5^ In this context, Ackermann and Greaney utilized a well-defined, stable ruthenium­(II) complex for *meta*-C–H alkylations, employing secondary and tertiary alkyl bromides at room temperature under light irradiation (Scheme 1b).^6^ Unfortunately, ruthenium-catalyzed *meta*-selective C–H alkylations rely on relatively expensive phosphine ligands,^6b,7^ such as P­(4-CF_3_–C_6_H_4_)_3_ (51 €/mmol).^8^ Recently, *meta*-*C*-aryl glycoside moieties, a key scaffold in drugs such as Canagliflozin and Dapagliflozin, gained attention due to their pharmacological effects and resistance to enzymatic degradation and hydrolysis.^9^ Hence, transition-metal-catalyzed cross-couplings were developed for the synthesis of *C*-aryl glycosides.^10^ Moreover, direct C–H functionalization mediated by transition metals was proven to be a sustainable and elegant strategy for the synthesis of complex glycosides from readily available glycosyl donors.^11^ However, reports on the synthesis of *meta*-C–H glycosides remain scarce. Glycoside functionalization is challenging, given the tendency toward epimerization or ring-opening under heat and acidic conditions.^12^ Therefore, selective arene C–H glycosylation with high stereoselectivity at ambient temperature remains elusive. Herein, we report on *meta*-C–H glycosylation enabled by photoinduced ruthenium catalysis at ambient temperature (Scheme 1c). In contrast to previous *meta*-C–H glycosylation approaches, our strategy features an exceedingly simple catalytic system, avoids external photocatalysts, can be conducted at ambient temperature, and obviates expensive phosphine ligands required under *meta*-selective ruthenium­(II) catalysis. Importantly, readily accessible glycosyl bromides can be converted into *C*-aryl glycosides with outstanding *meta*- and anomeric α-selectivities.

**Scheme 1:**
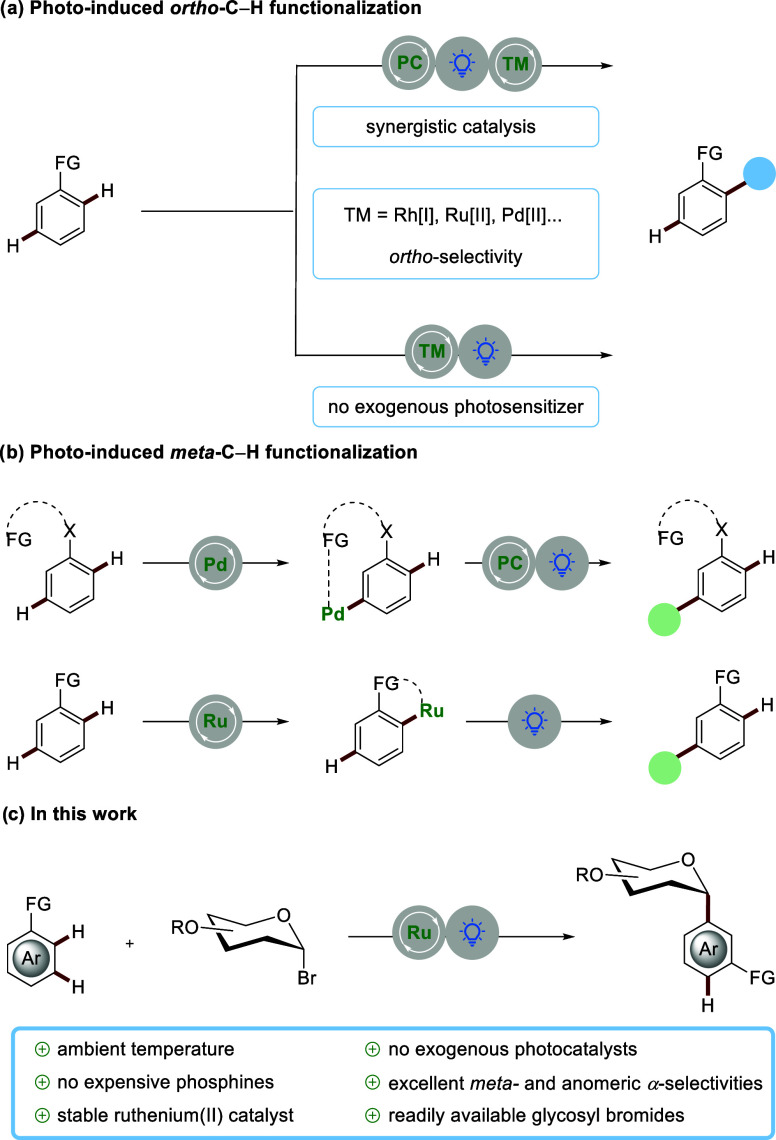
(a) Strategies for Photocatalyzed *ortho*-C–H and (b) *meta*-C–H Functionalization; (c) Photoinduced Ruthenium­(II)-Catalyzed *meta*-C–H Glycosylation toward *meta*-*C*-Aryl Glycosides

**Scheme 2:**
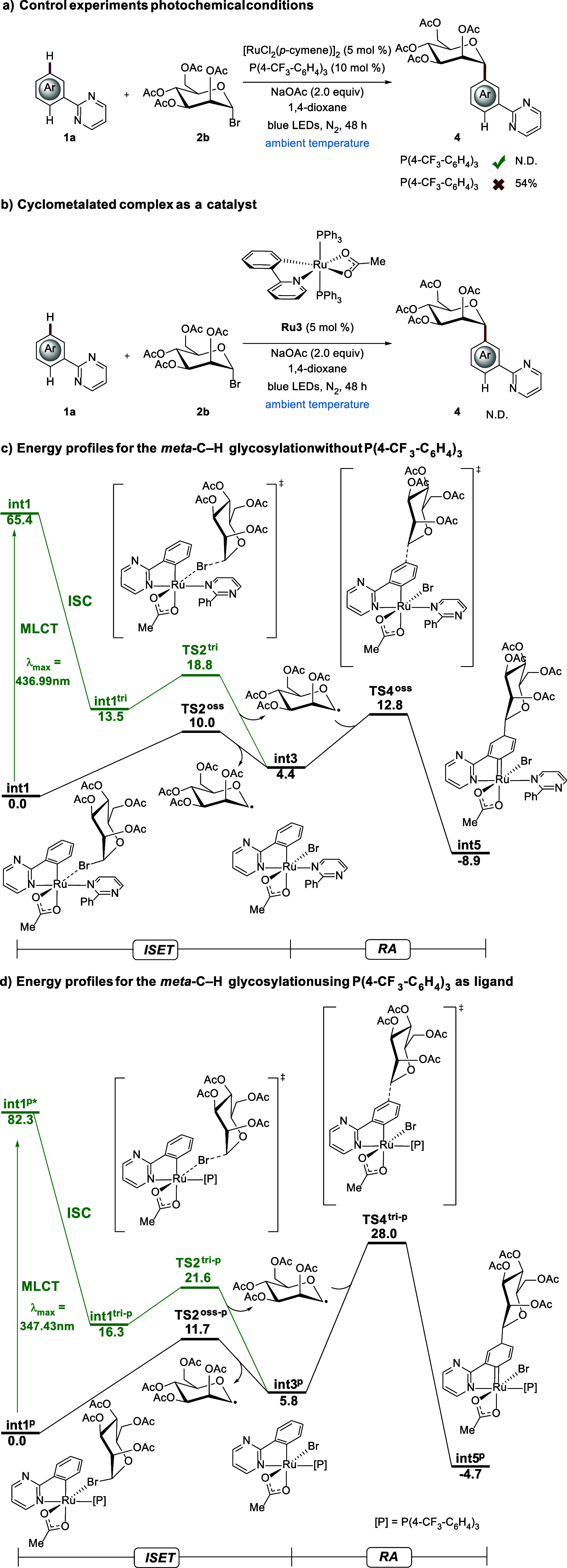
Key Mechanistic Studies

## Results and Discussion

We initiated our studies toward the desired *meta*-*C*(sp^2^)–H glycosylation using mannosyl bromide **2a** as the glycosyl donor, NaOAc as a base, and [RuCl_2_(*p*-cymene)]_2_ (see Table 1). While K_2_CO_3_ and organic bases were not suitable as bases, K_3_PO_4_ afforded the *meta*-substituted arene **3** in 61% yield (Entry 2).^13^ Among a variety of solvents, 1,4-dioxane proved to be optimal (Entry 3). Control experiments verified the essential roles of NaOAc, light irradiation, and the [RuCl_2_(*p*-cymene)]_2_ precatalyst (Entry 4). Well-defined ruthenium­(II) carboxylate complexes furnished lower yields (Entries 5 and 6). Cyclometalated ruthenium­(II) complex **Ru1** failed to give the *meta*-*C*-aryl product (Entry 7). Likewise, an air-stable ruthenium aqua complex^14^ **Ru2** proved unsuitable (Entry 8). The mass balance accounted for unreacted substrate **1a**, and side products derived from it have not been observed. Utilization of [Ru­(OAc)_2_(*p*-cymene)] led to a yield of 38% (Entry 6), while other transition metals, such as cobalt­(II), palladium­(II), and osmium­(II) compounds, proved to be ineffective (Entry 9). The reaction was further examined at various wavelengths (Entry 10), and 450 nm proved to be optimal. Shortening the reaction time to 24 h resulted in a yield of 50% (Entry 11). The addition of the preligand (C_6_H_5_O)_2_P­(O)­OH inhibited product formation, and trace amounts of the desired product were formed (Entry 12).

**Table 1:**
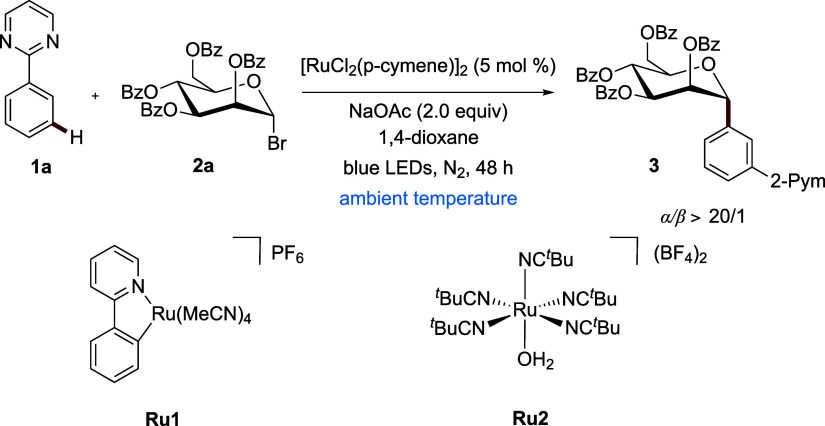
Optimization of the Photoinduced *meta*-C–H Glycosylation at RT^a^

entry	derivatives	yields/%^b^
1	none	66
2	K_2_CO_3_/K_3_PO_4_/NEt_3_	N.D./61/traces
3	NMP/THF/PhMe	traces/13/14
4	without [RuCl_2_(*p-*cymene)]_2_ or light or NaOAc	N.D.
5	[Ru(MesCO_2_)_2_(*p*-cymene)]	9
6	[Ru(OAc)_2_(*p*-cymene)]	38
7	**Ru1** as catalyst	N.D.
8	**Ru2** as catalyst	N.D.^c^
9	CoBr_2_/Pd(OAc)_2_/CoCl_2_/[OsCl_2_-*p*-cymene)]_2_	N.D.
10	390 nm/427 nm/440 nm	20/10/34
11	24 h instead of 48 h	50
12	with 20 mol % (C_6_H_5_O)_2_P(O)OH	traces^d^

a Reaction conditions: **1a** (0.1 mmol), **2a** (0.2 mmol), catalyst (5 mol %), base (0.2 mmol), solvent (1.0 mL), 450 nm, 25–33 °C, 48 h, under N_2_.

b Yield of isolated product.

c 21 h.

d 24 h. NMP: *N*-methyl-2-pyrrolidone. 2-Pym: 2-phenylpyrimidine. THF: tetrahydrofuran.

Next, the influence of the phosphine ligand was explored in more detail. For the photochemical reaction, control experiments were conducted both with and without the phosphine ligand (Scheme 2a). Interestingly, the reaction proceeded only in the absence of the phosphine ligand (Scheme 2a). Likewise, the *p*-cymene-free ruthenium­(II) complex **Ru3** proved to be ineffective under otherwise identical reaction conditions (Scheme 2b). This is the first example of ruthenium-catalyzed *meta*-C–H glycosylation under photochemical conditions without the use of a phosphine ligand.

Given the unique power of photoinduced ruthenium-catalyzed*meta*-selective-C–H glycosylation in the absence of phosphines, computational studies were carried out at the PBE0-D4/def2-TZVP-SDD-SMD­(1,4-dioxane)//PBE0-D3­(BJ)/def2-SVP level of theory (Scheme 2c,d).^13^ Under the photoexcitation conditions,^15^ blue-light irradiation of the *in situ* formed ruthenium­(II)-cyclometalated complex **int1**, followed by intersystem crossing, generated a long-lived excited state of the ruthenium­(II)-complex **int1** ^**tri**^, which can undergo the inner-sphere single electron transfer to the mannosyl bromide and produce the *C*-centered radical and the ruthenium­(III)-cyclometalated complex **int3**. The computed energy barrier for the photoinduced SET was calculated to be 5.3 kcal mol^–1^. The energetic span for the direct ISET conversion from **int1** to **int3** was determined to be 10.0 kcal mol^–1^, which is less favorable compared with the photoinduced catalysis under blue-light irradiation. Subsequently, the nucleophilic mannosyl radical undergoes radical attack on the *para* position to the Ru–C bond of electrophilic **int3** *via* transition state **TS4** ^**oss**^, generating the ruthenium-carbene complex **int5**. It is worth noting that the coordination of the phosphine ligand exhibited the maximum absorption of the intermediate **int1** ^**p**^ at 347.43 nm, which locates outside the visible-light range (Scheme 2d). Furthermore, the energy required for the radical attack step is not accessible at room temperature. These findings are consistent with the observed experimental results (Scheme 2a).

Subsequently, the general applicability of our photoinduced *meta*-C­(sp^2^)–H glycosylation was investigated (Scheme 3). Different substitution patterns on the pyrimidine as well as on the glycoside were evaluated. The benzoyl-protected glycoside **2a** was well tolerated, and the acetate-protected glycoside **2b** also furnished acceptable results with a 54% yield. Notably, electron-withdrawing glycosyl donor **2n** resulted in a higher yield of 37% for product **22** compared with 17% of product **23** obtained from electron-donating glycosyl donor **2o**. Next, a range of *para*-substituted arenes **1** was tolerated. Methyl-substituted pyrimidines **1b** demonstrated good tolerance, achieving an 80% yield. In contrast, electrophilic halogen substituents **10** and **13** led to reduced yields, although no side reactions were observed. Interestingly, chain extension to ethyl arenes **7** also proved feasible, affording a 36% yield. To further highlight the efficiency of the developed photocatalysis, a variety of glycoside components were investigated. Rhamnose **16** was well received as a glycoside component, providing a 47% yield. The combination of α/β-arabinose bromides highlighted the efficacy of our photoinduced reaction, affording exclusively α-product **17**. Furthermore, galactose **19** and fucose **20** proved to be viable. Beyond substituted arenes, imine groups **14** could also be employed as directing groups. Glucosides such as **15** and **18** proved to be incompatible with ruthenium catalysis. For a deeper understanding of the influence of the substituent at the *C*2 position, a more detailed investigation was conducted (Scheme 4). For further analysis, various substituted benzoyl groups were tested at the *C*2 position of the mannose unit. Fluoro-substituted glycosyl donor **25** resulted in a slightly higher yield of 32% compared with standard benzoyl group **24**, which afforded 30%.

However, when a redox-relevant *para*-NO_2_ substituted aryl group or a 2-deoxyglycoside, no *meta*-C­(sp^2^)–H glycosylation occurred.

**Scheme 3:**
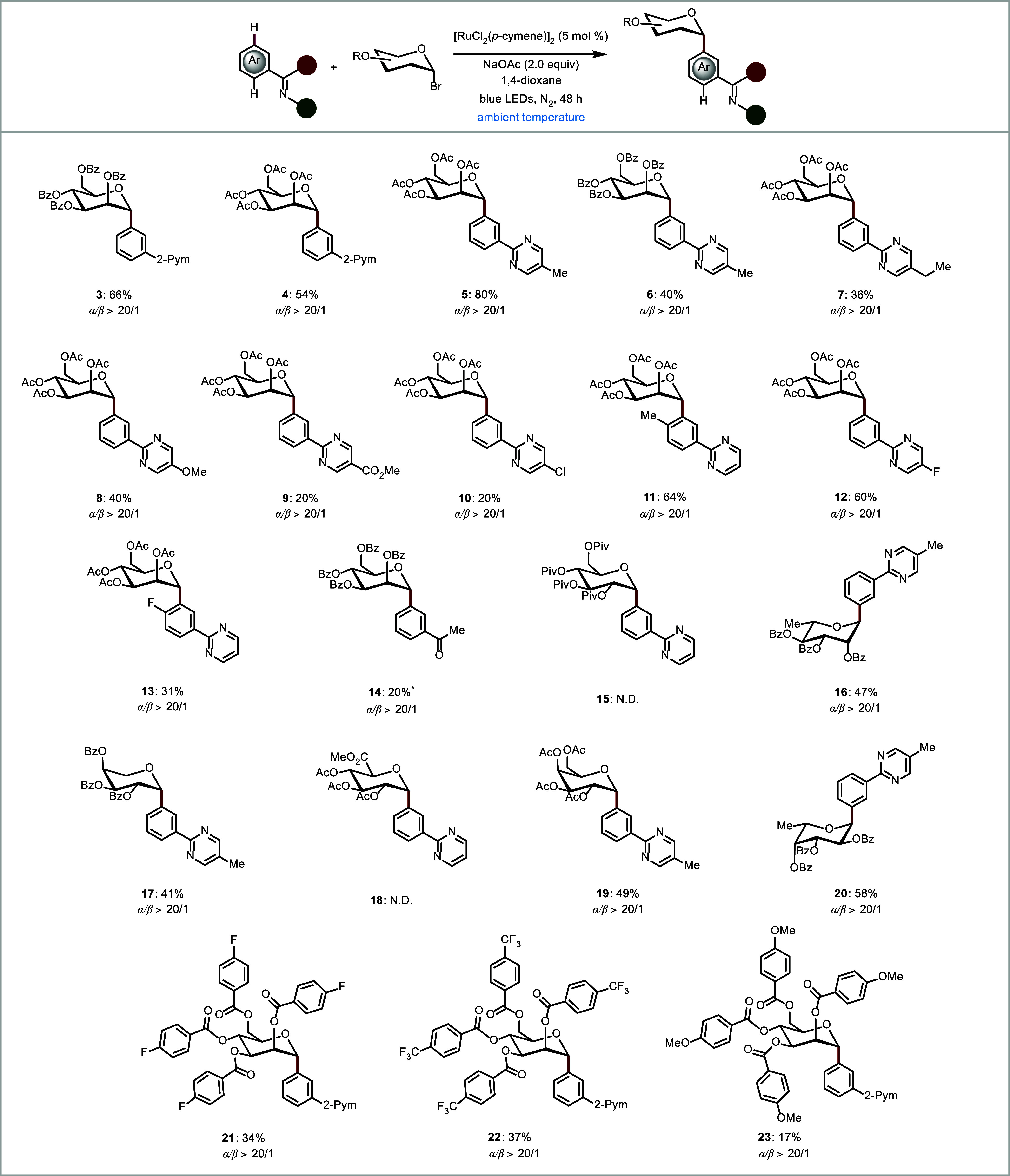
Photoinduced Ruthenium-Catalyzed C–H Glycosylation of Heteroarenes and Glycosyl Bromides^a^

a Reaction conditions: **1a** (0.1 mmol), **2a** (0.2 mmol), catalyst (5 mol %), base (0.2 mmol), solvent (1.0 mL), 450 nm, 25–33 °C, 48 h, under N_2_. *With 3,4,5-trimethoxyphenyl (TMP) protected ketimine (0.1 mmol) and HCl for workup (1 mL).^13^

**Scheme 4:**
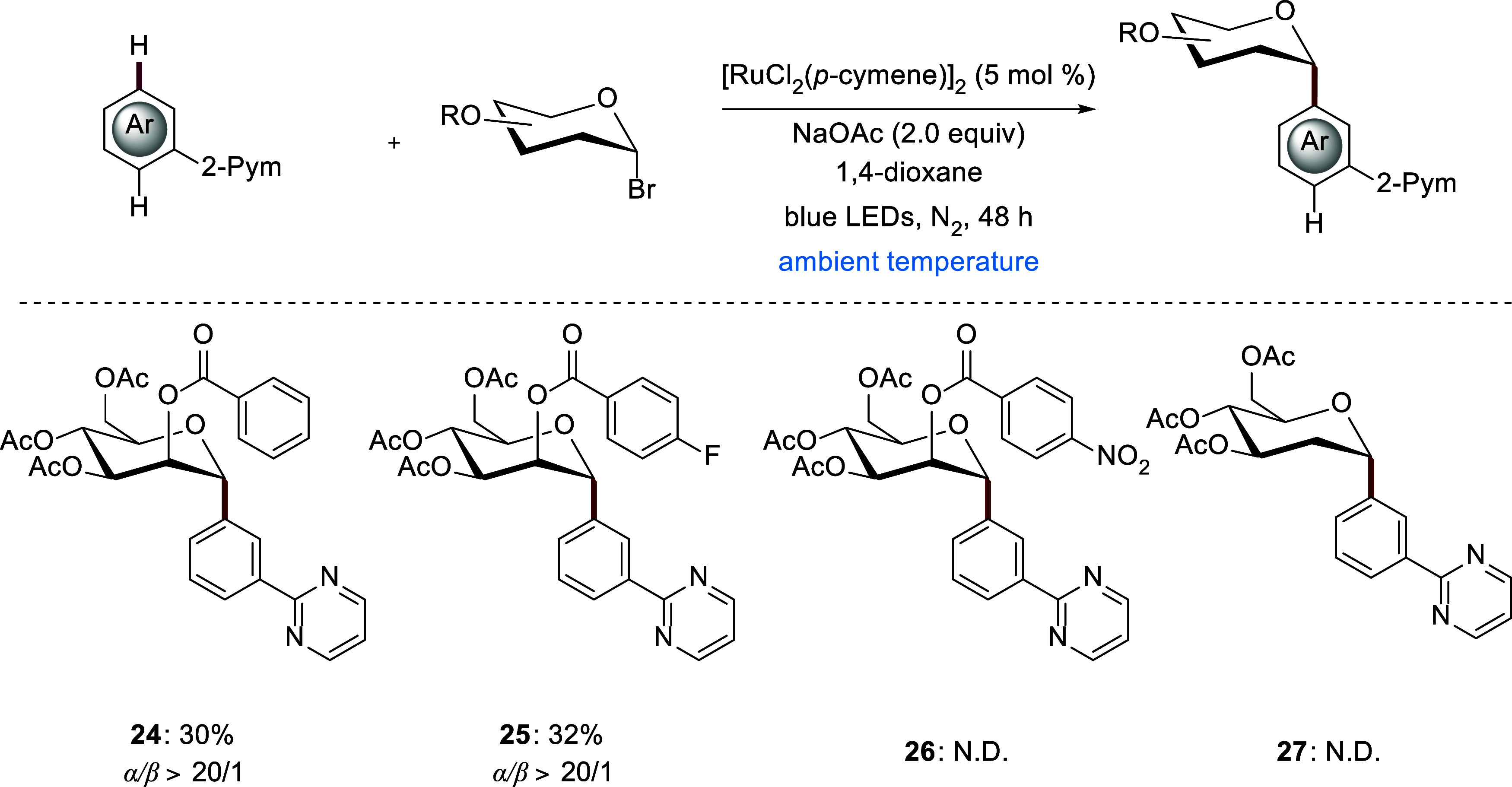
Influence of the Neighboring Group Effect with *para*-Substituted Benzoyl Groups

Finally, the robustness of the photoinduced *meta*-*C*-aryl glycoside arrangement was reflected by employing structurally complex glycosyl bromides (Scheme 5). Saccharides were transformed into the corresponding *C*-aryl glycosides, providing access to various complex natural products and drug hybrid structures, reflecting the ruthenium­(II) photocatalysts’ unique chemo- and stereoselectivity. Thus, naproxen **28** and ibuprofen **30** proved to be suitable for late-stage diversification, both yielding 87% of the desired product. Ciprofibrate **29** was fully tolerated despite the presence of two chloro groups. Cyclic compounds were also well tolerated. Namely, cyclopropyl, cyclopentyl, and cyclohexyl glycosides furnished desired products **31**–**33**. Subsequently, glycolipids were tested, and both butyric acid ester **34** and valeric acid ester **35** were obtained with an outstanding anomeric α-selectivity.

**Scheme 5:**
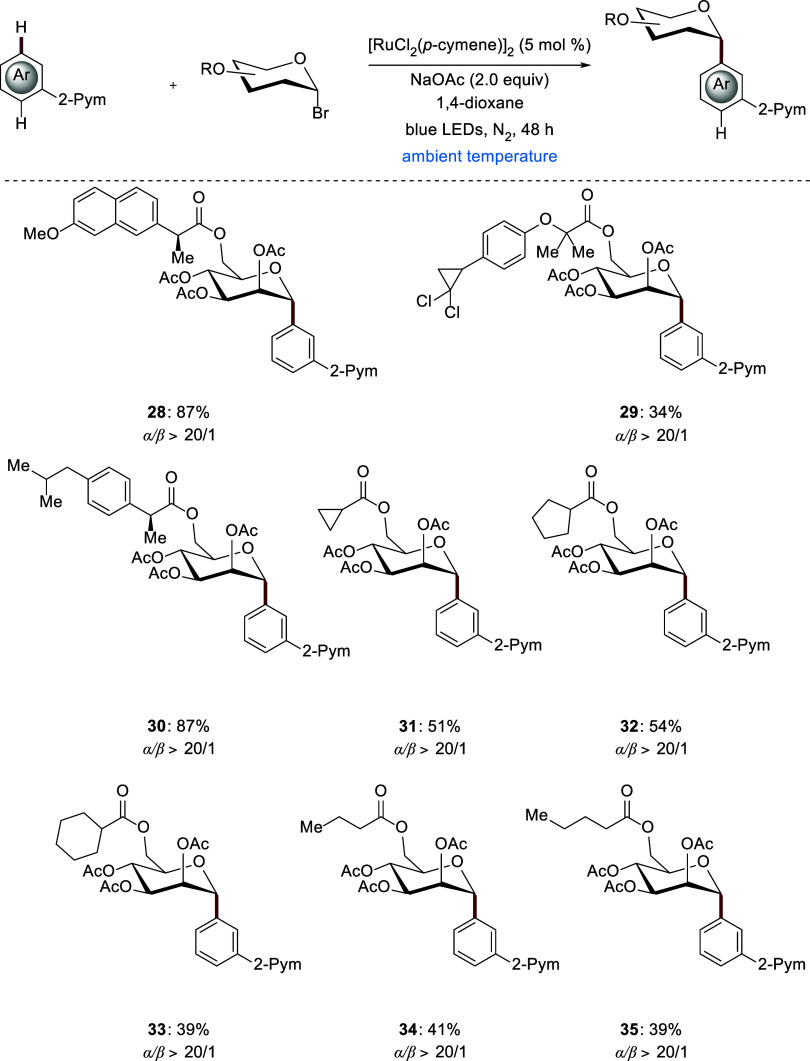
Late-Stage Transformation for *meta*-*C*(sp^2^)–H Glycosylation

## Conclusions

In summary, we have developed a selective phosphine-ligand-free photocatalyzed ruthenium­(II) *meta*-C–H glycosylation strategy. This approach does not require the use of an exogenous photocatalyst and utilizes readily available glycosyl bromides. The products were obtained with excellent *meta*- and anomeric α-selectivity. The robustness of the catalysis was demonstrated with pharmaceutically relevant drug moieties. Our study reveals a new platform for the synthesis of biologically relevant *meta*-*C*-aryl glycosides under mild and robust photoinduced ruthenium catalysis.

## Materials and Methods

### General Procedure for the Photoinduced Ruthenium-Catalyzed *meta*-C–H Glycosylation

Heteroarene **1** (0.1 mmol, 1.0 equiv), [RuCl_2_(*p*-cymene)]_2_ (3.1 mg, 5.0 mol %), and the glycoside **2** (0.2 mmol, 2.0 equiv) were added to a 10 mL vial with a stirring bar and transferred to the glovebox. Then, NaOAc (16.4 mg, 0.2 mmol, 2.0 equiv) and 1,4-dioxane (1.0 mL) were sequentially added, and the vial was closed with a plastic lid. Parafilm was then wrapped around, and the mixture was stirred under visible light irradiation (using 2 × Kessil A360N). The reaction was carried out at ambient temperature (25–33 °C). After 48 h, the resulting reaction mixture was diluted with CH_2_Cl_2_ and concentrated in *vacuo*. Purification of the residue by column chromatography on silica gel (*n*-hexane/EtOAc) yielded the product (refer to the Supporting Information for additional details).

## Supplementary Material



## Notes

All experimental procedures; optimization studies; mechanistic experiments; characterization of the new compounds; NMR spectra; and DFT studies (PDF)
