# Supplementary material for: Photoinduced Ruthenium-Catalyzed meta-C–H Glycosylation
Source: ACS Catal. 2025 Jun 5;15(12):10542–9. doi: 10.1021/acscatal.5c02183 (PMC12186293; doi:10.1021/acscatal.5c02183)
Supplement: Supplementary file 1 [file cs5c02183_si_001.pdf]

Supporting Information

Photoinduced Ruthenium-Catalyzed *meta*-C–H Glycosylation

J. Pöhlmann,[a,b] B. Yuan,[a] R. Purushothaman,[a] J. Wu,[a] L. Ackermann*[a,b]

[a] WISCh (Wöhler Research Institute for Sustainable Chemistry), Georg-August-Universität Göttingen, Tammannstraße 2, 37077 Göttingen, Germany

Email: Lutz.Ackermann@chemie.uni-goettingen.de

[b] DZHK (German Centre for Cardiovascular Research), Potsdamer Straße 58, 10785, Berlin, Germany.

Table of Contents

General Remarks....3

General Procedure....4

Photochemical Setup....4

Optimization of the Reaction Condition....5

Examination of Glycosyl Halides....12

Control Experiments for the Need of Phosphine....13

Kinetic Experiment....14

Determination of Quantum Yield....16

On/Off Experiment....17

Influence of *para*-cymene....19

Reaction of a *para*-cymene-free Complex....19

Computational Studies....20

List of Substrate Scope for *meta*-C–H Glycosylation....67

Characterization Data of Products....69

References....98

^{1}H and ^{13}C NMR Spectra....100

General Remarks

Catalytic reactions were conducted under a nitrogen atmosphere (N_2), utilizing predried glassware and standard Schlenk techniques. For purity assurance, 1,4-dioxane was subjected to sodium (Na) drying followed by distillation under N_2. Remaining chemicals were commercially procured and utilized without additional purification steps. Cyclometallated ruthenium complexes **Ru1**, **Ru2**, and **Ru3** were synthesized according to the reported procedure.[1] The preparation of glycoside bromides adhered to well-established procedures documented in the literature.[2] The glycosyl donors were always freshly prepared and used directly, as some glycosyl donors lose reactivity or are not stable. Yields were assessed for individual compounds, with estimated purity levels surpassing >95%, as verified through ^{1}H NMR analysis.

Flash chromatography was carried out employing Merck silica gel 60 with particle sizes ranging from 40 to 63 µm.

NMR spectra were obtained utilizing a range of instruments, which included the Bruker Avance Neo 600, Bruker Avance III HD 500, Bruker Avance III HD 400, Bruker Avance Neo 400, Bruker Avance III HD 300. Chemical shifts (δ) were reported in parts per million (ppm) relative to the residual solvent peak. ^{1}H NMR information is presented in the following format: chemical shift, multiplicity (s = singlet, d = doublet, t = triplet, q = quartet, dd = doublet of doublet, m = multiplet), coupling constants *J* (in Hertz), and the number of protons. Chemical shifts are stated as δ-values in parts per million (ppm) referenced to the residual proton peak of the deuterated solvent (^{1}H ($CDCl_3$): 7.26 ppm) or the carbon peak of the solvent (^{13}C ($CDCl_3$): 77.16 ppm). The evaluation of the NMR spectra was carried out using the MestReNova v 14.2.0-26256 from Mestrelab Research.

IR spectra were captured employing a Bruker FTIR alpha-P device.

EI-MS data were collected utilizing a Joel AccuTof at 70 eV, whereas **ESI-MS** analysis was carried out employing a Bruker Daltonic micrOTOF and maXis instrument. High-resolution mass spectrometry (**HR-MS**) was recorded on Exactive Plus, 2.9 Build 290492 by Thermo Fisher Scientific.

General Procedure

Heteroarene **1** (0.1 mmol, 1.0 equiv.), $[RuCl_2(p\text{-cymene})]_2$ (3.1 mg, 5.0 mol %), and the glycoside **2** (0.2 mmol, 2.0 equiv) were added to a 10 mL vial with a stirring bar and transferred to the glovebox. Then, NaOAc (16.4 mg, 0.2 mmol, 2.0 equiv) and 1,4-dioxane (1.0 mL) were sequentially added and the vial was closed with a plastic lid. Parafilm was then wrapped around and the mixture was stirred under visible light irradiation (using 2 × Kessil A360N). The reaction was carried out at ambient temperature (25–33 °C). After 48 hours the resulting reaction mixture was diluted with CH_2Cl_2 and concentrated in *vacuo*. Purification of the residue by column chromatography on silica gel (*n*-hexane/EtOAc) yielded the product.

Photochemical Setup

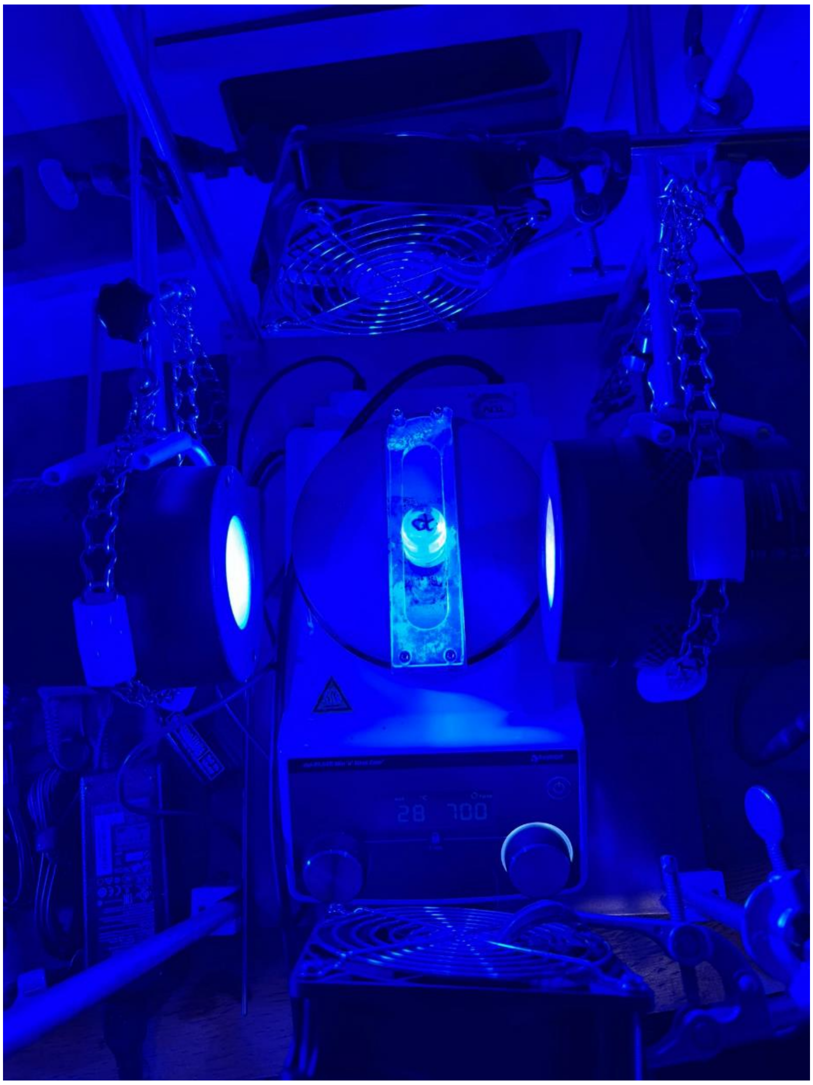

Figure S1. Reaction setup equipped with 2 × Kessil A360N lamps and 2 × fans.

Optimization of the Reaction Condition

Table 1. Optimization of bases for photoinduced ruthenium-catalyzed *meta*-C–H glycosylation.

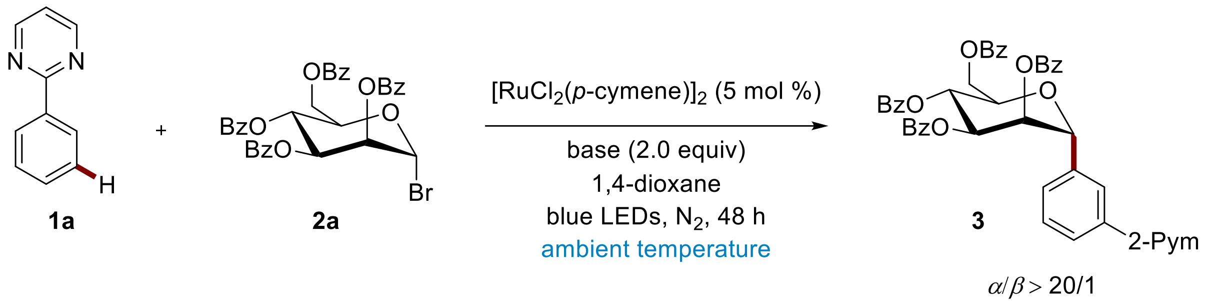

Entry	Base	Yield [%][a]
1	$NaHCO_3$	Traces
2	K_3PO_4	61
3	KOAc	Traces
4	NEt_3	Traces
5	NaOAc	66

Reaction conditions: **1a** (0.1 mmol), **2a** (0.2 mmol), catalyst (5 mol %), base (0.2 mmol), solvent (1.0 mL), 450 nm, 25–33 °C, 48 h, under N_2. [a] Yield of isolated product. 2-Pym: 2-Phenylpyrimidine.

Table 2. Optimization of catalyst loading for photoinduced ruthenium-catalyzed *meta*-C–H glycosylation.

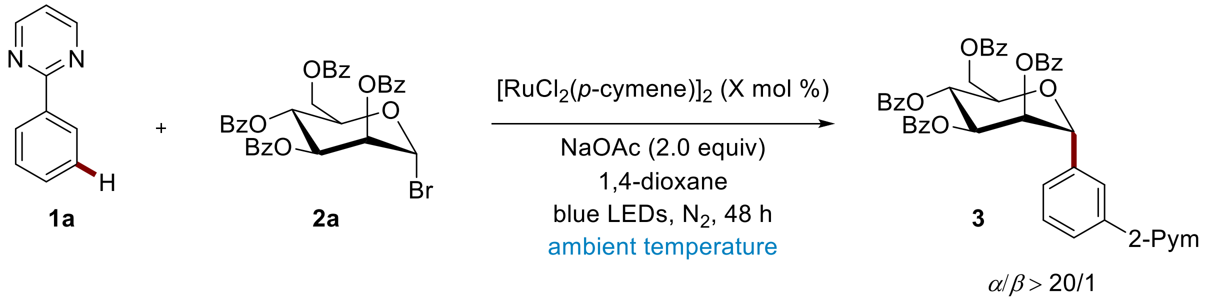

Entry	Catalyst Loading [mol %]	Yield [%][a]
1	20	43
2	10	42
3	5	66

Reaction conditions: **1a** (0.1 mmol), **2a** (0.2 mmol), catalyst (5 mol %), base (0.2 mmol), solvent (1.0 mL), 450 nm, 25–33 °C, 48 h, under N_2. [a] Yield of isolated product. 2-Pym: 2-Phenylpyrimidine.

Table 3. Optimization of solvents for photoinduced ruthenium-catalyzed *meta*-C–H glycosylation.

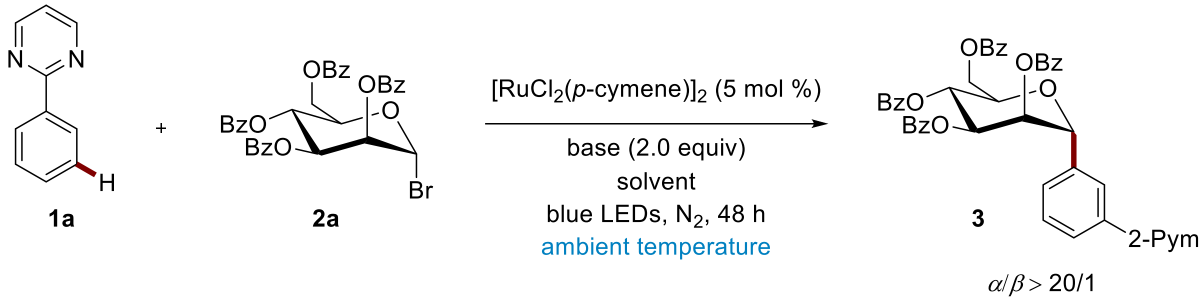

Entry	Solvent	Yield [%][a]
1	NMP	Traces
2	PhMe	14
3	THF	13
4	PEG400	26

Reaction conditions: **1a** (0.1 mmol), **2a** (0.2 mmol), catalyst (5 mol %), base (0.2 mmol), solvent (1.0 mL), 450 nm, 25–33 °C, 48 h, under N_2.[a] Yield of isolated product. *N*-Methyl-2-pyrrolidone. PEG400: Polyethylenglykol 400. 2-Pym: 2-Phenylpyrimidine. THF: Tetrahydrofuran.

Table 4. Optimization of catalysts for photoinduced ruthenium-catalyzed *meta*-C–H glycosylation.

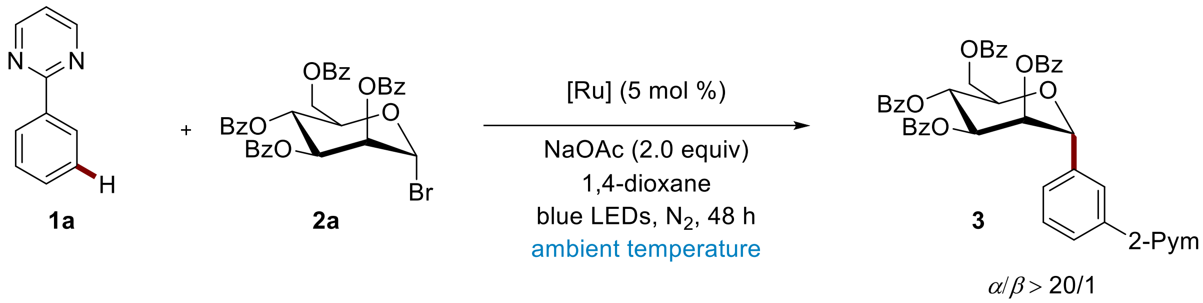

Entry	Catalyst	Yield [%][a]
1	[Ru1]	N.D.
2	[Ru2]	N.D.[b]
3	[Ru4]	N.D.
4	[Ru5]	N.D.
5	[Ru6]	N.D.
6	[Ru7]	N.D.
7	[RuCl$_2$(*p*-cymene)]$_2$	66
8	[Ru(MesCO$_2$)$_2$(*p*-cymene)]	9
9	[Ru(OAc)$_2$(*p*-cymene)]	38
10	[Ru(OPiv)$_2$(*p*-cymene)]	Traces
11	RuCl$_3$	N.D.

12	$[Ru(^tBuCN)_6][PF_6]_2$	N.D.
13	$Pd(OAc)_2$	N.D.
14	$CoBr_2$	N.D.
15	$CoCl_2$	N.D.
16	$[OsCl_2$-(*p*-cymene)$]_2$	N.D.

Reaction conditions: **1a** (0.1 mmol), **2a** (0.2 mmol), catalyst (5 mol %), base (0.2 mmol), solvent (1.0 mL), 450 nm, 25–33 °C, 48 h, under N_2. [a] Yield of isolated product. [b] For 21 h instead of 48 h. 2-Pym: 2-Phenylpyrimidine.

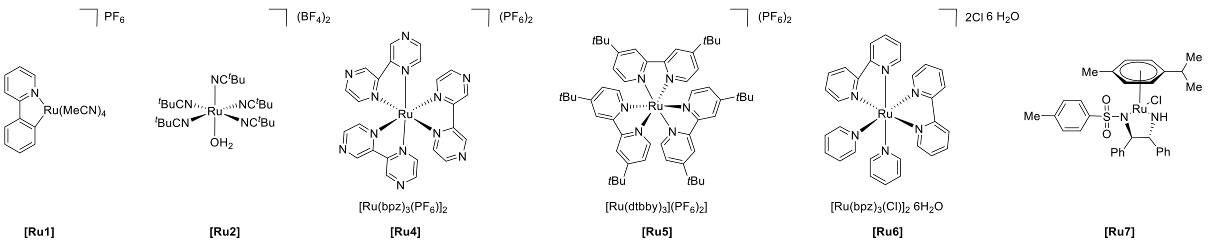

Table 5. Optimization of additives for photoinduced ruthenium-catalyzed *meta*-C–H glycosylation.

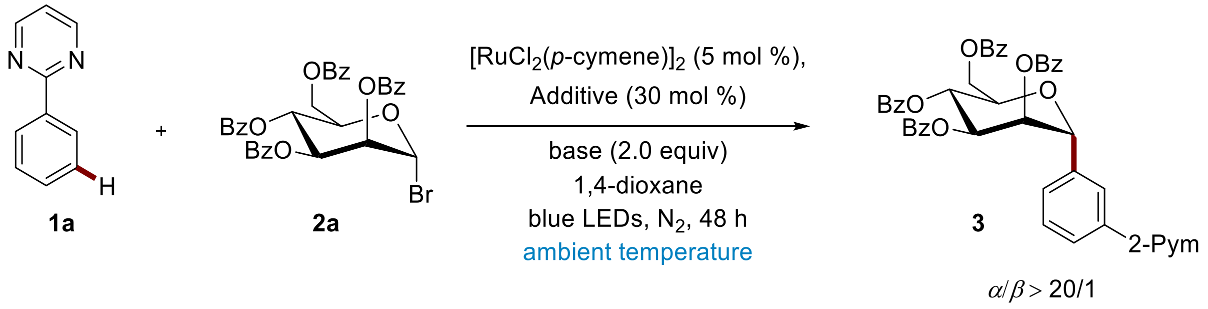

Entry	Base	Additive	Solvent	Yield [%][a]
1	NaOAc	$(C_6H_5O)_2P(O)OH$	1,4-dioxane	Traces
2	NaOAc	$(C_6H_5O)_2P(O)OH$	NMP	Traces
3	NaOAc	$P(4\text{-}CF_3\text{-}C_6H_4)_3$	1,4-dioxane	Traces
4	K_2CO_3	$(C_6H_5O)_2P(O)OH$	1,4-dioxane	N.D.

Reaction conditions: **1a** (0.1 mmol), **2a** (0.2 mmol), catalyst (5 mol %), base (0.2 mmol), additive (30 mol %), solvent (1.0 mL), 450 nm, 25–33 °C, 48 h, under N_2. [a] Yield of isolated product. 2-Pym: 2-Phenylpyrimidine. NMP: *N*-Methyl-2-pyrrolidin.

Table 6. Optimization of molarity for photoinduced ruthenium-catalyzed *meta*-C–H glycosylation.

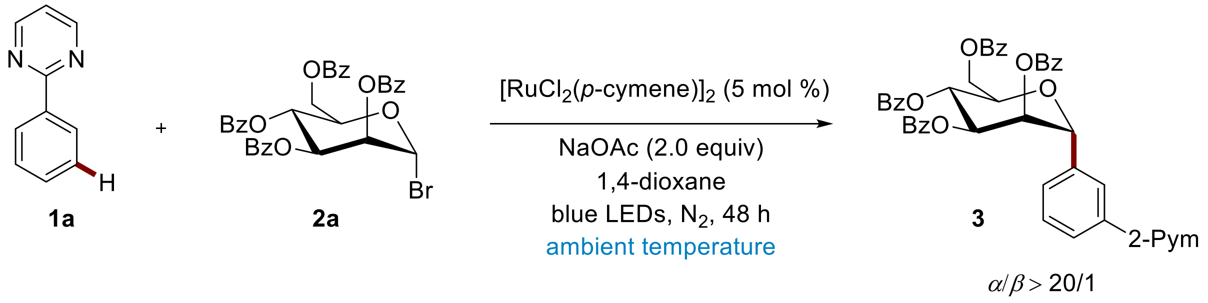

Entry	Solvent	Yield [%][a]
1	2.0 mL instead of 1.0 mL	33
2	0.5 mL instead of 1.0 mL	41
3	0.5 mL instead of 1.0 mL	47[b]

Reaction conditions: **1a** (0.1 mmol), **2a** (0.2 mmol), catalyst (5 mol %), base (0.2 mmol), solvent (1.0 mL), 450 nm, 25–33 °C, 48 h, under N_2.[a] Yield of isolated product. [b] 0.2 mmol scale. 2-Pym: 2-Phenylpyrimidine.

Table 7. Control experiments for photoinduced ruthenium-catalyzed *meta*-C–H glycosylation.

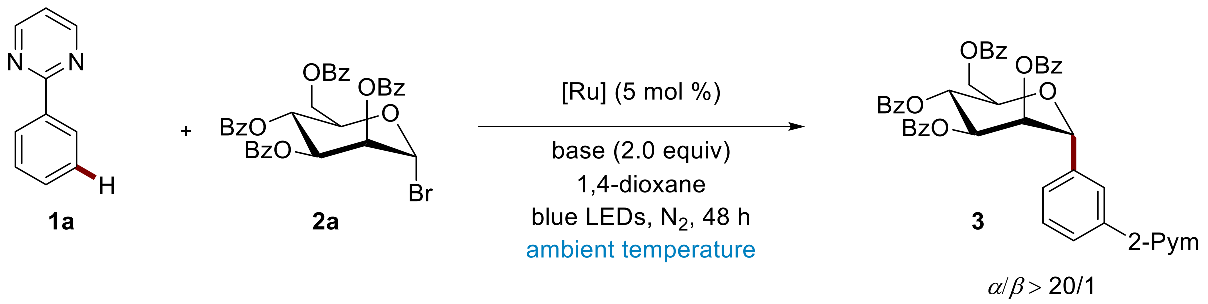

Entry	Catalyst	Base	Solvent	Temp. [°C]	Light	Yield [%][a]
1	-	NaOAc	1,4-dioxane	30	-	N.D.
2	[RuCl$_2$(*p*-cymene)$_2$]	-	1,4-dioxane	30	Blue LEDs	N.D.
3	[RuCl$_2$(*p*-cymene)$_2$]	NaOAc	H_2O	30	Blue LEDs	N.D.
4	[Ru(OAc)$_2$(*p*-cymene)]	K_2CO_3	1,4-dioxane	30	Blue LEDs	N.D.
5	[Ru1]	NaOAc	1,4-dioxane	60	-	5
6	[RuCl$_2$(*p*-cymene)$_2$]	NaOAc	1,4-dioxane	30	-	N.D.
7	[RuCl$_2$(*p*-cymene)$_2$]	NaOAc	1,4-dioxane	60	-	N.D.
8	[RuCl$_2$(*p*-cymene)$_2$]	NaOAc	1,4-dioxane	30[b]	-	N.D.
9	[Ru1]	NaOAc	1,4-dioxane	30[b]	-	5

Reaction conditions: **1a** (0.1 mmol), **2a** (0.2 mmol), catalyst (5 mol %), base (0.2 mmol), solvent (1.0 mL), 450 nm, 25–33 °C, 48 h, under N_2.[a] Yield of isolated product. [b] Heated in a metal block. 2-Pym: 2-Phenylpyrimidine.

Table 8. Different ratio of glycosylbromide for photoinduced ruthenium-catalyzed *meta*-C–H glycosylation.

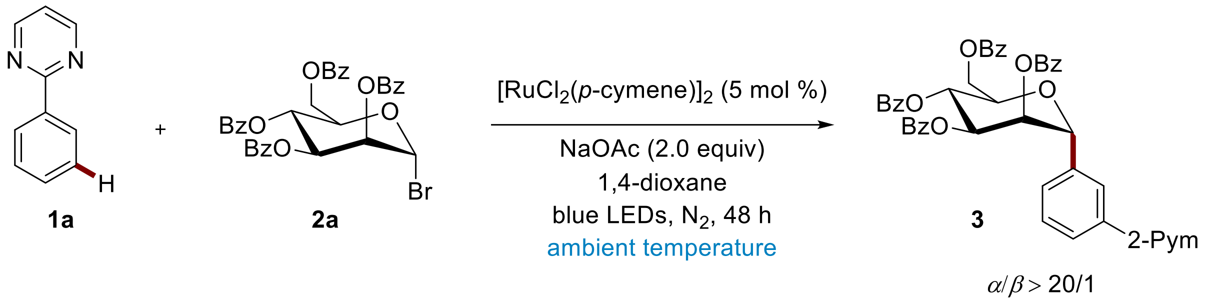

Entry	Deviation from the standard conditions	Yield [%][a]
1	1.0 equiv of glycosylbromide instead of 2.00 equiv	21
2	1.5 equiv of glycosylbromide instead of 2.00 equiv	31
3	3.0 equiv of glycosylbromide instead of 2.00 equiv	33

Reaction conditions: **1a** (0.1 mmol), **2a** (0.2 mmol), catalyst (5 mol %), base (0.2 mmol), solvent (1.0 mL), 450 nm, 25–33 °C, 48 h, under N_2. [a] Yield of isolated product. 2-Pym: 2-Phenylpyrimidine.

Table 9. Optimization of wavelength for photoinduced ruthenium-catalyzed *meta*-C–H glycosylation.

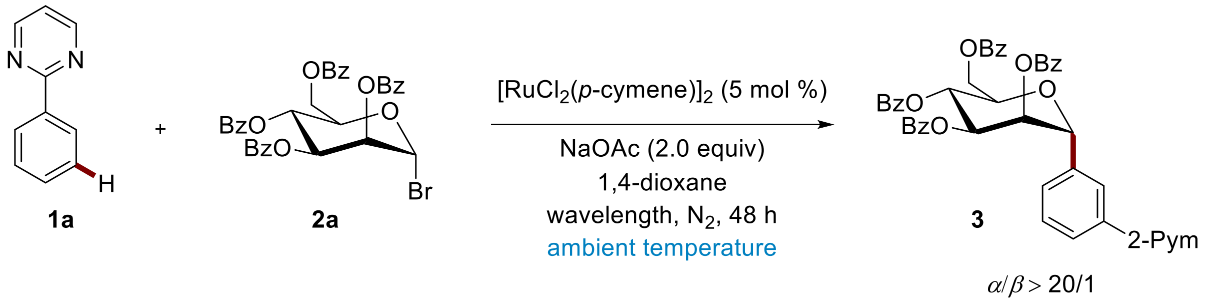

Entry	Wavelength	Yield [%][a]
1	390 nm	20
2	440 nm	34
3	427 nm	10

Reaction conditions: **1a** (0.1 mmol), **2a** (0.2 mmol), catalyst (5 mol %), base (0.2 mmol), solvent (1.0 mL), 450 nm, 25–33 °C, 48 h, under N_2. [a] Yield of isolated product. 2-Pym: 2-Phenylpyrimidine.

Table 10. Optimization of ratio for photoinduced ruthenium-catalyzed *meta*-C–H glycosylation.

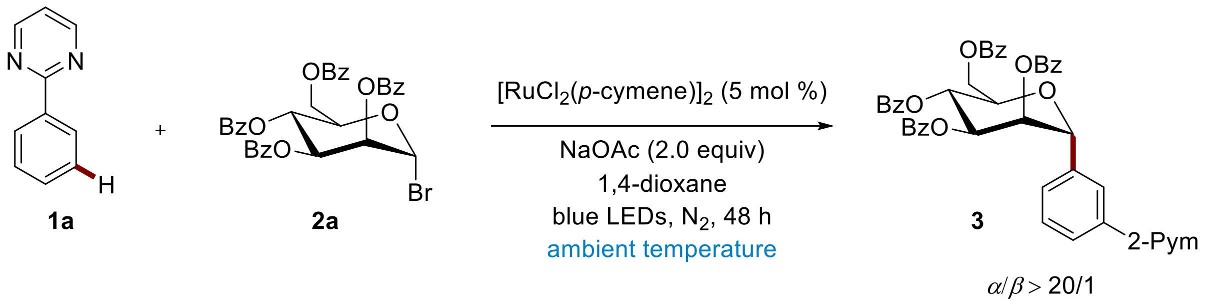

Entry	Deviation from the standard conditions	Yield [%][a]	1a left [%][a]	2a left [%][a]
1	None	66	-	-
2	**2a** (1.0 equiv) **1a** (2.0 equiv)	55	30	-
3	**2a** (3.0 equiv) **1a** (1.0 equiv)	18	70	72
4	**2a** (4.0 equiv) **1a** (1.0 equiv)	23	62	80
5	Molecular sieve 4Å (30 mg)	42	-	-

Reaction conditions: **1a** (0.2 or 0.1 mmol), **2a** (0.1 or 0.3 or 0.4 mmol), catalyst (5 mol %), base (0.2 mmol), solvent (1.0 mL), 450 nm, 25–33 °C, 48 h, under N_2. [a] Yield of isolated product. 2-Pym: 2-Phenylpyrimidine.

Table 11. Optimization of rutheniumaquacomplex for photoinduced ruthenium-catalyzed *meta*-C–H glycosylation.

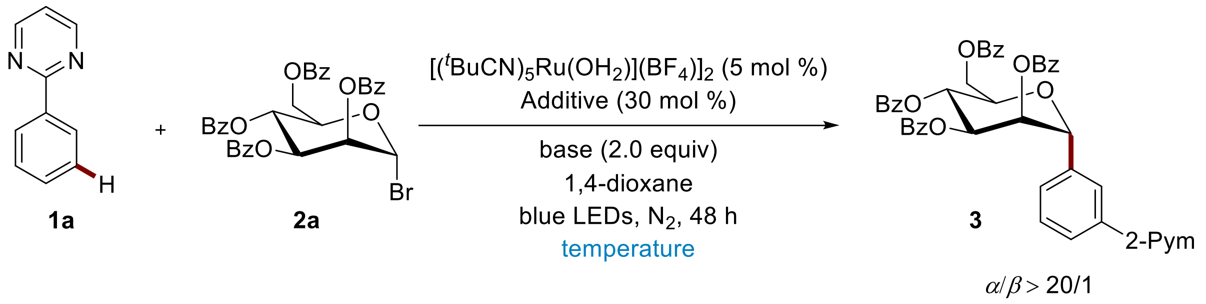

Entry	Base/Additive	Time [h]	Light/Heating Source	Yield [%]
1	NaOAc	21	Blue LEDs/ ambient temperature	N.D.
2	NaOAc<br>$P(4\text{-}CF_3\text{-}C_6H_4)_3$ (20 mol %)	21	Blue LEDs/ ambient temperature	N.D.
3	K_2CO_3	21	Blue LEDs/ ambient temperature	N.D.
4	K_2CO_3<br>$P(4\text{-}CF_3\text{-}C_6H_4)_3$ (20 mol %)	21	Blue LEDs/ambient temperature	N.D.
5	NaOAc	21	-/40°C[b]	N.D.

6	NaOAc<br>$P(4\text{-}CF_3\text{-}C_6H_4)_3$ (20mol %)	21	-/40°C[b]	N.D.
7	K_2CO_3	21	-/40°C[b]	N.D.
8	K_2CO_3<br>$P(4\text{-}CF_3\text{-}C_6H_4)_3$ (20mol %)	21	-/40°C[b]	N.D.

Reaction conditions: **1a** (0.1 mmol), **2a** (0.2 mmol), catalyst (5 mol %), base (0.2 mmol), solvent (1.0 mL), 450 nm, 25–33 °C, 48 h, under N_2. [a] Yield of isolated product. [b] Heated in a metal block. 2-Pym: 2-Phenylpyrimidine.

Table 12. Optimization of iminereaction for photoinduced ruthenium-catalyzed *meta*-C–H glycosylation.

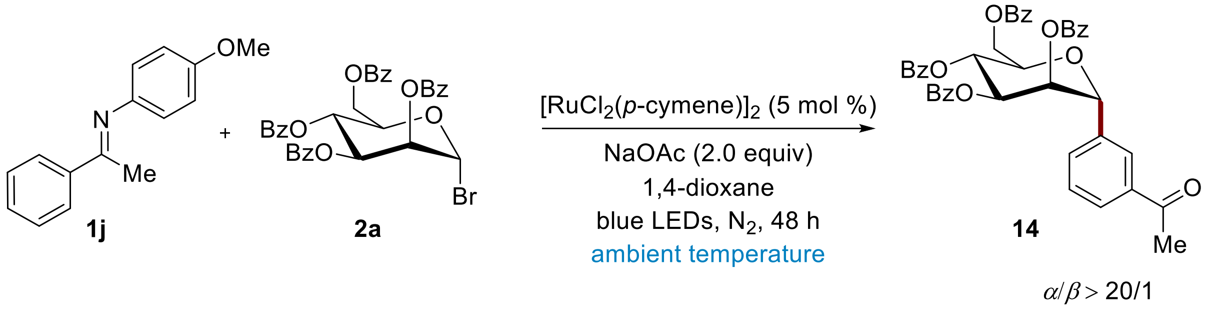

Entry	Deviation from the standard conditions	Yield[a]
1	None	20
2	Molecular sieve 4Å 50 mg	19
3	Molecular sieve 4Å 100 mg	25
4	$[Ru(OAc)_2(p\text{-cymene})]$	4
5	$[Ru(OAc)_2(p\text{-cymene})]$ without NaOAc	2
6	$[RuCl_2(p\text{-cymene})]_2$, NMP	N.D.

Reaction conditions: **1j** (0.1 mmol), **2a** (0.2 mmol), catalyst (5 mol %), base (0.2 mmol), solvent (1.0 mL), 450 nm, 25–33 °C, 48 h, under N_2. [a] Yield of isolated product. NMP: *N*-Methyl-2-pyrrolidin. 2-Pym: 2-Phenylpyrimidine.

Examination of Glycosyl Halides

Table 13. Testing different glycosyl donors for photoinduced ruthenium-catalyzed *meta*-C–H glycosylation.

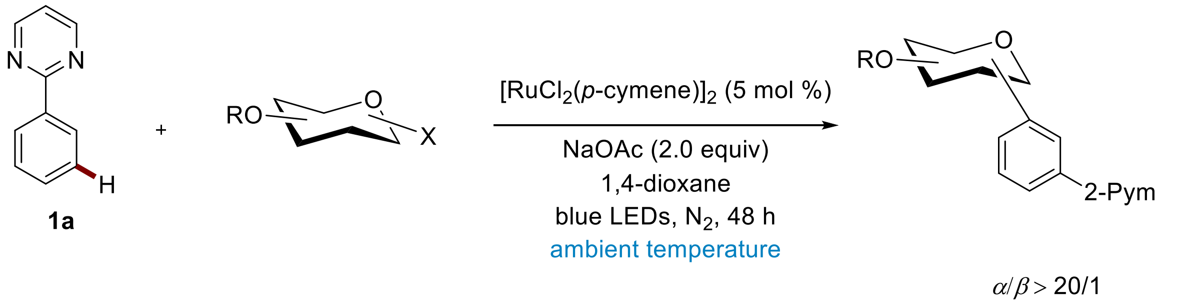

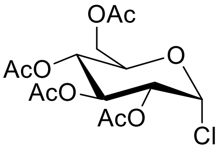

2c

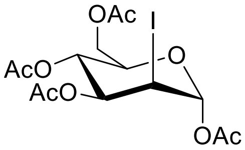

2d

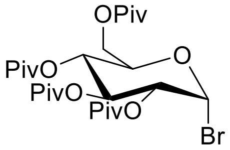

2e

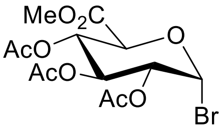

2f

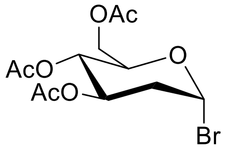

2g

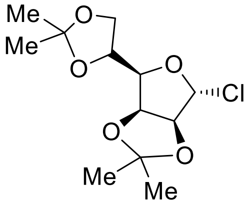

2h

Entry	Glycosyl Halide	Yield [%][a]
1	**2c**	N.D.
2	**2d**	N.D.
3	**2e**	N.D.
4	**2f**	N.D.
5	**2g**	N.D.
6	**2h**	N.D.

Reaction conditions: **1a** (0.1 mmol), **2** (0.2 mmol), catalyst (5 mol %), base (0.2 mmol), solvent (1.0 mL), 450 nm, 25–33 °C, 48 h, under N_2. [a] Yield of isolated product. 2-Pym: 2-Phenylpyrimidine.

Control Experiments for the Need of Phosphine

Table 14. Control experiments for the need of the phosphine ligand for photoinduced ruthenium-catalyzed *meta*-C–H glycosylation.

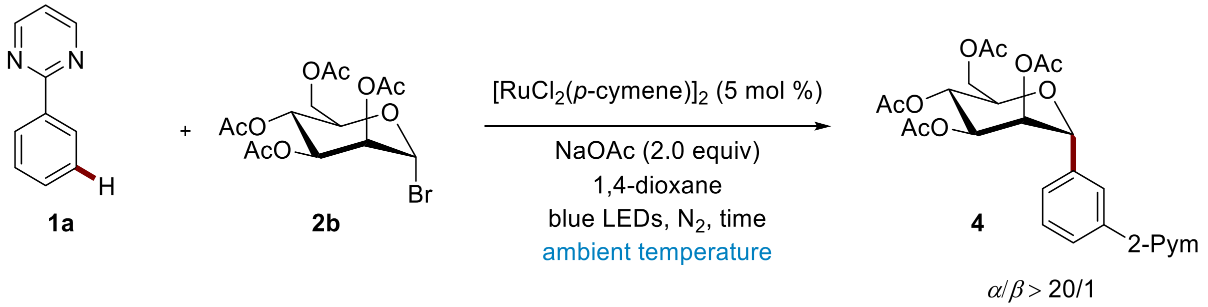

Entry	Additive	Base	Time [h]	Yield [%][a]
1	-	NaOAc	48	54
2	$P(4\text{-}CF_3\text{-}C_6H_4)_3$ (10 mol %)	NaOAc	48	N.D.

Reaction conditions: **1a** (0.1 mmol), **2b** (0.2 mmol), catalyst (5 mol %), base (0.2 mmol), solvent (1.0 mL), 450 nm, 25–33 °C, 48 h, under N_2. [a] Yield of isolated product. 2-Pym: 2-Phenylpyrimidine.

Table 15. Control experiments for the need of the phosphine ligand for thermal ruthenium-catalyzed *meta*-C–H glycosylation.

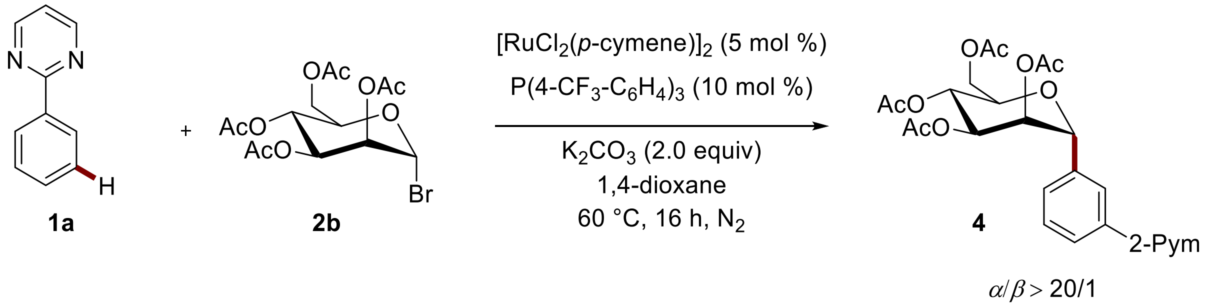

Entry	Additive	Base	Time [h]	Yield [%][a]
1	-	K_2CO_3	16	N.D.
2	$P(4\text{-}CF_3\text{-}C_6H_4)_3$ (10 mol %)	K_2CO_3	16	75[b]
3	-	NaOAc	16	N.D.
4	$P(4\text{-}CF_3\text{-}C_6H_4)_3$ (10 mol %)	NaOAc	16	N.D.

Reaction conditions: **1a** (0.1 mmol), **2b** (0.2 mmol), catalyst (5 mol %), base (0.2 mmol), solvent (1.0 mL), 60 °C, 16 h, under N_2. [a] Yield of isolated product. [b] As reported in the literature.[3] 2-Pym: 2-Phenylpyrimidine.

Kinetic Experiment

A stock solution of 7 mL was prepared in the glovebox. 2-phenylpyrimidine (**1a**) (1.092 g, 0.70 mmol), $[RuCl_2(p\text{-cymene})]_2$ (21.4 mg, 5.0 mol %), and the glycoside (**2b**) (574.0 mg, 2.0 equiv) were added to a 10 mL vial with a stirring bar. Then, 7 mL of dry 1,4-dioxane was added and stirred for 2 minutes until a homogeneous solution was formed. Subsequently, 1 mL was taken and transferred to a 10-mL vial containing a stirring bar. This step was repeated for six vials in total. Sodium acetate (16.4 mg, 0.2 mmol) was added to each vial individually. The vials were closed with a screw cap and wrapped with parafilm. The vials were taken out of the glovebox, the reaction mixture was stirred under visible light irradiation 2 × Kessil A360N and the temperature was maintained between 25–35 °C. After the indicated time, the solution was diluted with CH_2Cl_2, and concentrated in *vacuo*. The solution mixture was purified by column chromatography (*n*-hexane/EtOAc: 5/1 to 2/1).

Table 16. Kinetic experiment for photoinduced ruthenium-catalyzed *meta*-C–H glycosylation.

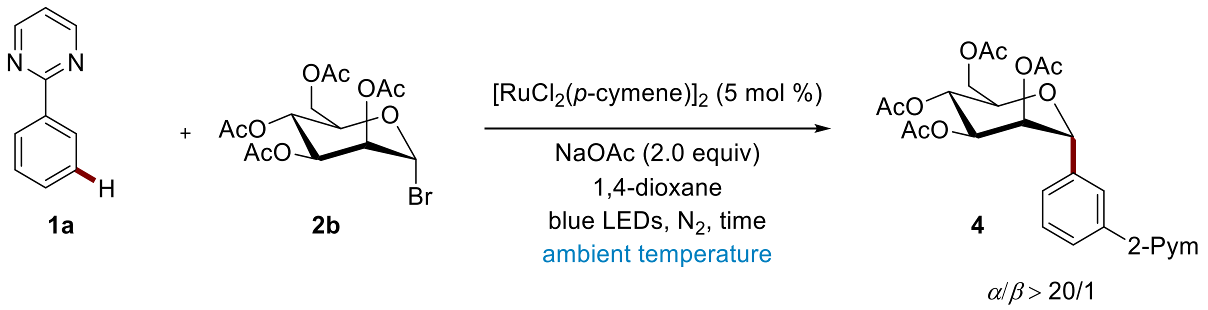

Entry	Time [h]	Yield [%][a]
1	8	13.70
2	16	20.16
3	24	30.85
4	32	34.35
5	40	38.67
6	48	54.00

Reaction conditions: **1a** (0.1 mmol), **2b** (0.2 mmol), catalyst (5 mol %), base (0.2 mmol), solvent (1.0 mL), 450 nm, 25–33 °C, 48 h, under N_2. [a] Yield of isolated product. 2-Pym: 2-Phenylpyrimidine.

Using the conversion data from Table 16, the reaction rate k_1 was determined based on the previous report to be 2.58591×10^{-10} mol/s.[4]

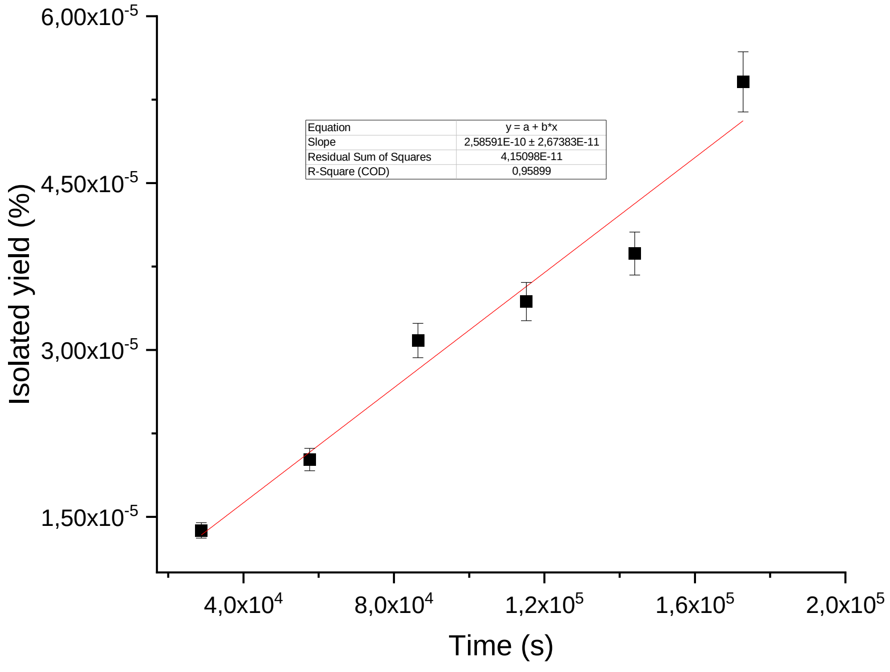

Figure S2. Determination of the average reaction rate k_1.

Determination of Quantum Yield

The quantum yield was assessed using a modified version of the literature procedure.[5]

Preparation of potassium ferrioxalate solution:

Potassium ferrioxalate (295 mg) and H_2SO_4 (140 μL) were diluted with water to a final volume of 50 mL.

Preparation of buffer solution:

NaOAc (2.478 g) and H_2SO_4 (0.5 mL) were diluted with water to a final volume of 50 mL.

Using the same setup as for the catalytic reactions, 2 mL of the potassium ferrioxalate solution were irradiated for 20 seconds.

The sample solution was added to 4 mL of a buffer solution containing 1,10-phenanthroline (2 mg). The mixture was then diluted with water to a final volume of 10 mL. Subsequently, the absorbance of this solution was determined at 510 nm. The same procedure was followed for an unirradiated sample.

Calculation Number of Photons:

Abs of Fe^{2+} (at 510 nm) = 4.73345 (after irradiation of 20 sec)

Abs of Fe^{2+} (at 510 nm) = 1.65721 (no irradiation)

Abs of Fe^{2+} (at 510 nm) = 4.73345−1.65721 = 3.07624

$$[\mathrm{Fe}^{2+}] = \frac{\text{Abs of Fe}^{2+}\text{(at 500 nm)}}{\varepsilon \times 1}$$

$$[\mathrm{Fe}^{2+}] = \frac{3.07624}{11100\ \mathrm{M^{-1}cm^{-1}} \times 1\ \mathrm{cm}} = 2.771 \times 10^{-4}\mathrm{M}$$

$$\mathrm{n}_{(\mathrm{Fe}^{2+})} = 2.771 \times 10^{-4}\ \mathrm{M} \times 0.010\ \mathrm{L} = 2.771 \times 10^{-6}\ \mathrm{mol}$$

With quantum yield of 0.805 for the absorption of Fe^{3+}:[5a]

$$\mathrm{n}_{(\text{ photons})} = 3.442 \times 10^{-6}\ \mathrm{mol}$$

$$\mathrm{n}_{(\text{ photons/s})} = 1.721 \times 10^{-7}\ \mathrm{mol/s}$$

The reaction rate k_1 was determined to be 2.58591×10^{-10} mol/s.

$$\text{Quantum Yield} = \frac{k_1}{\text{photons/s}} = \frac{2.58591 \times 10^{-10}\ \mathrm{mol/s}}{1.721 \times 10^{-7}\mathrm{mol/s}} = 0.00150$$

On/Off Experiment

A stock solution of 5 mL was prepared in the glovebox. 2-phenylpyrimidine (**1a**) (78.0 mg, 0.50 mmol), $[RuCl_2(p\text{-cymene})]_2$ (15.3 mg, 5.0 mol %), and the glycoside (**2b**) (410.0 mg, 2.0 equiv.) were added to a 10 mL vial with a stirring bar. Then, 5 mL of dry 1,4-dioxane was added and stirred for 2 minutes until a homogeneous solution was formed. Subsequently, 1 mL was taken and transferred to a 10-mL vial containing a stirring bar. This step was repeated for four vials in total. Sodium acetate (16.4 mg, 0.2 mmol) was added to each vial individually. The vials were closed with a screw cap and wrapped with parafilm. The vials were taken out of the glovebox, the reaction mixture was stirred under visible light irradiation 2 × Kessil A360N and the temperature was maintained between 25–35 °C. During the off reaction periods, the samples were shielded from light by wrapping them in aluminum foil and subsequently placed in a metal block maintained at 30 °C, where they were stirred for the specified duration. After the indicated time, the solution was diluted with CH_2Cl_2, and concentrated in *vacuo*. The solution mixture was purified by column chromatography (*n*-hexane/EtOAc: 5/1 to 2/1).

Table 17. On/Off experiment for photoinduced ruthenium-catalyzed *meta*-C–H glycosylation.

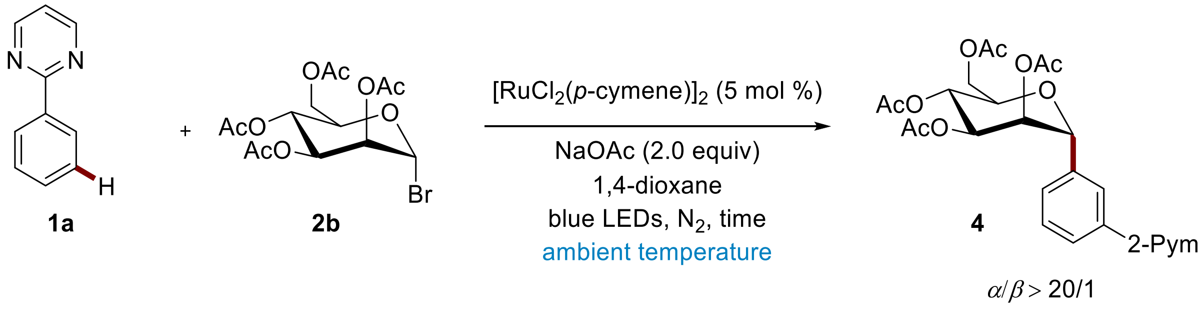

Entry	Light	Time [h]	Yield [%][a]
1	On	15	16.4
2	Off	28	16.9
3	On	40	17.3
4	Off	48	17.4

Reaction conditions: **1a** (0.1 mmol), **2b** (0.2 mmol), catalyst (5 mol %), base (0.2 mmol), solvent (1.0 mL), 450 nm, 25–33 °C, 48 h, under N_2. [a] Yield of isolated product. 2-Pym: 2-Phenylpyrimidine.

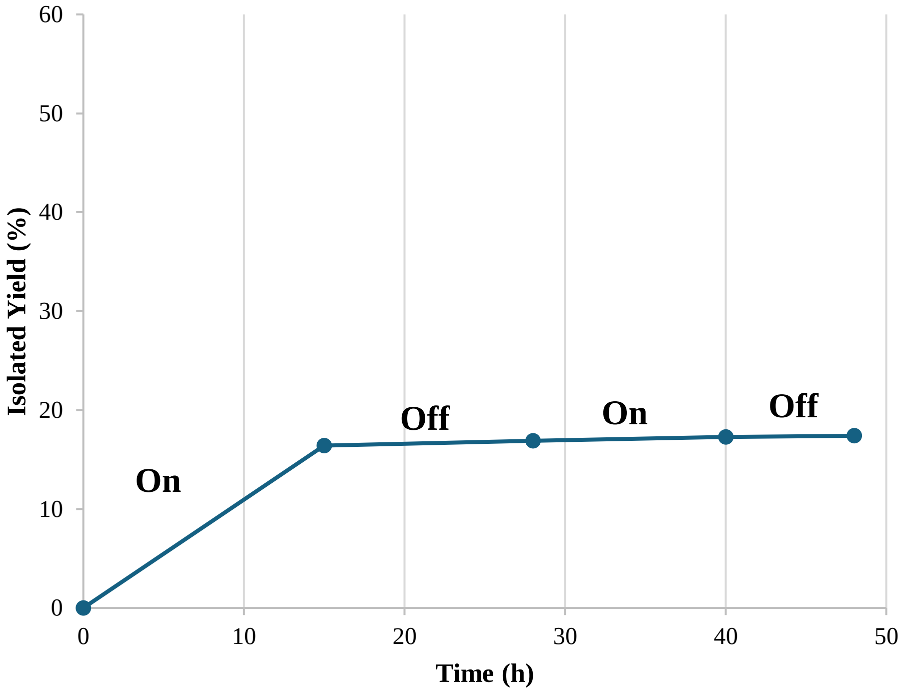

Figure S3. On/Off experiment.

Influence of *para*-cymene

Table 18. Influence of *para*-cymene for photoinduced ruthenium-catalyzed *meta*-C–H glycosylation.

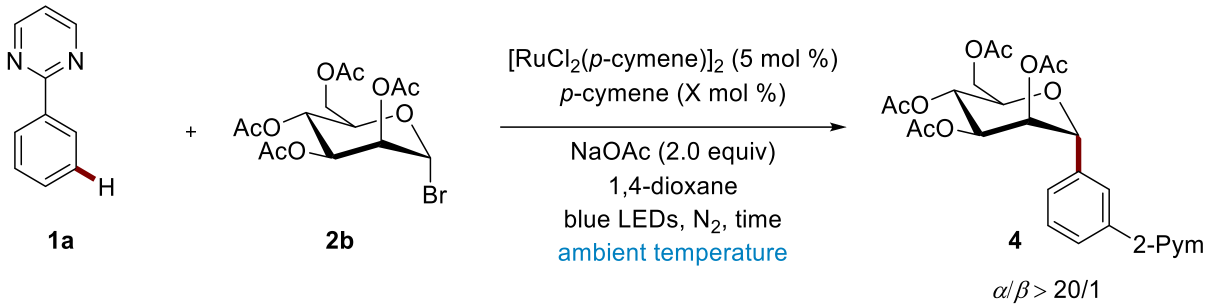

Entry	*p*-cymene [X mol %]	Yield [%][a]
1	5	16
2	45	11
3	60	24
4	127	24

Reaction conditions: **1a** (0.1 mmol), **2b** (0.2 mmol), catalyst (5 mol %), base (0.2 mmol), solvent (1.0 mL), 450 nm, 25–33 °C, 48 h, under N_2. [a] Yield of isolated product. 2-Pym: 2-Phenylpyrimidine

Reaction of a *para*-cymene-free Complex

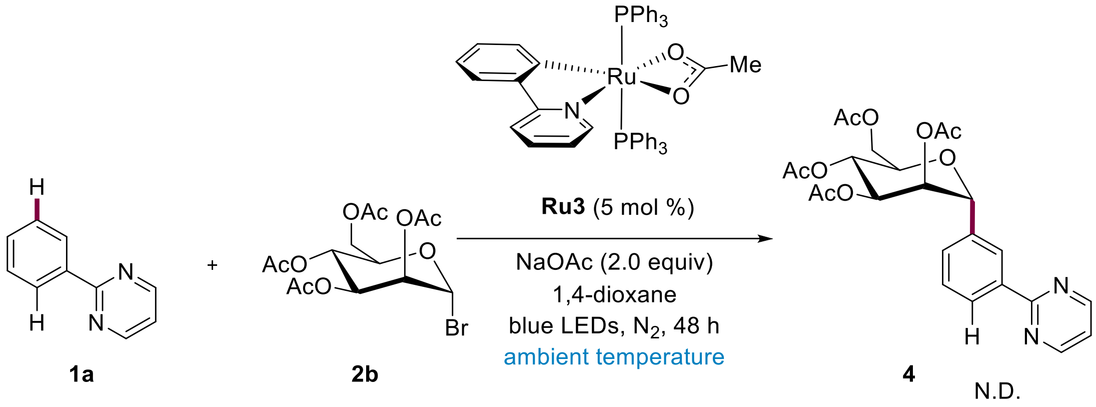

The complex was synthesized according to Korkit Korvorapun.[1c] 2-phenylpyrimidine (**1a**) (15.5 mg, 0.10 mmol), (2*R*,3*R*,4*S*,5*S*,6*R*)-2-(acetoxymethyl)-6-bromotetrahydro-2*H*-pyran-3,4,5-triyl-triacetate (**2b**) (82.0 mg, 0.20 mmol), were added to a 10 mL vial with a stirring bar and transferred to the glovebox. Then, NaOAc (16.4 mg, 0.2 mmol, 2.0 equiv), **Ru3** (5.0 mol %) and 1,4-dioxane (1.0 mL) were sequently added and the vial was closed with a plastic lid. Parafilm was then wrapped around and the mixture was stirred under visible light irradiation (using 2 × Kessil A360N). The reaction was carried out at ambient temperature (25–33 °C). After 48 hours no product was detected.

Computational Studies

Computation Methods

All DFT calculations were performed with Gaussian 16, Revision A.03 package.[6] The geometry optimizations were conducted at the PBE0[7] level of theory in combination with Grimme's D3 dispersion corrections with a Becke-Johnson damping scheme (D3BJ)[8] in the gas phase. All atoms were described with a def2-SVP basis set,[9] while ruthenium and bromine were also described with a SDD pseudopotential.[10] To confirm whether each optimized stationary point is an energy minimum or a transition state as well as evaluate the thermal and nonthermal corrections at 298.15 K, the vibrational frequencies were computed at the same level of theory as for the geometry optimizations. The single-point energies were further evaluated with PBE0[7] functional in combination with a standalone version of Grimme's D4 dispersion corrections,[11] with a def2-TZVP basis set in combination with a SDD pseudopotential for ruthenium.[10] Solvent effects were taken into account using the implicit solvation model SMD[12] with a dielectric constant of $\varepsilon = 2.21$, which corresponds to 1,4-dioxane. All reported energies are based on gas-phase Gibbs free energies with def2-SVP basis set for which the electronic energies were corrected by PBE0-D4 with a def2-TZVP basis set and solvent effects. The 3D structures of the optimized transition state geometries were constructed by using CYLview software.[13]

Given the unique features of the photoinduced ruthenium-catalyzed *meta*-selective-C–H glycosylation being operative in the absence of phosphines, computational studies were carried out to gain insight into the details of the reaction mechanism at the PBE0-D4/def2-TZVP-SDD-SMD(1,4-dioxane)//PBE0-D3(BJ)/def2-SVP level of theory (Figure S4). Under photoexcitation conditions, the η^6-*p*-cymene dissociated from the catalyst, forming the active species int1. The facile C–H activation of 2-phenylpyrimidine was computed to occured *via* a 6-center transition state **TS3** with a small barrier of 3.0 kcal mol^{-1}. The cycloruthenated complex **int4** then underwent ligand exchange (L.exc.) to form **int5**. Blue-light irradiation of the *in situ* formed ruthenium(II)-cyclometalated complex int5, followed by the intersystem crossing generates a long-lived excited state of the ruthenium(II)-complex **int5^{tri}**, which underwent the inner-sphere single electron transfer (ISET) with the mannosyl bromide and produced the *C*-centered radical along with the ruthenium(III)-cyclometalated complex **int7**. The computed energy barrier for the photoinduced single electron transfer (SET) was calculated to be 5.3 kcal mol^{-1}. Direct ISET conversion from **int4** to **int7** was determined to present a barrier of 16.9 kcal mol^{-1}, which is less favorable compared to the photochemical activity under blue-light irradiation. Subsequently, the mannosyl radical attacked the *para*-position to the Ru–C bond of **int7** *via* transition state **TS8^{oss}**, generating the ruthenium-carbene complex int9. The overall energy barrier of 19.7 kcal mol^{-1} for the radical attack (RA) step indicates it to be the rate-limiting step for the whole catalytic cycle. It's worth noting that, the coordination of the phosphine ligand changes the maximum absorption of the intermediate **int14** to 347.43 nm (Figure S5), which is located outside the visible light range. Furthermore, without the assistance of photo-irradiation after the dissociation of *para*-cymene, the potential SET process through the inner-sphere regime was proven to be facile with an energy barrier of 15.5 kcal mol^{-1}. However, the rate-determining step, radical attack, requires an insurmountable energy barrier of 30.1 kcal mol^{-1}. These findings are consistent with the observed experimental results that no desired product was observed when phosphine ligand was added to the reaction system (Table 14 and Table 15).

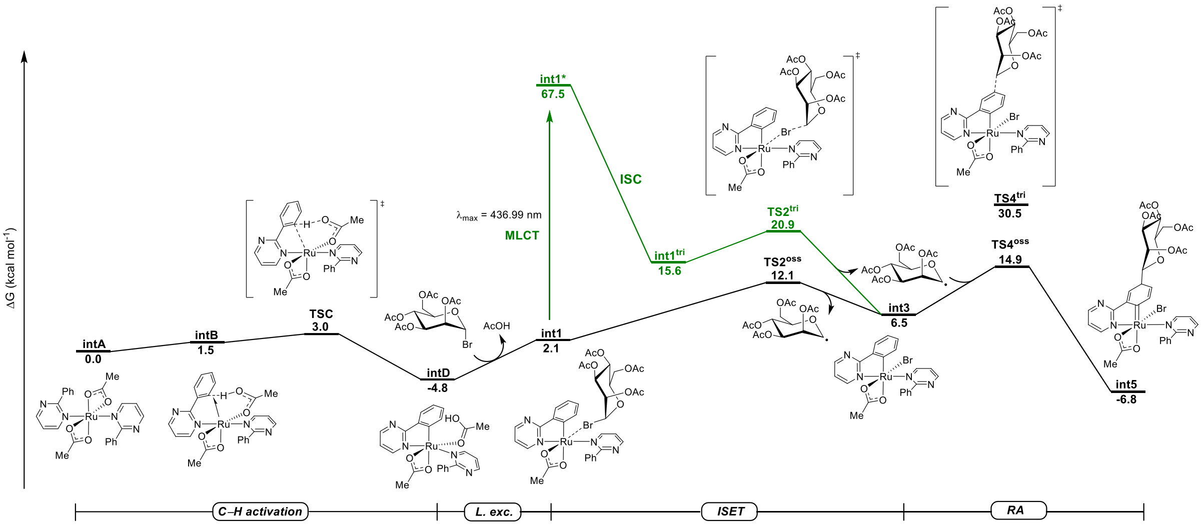

Figure S4. Computed relative Gibbs free energies ($\Delta G_{298.15}$) in kcal mol^{-1} for photoinduced ruthenium-catalyzed *meta*-C–H glycosylation at the PBE0-D4/def2-TZVP-SMD(1,4-dioxane)//PBE0-D3(BJ)/def2-SVP level of theory.

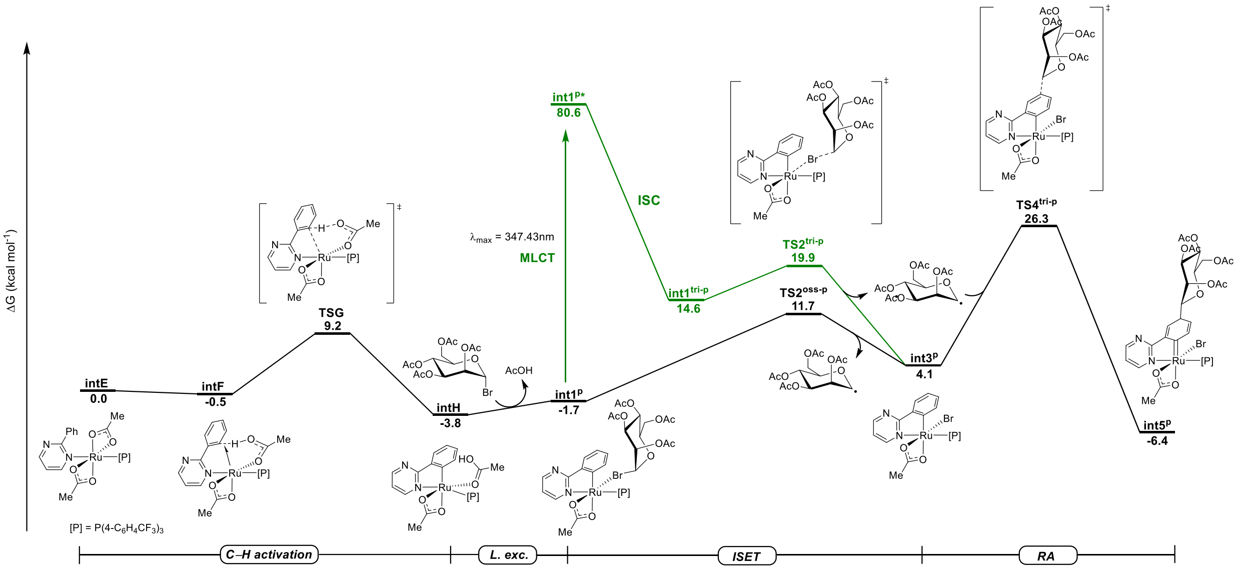

Figure S5. Computed relative Gibbs free energies ($\Delta G_{298.15}$) in kcal mol^{-1} for photoinduced ruthenium-catalyzed *meta*-C–H glycosylation using phosphine as ligand at the PBE0-D4/def2-TZVP-SMD(1,4-dioxane)//PBE0-D3(BJ)/def2-SVP level of theory.

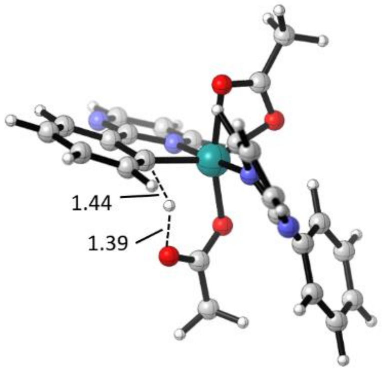

Figure S6. Computed transition state structure for C–H activation elementary step **TSB**. Key distances are provided in Å.

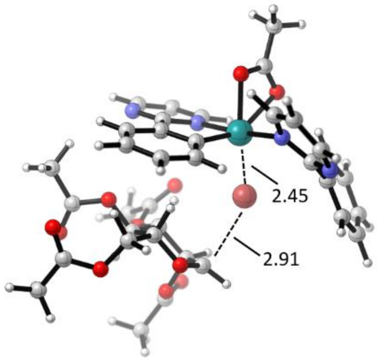

Figure S7. Computed transition state structure for ISET elementary step at the open-shell singlet state **TS2^{oss}**. Key distances are provided in Å.

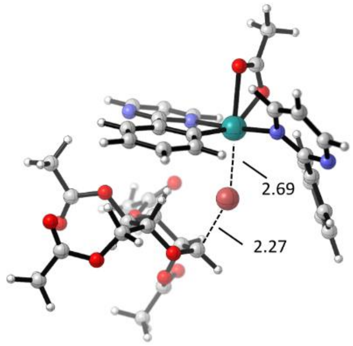

Figure S8. Computed transition state structure for ISET elementary step at the triplet state **TS2^{tri}**. Key distances are provided in Å.

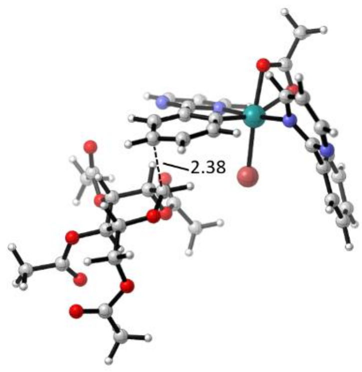

Figure S9. Computed transition state structure for radical attack elementary step at the open-shell singlet state **TS4^{oss}**. Key distances are provided in Å.

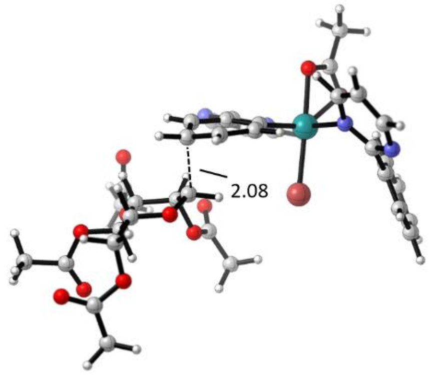

Figure S10. Computed transition state structure for radical attack elementary step at the triplet state **TS4^{tri}**. Key distances are provided in Å.

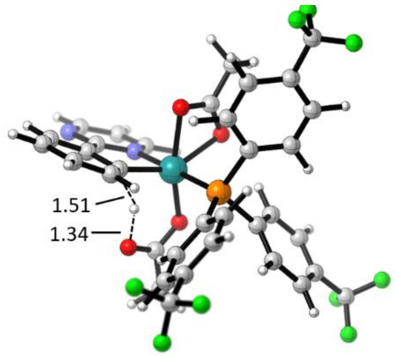

Figure S11. Computed transition state structure for C–H activation elementary step **TSG**. Key distances are provided in Å.

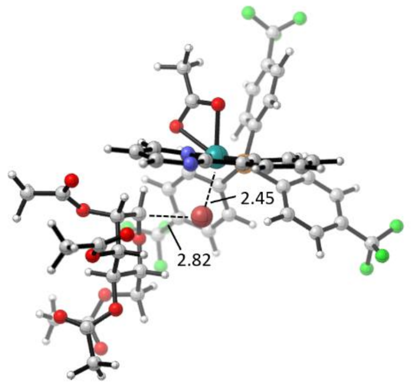

Figure S12. Computed transition state structure for ISET elementary step at the open-shell singlet state **TS2**$^{\mathbf{oss\text{-}p}}$. Key distances are provided in Å.

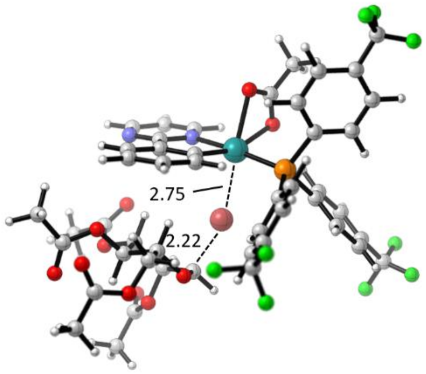

Figure S13. Computed transition state structure for ISET elementary step at the triplet state **TS2**$^{\mathbf{tri\text{-}p}}$. Key distances are provided in Å.

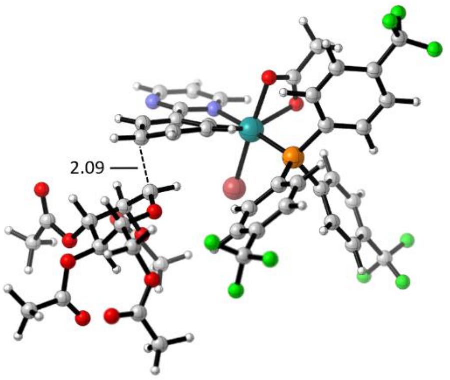

Figure S14. Computed transition state structure for radical attack elementary step at the triplet state **TS4**$^{\mathbf{tri\text{-}p}}$. Key distances are provided in Å.

Table 19. Calculated electronic energies at the PBE0-D4/def2-TZVP-SDD-SMD(1,4 dioxane) level of theory and Gibbs free energies with dispersion corrections for all structures (all in Hartree).

Structure	Electronic Energy	Total Gibbs Free Energy
**intA**	-1541.70700620	-1541.34364720
**intB**	-1541.70512259	-1541.34122559
**TSC**	-1541.69954125	-1541.33893125
**intD**	-1541.71583335	-1541.35123435
**int1**	-2547.25132632	-2546.64051032
**int1***	-2547.14705866	-2546.53624266
**int1^{tri}**	-2547.22444819	-2546.61897019
**TS2^{oss}**	-2547.23182049	-2546.62458649
**TS2^{tri}**	-2547.21654820	-2546.61053020
**int3**	-1326.07723383	-1325.77301183
**TS4^{oss}**	-2547.21382577	-2546.62011677
**TS4^{tri}**	-2547.19957904	-2546.59522804
**int5**	-2547.25127975	-2546.65467375
**intE**	-3093.01880097	-3092.55094397
**intF**	-3093.02101410	-3092.55175410
**TSG**	-3093.01236919	-3092.54741219
**intH**	-3093.02441299	-3092.55695799
**int1^{p}**	-4098.56748457	-4097.85376957
**int1^{p*}**	-4098.43633781	-4097.72262281
**int2$^{tri\text{-}p}$**	-4098.53769136	-4097.82783936
**TS2$^{oss\text{-}p}$**	-4098.54316185	-4097.83239985
**TS2$^{tri\text{-}p}$**	-4098.52801955	-4097.81945155
**int3^{p}**	-2877.39242723	-2876.98409923
**TS4$^{tri\text{-}p}$**	-4098.51987175	-4097.80926575
**int5^{p}**	-4098.57746415	-4097.86138315
**mannosyl bromide**	-1505.798118	-1505.394853
**mannosyl radical**	-1505.814449	-1505.407638
**AcOH**	-228.9398898	-228.9090408

Cartesian Coordinates of the Optimized Structure

intA
Lowest frequency = 23.1434 cm^{-1}
Charge = 0, Multiplicity = 1

55

Ru	-0.000037000	0.000045000	-0.000027000
O	1.270967000	1.018386000	-1.372218000
C	0.952873000	0.222463000	-2.299484000
O	0.109216000	-0.679199000	-2.023523000
C	-0.952980000	-0.222480000	2.299392000
O	-1.270990000	-1.018402000	1.372089000
O	-0.109321000	0.679188000	2.023525000
C	-1.588664000	-0.315062000	3.648557000
H	-0.984183000	0.208165000	4.399101000
H	-2.582999000	0.155676000	3.599760000
H	-1.733566000	-1.367035000	3.926941000
C	1.588635000	0.314711000	-3.648632000
H	0.982330000	-0.205724000	-4.399666000
H	1.736836000	1.366521000	-3.925823000
H	2.581346000	-0.159530000	-3.600667000
N	1.429984000	-1.447128000	0.355036000
C	2.781069000	-1.321548000	0.309605000
C	0.926686000	-2.695944000	0.371679000
C	3.419726000	0.005603000	0.412741000
N	3.604046000	-2.361599000	0.162052000
C	1.732928000	-3.816390000	0.277004000
H	-0.161100000	-2.759771000	0.445976000
C	4.621152000	0.225620000	-0.273932000
C	2.887905000	1.023399000	1.213697000
C	3.098350000	-3.583784000	0.131067000
H	1.306704000	-4.819897000	0.285500000
C	5.258775000	1.458943000	-0.198235000
H	5.041266000	-0.587040000	-0.868951000
C	3.540288000	2.249635000	1.299985000
H	1.965603000	0.857030000	1.771153000
H	3.806056000	-4.408921000	-0.009805000
C	4.717147000	2.476316000	0.587478000
H	6.185459000	1.626971000	-0.752124000
H	3.121714000	3.035938000	1.932764000
H	5.219441000	3.444938000	0.651281000
N	-1.429968000	1.447176000	-0.355192000
C	-2.781054000	1.321632000	-0.309979000
C	-0.926643000	2.695987000	-0.372058000
C	-3.419684000	-0.005574000	-0.412552000
N	-3.604072000	2.361741000	-0.163035000
C	-1.732897000	3.816467000	-0.277932000
H	0.161159000	2.759793000	-0.446096000
C	-2.887914000	-1.023672000	-1.213156000
C	-4.621022000	-0.225353000	0.274349000
C	-3.098375000	3.583932000	-0.132380000
H	-1.306656000	4.819966000	-0.286580000
C	-3.540261000	-2.249964000	-1.298869000

H	-1.965667000	-0.857488000	-1.770760000
C	-5.258607000	-1.458733000	0.199229000
H	-5.041107000	0.587538000	0.869077000
H	-3.806114000	4.409126000	0.007991000
C	-4.717029000	-2.476401000	-0.586131000
H	-3.121727000	-3.036517000	-1.931365000
H	-6.185223000	-1.626570000	0.753290000
H	-5.219290000	-3.445069000	-0.649481000

intB

Lowest frequency = 31.6191 cm^{-1}

Charge = 0, Multiplicity = 1

55

Ru	0.378580000	-0.141292000	0.344593000
O	-0.605102000	-1.290095000	-0.996492000
C	-1.007151000	-0.797343000	-2.121018000
O	-0.803771000	0.359815000	-2.479294000
C	0.689610000	-0.453452000	2.811389000
O	1.243856000	0.508836000	2.209555000
O	-0.044511000	-1.232951000	2.132787000
C	0.922178000	-0.694722000	4.268035000
H	0.038528000	-1.158299000	4.724469000
H	1.768998000	-1.389664000	4.379899000
H	1.178677000	0.243607000	4.774895000
C	-1.763145000	-1.779990000	-2.980440000
H	-1.124931000	-2.648805000	-3.198314000
H	-2.639550000	-2.146912000	-2.427793000
H	-2.079464000	-1.302695000	-3.915023000
N	-1.188775000	1.261187000	0.701650000
C	-2.477825000	1.246428000	0.278529000
C	-0.730997000	2.420319000	1.207841000
C	-3.186562000	-0.015925000	-0.003431000
N	-3.207690000	2.355113000	0.145367000
C	-1.474652000	3.588060000	1.185818000
H	0.282889000	2.388125000	1.611496000
C	-4.247310000	-0.002337000	-0.920527000
C	-2.898782000	-1.190960000	0.700640000
C	-2.714150000	3.509741000	0.557440000
H	-1.077461000	4.517158000	1.595042000
C	-4.997735000	-1.151135000	-1.142399000
H	-4.467837000	0.924243000	-1.451605000
C	-3.662736000	-2.333986000	0.485345000
H	-2.079932000	-1.210470000	1.420754000
H	-3.332991000	4.399978000	0.395637000
C	-4.708751000	-2.319985000	-0.436322000
H	-5.813427000	-1.135420000	-1.868841000
H	-3.434400000	-3.244903000	1.043048000
H	-5.302266000	-3.222044000	-0.605211000
N	1.956088000	-1.325506000	-0.019552000
C	3.059117000	-0.741713000	-0.556607000
C	1.982981000	-2.638886000	0.230854000
C	2.901924000	0.671452000	-0.863613000
N	4.193703000	-1.392223000	-0.791805000
C	3.127721000	-3.381633000	-0.016898000
H	1.067211000	-3.063399000	0.648417000

C	1.591615000	1.213423000	-0.775672000
C	3.996593000	1.479157000	-1.183217000
C	4.230345000	-2.689864000	-0.521311000
H	3.160583000	-4.452705000	0.184170000
C	1.427770000	2.575188000	-1.081447000
H	0.587641000	0.631262000	-1.173262000
C	3.813338000	2.833671000	-1.431330000
H	4.985985000	1.018443000	-1.220235000
H	5.176296000	-3.207241000	-0.716989000
C	2.524755000	3.375071000	-1.382594000
H	0.421893000	2.996028000	-1.117458000
H	4.667966000	3.468111000	-1.675436000
H	2.372117000	4.434164000	-1.606184000

TSC
Lowest frequency = -975.4578 cm^{-1}
Charge = 0, Multiplicity = 1

55

Ru	0.355885000	-0.130998000	0.356746000
O	-0.599156000	-1.335285000	-0.999802000
C	-0.799847000	-0.860896000	-2.158845000
O	-0.387499000	0.274317000	-2.484608000
C	0.667418000	-0.390104000	2.843804000
O	1.187500000	0.563599000	2.190974000
O	-0.056986000	-1.217908000	2.224196000
C	0.946696000	-0.548295000	4.305297000
H	0.106638000	-1.048822000	4.802816000
H	1.842495000	-1.177183000	4.426471000
H	1.150727000	0.426654000	4.765181000
C	-1.580565000	-1.700878000	-3.122413000
H	-1.272226000	-2.751401000	-3.042926000
H	-2.642879000	-1.645872000	-2.839903000
H	-1.456683000	-1.332571000	-4.147127000
N	-1.232446000	1.244811000	0.695156000
C	-2.527630000	1.196001000	0.293890000
C	-0.806197000	2.412136000	1.213732000
C	-3.186163000	-0.086467000	-0.022902000
N	-3.302117000	2.277481000	0.197856000
C	-1.593732000	3.550930000	1.229698000
H	0.215832000	2.409617000	1.596431000
C	-4.228350000	-0.096829000	-0.961052000
C	-2.868225000	-1.261590000	0.668079000
C	-2.843736000	3.440979000	0.628487000
H	-1.220860000	4.485950000	1.648330000
C	-4.920723000	-1.273105000	-1.227890000
H	-4.483728000	0.833212000	-1.470937000
C	-3.574917000	-2.431691000	0.408374000
H	-2.067051000	-1.260433000	1.409278000
H	-3.499537000	4.310176000	0.502755000
C	-4.594568000	-2.444354000	-0.542179000
H	-5.722760000	-1.275642000	-1.969782000
H	-3.321919000	-3.343279000	0.954178000
H	-5.142028000	-3.367967000	-0.746263000
N	1.970433000	-1.285920000	0.039143000
C	3.054855000	-0.677055000	-0.513719000

C	2.051730000	-2.583009000	0.350638000
C	2.836225000	0.713414000	-0.867651000
N	4.214589000	-1.295365000	-0.720774000
C	3.225342000	-3.291127000	0.137522000
H	1.154775000	-3.025250000	0.790782000
C	1.502672000	1.195717000	-0.730534000
C	3.886496000	1.535325000	-1.290921000
C	4.299594000	-2.577964000	-0.398074000
H	3.301060000	-4.349478000	0.388073000
C	1.290382000	2.542492000	-1.088495000
H	0.368770000	0.553323000	-1.351393000
C	3.643008000	2.866722000	-1.597488000
H	4.887329000	1.103595000	-1.362261000
H	5.264885000	-3.066713000	-0.573326000
C	2.338137000	3.361533000	-1.496988000
H	0.277087000	2.949513000	-1.081156000
H	4.457108000	3.517395000	-1.923746000
H	2.132529000	4.402931000	-1.760258000

intD

Lowest frequency = 27.5479 cm^{-1}

Charge = 0, Multiplicity = 1

55

Ru	0.377141000	-0.163698000	0.466860000
O	-0.399567000	-0.912133000	-1.324976000
C	-0.668236000	-0.381885000	-2.399311000
O	-0.412230000	0.869947000	-2.668676000
C	0.558957000	-1.015702000	2.884130000
O	1.029688000	0.077166000	2.430085000
O	-0.080436000	-1.780104000	2.124087000
C	0.822424000	-1.378181000	4.316424000
H	0.014797000	-2.012353000	4.703066000
H	1.762373000	-1.949641000	4.369000000
H	0.939811000	-0.476001000	4.929893000
C	-1.315918000	-1.140694000	-3.506190000
H	-0.597368000	-1.263091000	-4.330928000
H	-1.648305000	-2.118200000	-3.142567000
H	-2.170794000	-0.568593000	-3.891264000
N	-1.281502000	0.994993000	1.004582000
C	-2.559364000	0.940387000	0.548205000
C	-0.977419000	2.030620000	1.813704000
C	-3.066430000	-0.271574000	-0.128164000
N	-3.434027000	1.935241000	0.689090000
C	-1.870562000	3.056895000	2.070204000
H	0.031992000	2.016340000	2.228685000
C	-4.020089000	-0.132875000	-1.146121000
C	-2.681486000	-1.551900000	0.288620000
C	-3.096809000	2.990513000	1.414718000
H	-1.596270000	3.888958000	2.719349000
C	-4.554627000	-1.258356000	-1.765150000
H	-4.335147000	0.870922000	-1.436458000
C	-3.233969000	-2.674070000	-0.322394000
H	-1.947109000	-1.671670000	1.089061000
H	-3.831970000	3.799996000	1.486356000
C	-4.162511000	-2.532948000	-1.353318000

H	-5.288161000	-1.141537000	-2.566562000
H	-2.932734000	-3.668997000	0.013217000
H	-4.589094000	-3.417939000	-1.832342000
N	2.102145000	-1.112711000	-0.000850000
C	3.059914000	-0.352661000	-0.605624000
C	2.375058000	-2.397503000	0.247387000
C	2.659630000	1.025824000	-0.821437000
N	4.250625000	-0.822122000	-0.967433000
C	3.599309000	-2.949258000	-0.104989000
H	1.584021000	-2.961416000	0.749346000
C	1.337997000	1.347108000	-0.377083000
C	3.514323000	1.958980000	-1.414608000
C	4.516697000	-2.098045000	-0.721926000
H	3.825979000	-3.996742000	0.095735000
C	0.944603000	2.689624000	-0.598935000
H	0.100910000	1.262445000	-1.916277000
C	3.084934000	3.267310000	-1.605118000
H	4.511492000	1.632784000	-1.720523000
H	5.504236000	-2.462966000	-1.027070000
C	1.796234000	3.621028000	-1.195964000
H	-0.054462000	3.026012000	-0.308459000
H	3.741177000	4.005875000	-2.070185000
H	1.444119000	4.645868000	-1.347149000

int1

Lowest frequency = 14.8960cm^{-1}

Charge = 0, Multiplicity = 1

90

Ru	1.984090000	-0.591938000	0.590855000
C	3.533511000	-0.984049000	2.633016000
O	3.023842000	-1.849868000	1.842513000
O	3.326619000	0.230243000	2.440752000
C	4.336033000	-1.476966000	3.801290000
H	5.070238000	-0.719621000	4.102669000
H	3.652986000	-1.651768000	4.647201000
H	4.829042000	-2.427761000	3.562307000
N	3.658480000	-0.761256000	-0.692907000
C	4.308283000	0.155143000	-1.455719000
C	4.050235000	-2.043530000	-0.833375000
C	4.135761000	1.602118000	-1.215478000
N	5.143498000	-0.169635000	-2.442274000
C	4.976010000	-2.438059000	-1.784104000
H	3.570795000	-2.757823000	-0.163595000
C	4.177721000	2.480498000	-2.307087000
C	3.997088000	2.116107000	0.080588000
C	5.458668000	-1.441935000	-2.626772000
H	5.266878000	-3.484222000	-1.882106000
C	4.027734000	3.847797000	-2.110504000
H	4.317084000	2.068002000	-3.307462000
C	3.867302000	3.489979000	0.271363000
H	3.989296000	1.446296000	0.943370000
H	6.127253000	-1.671370000	-3.464391000
C	3.867598000	4.355950000	-0.819747000
H	4.038639000	4.524332000	-2.968412000
H	3.758258000	3.880526000	1.285518000

H	3.751214000	5.431665000	-0.665997000
N	0.469122000	-0.829871000	1.925568000
C	-0.264900000	-1.967931000	1.739992000
C	0.275401000	-0.119761000	3.039558000
C	-0.010190000	-2.623257000	0.467695000
N	-1.160084000	-2.411242000	2.616560000
C	-0.645406000	-0.531599000	3.994837000
H	0.877265000	0.783896000	3.148693000
C	0.984925000	-1.999908000	-0.339941000
C	-0.755424000	-3.724271000	0.028069000
C	-1.343458000	-1.705843000	3.727407000
H	-0.813481000	0.054226000	4.898089000
C	1.124128000	-2.502010000	-1.650719000
C	-0.553185000	-4.222356000	-1.252418000
H	-1.499915000	-4.160163000	0.697932000
H	-2.083768000	-2.092255000	4.437623000
C	0.376512000	-3.592325000	-2.091480000
H	1.822659000	-2.025093000	-2.343292000
H	-1.130892000	-5.076731000	-1.611267000
H	0.513843000	-3.960320000	-3.112918000
Br	1.065928000	1.369120000	-0.782821000
C	-0.867772000	1.721264000	-1.573851000
O	-1.391600000	0.695763000	-2.255147000
C	-1.709550000	2.238514000	-0.424554000
H	-0.585595000	2.517347000	-2.272569000
C	-1.837921000	-0.422447000	-1.490448000
C	-2.160835000	1.089747000	0.465977000
H	-1.169103000	2.996269000	0.158440000
O	-2.858089000	2.820293000	-1.040645000
C	-2.440581000	-1.438309000	-2.428379000
C	-2.801793000	0.021640000	-0.392567000
H	-0.951906000	-0.886708000	-1.028184000
O	-3.124134000	1.537997000	1.399303000
H	-1.283181000	0.687003000	0.990841000
C	-3.439609000	3.870779000	-0.428181000
H	-1.798994000	-1.520490000	-3.316616000
H	-2.490181000	-2.414941000	-1.931649000
O	-3.740413000	-1.043994000	-2.846518000
O	-3.092168000	-1.116136000	0.390037000
H	-3.727928000	0.416906000	-0.832080000
C	-2.661068000	2.167560000	2.499678000
O	-2.993387000	4.396723000	0.555728000
C	-4.706103000	4.250798000	-1.128540000
C	-4.763532000	-1.868379000	-2.553056000
C	-4.386316000	-1.408622000	0.645049000
O	-1.490266000	2.273488000	2.748399000
C	-3.786983000	2.720980000	3.310906000
H	-5.476520000	3.506080000	-0.874600000
H	-4.572080000	4.228240000	-2.217981000
H	-5.032992000	5.240747000	-0.792035000
O	-4.623582000	-2.944355000	-2.030835000
C	-6.077872000	-1.249321000	-2.906364000
O	-5.300172000	-0.707865000	0.298389000
C	-4.496678000	-2.723083000	1.343485000
H	-3.456799000	2.882346000	4.343250000
H	-4.666221000	2.065596000	3.268600000
H	-4.056714000	3.691946000	2.866820000
H	-6.805964000	-2.035603000	-3.137804000

H	-6.421480000	-0.709690000	-2.009428000
H	-5.980887000	-0.537792000	-3.735178000
H	-4.572183000	-3.486377000	0.552222000
H	-3.599797000	-2.929897000	1.942334000
H	-5.412987000	-2.750514000	1.944623000

int1^{tri}
Lowest frequency = 11.1673 cm^{-1}
Charge = 0, Multiplicity = 1

90

Ru	2.211059000	-0.986779000	0.215704000
C	4.322665000	-2.074207000	1.480898000
O	3.981460000	-2.710621000	0.465962000
O	3.686235000	-1.033670000	1.852862000
C	5.505605000	-2.504472000	2.307408000
H	6.282537000	-1.726775000	2.259205000
H	5.208392000	-2.596397000	3.361918000
H	5.910921000	-3.455826000	1.943383000
N	3.613935000	-0.044853000	-1.021256000
C	3.986249000	1.260552000	-1.034462000
C	4.270537000	-0.885121000	-1.843782000
C	3.288720000	2.240266000	-0.178934000
N	4.929014000	1.742345000	-1.846927000
C	5.273657000	-0.449773000	-2.690281000
H	3.965813000	-1.932226000	-1.792949000
C	3.161919000	3.553575000	-0.657200000
C	2.713770000	1.906245000	1.055362000
C	5.561970000	0.913164000	-2.659285000
H	5.794449000	-1.144505000	-3.349544000
C	2.413551000	4.490546000	0.043768000
H	3.635625000	3.809972000	-1.605703000
C	1.964887000	2.849806000	1.755383000
H	2.902646000	0.932394000	1.514857000
H	6.327794000	1.348213000	-3.311766000
C	1.794372000	4.134386000	1.243015000
H	2.299897000	5.501086000	-0.355628000
H	1.503660000	2.566280000	2.701474000
H	1.182961000	4.857226000	1.788642000
N	0.923572000	-2.037113000	1.390224000
C	-0.049598000	-2.750630000	0.756619000
C	1.014831000	-2.128143000	2.721998000
C	-0.052074000	-2.576986000	-0.687275000
N	-0.919479000	-3.528963000	1.392998000
C	0.136477000	-2.923169000	3.443340000
H	1.822314000	-1.553664000	3.180599000
C	0.953864000	-1.708274000	-1.189257000
C	-0.980269000	-3.212482000	-1.520592000
C	-0.831589000	-3.612288000	2.714570000
H	0.209129000	-3.002647000	4.528315000
C	0.973223000	-1.510720000	-2.582124000
C	-0.934442000	-2.982814000	-2.889683000
H	-1.729970000	-3.872651000	-1.080323000
H	-1.562561000	-4.256293000	3.217132000
C	0.042062000	-2.126572000	-3.416082000
H	1.718896000	-0.846968000	-3.028451000

H	-1.653710000	-3.469750000	-3.552568000
H	0.074817000	-1.942742000	-4.493835000
Br	0.423346000	1.850604000	-1.882859000
C	-1.438679000	2.379110000	-1.295860000
O	-2.382807000	1.686382000	-1.976198000
C	-1.491483000	2.220329000	0.218716000
H	-1.508779000	3.435670000	-1.576506000
C	-2.457796000	0.298279000	-1.674483000
C	-1.682404000	0.758483000	0.628851000
H	-0.591566000	2.641559000	0.687975000
O	-2.642427000	2.956085000	0.628872000
C	-3.465411000	-0.341084000	-2.593222000
C	-2.800465000	0.155347000	-0.194965000
H	-1.474578000	-0.165575000	-1.883620000
O	-2.074612000	0.689474000	1.988708000
H	-0.737700000	0.211094000	0.476654000
C	-2.604808000	3.558956000	1.830929000
H	-3.248602000	-0.029465000	-3.625280000
H	-3.392334000	-1.433861000	-2.519639000
O	-4.781923000	0.079053000	-2.267749000
O	-2.970431000	-1.208071000	0.124602000
H	-3.734837000	0.695832000	0.012536000
C	-1.111108000	0.651699000	2.925289000
O	-1.626944000	3.590758000	2.531540000
C	-3.939971000	4.144859000	2.166641000
C	-5.686988000	-0.868275000	-1.964431000
C	-4.118234000	-1.572240000	0.742090000
O	0.061089000	0.598275000	2.663800000
C	-1.696391000	0.716225000	4.298833000
H	-4.622042000	3.319731000	2.424396000
H	-4.367022000	4.663728000	1.298275000
H	-3.844615000	4.822542000	3.021982000
O	-5.457513000	-2.049592000	-2.020211000
C	-6.973248000	-0.260762000	-1.504552000
O	-4.955790000	-0.780649000	1.089199000
C	-4.212645000	-3.055867000	0.862458000
H	-0.988373000	0.297859000	5.023172000
H	-2.664193000	0.200911000	4.342418000
H	-1.858582000	1.780248000	4.532634000
H	-7.799908000	-0.953396000	-1.700763000
H	-6.883530000	-0.117615000	-0.415940000
H	-7.148373000	0.714505000	-1.974688000
H	-4.641491000	-3.416984000	-0.086092000
H	-3.218941000	-3.505366000	0.993444000
H	-4.896383000	-3.315988000	1.678841000

TS2^{oss}
Lowest frequency = -43.7879 cm^{-1}
Charge = 0, Multiplicity = 1

90

Ru	-2.356470000	-0.216012000	-0.405789000
C	-4.351550000	-0.287494000	-2.047984000
O	-3.863997000	-1.277606000	-1.409819000
O	-3.852826000	0.846651000	-1.877522000
C	-5.476126000	-0.522151000	-3.009191000

H	-6.069052000	0.392673000	-3.130428000
H	-5.050473000	-0.791127000	-3.988467000
H	-6.105420000	-1.356206000	-2.673875000
N	-3.718220000	-0.069021000	1.224814000
C	-3.902372000	0.931022000	2.124958000
C	-4.352622000	-1.230454000	1.470770000
C	-3.505629000	2.315204000	1.817923000
N	-4.477856000	0.738112000	3.312865000
C	-5.056634000	-1.459126000	2.640579000
H	-4.255147000	-1.995115000	0.698071000
C	-3.037668000	3.134081000	2.853422000
C	-3.660286000	2.843301000	0.531001000
C	-5.021838000	-0.436344000	3.584383000
H	-5.558162000	-2.410385000	2.819357000
C	-2.678338000	4.450505000	2.592160000
H	-2.946534000	2.715369000	3.856713000
C	-3.323198000	4.169571000	0.281736000
H	-4.038370000	2.218291000	-0.279731000
H	-5.456319000	-0.562625000	4.582842000
C	-2.818534000	4.970272000	1.304524000
H	-2.288187000	5.077686000	3.397092000
H	-3.444866000	4.574436000	-0.725245000
H	-2.537881000	6.006533000	1.100417000
N	-1.154568000	-0.596776000	-1.991716000
C	-0.537451000	-1.807856000	-1.973718000
C	-0.984235000	0.196894000	-3.050343000
C	-0.701469000	-2.521856000	-0.714876000
N	0.188754000	-2.275571000	-2.980942000
C	-0.229970000	-0.228536000	-4.135072000
H	-1.470490000	1.173419000	-3.006331000
C	-1.445116000	-1.830031000	0.275492000
C	-0.108613000	-3.762174000	-0.462201000
C	0.331966000	-1.498559000	-4.049998000
H	-0.067770000	0.421382000	-4.993753000
C	-1.488836000	-2.396902000	1.560376000
C	-0.215351000	-4.324471000	0.804334000
H	0.445692000	-4.258975000	-1.260066000
H	0.932907000	-1.902098000	-4.872641000
C	-0.886368000	-3.627944000	1.816497000
H	-1.988010000	-1.870024000	2.376123000
H	0.254146000	-5.286999000	1.017667000
H	-0.934252000	-4.052913000	2.823062000
Br	-0.699958000	1.412617000	0.388159000
C	1.937962000	1.378183000	1.605512000
O	2.094362000	0.168137000	2.158441000
C	2.628759000	1.692950000	0.329545000
H	1.748448000	2.165650000	2.336701000
C	2.001999000	-0.949517000	1.287151000
C	2.542855000	0.522960000	-0.647435000
H	2.213692000	2.601447000	-0.129280000
O	4.028073000	1.919358000	0.626205000
C	2.325238000	-2.200919000	2.065715000
C	2.902453000	-0.757946000	0.069726000
H	0.952145000	-1.023429000	0.948206000
O	3.463640000	0.698336000	-1.708576000
H	1.516579000	0.475836000	-1.039142000
C	4.696575000	2.796022000	-0.135583000
H	1.828547000	-2.143526000	3.044628000

H	1.957295000	-3.079654000	1.523745000
O	3.723777000	-2.330253000	2.282420000
O	2.697849000	-1.859844000	-0.794414000
H	3.956382000	-0.721046000	0.379345000
C	3.027185000	1.315094000	-2.823428000
O	4.188060000	3.468246000	-0.995987000
C	6.153497000	2.799251000	0.216976000
C	4.329313000	-3.424974000	1.788950000
C	3.779721000	-2.510567000	-1.269813000
O	1.876773000	1.606061000	-3.014298000
C	4.168042000	1.599178000	-3.747746000
H	6.609522000	1.886386000	-0.196682000
H	6.291423000	2.772077000	1.305893000
H	6.639689000	3.679004000	-0.219207000
O	3.740443000	-4.326687000	1.247760000
C	5.813146000	-3.344005000	1.952462000
O	4.910875000	-2.185472000	-1.021288000
C	3.367236000	-3.695497000	-2.080419000
H	3.793384000	1.774413000	-4.762483000
H	4.903971000	0.784867000	-3.727593000
H	4.657997000	2.514491000	-3.380009000
H	6.235266000	-4.354421000	2.003807000
H	6.201577000	-2.846897000	1.049312000
H	6.090475000	-2.748838000	2.830867000
H	3.218094000	-4.524040000	-1.369660000
H	2.419434000	-3.506162000	-2.602260000
H	4.171503000	-3.967980000	-2.773444000

TS2^{tri}

Lowest frequency = -166.6887 cm^{-1}

Charge = 0, Multiplicity = 1

90

Ru	-2.353325000	-0.540312000	-0.562092000
C	-4.099289000	-1.005912000	-2.489255000
O	-3.769156000	-1.974319000	-1.766676000
O	-3.614453000	0.146654000	-2.268987000
C	-5.041929000	-1.190348000	-3.644301000
H	-5.776058000	-0.373310000	-3.665186000
H	-4.467618000	-1.139347000	-4.582021000
H	-5.545525000	-2.162441000	-3.585747000
N	-3.912673000	-0.505250000	0.854026000
C	-4.321323000	0.539701000	1.616709000
C	-4.479562000	-1.702061000	1.094186000
C	-3.874764000	1.904496000	1.290693000
N	-5.122253000	0.404271000	2.673433000
C	-5.374776000	-1.893507000	2.132353000
H	-4.176104000	-2.509547000	0.425069000
C	-3.640324000	2.811061000	2.332581000
C	-3.702912000	2.314991000	-0.037682000
C	-5.627614000	-0.788904000	2.942600000
H	-5.821706000	-2.870685000	2.316649000
C	-3.180139000	4.091162000	2.051011000
H	-3.803232000	2.484178000	3.360661000
C	-3.258466000	3.605420000	-0.313204000
H	-3.936412000	1.636746000	-0.862149000

H	-6.264293000	-0.867416000	3.831361000
C	-2.980380000	4.488440000	0.727306000
H	-2.972976000	4.785911000	2.868348000
H	-3.123753000	3.917178000	-1.351171000
H	-2.615819000	5.495025000	0.508093000
N	-0.868381000	-0.753153000	-1.954504000
C	-0.035457000	-1.817420000	-1.785913000
C	-0.715729000	0.009543000	-3.039944000
C	-0.239377000	-2.520148000	-0.529426000
N	0.901234000	-2.163320000	-2.661754000
C	0.250078000	-0.291550000	-3.989632000
H	-1.397352000	0.856602000	-3.134231000
C	-1.277284000	-2.006821000	0.287615000
C	0.578460000	-3.578634000	-0.117899000
C	1.037841000	-1.413678000	-3.749704000
H	0.389191000	0.340105000	-4.866071000
C	-1.418709000	-2.568621000	1.566388000
C	0.398811000	-4.127441000	1.145495000
H	1.360192000	-3.938316000	-0.789204000
H	1.815157000	-1.716589000	-4.460588000
C	-0.589866000	-3.607533000	1.989383000
H	-2.166903000	-2.177130000	2.259767000
H	1.038148000	-4.945546000	1.484708000
H	-0.713145000	-4.020854000	2.994583000
Br	-0.728368000	1.060944000	0.869992000
C	1.262413000	1.711313000	1.733847000
O	1.796160000	0.652527000	2.372099000
C	2.018248000	2.196376000	0.526618000
H	0.962284000	2.486581000	2.445350000
C	2.062353000	-0.484292000	1.561441000
C	2.337755000	1.031971000	-0.406550000
H	1.457937000	2.975437000	-0.007993000
O	3.254405000	2.737224000	1.013707000
C	2.645840000	-1.579222000	2.419511000
C	2.957038000	-0.100462000	0.384048000
H	1.094498000	-0.850684000	1.172147000
O	3.268534000	1.433450000	-1.394623000
H	1.403211000	0.703066000	-0.882627000
C	3.806324000	3.758909000	0.337937000
H	2.093735000	-1.609150000	3.369563000
H	2.538475000	-2.543643000	1.907753000
O	4.017177000	-1.344107000	2.707417000
O	3.082962000	-1.240935000	-0.441858000
H	3.947962000	0.207190000	0.746237000
C	2.773082000	2.035139000	-2.494658000
O	3.285267000	4.300279000	-0.601753000
C	5.151464000	4.098089000	0.902320000
C	4.906992000	-2.261279000	2.286161000
C	4.316734000	-1.619811000	-0.834713000
O	1.595997000	2.145167000	-2.711760000
C	3.877394000	2.554925000	-3.357796000
H	5.865695000	3.325069000	0.578605000
H	5.128517000	4.086539000	1.999931000
H	5.476849000	5.073996000	0.525402000
O	4.596408000	-3.295149000	1.751112000
C	6.310680000	-1.802471000	2.519182000
O	5.316517000	-1.013662000	-0.551847000
C	4.250716000	-2.900038000	-1.600979000

H	3.515135000	2.696308000	-4.382349000
H	4.749608000	1.889302000	-3.328950000
H	4.173970000	3.531949000	-2.945029000
H	6.968237000	-2.671447000	2.638907000
H	6.616248000	-1.254792000	1.613434000
H	6.377601000	-1.128136000	3.381429000
H	4.285275000	-3.711455000	-0.856347000
H	3.306840000	-2.975746000	-2.157591000
H	5.125343000	-2.987380000	-2.255850000

int3
Lowest frequency = 25.3533 cm^{-1}
Charge = 0, Multiplicity = 1

48

Ru	0.297162000	-0.053692000	0.305839000
C	0.489242000	-0.545393000	2.847861000
O	0.950452000	0.479418000	2.254329000
O	-0.126800000	-1.408103000	2.178655000
C	0.728752000	-0.714197000	4.316690000
H	-0.044429000	-1.355245000	4.757514000
H	1.706161000	-1.200878000	4.459213000
H	0.762339000	0.261472000	4.817777000
N	-1.359166000	1.219747000	0.677545000
C	-2.644117000	1.078428000	0.261732000
C	-1.034567000	2.378059000	1.279603000
C	-3.162422000	-0.232401000	-0.160256000
N	-3.511455000	2.091243000	0.239240000
C	-1.921370000	3.436630000	1.372263000
H	-0.017750000	2.438563000	1.671905000
C	-4.133877000	-0.286842000	-1.167911000
C	-2.750473000	-1.412012000	0.470539000
C	-3.157278000	3.255377000	0.756413000
H	-1.636808000	4.369453000	1.859374000
C	-4.644841000	-1.511921000	-1.576553000
H	-4.461305000	0.642806000	-1.635174000
C	-3.282257000	-2.633893000	0.071539000
H	-2.019332000	-1.380474000	1.280471000
H	-3.887879000	4.069863000	0.689654000
C	-4.217090000	-2.688842000	-0.960069000
H	-5.380692000	-1.551942000	-2.382863000
H	-2.956961000	-3.550187000	0.569133000
H	-4.621043000	-3.651707000	-1.282404000
N	1.964426000	-1.176067000	0.063370000
C	3.072428000	-0.511342000	-0.354057000
C	2.051713000	-2.486532000	0.301698000
C	2.824255000	0.882082000	-0.675805000
N	4.262595000	-1.084486000	-0.498265000
C	3.260178000	-3.151129000	0.152388000
H	1.133479000	-2.978537000	0.629679000
C	1.482865000	1.304394000	-0.524090000
C	3.816654000	1.733384000	-1.171733000
C	4.355067000	-2.382503000	-0.245119000
H	3.343054000	-4.221235000	0.342773000
C	1.157674000	2.597957000	-0.951431000
C	3.474920000	3.024696000	-1.551996000

H	4.837380000	1.355918000	-1.264562000
H	5.344438000	-2.838077000	-0.366448000
C	2.143905000	3.444819000	-1.455779000
H	0.124363000	2.948144000	-0.922924000
H	4.236447000	3.702533000	-1.943850000
H	1.869565000	4.449453000	-1.789261000
Br	-0.427038000	-0.758400000	-1.942845000

TS4^{oss}

Lowest frequency = -302.6441 cm^{-1}

Charge = 0, Multiplicity = 1

90

Ru	-1.941567000	-0.674456000	-0.479569000
C	-3.249347000	0.271196000	-2.516611000
O	-3.384539000	-0.879006000	-1.984238000
O	-2.398129000	1.062770000	-2.057234000
C	-4.144164000	0.650826000	-3.658292000
H	-3.626050000	1.349490000	-4.327228000
H	-4.480174000	-0.238722000	-4.205420000
H	-5.030486000	1.164499000	-3.254098000
N	-3.536888000	-0.063252000	0.780310000
C	-3.552525000	0.847731000	1.787374000
C	-4.633961000	-0.829916000	0.638436000
C	-2.555885000	1.928826000	1.852662000
N	-4.479940000	0.847164000	2.747059000
C	-5.679629000	-0.805551000	1.545237000
H	-4.632858000	-1.496504000	-0.226495000
C	-2.149638000	2.404449000	3.106106000
C	-2.073023000	2.539367000	0.689508000
C	-5.509075000	0.022810000	2.651786000
H	-6.555353000	-1.441403000	1.413663000
C	-1.234667000	3.445859000	3.193480000
H	-2.551350000	1.932932000	4.003994000
C	-1.176460000	3.598534000	0.784357000
H	-2.396227000	2.190515000	-0.292992000
H	-6.230072000	0.031776000	3.477533000
C	-0.745063000	4.044658000	2.031758000
H	-0.900632000	3.795213000	4.173210000
H	-0.804777000	4.069894000	-0.128093000
H	-0.027825000	4.866357000	2.101676000
N	-0.541590000	-1.399670000	-1.751413000
C	-0.265419000	-2.718343000	-1.612277000
C	0.140771000	-0.700566000	-2.661802000
C	-0.957682000	-3.333069000	-0.488064000
N	0.617938000	-3.375946000	-2.348415000
C	1.097585000	-1.320084000	-3.453404000
H	-0.107396000	0.361105000	-2.734196000
C	-1.872042000	-2.474518000	0.206140000
C	-0.576816000	-4.569350000	0.000549000
C	1.294951000	-2.686471000	-3.255609000
H	1.669114000	-0.755376000	-4.190185000
C	-2.465877000	-3.004623000	1.385339000
C	-0.990354000	-4.937666000	1.297195000
H	0.155335000	-5.161342000	-0.556992000
H	2.032016000	-3.242876000	-3.844636000

C	-2.040221000	-4.202956000	1.915756000
H	-3.206786000	-2.423951000	1.936105000
H	-0.713151000	-5.916400000	1.697142000
H	-2.446737000	-4.554190000	2.867764000
Br	-0.058711000	-0.099658000	1.087803000
C	0.739882000	-3.672790000	2.334198000
O	0.926602000	-4.119715000	3.581983000
C	1.898561000	-3.668620000	1.389919000
H	0.085587000	-2.796507000	2.293470000
C	1.774007000	-5.241016000	3.783030000
C	2.751935000	-4.917545000	1.542988000
H	1.553753000	-3.574773000	0.353840000
O	2.686223000	-2.514632000	1.700429000
C	1.980333000	-5.423345000	5.269132000
C	3.081321000	-5.094606000	3.005844000
H	1.246652000	-6.140373000	3.409089000
O	3.942850000	-4.789997000	0.800404000
H	2.182791000	-5.785750000	1.174016000
C	3.154930000	-1.796031000	0.662310000
H	1.013949000	-5.288849000	5.776643000
H	2.367360000	-6.430608000	5.473627000
O	2.884968000	-4.459597000	5.777286000
O	3.836241000	-6.272539000	3.208415000
H	3.645003000	-4.223157000	3.367175000
C	3.864338000	-5.130055000	-0.506932000
O	3.073675000	-2.158595000	-0.483780000
C	3.744385000	-0.505145000	1.127255000
C	4.018460000	-4.908329000	6.354109000
C	5.129649000	-6.131491000	3.580500000
O	2.900605000	-5.662120000	-0.987124000
C	5.100350000	-4.720084000	-1.238290000
H	4.255410000	-0.626860000	2.090578000
H	2.907057000	0.198149000	1.266162000
H	4.423047000	-0.110019000	0.363073000
O	4.259009000	-6.076920000	6.517354000
C	4.932413000	-3.779360000	6.701256000
O	5.662357000	-5.064331000	3.722468000
C	5.756083000	-7.461268000	3.844415000
H	5.192232000	-5.294603000	-2.166834000
H	5.991716000	-4.832556000	-0.608047000
H	4.984287000	-3.651838000	-1.480319000
H	5.568458000	-4.065908000	7.546823000
H	5.574148000	-3.612328000	5.821397000
H	4.375223000	-2.858699000	6.911880000
H	5.515202000	-7.722718000	4.887093000
H	5.348544000	-8.237014000	3.184617000
H	6.844420000	-7.382829000	3.742883000

TS4^{tri}

Lowest frequency = -483.4498 cm^{-1}

Charge = 0, Multiplicity = 1

90

Ru	-2.993272000	0.413365000	-0.449445000
C	-4.875697000	1.978552000	-1.296576000
O	-4.017029000	1.390248000	-2.024415000

O	-4.849094000	1.790072000	-0.056419000
C	-5.864054000	2.909847000	-1.926956000
H	-6.786695000	2.941938000	-1.334218000
H	-5.430022000	3.921728000	-1.941880000
H	-6.072181000	2.613356000	-2.962474000
N	-4.116400000	-1.291752000	-0.997219000
C	-4.612908000	-2.257767000	-0.183228000
C	-4.212150000	-1.494035000	-2.323322000
C	-4.762174000	-2.015587000	1.260046000
N	-5.007569000	-3.449645000	-0.632040000
C	-4.692566000	-2.678465000	-2.853805000
H	-3.865869000	-0.671999000	-2.952694000
C	-4.597870000	-3.084783000	2.149906000
C	-5.112387000	-0.752225000	1.750793000
C	-5.026566000	-3.671399000	-1.935526000
H	-4.757222000	-2.828191000	-3.931680000
C	-4.726392000	-2.877736000	3.517164000
H	-4.346681000	-4.068363000	1.750797000
C	-5.258290000	-0.555626000	3.120355000
H	-5.287654000	0.080140000	1.066038000
H	-5.329063000	-4.672879000	-2.262743000
C	-5.051770000	-1.611178000	4.005795000
H	-4.569601000	-3.708116000	4.209361000
H	-5.532993000	0.432690000	3.495523000
H	-5.152767000	-1.450897000	5.082041000
N	-1.933892000	2.108465000	-0.063984000
C	-0.846523000	2.318998000	-0.845616000
C	-2.219173000	3.003464000	0.885683000
C	-0.509635000	1.185633000	-1.688697000
N	-0.078638000	3.397482000	-0.767956000
C	-1.434235000	4.134615000	1.047693000
H	-3.095060000	2.789863000	1.501604000
C	-1.402982000	0.091696000	-1.620471000
C	0.724627000	1.102871000	-2.327228000
C	-0.363929000	4.291348000	0.165151000
H	-1.649926000	4.861916000	1.830346000
C	-1.039168000	-1.092143000	-2.296138000
C	1.205486000	-0.190974000	-2.684047000
H	1.372196000	1.983637000	-2.346024000
H	0.293330000	5.166109000	0.220907000
C	0.212665000	-1.220169000	-2.867525000
H	-1.721768000	-1.943427000	-2.333930000
H	2.117089000	-0.255563000	-3.286952000
H	0.500230000	-2.156944000	-3.351747000
Br	-1.705217000	-0.741773000	1.332817000
C	2.010870000	-0.824084000	-0.877696000
O	2.988510000	-1.756908000	-1.084536000
C	2.372034000	0.367993000	-0.039574000
H	1.103691000	-1.314612000	-0.512681000
C	4.277442000	-1.279900000	-1.400520000
C	3.767669000	0.886880000	-0.348017000
H	1.648306000	1.176946000	-0.194672000
O	2.294824000	-0.026870000	1.334469000
C	5.209662000	-2.462917000	-1.533990000
C	4.742592000	-0.265450000	-0.355387000
H	4.252497000	-0.772799000	-2.386583000
O	4.160446000	1.835446000	0.620863000
H	3.753153000	1.367829000	-1.339273000

C	1.703265000	0.827923000	2.184708000
H	4.704684000	-3.239606000	-2.126665000
H	6.138622000	-2.160039000	-2.035059000
O	5.518723000	-3.010745000	-0.263399000
O	6.032538000	0.188948000	-0.720894000
H	4.779355000	-0.735231000	0.637371000
C	3.798485000	3.116590000	0.390150000
O	1.373086000	1.948245000	1.884074000
C	1.478170000	0.190984000	3.518186000
C	6.812103000	-3.039989000	0.109636000
C	6.998440000	0.170706000	0.223095000
O	3.313814000	3.488470000	-0.643971000
C	4.051002000	3.963632000	1.595643000
H	2.292479000	-0.499478000	3.771175000
H	0.542472000	-0.386903000	3.448194000
H	1.359761000	0.965384000	4.284457000
O	7.717383000	-2.692956000	-0.605505000
C	6.956955000	-3.501422000	1.523364000
O	6.812535000	-0.190617000	1.354178000
C	8.312180000	0.592334000	-0.350328000
H	4.115420000	5.018032000	1.303915000
H	4.955151000	3.640336000	2.127049000
H	3.189564000	3.823870000	2.267606000
H	7.941845000	-3.962172000	1.662550000
H	6.897961000	-2.601092000	2.155536000
H	6.149494000	-4.186311000	1.808879000
H	8.766220000	-0.307518000	-0.794801000
H	8.184926000	1.346413000	-1.136768000
H	8.964175000	0.958098000	0.451013000

int5

Lowest frequency = 5.9465 cm^{-1}

Charge = 0, Multiplicity = 1

90

Ru	-3.179455000	0.402757000	-0.537305000
C	-5.653318000	1.086465000	-1.007338000
O	-4.717513000	0.925419000	-1.859358000
O	-5.415659000	0.912471000	0.205965000
C	-7.002683000	1.522361000	-1.496566000
H	-7.778453000	1.238175000	-0.775250000
H	-7.005141000	2.618937000	-1.597741000
H	-7.212868000	1.095448000	-2.485624000
N	-3.713990000	-1.615135000	-0.954647000
C	-3.663848000	-2.709635000	-0.151549000
C	-3.987961000	-1.832970000	-2.253823000
C	-3.727646000	-2.585466000	1.312041000
N	-3.609498000	-3.953584000	-0.632689000
C	-4.059154000	-3.104264000	-2.797227000
H	-4.124181000	-0.934169000	-2.859514000
C	-3.063051000	-3.528300000	2.106996000
C	-4.513730000	-1.597578000	1.916945000
C	-3.768872000	-4.154515000	-1.928647000
H	-4.277375000	-3.255675000	-3.854634000
C	-3.147281000	-3.454760000	3.491318000
H	-2.470945000	-4.304455000	1.620298000

C	-4.615777000	-1.546537000	3.302956000
H	-5.048751000	-0.866246000	1.307990000
H	-3.679644000	-5.187003000	-2.286133000
C	-3.924492000	-2.463134000	4.092076000
H	-2.605692000	-4.175743000	4.108050000
H	-5.230452000	-0.772710000	3.767651000
H	-3.993096000	-2.408686000	5.181458000
N	-2.702919000	2.357188000	-0.285553000
C	-1.518267000	2.724089000	-0.825425000
C	-3.396470000	3.269052000	0.400805000
C	-0.834832000	1.604632000	-1.469696000
N	-0.997012000	3.935838000	-0.741258000
C	-2.913083000	4.564259000	0.532077000
H	-4.336813000	2.924333000	0.838624000
C	-1.656131000	0.393818000	-1.594515000
C	0.489628000	1.616636000	-1.726976000
C	-1.688842000	4.850542000	-0.070479000
H	-3.468270000	5.316817000	1.092628000
C	-1.054016000	-0.665546000	-2.386229000
C	1.189058000	0.350095000	-2.063180000
H	1.068630000	2.527715000	-1.540875000
H	-1.247055000	5.850625000	-0.004280000
C	0.272398000	-0.682589000	-2.630171000
H	-1.662033000	-1.502721000	-2.728521000
H	2.033865000	0.544429000	-2.745407000
H	0.728532000	-1.535219000	-3.140477000
Br	-1.646166000	-0.033637000	1.446501000
C	1.766106000	-0.266856000	-0.730419000
O	2.608204000	-1.358268000	-0.990957000
C	2.428723000	0.749239000	0.188666000
H	0.902711000	-0.665574000	-0.176587000
C	3.893973000	-1.055540000	-1.474247000
C	3.801916000	1.137862000	-0.335152000
H	1.802422000	1.643079000	0.304946000
O	2.552499000	0.120960000	1.456994000
C	4.640605000	-2.352583000	-1.704158000
C	4.626278000	-0.115118000	-0.516111000
H	3.831100000	-0.553283000	-2.462516000
O	4.458843000	2.003973000	0.565303000
H	3.682353000	1.652947000	-1.302526000
C	2.333084000	0.880484000	2.547013000
H	3.959926000	-3.061365000	-2.197883000
H	5.519534000	-2.178823000	-2.339084000
O	5.057502000	-2.927294000	-0.477394000
O	5.886057000	0.201286000	-1.079314000
H	4.766170000	-0.606462000	0.455813000
C	4.072349000	3.296937000	0.516064000
O	2.184870000	2.073276000	2.505136000
C	2.270311000	0.032132000	3.776676000
C	6.378330000	-3.108955000	-0.288180000
C	6.976416000	0.064991000	-0.292832000
O	3.323731000	3.720271000	-0.325143000
C	4.662998000	4.078351000	1.642599000
H	3.078459000	-0.711393000	3.775877000
H	1.313482000	-0.512950000	3.766176000
H	2.319971000	0.666372000	4.668285000
O	7.207937000	-2.877424000	-1.130559000
C	6.667017000	-3.575272000	1.101673000

O	6.923984000	-0.275997000	0.858271000
C	8.227995000	0.328463000	-1.065031000
H	4.654406000	5.146960000	1.400539000
H	5.675441000	3.728776000	1.881377000
H	4.021368000	3.899124000	2.519580000
H	7.593640000	-4.161010000	1.109913000
H	6.819849000	-2.670208000	1.711001000
H	5.828213000	-4.145285000	1.518789000
H	8.503678000	-0.620524000	-1.551831000
H	8.071587000	1.088668000	-1.840177000
H	9.031199000	0.618805000	-0.378293000

intE
Lowest frequency = 8.3272 cm^{-1}
Charge = 0, Multiplicity = 1

78

Ru	-1.052681000	-0.685318000	-1.130228000
O	-1.105550000	0.809605000	-2.658424000
C	-1.857747000	1.540219000	-1.944240000
O	-2.149831000	1.107143000	-0.797518000
C	-0.471011000	-3.029256000	-0.463693000
O	-1.145817000	-2.273679000	0.295702000
O	-0.077012000	-2.530255000	-1.557589000
P	0.723574000	0.152601000	-0.021656000
C	1.679321000	-1.156549000	0.844938000
C	2.535443000	-1.959823000	0.078999000
C	1.485104000	-1.456912000	2.195136000
C	3.188695000	-3.041875000	0.655864000
H	2.684027000	-1.741972000	-0.980913000
C	2.131549000	-2.546168000	2.773849000
H	0.818957000	-0.841009000	2.802243000
C	2.977970000	-3.341673000	2.003394000
H	3.857636000	-3.663177000	0.057317000
H	1.977058000	-2.779281000	3.828865000
C	0.210966000	1.283342000	1.333379000
C	-1.000238000	0.978541000	1.969517000
C	0.927460000	2.414930000	1.732594000
C	-1.475275000	1.778819000	3.000614000
H	-1.581359000	0.118344000	1.630827000
C	0.448840000	3.223054000	2.761201000
H	1.862044000	2.681061000	1.235729000
C	-0.750445000	2.904034000	3.396285000
H	-2.420579000	1.535217000	3.489189000
H	1.011451000	4.104832000	3.073147000
C	2.077241000	1.057382000	-0.872555000
C	1.852228000	1.582275000	-2.148947000
C	3.331587000	1.229975000	-0.268544000
C	2.862624000	2.282552000	-2.805777000
H	0.881413000	1.431389000	-2.628219000
C	4.338600000	1.928971000	-0.923249000
H	3.526420000	0.806755000	0.719884000
C	4.102421000	2.456496000	-2.194734000
H	2.684539000	2.695195000	-3.800445000
H	5.311139000	2.068660000	-0.447180000
C	-0.139318000	-4.428349000	-0.066001000

H	0.214253000	-5.001663000	-0.930957000
H	0.656173000	-4.406334000	0.694410000
H	-1.017309000	-4.909082000	0.385653000
C	-2.341393000	2.869984000	-2.419688000
H	-3.327812000	3.086908000	-1.990574000
H	-1.641103000	3.644941000	-2.070028000
H	-2.374344000	2.899750000	-3.515710000
C	3.594113000	-4.577785000	2.593395000
C	-1.299198000	3.804501000	4.467961000
C	5.212825000	3.164742000	-2.921498000
F	3.751587000	-4.478967000	3.914716000
F	2.818766000	-5.650389000	2.369196000
F	4.788406000	-4.843670000	2.059068000
F	-2.142689000	4.710759000	3.960127000
F	-1.976394000	3.115368000	5.391405000
F	-0.330910000	4.478089000	5.095358000
F	4.743614000	4.025642000	-3.827585000
F	5.999776000	2.298598000	-3.570015000
F	5.994033000	3.847654000	-2.079341000
N	-2.751387000	-1.440759000	-2.195571000
C	-2.408550000	-2.019630000	-3.356541000
C	-4.053402000	-1.118402000	-2.013676000
C	-3.327504000	-2.227883000	-4.371911000
H	-1.356397000	-2.298993000	-3.451393000
C	-4.522092000	-0.603265000	-0.712520000
N	-4.973757000	-1.238890000	-2.972493000
C	-4.619667000	-1.767077000	-4.133548000
H	-3.036488000	-2.695275000	-5.312834000
C	-3.970700000	-1.050101000	0.493546000
C	-5.574293000	0.321151000	-0.690896000
H	-5.397135000	-1.831254000	-4.903697000
C	-4.452371000	-0.554577000	1.701863000
H	-3.165855000	-1.786118000	0.488299000
C	-6.035851000	0.829651000	0.518053000
H	-6.015520000	0.637728000	-1.637506000
C	-5.473254000	0.394988000	1.718478000
H	-4.026239000	-0.918366000	2.640340000
H	-6.842205000	1.566615000	0.525733000
H	-5.840499000	0.789696000	2.669118000

intF

Lowest frequency = 10.5429 cm^{-1}

Charge = 0, Multiplicity = 1

78

Ru	-1.614421000	-0.697967000	-0.292753000
C	-3.681937000	-1.338941000	1.682941000
C	-2.323052000	-0.921115000	1.700626000
C	-1.842067000	-0.322876000	2.876136000
H	-1.470021000	-1.619472000	1.146422000
C	-4.009381000	-0.552475000	3.941671000
C	-2.678835000	-0.126192000	3.970129000
H	-0.797588000	-0.020093000	2.937626000
H	-4.654100000	-0.405230000	4.810523000
H	-2.280014000	0.354819000	4.866802000
N	-3.402876000	-1.666300000	-0.640967000

C	-4.507077000	-1.160048000	2.795993000
H	-5.546025000	-1.488937000	2.726535000
O	-0.202916000	-3.273259000	0.927662000
C	-0.202574000	-3.278514000	-0.301284000
O	-0.758135000	-2.375018000	-1.034375000
C	-2.412985000	1.256356000	-1.634086000
O	-1.775333000	0.292675000	-2.168373000
O	-2.630338000	1.191601000	-0.393569000
P	0.470506000	0.246951000	-0.004783000
C	1.680862000	-0.499886000	-1.163152000
C	1.258977000	-0.750361000	-2.477355000
C	2.965259000	-0.882527000	-0.767609000
C	2.111548000	-1.379963000	-3.375075000
H	0.252273000	-0.461377000	-2.786113000
C	3.814975000	-1.526155000	-1.664706000
H	3.307542000	-0.691841000	0.250925000
C	3.384469000	-1.781897000	-2.964422000
H	1.782153000	-1.575859000	-4.397162000
H	4.814713000	-1.831638000	-1.351304000
C	1.369937000	0.200393000	1.593056000
C	1.431332000	-1.019355000	2.284000000
C	2.012715000	1.325388000	2.120274000
C	2.122027000	-1.101287000	3.488167000
H	0.929826000	-1.907981000	1.882507000
C	2.695709000	1.241685000	3.331193000
H	1.985986000	2.276501000	1.585582000
C	2.747364000	0.029449000	4.016722000
H	2.171245000	-2.053148000	4.020537000
H	3.198097000	2.120316000	3.739537000
C	0.471647000	2.027859000	-0.434567000
C	-0.367945000	2.869252000	0.310301000
C	1.184540000	2.551557000	-1.515856000
C	-0.492751000	4.211189000	-0.025075000
H	-0.946764000	2.463166000	1.142802000
C	1.048625000	3.893954000	-1.863221000
H	1.845286000	1.907473000	-2.099155000
C	0.204271000	4.719635000	-1.123776000
H	-1.148330000	4.863231000	0.555036000
H	1.600436000	4.300361000	-2.712665000
C	0.509127000	-4.349727000	-1.091339000
H	-0.100100000	-4.670386000	-1.947088000
H	1.441485000	-3.925490000	-1.497440000
H	0.752123000	-5.203135000	-0.447618000
C	-2.836517000	2.438398000	-2.438961000
H	-3.781475000	2.841624000	-2.053426000
H	-2.070386000	3.223768000	-2.337513000
H	-2.926918000	2.173162000	-3.499250000
C	3.435568000	-0.055314000	5.351866000
C	-0.032547000	6.138540000	-1.558613000
C	4.257411000	-2.560110000	-3.908719000
F	2.564627000	0.098816000	6.355848000
F	4.028704000	-1.238748000	5.525163000
F	4.368140000	0.890686000	5.490726000
F	-0.256114000	6.946273000	-0.519864000
F	-1.108632000	6.217219000	-2.355413000
F	1.000344000	6.631625000	-2.244150000
F	5.552245000	-2.439668000	-3.606801000
F	4.096713000	-2.161836000	-5.173692000

F	3.962342000	-3.865804000	-3.868423000
C	-5.775902000	-2.923231000	-0.822991000
H	-6.754880000	-3.414162000	-0.863896000
C	-4.991591000	-2.800546000	-1.971921000
H	-5.314692000	-3.193074000	-2.936267000
C	-3.780532000	-2.139320000	-1.830076000
H	-3.090674000	-1.959841000	-2.658298000
C	-4.206014000	-1.862626000	0.430551000
N	-5.390232000	-2.464939000	0.359395000

TSG

Lowest frequency = -931.3761 cm^{-1}

Charge = 0, Multiplicity = 1

78

Ru	1.697665000	-0.023301000	0.668140000
C	3.921716000	-1.641611000	-0.274378000
C	2.514582000	-1.524283000	-0.473365000
C	1.996845000	-2.170642000	-1.611139000
H	1.702737000	-1.903878000	0.741001000
C	4.191593000	-2.991719000	-2.255100000
C	2.817248000	-2.877862000	-2.486241000
H	0.927495000	-2.130408000	-1.817734000
H	4.823589000	-3.557343000	-2.942905000
H	2.372862000	-3.357624000	-3.362679000
N	3.602625000	-0.074587000	1.462478000
C	4.742070000	-2.368887000	-1.144147000
H	5.812698000	-2.413353000	-0.932591000
O	1.312067000	-2.784925000	1.666488000
C	0.991916000	-2.013843000	2.608896000
O	1.049837000	-0.759274000	2.484359000
C	2.106793000	2.361449000	-0.024667000
O	1.592957000	2.129200000	1.110085000
O	2.393535000	1.372986000	-0.760840000
P	-0.467408000	0.054485000	-0.102318000
C	-1.653917000	0.413847000	1.256318000
C	-1.258219000	1.327546000	2.244717000
C	-2.909068000	-0.194992000	1.348393000
C	-2.112346000	1.630902000	3.297701000
H	-0.274989000	1.798735000	2.179890000
C	-3.760952000	0.102202000	2.410357000
H	-3.230050000	-0.912853000	0.591476000
C	-3.363846000	1.016702000	3.382918000
H	-1.798917000	2.338327000	4.068087000
H	-4.736055000	-0.382221000	2.484031000
C	-1.312229000	-1.336016000	-0.957740000
C	-1.151758000	-2.634040000	-0.452968000
C	-2.146708000	-1.130805000	-2.061424000
C	-1.815757000	-3.703689000	-1.042195000
H	-0.484680000	-2.820541000	0.391674000
C	-2.806152000	-2.203400000	-2.656982000
H	-2.289064000	-0.125063000	-2.461211000
C	-2.640654000	-3.489930000	-2.147769000
H	-1.687175000	-4.711495000	-0.642897000
H	-3.459195000	-2.037504000	-3.515622000
C	-0.714132000	1.448008000	-1.267749000

C	0.052333000	1.442945000	-2.442223000
C	-1.536691000	2.541960000	-0.987909000
C	-0.006603000	2.515918000	-3.322014000
H	0.719250000	0.604012000	-2.654018000
C	-1.586066000	3.625493000	-1.862431000
H	-2.138325000	2.556370000	-0.077165000
C	-0.815811000	3.614866000	-3.023535000
H	0.592737000	2.510981000	-4.234218000
H	-2.224600000	4.482334000	-1.640239000
C	0.472846000	-2.578925000	3.893315000
H	-0.618324000	-2.695894000	3.798183000
H	0.908618000	-3.566762000	4.085189000
H	0.668723000	-1.891145000	4.724529000
C	2.323077000	3.756271000	-0.512230000
H	3.250406000	3.815381000	-1.096564000
H	1.491067000	4.026773000	-1.181903000
H	2.343961000	4.461215000	0.327392000
C	-3.308643000	-4.660389000	-2.816610000
C	-0.781439000	4.825351000	-3.913216000
C	-4.290553000	1.382213000	4.509464000
F	-2.491893000	-5.246068000	-3.698930000
F	-3.664091000	-5.594396000	-1.930556000
F	-4.407066000	-4.291126000	-3.480166000
F	-0.589937000	4.497687000	-5.192856000
F	0.221350000	5.644456000	-3.563594000
F	-1.910052000	5.533822000	-3.838480000
F	-4.913810000	2.541828000	4.269059000
F	-3.628584000	1.527531000	5.661354000
F	-5.235236000	0.458046000	4.699457000
C	6.161342000	-0.323608000	2.262920000
H	7.211507000	-0.440927000	2.554621000
C	5.319076000	0.538596000	2.969020000
H	5.667534000	1.114464000	3.826512000
C	4.012987000	0.638553000	2.512540000
H	3.269736000	1.302477000	2.961703000
C	4.479809000	-0.907057000	0.846587000
N	5.752074000	-1.032945000	1.221957000

intH

Lowest frequency = 9.0546 cm^{-1}

Charge = 0, Multiplicity = 1

78

Ru	-1.737027000	0.067186000	-0.526734000
C	-3.934001000	-1.409859000	0.652740000
C	-2.520252000	-1.228192000	0.791721000
C	-1.957239000	-1.835373000	1.936439000
H	-1.953708000	-2.481482000	-0.488533000
C	-4.094725000	-2.751073000	2.651498000
C	-2.719957000	-2.572292000	2.839808000
H	-0.890129000	-1.732041000	2.134027000
H	-4.681049000	-3.336880000	3.362519000
H	-2.229970000	-3.019056000	3.709869000
N	-3.703616000	0.069106000	-1.171476000
C	-4.701579000	-2.158498000	1.554048000
H	-5.775641000	-2.246566000	1.374569000

O	-1.736772000	-3.105737000	-1.245440000
C	-1.370694000	-2.369136000	-2.255163000
O	-1.292686000	-1.142717000	-2.181883000
C	-1.897247000	2.593267000	-0.251939000
O	-1.500713000	2.191885000	-1.379281000
O	-2.190065000	1.735348000	0.637046000
P	0.464284000	0.085819000	0.094693000
C	1.589294000	0.164260000	-1.363915000
C	1.163203000	0.906974000	-2.474825000
C	2.828657000	-0.483268000	-1.408516000
C	1.964511000	0.994297000	-3.607743000
H	0.202628000	1.426609000	-2.436531000
C	3.626960000	-0.403143000	-2.547057000
H	3.180555000	-1.057832000	-0.549734000
C	3.192800000	0.331982000	-3.648598000
H	1.634127000	1.582229000	-4.466347000
H	4.594221000	-0.908057000	-2.576189000
C	1.287349000	-1.237923000	1.076817000
C	1.074695000	-2.571880000	0.702506000
C	2.132571000	-0.964905000	2.155791000
C	1.692765000	-3.608670000	1.388577000
H	0.401037000	-2.808729000	-0.122102000
C	2.747541000	-2.003154000	2.853618000
H	2.318381000	0.067616000	2.457183000
C	2.528034000	-3.324193000	2.471333000
H	1.522066000	-4.644033000	1.087542000
H	3.408296000	-1.782946000	3.693912000
C	0.895615000	1.598008000	1.038377000
C	0.190296000	1.830920000	2.227902000
C	1.795144000	2.560089000	0.572749000
C	0.388963000	3.005402000	2.942103000
H	-0.538998000	1.097360000	2.579098000
C	1.984284000	3.745667000	1.279694000
H	2.347082000	2.390994000	-0.353810000
C	1.276752000	3.969795000	2.459093000
H	-0.162502000	3.185520000	3.866663000
H	2.682900000	4.499467000	0.912248000
C	-1.004891000	-3.106779000	-3.495954000
H	-0.933167000	-2.411636000	-4.339024000
H	-0.025975000	-3.587586000	-3.343231000
H	-1.735703000	-3.900924000	-3.698508000
C	-1.980762000	4.050179000	0.079434000
H	-2.853433000	4.248466000	0.715328000
H	-1.083331000	4.332625000	0.652792000
H	-2.018936000	4.653181000	-0.835638000
C	3.146427000	-4.454496000	3.247504000
C	1.396472000	5.289497000	3.167143000
C	4.020590000	0.374177000	-4.902998000
F	2.289007000	-4.954644000	4.143815000
F	3.504188000	-5.460940000	2.444453000
F	4.233471000	-4.062425000	3.915728000
F	1.241081000	5.162666000	4.487076000
F	0.456406000	6.146296000	2.742382000
F	2.579910000	5.868342000	2.950777000
F	5.315384000	0.163550000	-4.653171000
F	3.913694000	1.550991000	-5.525801000
F	3.630211000	-0.565586000	-5.773857000
C	-6.321830000	-0.147664000	-1.778090000

H	-7.390862000	-0.257915000	-1.994703000
C	-5.518581000	0.683088000	-2.562252000
H	-5.917824000	1.247021000	-3.405487000
C	-4.179800000	0.764697000	-2.205853000
H	-3.458171000	1.401269000	-2.725454000
C	-4.554760000	-0.717260000	-0.461119000
N	-5.851370000	-0.833660000	-0.747091000

int1^{p}
Lowest frequency = 8.4231 cm^{-1}
Charge = 0, Multiplicity = 1

113

Ru	0.617739000	-0.196371000	-1.397672000
C	-1.230452000	1.885655000	-2.158468000
C	-0.189711000	1.628677000	-1.215716000
C	0.206025000	2.743851000	-0.450160000
C	-1.418845000	4.205939000	-1.516938000
C	-0.388490000	3.996610000	-0.595203000
H	1.000662000	2.646316000	0.288205000
H	-1.887940000	5.186811000	-1.614611000
H	-0.041969000	4.825933000	0.028195000
N	-0.923407000	-0.369200000	-2.786463000
C	-1.834342000	3.141416000	-2.302950000
H	-2.626275000	3.256442000	-3.045229000
C	2.245209000	-0.798548000	-3.263348000
O	1.794104000	-1.780073000	-2.619731000
O	1.847773000	0.368533000	-2.954016000
P	2.285889000	-0.009756000	0.154709000
C	2.803806000	-1.601841000	0.926870000
C	2.898903000	-2.734602000	0.105923000
C	3.095472000	-1.718850000	2.289973000
C	3.281785000	-3.958676000	0.643418000
H	2.663609000	-2.653220000	-0.958956000
C	3.473747000	-2.946000000	2.828451000
H	3.028253000	-0.847632000	2.943865000
C	3.564826000	-4.067166000	2.005698000
H	3.359984000	-4.836616000	-0.000722000
H	3.704477000	-3.030676000	3.891968000
C	2.012359000	1.019195000	1.654648000
C	0.776402000	0.901707000	2.304283000
C	2.965363000	1.908814000	2.155366000
C	0.497574000	1.658003000	3.433278000
H	0.018361000	0.227546000	1.905586000
C	2.683162000	2.680862000	3.282419000
H	3.933213000	2.011270000	1.660084000
C	1.450768000	2.557616000	3.919072000
H	-0.468152000	1.554212000	3.933018000
H	3.426396000	3.379911000	3.669368000
C	3.862847000	0.649364000	-0.510962000
C	3.793142000	1.799228000	-1.309973000
C	5.100393000	0.036885000	-0.292322000
C	4.947219000	2.332416000	-1.870600000
H	2.824926000	2.264466000	-1.507085000
C	6.256287000	0.563997000	-0.864180000
H	5.165796000	-0.860540000	0.326290000

C	6.178435000	1.709531000	-1.654373000
H	4.891118000	3.230796000	-2.488113000
H	7.222364000	0.086468000	-0.691113000
C	3.279720000	-0.955369000	-4.331567000
H	3.154175000	-0.188812000	-5.106638000
H	4.270865000	-0.810605000	-3.872293000
H	3.237928000	-1.962392000	-4.764024000
C	1.108034000	3.416445000	5.104884000
C	7.411161000	2.245745000	-2.328133000
C	3.917470000	-5.405747000	2.592856000
F	0.269534000	4.401333000	4.762426000
F	0.503089000	2.706233000	6.062287000
F	2.189224000	3.983812000	5.643000000
F	7.379002000	3.577111000	-2.429444000
F	7.533972000	1.761102000	-3.570122000
F	8.523434000	1.916116000	-1.666333000
F	4.664841000	-5.283421000	3.693642000
F	4.595244000	-6.162349000	1.725346000
F	2.820694000	-6.091044000	2.937202000
C	-2.972310000	-0.252309000	-4.530125000
H	-3.811454000	-0.168076000	-5.230266000
C	-2.286781000	-1.456613000	-4.385608000
H	-2.568200000	-2.354654000	-4.933721000
C	-1.238547000	-1.466656000	-3.476562000
H	-0.626289000	-2.350942000	-3.282833000
C	-1.648425000	0.761560000	-2.980803000
N	-2.662799000	0.838729000	-3.840716000
Br	-0.624935000	-1.778258000	0.149399000
C	-2.294017000	-1.396906000	1.456178000
O	-2.400459000	-0.103643000	1.776116000
C	-3.473473000	-2.004236000	0.726657000
H	-1.978708000	-1.976970000	2.330830000
C	-2.786070000	0.785037000	0.719637000
C	-3.902504000	-1.114374000	-0.433553000
H	-3.241785000	-3.018598000	0.372904000
O	-4.525590000	-2.052192000	1.686288000
C	-2.878707000	2.187818000	1.260946000
C	-4.076433000	0.303368000	0.063947000
H	-1.974930000	0.779013000	-0.025450000
O	-5.137490000	-1.556066000	-0.955337000
H	-3.129615000	-1.155507000	-1.215522000
C	-5.421505000	-3.057848000	1.581997000
H	-2.011290000	2.384190000	1.906319000
H	-2.862793000	2.899655000	0.425206000
O	-4.062173000	2.365997000	2.024988000
O	-4.336880000	1.153696000	-1.029060000
H	-4.910585000	0.345096000	0.778439000
C	-5.090262000	-2.537164000	-1.884203000
O	-5.319873000	-3.953232000	0.787975000
C	-6.536264000	-2.870618000	2.561012000
C	-4.940137000	3.296760000	1.599732000
C	-5.576607000	1.677658000	-1.154840000
O	-4.059598000	-2.959806000	-2.333610000
C	-6.464299000	-3.016421000	-2.220359000
H	-7.186664000	-2.062396000	2.191792000
H	-6.148135000	-2.560108000	3.539984000
H	-7.116518000	-3.796088000	2.643191000
O	-4.738142000	4.020196000	0.659473000

C	-6.197805000	3.264184000	2.407024000
O	-6.474717000	1.431879000	-0.394456000
C	-5.627068000	2.613184000	-2.316127000
H	-6.453196000	-3.530464000	-3.187917000
H	-7.184954000	-2.188633000	-2.214530000
H	-6.756596000	-3.732761000	-1.436474000
H	-6.657893000	4.259234000	2.412830000
H	-6.881861000	2.567817000	1.896239000
H	-6.016247000	2.902278000	3.426136000
H	-5.243287000	3.581295000	-1.956793000
H	-4.975017000	2.262288000	-3.127200000
H	-6.663765000	2.741810000	-2.646975000

int1^{tri-p}
Lowest frequency = 7.3065 cm^{-1}
Charge = 0, Multiplicity = 1

113

Ru	0.536765000	0.054596000	-1.408666000
C	-1.047886000	2.432849000	-1.976800000
C	0.011931000	2.006530000	-1.125144000
C	0.554504000	2.993143000	-0.284551000
C	-0.983897000	4.679590000	-1.103847000
C	0.064060000	4.299125000	-0.263318000
H	1.373394000	2.744677000	0.390402000
H	-1.370496000	5.700397000	-1.077778000
H	0.504310000	5.029066000	0.421339000
N	-1.102680000	0.183093000	-2.703539000
C	-1.533404000	3.744129000	-1.972360000
H	-2.345926000	4.007879000	-2.651686000
C	1.785497000	-1.124769000	-3.464433000
O	1.136052000	-1.742377000	-2.552044000
O	1.884127000	0.114766000	-3.441728000
P	2.300483000	-0.061542000	0.059557000
C	2.825087000	-1.726333000	0.655420000
C	2.752474000	-2.814790000	-0.222656000
C	3.300201000	-1.929681000	1.957575000
C	3.153315000	-4.080339000	0.194679000
H	2.350404000	-2.671056000	-1.229018000
C	3.702210000	-3.195097000	2.373793000
H	3.355721000	-1.095709000	2.659974000
C	3.627580000	-4.272552000	1.491743000
H	3.093908000	-4.925263000	-0.493969000
H	4.078328000	-3.345616000	3.387458000
C	2.031418000	0.776097000	1.673711000
C	0.851546000	0.448608000	2.354522000
C	2.897185000	1.725594000	2.219675000
C	0.539333000	1.062622000	3.558908000
H	0.164165000	-0.285312000	1.928411000
C	2.578530000	2.355703000	3.423046000
H	3.820524000	1.989085000	1.699100000
C	1.400196000	2.028267000	4.088979000
H	-0.383429000	0.800025000	4.081199000
H	3.250147000	3.104844000	3.845551000
C	3.868769000	0.671380000	-0.545968000
C	3.783364000	1.693810000	-1.497805000

C	5.125305000	0.257049000	-0.088396000
C	4.938936000	2.309292000	-1.968921000
H	2.806652000	1.993455000	-1.883127000
C	6.281123000	0.867944000	-0.563636000
H	5.205020000	-0.551732000	0.641202000
C	6.186493000	1.896277000	-1.502519000
H	4.869427000	3.109749000	-2.707462000
H	7.260493000	0.548650000	-0.202433000
C	2.459780000	-1.941489000	-4.530997000
H	2.669900000	-1.325901000	-5.413869000
H	3.416306000	-2.313896000	-4.131053000
H	1.848119000	-2.813276000	-4.798263000
C	1.009862000	2.734802000	5.357391000
C	7.439258000	2.519572000	-2.054785000
C	4.005450000	-5.651062000	1.958791000
F	0.029744000	3.618658000	5.134790000
F	0.556573000	1.875271000	6.275224000
F	2.032116000	3.401744000	5.896270000
F	7.237547000	3.784058000	-2.433306000
F	7.883712000	1.853446000	-3.126149000
F	8.427903000	2.518865000	-1.155221000
F	4.947369000	-5.611146000	2.905967000
F	4.469401000	-6.402992000	0.957480000
F	2.953775000	-6.294690000	2.479007000
C	-3.142965000	0.693129000	-4.389677000
H	-3.968832000	0.939798000	-5.066736000
C	-2.668192000	-0.612641000	-4.291827000
H	-3.108605000	-1.438047000	-4.849499000
C	-1.611923000	-0.824260000	-3.416946000
H	-1.148388000	-1.802737000	-3.266692000
C	-1.640901000	1.419430000	-2.843461000
N	-2.638712000	1.693877000	-3.677666000
Br	-0.828495000	-2.282083000	0.052185000
C	-2.383810000	-1.778209000	1.306030000
O	-2.297573000	-0.498637000	1.724421000
C	-3.658881000	-2.130640000	0.557491000
H	-2.216865000	-2.444331000	2.159747000
C	-2.542932000	0.510031000	0.742334000
C	-3.964424000	-1.089919000	-0.515313000
H	-3.593245000	-3.135325000	0.117698000
O	-4.690013000	-2.096127000	1.536807000
C	-2.401278000	1.871669000	1.371592000
C	-3.912888000	0.285407000	0.110410000
H	-1.754754000	0.428144000	-0.030992000
O	-5.261481000	-1.298388000	-1.031812000
H	-3.223601000	-1.180204000	-1.322702000
C	-5.736013000	-2.935642000	1.371075000
H	-1.470897000	1.913390000	1.952781000
H	-2.352731000	2.632393000	0.581108000
O	-3.491933000	2.142268000	2.239008000
O	-4.113378000	1.275099000	-0.874106000
H	-4.694649000	0.367042000	0.878445000
C	-5.375980000	-2.154060000	-2.072270000
O	-5.783417000	-3.768216000	0.506827000
C	-6.799516000	-2.653119000	2.383192000
C	-4.235250000	3.236343000	1.983529000
C	-5.263917000	1.989881000	-0.822489000
O	-4.426293000	-2.639921000	-2.624861000

C	-6.812262000	-2.414396000	-2.387665000
H	-7.311063000	-1.722127000	2.092912000
H	-6.360222000	-2.491622000	3.376391000
H	-7.522160000	-3.476106000	2.401614000
O	-3.985365000	4.018480000	1.103231000
C	-5.421987000	3.308225000	2.889567000
O	-6.134689000	1.768341000	-0.023839000
C	-5.254084000	3.090988000	-1.827904000
H	-6.904030000	-2.824237000	-3.399556000
H	-7.413983000	-1.504166000	-2.268018000
H	-7.172077000	-3.160135000	-1.661347000
H	-5.724457000	4.353907000	3.018943000
H	-6.238626000	2.768220000	2.384395000
H	-5.223093000	2.828014000	3.855201000
H	-4.802432000	3.960071000	-1.322925000
H	-4.634989000	2.836580000	-2.697714000
H	-6.282748000	3.344275000	-2.109480000

TS2$^{oss\text{-}p}$

Lowest frequency = -78.5794 cm^{-1}

Charge = 0, Multiplicity = 1

113

Ru	0.453286000	-1.781626000	-0.681983000
C	-1.632034000	-1.539152000	-2.649334000
C	-0.595827000	-0.777193000	-2.047859000
C	-0.396042000	0.519784000	-2.541749000
C	-2.275322000	0.301148000	-4.064166000
C	-1.223007000	1.050481000	-3.532400000
H	0.406228000	1.144080000	-2.146901000
H	-2.925314000	0.730721000	-4.829660000
H	-1.045912000	2.068694000	-3.889529000
N	-0.734561000	-3.327438000	-1.404027000
C	-2.474657000	-1.003561000	-3.626381000
H	-3.265893000	-1.634495000	-4.037501000
C	2.577382000	-3.119144000	-1.225521000
O	2.103845000	-3.297021000	-0.071340000
O	1.962066000	-2.346459000	-2.021297000
P	1.699084000	-0.092612000	0.275749000
C	1.958295000	-0.381004000	2.072000000
C	2.534561000	-1.594039000	2.474967000
C	1.524858000	0.525899000	3.042983000
C	2.672279000	-1.890896000	3.825588000
H	2.842292000	-2.323756000	1.721836000
C	1.651111000	0.221520000	4.395686000
H	1.068856000	1.472033000	2.747819000
C	2.220640000	-0.987981000	4.787524000
H	3.103353000	-2.844242000	4.136084000
H	1.292375000	0.926020000	5.148737000
C	1.107176000	1.643586000	0.242053000
C	-0.266237000	1.892722000	0.365339000
C	1.992687000	2.717581000	0.110059000
C	-0.742359000	3.198100000	0.366876000
H	-0.961639000	1.053723000	0.447204000
C	1.514801000	4.025842000	0.104629000
H	3.064101000	2.535928000	0.003340000

C	0.148387000	4.265465000	0.237220000
H	-1.814161000	3.387485000	0.450747000
H	2.205850000	4.862784000	-0.009030000
C	3.399438000	0.034131000	-0.397724000
C	3.533472000	0.078958000	-1.792698000
C	4.546250000	0.035495000	0.402414000
C	4.794300000	0.110503000	-2.375507000
H	2.644877000	0.058960000	-2.426648000
C	5.810955000	0.055790000	-0.180997000
H	4.455825000	0.012412000	1.489967000
C	5.933801000	0.082540000	-1.568994000
H	4.894844000	0.141020000	-3.461968000
H	6.705102000	0.051359000	0.444877000
C	3.869611000	-3.736540000	-1.654482000
H	3.833830000	-4.002404000	-2.718738000
H	4.668476000	-2.988084000	-1.525217000
H	4.103621000	-4.613906000	-1.039785000
C	-0.373045000	5.676126000	0.282182000
C	7.292954000	0.004034000	-2.207012000
C	2.371990000	-1.304067000	6.248941000
F	-1.555266000	5.782247000	-0.329336000
F	-0.545433000	6.087756000	1.543640000
F	0.465437000	6.535107000	-0.302247000
F	7.341904000	0.684308000	-3.354349000
F	7.614852000	-1.265644000	-2.488705000
F	8.247977000	0.483986000	-1.407600000
F	3.488811000	-0.768038000	6.754649000
F	2.425598000	-2.616200000	6.474716000
F	1.350153000	-0.811597000	6.965426000
C	-2.651192000	-5.007467000	-2.268299000
H	-3.464704000	-5.654213000	-2.614254000
C	-1.649264000	-5.508710000	-1.437782000
H	-1.633714000	-6.538616000	-1.084323000
C	-0.683985000	-4.604471000	-1.023599000
H	0.148946000	-4.882408000	-0.372526000
C	-1.724944000	-2.934040000	-2.238114000
N	-2.678438000	-3.747062000	-2.679536000
Br	-1.183565000	-1.541265000	1.130014000
C	-0.315543000	-3.542830000	2.912671000
O	-0.413588000	-2.988592000	4.127723000
C	-1.261829000	-4.611857000	2.516339000
H	0.697454000	-3.534127000	2.509703000
C	-1.722306000	-2.682062000	4.587797000
C	-2.682918000	-4.293526000	2.966857000
H	-1.237985000	-4.771189000	1.432061000
O	-0.859870000	-5.838263000	3.176132000
C	-1.630383000	-2.192210000	6.011867000
C	-2.639961000	-3.893401000	4.424766000
H	-2.108336000	-1.851125000	3.967159000
O	-3.511245000	-5.432341000	2.833315000
H	-3.070905000	-3.474844000	2.341262000
C	-1.014569000	-6.990244000	2.515926000
H	-0.811217000	-1.464690000	6.088117000
H	-2.575560000	-1.716080000	6.304615000
O	-1.348225000	-3.265152000	6.895389000
O	-3.929807000	-3.536608000	4.879151000
H	-2.258518000	-4.733025000	5.022676000
C	-4.071153000	-5.636149000	1.620873000

O	-1.370135000	-7.065530000	1.364850000
C	-0.710860000	-8.157863000	3.402814000
C	-2.237104000	-3.522695000	7.872875000
C	-4.534462000	-4.368167000	5.757491000
O	-3.965321000	-4.861096000	0.709900000
C	-4.780156000	-6.952146000	1.578076000
H	-1.544225000	-8.275269000	4.112856000
H	0.197260000	-7.972149000	3.991404000
H	-0.610689000	-9.068166000	2.801479000
O	-3.219115000	-2.853821000	8.070706000
C	-1.871103000	-4.761645000	8.623988000
O	-4.045008000	-5.398932000	6.135515000
C	-5.834047000	-3.798415000	6.222705000
H	-5.515415000	-6.951245000	0.765500000
H	-5.252607000	-7.181171000	2.541772000
H	-4.021763000	-7.724629000	1.374979000
H	-2.264033000	-4.703662000	9.645735000
H	-2.363162000	-5.601108000	8.107338000
H	-0.787358000	-4.929141000	8.620515000
H	-5.599768000	-3.128203000	7.065056000
H	-6.322622000	-3.213039000	5.434144000
H	-6.483497000	-4.604409000	6.582659000

TS2^{tri-p}

Lowest frequency = -130.3559 cm^{-1}

Charge = 0, Multiplicity = 1

113

Ru	1.092749000	-1.007283000	-1.221955000
C	-1.194308000	-0.137789000	-2.780651000
C	-0.130866000	0.402875000	-2.006239000
C	-0.056710000	1.803296000	-1.944708000
C	-2.051247000	2.051693000	-3.307501000
C	-0.997980000	2.614243000	-2.582372000
H	0.742008000	2.286015000	-1.382059000
H	-2.794023000	2.693243000	-3.786888000
H	-0.912834000	3.701653000	-2.503734000
N	-0.210491000	-2.230616000	-2.296737000
C	-2.145199000	0.668768000	-3.411888000
H	-2.945184000	0.186912000	-3.977104000
C	3.163075000	-2.243854000	-2.245181000
O	2.621491000	-2.625920000	-1.159364000
O	2.655194000	-1.296135000	-2.889343000
P	2.443875000	0.241465000	0.173408000
C	2.810121000	-0.558449000	1.787550000
C	2.880484000	-1.956178000	1.845144000
C	3.038568000	0.189613000	2.948311000
C	3.165174000	-2.592057000	3.049207000
H	2.729326000	-2.542816000	0.936151000
C	3.320536000	-0.447530000	4.152929000
H	2.996060000	1.280025000	2.916993000
C	3.378089000	-1.839809000	4.204444000
H	3.224146000	-3.681212000	3.090380000
H	3.501955000	0.139324000	5.055237000
C	1.909328000	1.913235000	0.710898000
C	0.702285000	2.007538000	1.421827000

C	2.602085000	3.080602000	0.383583000
C	0.199451000	3.245506000	1.789551000
H	0.142594000	1.102348000	1.666130000
C	2.082609000	4.328879000	0.731164000
H	3.547569000	3.024374000	-0.159687000
C	0.880407000	4.411527000	1.427025000
H	-0.734528000	3.304637000	2.353019000
H	2.616625000	5.240719000	0.459420000
C	4.107721000	0.514034000	-0.544687000
C	4.173851000	0.971635000	-1.867961000
C	5.288116000	0.189339000	0.130675000
C	5.402161000	1.104376000	-2.503849000
H	3.254279000	1.191598000	-2.414798000
C	6.518580000	0.308079000	-0.510065000
H	5.248620000	-0.172667000	1.159830000
C	6.573756000	0.757980000	-1.828414000
H	5.449847000	1.455602000	-3.536039000
H	7.439115000	0.047199000	0.015210000
C	4.435944000	-2.882625000	-2.713164000
H	4.430194000	-2.987199000	-3.806035000
H	5.270953000	-2.212675000	-2.451106000
H	4.594306000	-3.853604000	-2.228614000
C	0.273051000	5.739047000	1.784250000
C	7.889373000	0.789481000	-2.554838000
C	3.623521000	-2.532318000	5.516732000
F	-0.913482000	5.898444000	1.182701000
F	0.055619000	5.838171000	3.099483000
F	1.046260000	6.763503000	1.423389000
F	7.925539000	1.754965000	-3.476127000
F	8.112920000	-0.368846000	-3.189891000
F	8.914350000	0.984022000	-1.721502000
F	4.360874000	-1.782845000	6.341351000
F	4.257810000	-3.695834000	5.350728000
F	2.474693000	-2.797715000	6.149271000
C	-2.166777000	-3.540105000	-3.606676000
H	-2.986332000	-4.041271000	-4.132283000
C	-1.139325000	-4.279975000	-3.022799000
H	-1.114968000	-5.368543000	-3.068181000
C	-0.155877000	-3.562409000	-2.359770000
H	0.699105000	-4.029712000	-1.862833000
C	-1.239444000	-1.591154000	-2.900747000
N	-2.212542000	-2.215807000	-3.555794000
Br	-0.724401000	-1.218453000	0.833935000
C	-2.568913000	-0.625508000	1.917743000
O	-2.754918000	0.685001000	1.677936000
C	-3.626396000	-1.569307000	1.401321000
H	-2.283109000	-0.788875000	2.961616000
C	-3.082392000	1.042268000	0.333987000
C	-4.013973000	-1.221365000	-0.033997000
H	-3.291567000	-2.613331000	1.471188000
O	-4.770563000	-1.373387000	2.237274000
C	-3.261587000	2.535152000	0.240723000
C	-4.318591000	0.258659000	-0.102597000
H	-2.222477000	0.770548000	-0.306416000
O	-5.172298000	-1.934816000	-0.413480000
H	-3.181186000	-1.477946000	-0.705008000
C	-5.577931000	-2.430522000	2.454849000
H	-2.417385000	3.040453000	0.729617000

H	-3.280892000	2.827110000	-0.817086000
O	-4.461552000	2.949401000	0.875063000
O	-4.654470000	0.624023000	-1.423100000
H	-5.158768000	0.493251000	0.565962000
C	-4.984704000	-3.173509000	-0.918024000
O	-5.335961000	-3.542690000	2.068483000
C	-6.800846000	-2.009756000	3.207436000
C	-5.376237000	3.587099000	0.120720000
C	-5.933937000	0.976355000	-1.672597000
O	-3.896572000	-3.635005000	-1.132699000
C	-6.292211000	-3.867878000	-1.119877000
H	-7.454815000	-1.454386000	2.517166000
H	-6.538214000	-1.331032000	4.029617000
H	-7.329548000	-2.893485000	3.580975000
O	-5.201118000	3.871851000	-1.036604000
C	-6.634405000	3.835250000	0.888076000
O	-6.796793000	0.973510000	-0.835172000
C	-6.094970000	1.416905000	-3.090521000
H	-6.170383000	-4.692961000	-1.830487000
H	-7.067358000	-3.165633000	-1.452320000
H	-6.593735000	-4.276289000	-0.142475000
H	-7.148319000	4.713426000	0.480228000
H	-7.275779000	2.952379000	0.737201000
H	-6.436877000	3.949974000	1.960650000
H	-5.765168000	2.466839000	-3.135119000
H	-5.467543000	0.823151000	-3.766945000
H	-7.151073000	1.362683000	-3.377200000

int3^{p}
Lowest frequency = 8.2936 cm^{-1}
Charge = 0, Multiplicity = 1

71

Ru	1.544893000	-0.734130000	-0.593997000
C	4.195042000	0.191516000	0.022288000
C	2.838916000	0.444831000	0.357762000
C	2.595788000	1.406517000	1.347521000
C	4.968724000	1.871152000	1.564365000
C	3.644684000	2.110391000	1.939828000
H	1.577038000	1.632072000	1.661400000
H	5.781746000	2.433996000	2.028200000
H	3.422556000	2.861814000	2.702171000
N	3.314444000	-1.517875000	-1.339164000
C	5.246117000	0.903085000	0.605523000
H	6.268917000	0.673376000	0.299060000
C	0.910635000	-3.047915000	0.321408000
O	0.532782000	-2.787183000	-0.850661000
O	1.602531000	-2.177830000	0.934651000
P	-0.493764000	0.129221000	0.096663000
C	-1.819742000	0.011372000	-1.164194000
C	-1.862899000	-1.116780000	-1.994344000
C	-2.796497000	1.002742000	-1.295813000
C	-2.875103000	-1.248090000	-2.937618000
H	-1.105224000	-1.896415000	-1.891874000
C	-3.805973000	0.872596000	-2.245970000
H	-2.773559000	1.887553000	-0.657126000

C	-3.844454000	-0.252096000	-3.067307000
H	-2.911730000	-2.130752000	-3.578855000
H	-4.568829000	1.646573000	-2.345533000
C	-0.605801000	1.879844000	0.622295000
C	-0.008643000	2.850950000	-0.193605000
C	-1.256703000	2.267829000	1.797085000
C	-0.058305000	4.190749000	0.169311000
H	0.505924000	2.546346000	-1.109786000
C	-1.298546000	3.611415000	2.163629000
H	-1.733209000	1.520577000	2.434749000
C	-0.695597000	4.570703000	1.352668000
H	0.405191000	4.946033000	-0.468091000
H	-1.807546000	3.914980000	3.080094000
C	-1.176050000	-0.805781000	1.516675000
C	-0.367632000	-0.951761000	2.653147000
C	-2.412496000	-1.455716000	1.464992000
C	-0.790213000	-1.736219000	3.718528000
H	0.612918000	-0.472578000	2.691930000
C	-2.831639000	-2.253780000	2.527087000
H	-3.050479000	-1.349569000	0.585654000
C	-2.016846000	-2.400326000	3.647864000
H	-0.157078000	-1.851114000	4.600116000
H	-3.794370000	-2.766038000	2.482442000
C	0.527420000	-4.311696000	1.019621000
H	1.376917000	-4.701469000	1.595817000
H	-0.278518000	-4.084067000	1.735683000
H	0.168426000	-5.057611000	0.300996000
C	-0.683355000	6.015117000	1.774174000
C	-2.413699000	-3.346385000	4.746693000
C	-4.903164000	-0.378755000	-4.128555000
F	0.441403000	6.316039000	2.433364000
F	-0.754031000	6.834725000	0.723082000
F	-1.704522000	6.303667000	2.584786000
F	-1.964903000	-2.942089000	5.936676000
F	-1.904831000	-4.567176000	4.528146000
F	-3.738040000	-3.482550000	4.834555000
F	-5.991874000	0.335761000	-3.831069000
F	-5.284963000	-1.649336000	-4.290336000
F	-4.459618000	0.051293000	-5.313853000
C	5.760988000	-2.186380000	-2.235495000
H	6.772349000	-2.431371000	-2.579197000
C	4.657197000	-2.898363000	-2.705758000
H	4.757045000	-3.712009000	-3.424260000
C	3.416049000	-2.516195000	-2.215514000
H	2.479861000	-2.999890000	-2.506721000
C	4.435347000	-0.876096000	-0.938910000
N	5.654080000	-1.191603000	-1.364618000
Br	1.366622000	0.546445000	-2.697167000

TS4$^{tri\text{-}p}$

Lowest frequency = -468.0685 cm^{-1}

Charge = 0, Multiplicity = 1

113

Ru	-1.819058000	-1.785224000	0.311287000
C	0.532494000	-2.706754000	1.686748000

C	-0.303874000	-1.557688000	1.609147000
C	0.019515000	-0.463864000	2.428536000
C	2.233241000	-1.405387000	2.789342000
C	1.242549000	-0.409614000	3.079884000
H	-0.671681000	0.375055000	2.531506000
H	3.168756000	-1.383515000	3.356704000
H	1.503103000	0.455724000	3.692966000
N	-1.180860000	-3.759912000	0.449263000
C	1.764627000	-2.669303000	2.323840000
H	2.385704000	-3.567023000	2.360330000
C	-4.189415000	-2.417550000	1.047365000
O	-3.957759000	-2.453060000	-0.190288000
O	-3.231599000	-2.119500000	1.825747000
P	-2.457025000	0.417839000	-0.017699000
C	-2.973306000	0.760139000	-1.742579000
C	-3.733103000	-0.207466000	-2.414640000
C	-2.638344000	1.950179000	-2.393331000
C	-4.155822000	0.022376000	-3.718070000
H	-3.996069000	-1.139975000	-1.909609000
C	-3.056423000	2.175243000	-3.702614000
H	-2.043680000	2.709263000	-1.882194000
C	-3.813945000	1.211822000	-4.364578000
H	-4.753834000	-0.728262000	-4.238295000
H	-2.795672000	3.105902000	-4.209309000
C	-1.278863000	1.778812000	0.326551000
C	0.048822000	1.647445000	-0.103252000
C	-1.683865000	2.956060000	0.963452000
C	0.957347000	2.681115000	0.094668000
H	0.366297000	0.730265000	-0.605705000
C	-0.775386000	3.993327000	1.161251000
H	-2.715622000	3.073357000	1.300812000
C	0.541253000	3.856429000	0.724723000
H	1.995058000	2.562037000	-0.222263000
H	-1.095044000	4.916371000	1.647411000
C	-3.939068000	0.859474000	0.965769000
C	-3.890165000	0.656862000	2.352459000
C	-5.130530000	1.294492000	0.378144000
C	-5.014873000	0.881995000	3.135552000
H	-2.974934000	0.289554000	2.820406000
C	-6.263454000	1.507926000	1.160241000
H	-5.181182000	1.458709000	-0.699747000
C	-6.207296000	1.292866000	2.535691000
H	-4.973756000	0.719388000	4.214070000
H	-7.194600000	1.840910000	0.698413000
C	-5.551934000	-2.660745000	1.608349000
H	-5.483158000	-3.218578000	2.551313000
H	-6.015399000	-1.686633000	1.833303000
H	-6.178804000	-3.193424000	0.883603000
C	1.530320000	4.967501000	0.948846000
C	-7.452891000	1.414869000	3.368569000
C	-4.229608000	1.424803000	-5.794531000
F	2.356932000	4.693086000	1.969474000
F	2.294587000	5.172823000	-0.127527000
F	0.927100000	6.122108000	1.238941000
F	-7.183025000	1.833060000	4.606791000
F	-8.067022000	0.228425000	3.479126000
F	-8.331125000	2.264181000	2.832811000
F	-4.260406000	2.720965000	-6.115270000

F	-5.444378000	0.920242000	-6.031525000
F	-3.385363000	0.827549000	-6.641742000
C	0.097088000	-6.127196000	0.546033000
H	0.649352000	-7.072200000	0.598262000
C	-1.135470000	-6.055961000	-0.104551000
H	-1.590550000	-6.926127000	-0.577812000
C	-1.752731000	-4.813288000	-0.133133000
H	-2.713058000	-4.631064000	-0.623503000
C	0.013917000	-3.919138000	1.066200000
N	0.662515000	-5.076372000	1.124226000
Br	-0.368318000	-1.625294000	-1.696327000
C	2.843240000	-0.604479000	0.958471000
O	3.187434000	0.700493000	1.132665000
C	3.817686000	-1.482617000	0.238523000
H	1.836137000	-0.670412000	0.536808000
C	4.511414000	0.966784000	1.544179000
C	5.261390000	-1.190121000	0.631181000
H	3.584795000	-2.543753000	0.408535000
O	3.650351000	-1.201070000	-1.158755000
C	4.677594000	2.461114000	1.651637000
C	5.486924000	0.304036000	0.573154000
H	4.675056000	0.545006000	2.556178000
O	6.151506000	-1.835449000	-0.252091000
H	5.433555000	-1.561813000	1.654091000
C	3.693701000	-2.234297000	-2.018295000
H	3.862718000	2.869245000	2.265078000
H	5.643367000	2.706177000	2.112801000
O	4.599252000	3.055390000	0.367580000
O	6.807906000	0.625098000	0.958965000
H	5.301881000	0.669487000	-0.446659000
C	6.455245000	-3.120211000	0.044871000
O	3.964797000	-3.360113000	-1.690641000
C	3.337828000	-1.784350000	-3.400209000
C	5.633533000	3.822457000	-0.034596000
C	7.607046000	1.192447000	0.025669000
O	6.108316000	-3.652636000	1.063924000
C	7.224621000	-3.757372000	-1.065668000
H	3.919819000	-0.892956000	-3.671955000
H	2.273010000	-1.504867000	-3.412370000
H	3.515655000	-2.597194000	-4.112426000
O	6.586321000	4.060635000	0.660882000
C	5.447798000	4.273821000	-1.445842000
O	7.261621000	1.391377000	-1.107647000
C	8.921797000	1.586097000	0.615017000
H	7.778735000	-4.622990000	-0.685666000
H	7.893093000	-3.036256000	-1.552851000
H	6.486270000	-4.099697000	-1.807743000
H	6.084175000	5.144228000	-1.640544000
H	5.770239000	3.444992000	-2.095554000
H	4.393985000	4.497164000	-1.652700000
H	8.794001000	2.602876000	1.019668000
H	9.217135000	0.915438000	1.431005000
H	9.684022000	1.617333000	-0.171939000

int5^{p}

Lowest frequency = 6.6064 cm^{-1}

Charge = 0, Multiplicity = 1

113

Ru	1.818109000	-1.784596000	-0.471017000
C	-0.521486000	-2.574784000	-1.780120000
C	0.414603000	-1.447786000	-1.638280000
C	0.072162000	-0.286984000	-2.441676000
C	-2.292796000	-0.993107000	-2.368166000
C	-1.201523000	-0.076149000	-2.829990000
H	0.842339000	0.449986000	-2.675190000
H	-3.153057000	-0.978651000	-3.056112000
H	-1.479541000	0.837300000	-3.361124000
N	1.205131000	-3.759621000	-0.732010000
C	-1.804038000	-2.382797000	-2.152600000
H	-2.505058000	-3.223199000	-2.157393000
C	4.247693000	-2.440924000	-1.052352000
O	3.939719000	-2.578907000	0.156256000
O	3.352306000	-2.059500000	-1.871262000
P	2.451053000	0.387935000	-0.017197000
C	2.966468000	0.661528000	1.723156000
C	3.670888000	-0.355096000	2.380851000
C	2.702374000	1.861613000	2.390001000
C	4.105911000	-0.166629000	3.687507000
H	3.880490000	-1.294563000	1.864207000
C	3.133942000	2.046531000	3.700729000
H	2.154448000	2.661635000	1.888797000
C	3.834190000	1.031470000	4.349814000
H	4.658578000	-0.957879000	4.197321000
H	2.929690000	2.985246000	4.218621000
C	1.240531000	1.740888000	-0.270267000
C	-0.026741000	1.600455000	0.311404000
C	1.543738000	2.896197000	-0.995975000
C	-0.980943000	2.601538000	0.167404000
H	-0.255609000	0.698392000	0.888460000
C	0.590075000	3.901332000	-1.137973000
H	2.531039000	3.021849000	-1.445106000
C	-0.668838000	3.754149000	-0.556634000
H	-1.975381000	2.476931000	0.598228000
H	0.831586000	4.810848000	-1.691162000
C	3.929510000	0.932148000	-0.959565000
C	3.949829000	0.713038000	-2.344130000
C	5.055247000	1.483442000	-0.339360000
C	5.072824000	1.040797000	-3.093382000
H	3.093745000	0.248064000	-2.834909000
C	6.187341000	1.800918000	-1.086306000
H	5.055406000	1.657996000	0.738111000
C	6.198012000	1.573319000	-2.460972000
H	5.083621000	0.866365000	-4.170847000
H	7.065655000	2.226449000	-0.597774000
C	5.642588000	-2.650988000	-1.550681000
H	5.630912000	-3.117740000	-2.544254000
H	6.123475000	-1.664689000	-1.654088000
H	6.220824000	-3.254623000	-0.841199000
C	-1.699456000	4.833460000	-0.739874000
C	7.447052000	1.825428000	-3.259012000
C	4.257720000	1.205148000	5.782397000
F	-2.438521000	4.625272000	-1.842650000
F	-2.541900000	4.901326000	0.291306000

F	-1.135347000	6.036140000	-0.881482000
F	7.167313000	2.229852000	-4.500527000
F	8.179491000	0.708968000	-3.365610000
F	8.222134000	2.752808000	-2.693562000
F	4.422723000	2.493278000	6.097897000
F	5.409720000	0.576739000	6.034782000
F	3.346666000	0.707309000	6.625144000
C	-0.086183000	-6.104380000	-1.028838000
H	-0.641191000	-7.039215000	-1.162992000
C	1.153273000	-6.093512000	-0.389294000
H	1.608359000	-7.005409000	-0.002083000
C	1.780682000	-4.861817000	-0.252365000
H	2.744063000	-4.723571000	0.245943000
C	0.003256000	-3.865068000	-1.335480000
N	-0.657281000	-5.000270000	-1.498057000
Br	0.428727000	-1.812911000	1.634626000
C	-2.735372000	-0.432777000	-0.968409000
O	-3.234894000	0.871308000	-1.070554000
C	-3.652471000	-1.334706000	-0.154452000
H	-1.807230000	-0.356482000	-0.384608000
C	-4.564002000	1.002489000	-1.517428000
C	-5.102009000	-1.219043000	-0.603309000
H	-3.328586000	-2.382592000	-0.206639000
O	-3.528509000	-0.889911000	1.190972000
C	-4.888975000	2.473284000	-1.612903000
C	-5.500086000	0.238506000	-0.582093000
H	-4.674369000	0.587039000	-2.540728000
O	-5.947502000	-1.953222000	0.254928000
H	-5.191202000	-1.622518000	-1.625596000
C	-3.497259000	-1.830979000	2.156781000
H	-4.093012000	2.978602000	-2.176241000
H	-5.851889000	2.619078000	-2.119978000
O	-4.942171000	3.048721000	-0.319723000
O	-6.830933000	0.399998000	-1.030484000
H	-5.404729000	0.632973000	0.438465000
C	-5.991543000	-3.285386000	0.030264000
O	-3.705042000	-2.996546000	1.948082000
C	-3.139918000	-1.222808000	3.474133000
C	-6.076733000	3.678303000	0.042112000
C	-7.737085000	0.852089000	-0.132370000
O	-5.453397000	-3.802010000	-0.913037000
C	-6.745736000	-3.988897000	1.109437000
H	-3.688293000	-0.284300000	3.630337000
H	-2.063518000	-0.988325000	3.456834000
H	-3.343599000	-1.936096000	4.279941000
O	-7.021719000	3.813310000	-0.691752000
C	-6.008273000	4.126591000	1.465393000
O	-7.474174000	1.068649000	1.019585000
C	-9.058205000	1.099763000	-0.783580000
H	-7.092271000	-4.963317000	0.747432000
H	-7.579828000	-3.377608000	1.476489000
H	-6.038893000	-4.142035000	1.939995000
H	-6.725714000	4.937986000	1.631901000
H	-6.297212000	3.265629000	2.088728000
H	-4.989464000	4.432307000	1.733838000
H	-9.029557000	2.131103000	-1.170110000
H	-9.232531000	0.413219000	-1.620930000
H	-9.856876000	1.028975000	-0.036354000

mannosyl bromide
Lowest frequency = 29.8961 cm^{-1}
Charge = 0, Multiplicity = 1

43

Br	-2.361627000	-2.662130000	-0.615894000
C	-1.410304000	-1.617342000	0.854683000
O	-0.104693000	-1.963723000	0.943101000
C	0.717285000	-1.584511000	-0.151442000
C	2.117623000	-2.089519000	0.100729000
H	2.058132000	-3.130348000	0.450328000
H	2.703163000	-2.045143000	-0.827293000
O	2.764433000	-1.322597000	1.102515000
C	3.894582000	-0.672214000	0.760408000
O	4.407802000	-0.762492000	-0.325043000
C	4.377094000	0.198842000	1.874219000
H	5.464059000	0.321455000	1.802000000
H	3.904258000	1.183585000	1.731429000
H	4.083196000	-0.197311000	2.853584000
C	0.645308000	-0.072294000	-0.343349000
C	-0.788917000	0.345443000	-0.586164000
C	-1.652436000	-0.141666000	0.575763000
H	-2.714551000	0.058127000	0.378724000
O	-1.241490000	0.533391000	1.760035000
C	-1.886786000	1.673221000	2.087173000
O	-2.848794000	2.082944000	1.496309000
C	-1.218737000	2.338110000	3.248648000
H	-0.288516000	2.804462000	2.888306000
H	-0.943301000	1.601778000	4.015074000
H	-1.876414000	3.110164000	3.662635000
O	-0.828728000	1.754348000	-0.657340000
C	-1.834171000	2.298206000	-1.387081000
O	-2.596605000	1.637932000	-2.035180000
C	-1.858655000	3.783361000	-1.226386000
H	-2.416537000	4.237174000	-2.052994000
H	-0.841957000	4.191373000	-1.158502000
H	-2.378802000	3.997426000	-0.279448000
H	-1.172953000	-0.086243000	-1.523160000
O	1.448444000	0.279818000	-1.451585000
C	2.342796000	1.280632000	-1.282674000
O	2.462040000	1.892206000	-0.254798000
C	3.177675000	1.461733000	-2.507198000
H	4.006628000	0.739912000	-2.433866000
H	2.606604000	1.247764000	-3.418962000
H	3.592647000	2.475817000	-2.524751000
H	1.022012000	0.430248000	0.558174000
H	0.336980000	-2.088867000	-1.060229000
H	-1.921126000	-1.943452000	1.767167000

mannosyl radical
Lowest frequency = 27.6843 cm^{-1}
Charge = 0, Multiplicity = 2

42

C	-0.835076000	-2.320658000	-1.543857000
O	0.512843000	-2.258731000	-1.496917000
C	1.114103000	-0.978637000	-1.593968000
C	2.595046000	-1.120453000	-1.326519000
H	2.967636000	-2.000636000	-1.870355000
H	3.126698000	-0.222518000	-1.668685000
O	2.845683000	-1.317452000	0.054966000
C	3.604716000	-0.403107000	0.690395000
O	4.132217000	0.524164000	0.131651000
C	3.651572000	-0.665061000	2.160591000
H	4.587220000	-0.272634000	2.575705000
H	2.811214000	-0.109113000	2.606157000
H	3.529672000	-1.731408000	2.385309000
C	0.429744000	0.011713000	-0.655877000
C	-1.042133000	0.085237000	-0.996644000
C	-1.642822000	-1.307949000	-0.823403000
H	-2.688710000	-1.317929000	-1.161687000
O	-1.618815000	-1.615322000	0.591229000
C	-2.701838000	-1.301470000	1.319180000
O	-3.725733000	-0.875512000	0.850275000
C	-2.443256000	-1.531395000	2.776684000
H	-1.783635000	-0.727448000	3.138847000
H	-1.919321000	-2.483418000	2.934114000
H	-3.388031000	-1.506340000	3.330899000
O	-1.657090000	1.009781000	-0.126228000
C	-2.773193000	1.623727000	-0.586832000
O	-3.167999000	1.497515000	-1.712836000
C	-3.425950000	2.418466000	0.496851000
H	-4.089177000	3.172056000	0.057770000
H	-2.679214000	2.878355000	1.156806000
H	-4.025376000	1.713288000	1.093958000
H	-1.186993000	0.410615000	-2.038701000
O	1.040522000	1.275158000	-0.825669000
C	1.423098000	1.938456000	0.288754000
O	1.249672000	1.516872000	1.400928000
C	2.132025000	3.205254000	-0.062008000
H	3.185718000	2.938460000	-0.241090000
H	1.724284000	3.654821000	-0.975755000
H	2.082159000	3.902368000	0.782056000
H	0.544830000	-0.317617000	0.386023000
H	0.998129000	-0.615504000	-2.633667000
H	-1.203729000	-3.341920000	-1.656988000

AcOH
Lowest frequency = 80.9359 cm^{-1}
Charge = 0, Multiplicity = 1

8

O	0.639350000	1.192097000	-0.000061000
C	0.092582000	0.121760000	0.000154000
C	-1.388318000	-0.109203000	-0.000020000
H	-1.673519000	-0.696492000	-0.885113000
H	-1.673395000	-0.698108000	0.884038000
H	-1.912015000	0.852459000	0.000989000
O	0.775496000	-1.034611000	-0.000058000

H	1.714579000	-0.793097000	0.000238000

List of Substrate Scope for *meta*-C–H Glycosylation

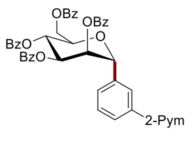

3: 66%

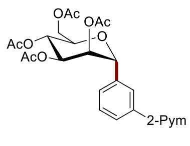

4: 54%

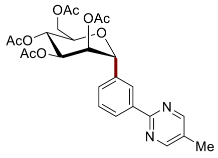

5:80%

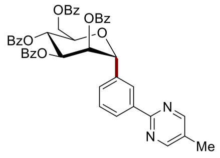

6: 40%

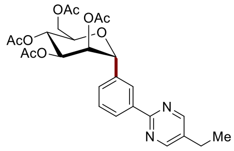

7: 36%

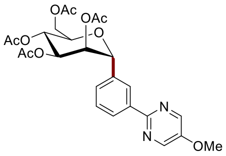

8: 40%

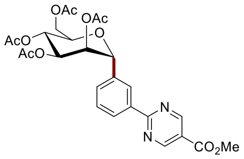

9: 20%

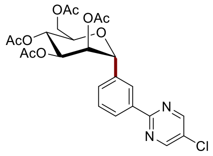

10: 20%

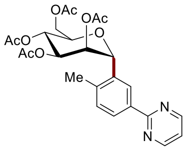

11: 64%

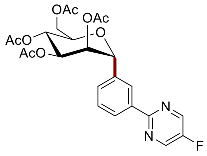

12: 60%

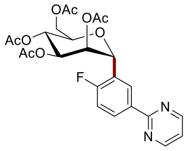

13: 31%

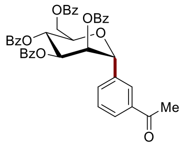

14: 20%*

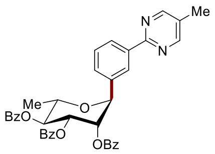

16:47%

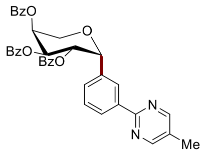

17: 41%

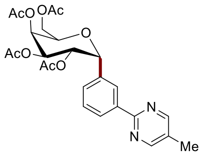

19: 49%

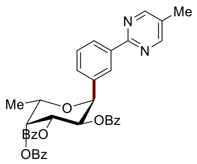

20: 58%

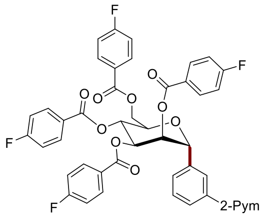

21: 34%
$\alpha/\beta > 20/1$

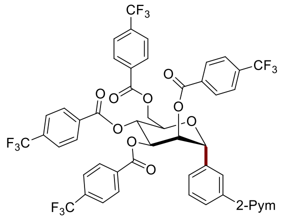

22: 37%
$\alpha/\beta > 20/1$

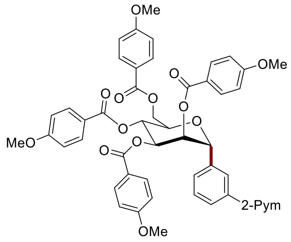

23: 17%
$\alpha/\beta > 20/1$

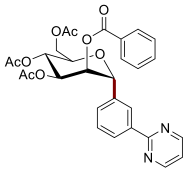

24: 30%

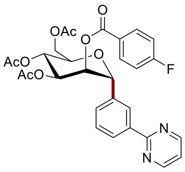

25: 32%

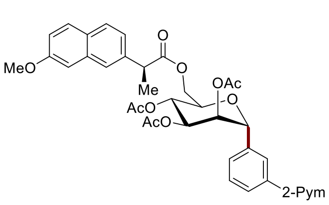

From Naproxen

28: 87%

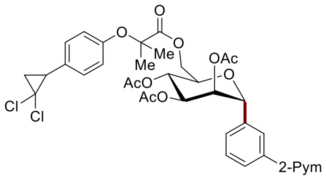

From Ciprofibrate

29: 34%

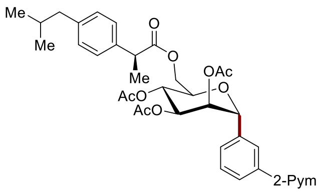

From Ibuprofen

30: 87%

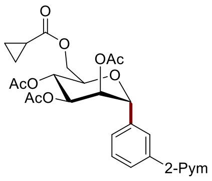

31: 51%

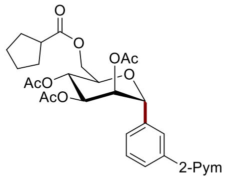

32: 54%

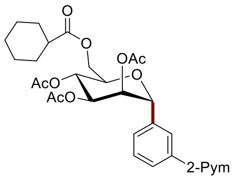

33: 39%

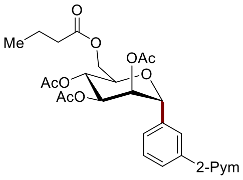

34: 41%

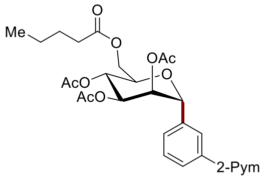

35: 39%

Failed examples:

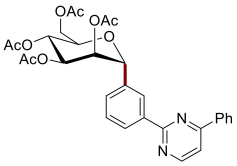

N.D.

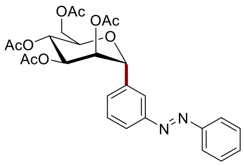

N.D.

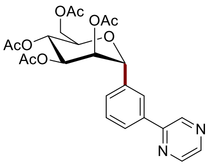

N.D.

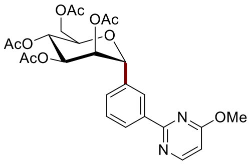

N.D.

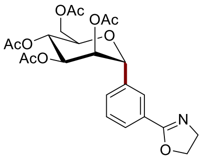

N.D.

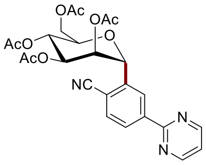

N.D.

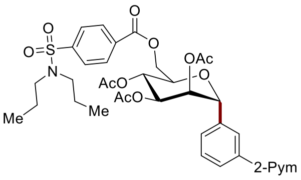

N.D.

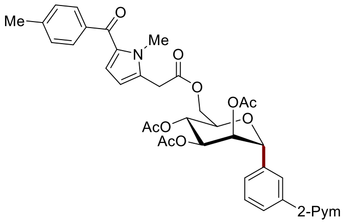

N.D.

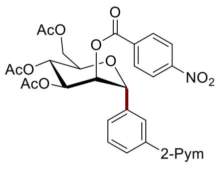

N.D.

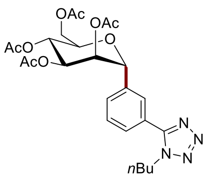

N.D.

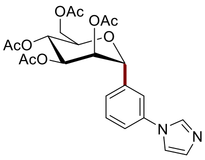

N.D.

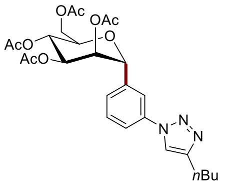

N.D.

Characterization Data of Products

(2*R*,3*R*,4*R*,5*R*,6*R*)-2-((Benzoyloxy)methyl)-6-(3-(pyrimidin-2-yl)phenyl)tetrahydro-2*H*-pyran-3,4,5-triyl-tribenzoate (3)

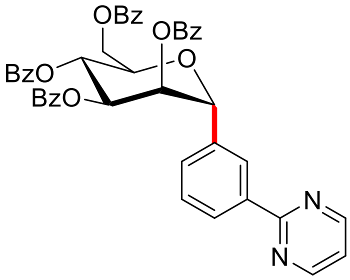

The general procedure was followed using 2-phenylpyrimidine (**1a**) (15.5 mg, 0.10 mmol), (2*R*,3*R*,4*S*,5*S*,6*R*)-2-[(benzoyloxy)methyl]-6-bromotetrahydro-2*H*-pyran-3,4,5-triyltribenzoate (**2a**) (131.6 mg, 0.20 mmol), [RuCl$_2$(*p*-cymene)]$_2$ (3.1 mg, 5.0 mol %), NaOAc (16.4 mg, 0.2 mmol) in 1,4-dioxane (1.0 mL) at ambient temperature. Purification by column chromatography on silica gel (*n*-hexane/EtOAc: 10/1 to 3/1) yielded **3** (48.5 mg, 66%) as a sticky solid.

^{1}H NMR (400 MHz, CDCl$_3$) δ 8.84 (s, 1H), 8.77 (d, *J* = 4.8 Hz, 2H), 8.50 (d, *J* = 7.8 Hz, 1H), 8.17 (d, *J* = 8.3, 2H), 8.11 (d, *J* = 8.3, 2H), 7.97 – 7.89 (m, 4H), 7.88 – 7.85 (m, 1H), 7.65 (t, *J* = 7.8 Hz, 1H), 7.59 (tdd, *J* = 7.4, 4.4, 1.4 Hz, 2H), 7.51 – 7.45 (m, 2H), 7.43 (t, *J* = 7.8 Hz, 4H), 7.32 (td, *J* = 7.8, 2.9 Hz, 4H), 7.20 (t, *J* = 4.8 Hz, 1H), 6.60 (t, *J* = 2.9 Hz, 1H), 6.19 (t, *J* = 9.3 Hz, 1H), 5.73 (dd, *J* = 9.4, 3.1 Hz, 1H), 5.54 (d, *J* = 2.6 Hz, 1H), 4.76 (dd, *J* = 12.1, 2.7 Hz, 1H), 4.60 (dd, *J* = 12.0, 5.7 Hz, 1H), 4.32 (ddd, *J* = 8.8, 5.7, 2.6 Hz, 1H).

^{13}C NMR (101 MHz, CDCl$_3$) δ 166.5 (C$_q$), 166.0 (C$_q$), 165.8 (C$_q$), 165.6 (C$_q$), 164.4(C$_q$), 157.5 (CH), 138.9 (C$_q$), 136.0 (C$_q$), 133.6 (CH), 133.6 (CH), 133.5 (CH), 133.2 (CH), 130.1 (C$_q$), 130.1 (CH), 130.0 (CH), 130.0 (CH), 130.0 (CH), 130.0 (CH), 130.0 (C$_q$), 129.2 (C$_q$), 129.1 (C$_q$), 128.8 (CH), 128.7 (CH), 128.6 (CH), 128.5 (CH), 126.7 (CH), 119.5 (CH), 76.4 (CH), 71.5 (CH), 71.0 (CH), 70.8 (CH), 67.7 (CH), 63.5 (CH$_2$).

IR (ATR): $\tilde{\nu}$ = 1720, 1601, 1451, 1410, 1265, 1178, 1093, 1070, 1027, 709 cm^{-1}.

MS (ESI) *m/z* (relative intensity): 757 (50) [M+Na]$^+$, 735 (100) [M+H]$^+$.

HR-MS (ESI): m/z calcd for $C_{44}H_{35}N_2O_9^+$ [M+H]$^+$ 735.2337, found 735.2315.

(2*R*,3*R*,4*R*,5*R*,6*R*)-2-(Acetoxymethyl)-6-(3-(pyrimidin-2-yl)phenyl)tetrahydro-2*H*-pyran-3,4,5-triyl-triacetate (4)

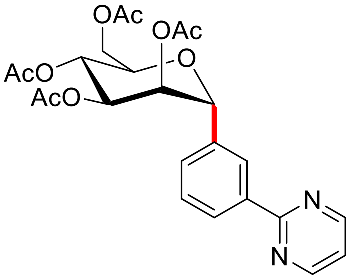

The general procedure was followed using 2-phenylpyrimidine (**1a**) (15.5 mg, 0.10 mmol), (2*R*,3*R*,4*S*,5*S*,6*R*)-2-(acetoxymethyl)-6-bromotetrahydro-2*H*-pyran-3,4,5-triyl-triacetate (**2b**) (82.0 mg, 0.20 mmol), [RuCl$_2$(*p*-cymene)]$_2$ (3.1 mg, 5.0 mol %), NaOAc (16.4 mg, 0.2 mmol) in 1,4-dioxane (1.0 mL) at ambient temperature. Purification by column chromatography on silica gel (*n*-hexane/EtOAc: 5/1 to 2/1) yielded **4** (26.3 mg, 54%) as a sticky solid.

^{1}H NMR (400 MHz, CDCl$_3$) δ 8.82 (d, *J* = 4.8 Hz, 2H), 8.62 (d, *J* = 2.1 Hz, 1H), 8.44 (d, *J* = 7.8 Hz, 1H), 7.68 – 7.60 (m, 1H), 7.56 (t, *J* = 7.7 Hz, 1H), 7.21 (t, *J* = 4.8 Hz, 1H), 6.04 (t, *J* = 3.3 Hz, 1H), 5.33 (t, *J* = 8.5 Hz, 1H), 5.24 – 5.18 (m, 2H), 4.41 (dd, *J* = 12.0, 6.8 Hz, 1H), 4.18 (dd, *J* = 12.1, 2.8 Hz, 1H), 3.88 (ddd, *J* = 8.2, 6.7, 2.9 Hz, 1H), 2.16 (s, 3H), 2.15 (s, 3H), 2.08 (s, 3H), 2.02 (s, 3H).

^{13}C NMR (101 MHz, CDCl$_3$) δ 170.9 (C$_q$), 170.4 (C$_q$), 170.3 (C$_q$), 169.8 (C$_q$), 164.4 (C$_q$), 157.4 (CH), 138.6 (C$_q$), 136.0 (C$_q$), 129.6 (CH), 128.7 (CH), 128.4 (CH), 126.7 (CH), 119.5 (CH), 75.6 (CH), 71.7 (CH), 69.7 (CH), 69.5 (CH), 67.2 (CH), 62.5 (CH$_2$), 21.1 (CH$_3$), 20.9 (CH$_3$), 20.9 (CH$_3$), 20.9 (CH$_3$).

IR (ATR): $\tilde{\nu}$ = 1737, 1556, 1411, 1367, 1211, 1048, 1020, 1051, 731, 700 cm^{-1}.

MS (ESI) *m/z* (relative intensity): 509 (100) [M+Na]$^+$, 487 (70) [M+H]$^+$.

HR-MS (ESI): m/z calcd for $C_{24}H_{27}N_2O_9^+$ [M+H]$^+$ 487.1711, found 487.1712.

(2*R*,3*R*,4*R*,5*R*,6*R*)-2-(Acetoxymethyl)-6-(3-(5-methylpyrimidin-2-yl)phenyl)tetrahydro-2*H*-pyran-3,4,5-triyl-triacetate (5)

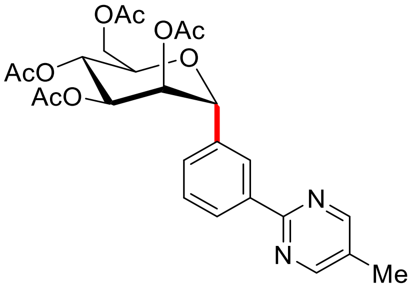

The general procedure was followed using 5-methyl-2-phenylpyrimidine (**1b**) (17.0 mg, 0.10 mmol), (2*R*,3*R*,4*S*,5*S*,6*R*)-2-(acetoxymethyl)-6-bromotetrahydro-2*H*-pyran-3,4,5-triyl-triacetate (**2b**) (82.0 mg, 0.20 mmol), [RuCl$_2$(*p*-cymene)]$_2$ (3.1 mg, 5.0 mol %), NaOAc (16.4 mg, 0.2 mmol) in 1,4-dioxane (1.0 mL) at ambient temperature. Purification by column chromatography on silica gel (*n*-hexane/EtOAc: 5/1 to 2/1) yielded **5** (40.0 mg, 80%) as a sticky solid.

^{1}H NMR (400 MHz, CDCl$_3$) δ 8.64 (d, *J* = 0.8 Hz, 2H), 8.61 – 8.54 (m, 1H), 8.39 (d, *J* = 7.7, 1H), 7.65 – 7.56 (m, 1H), 7.54 (t, *J* = 7.7 Hz, 1H), 6.04 (t, *J* = 3.3 Hz, 1H), 5.33 (t, *J* = 8.5 Hz, 1H), 5.28 – 5.11 (m, 2H), 4.40 (dd, *J* = 12.0, 6.7 Hz, 1H), 4.18 (dd, *J* = 12.0, 2.8 Hz, 1H), 3.87 (ddd, *J* = 8.3, 6.7, 2.8 Hz, 1H), 2.35 (s, 3H), 2.16 (s, 3H), 2.15 (s, 3H), 2.07 (s, 3H), 2.01 (s, 3H).

^{13}C NMR (101 MHz, CDCl$_3$) δ 170.9 (C$_q$), 170.4 (C$_q$), 170.3 (C$_q$), 169.8 (C$_q$), 162.1 (C$_q$), 157.6 (CH), 138.7 (C$_q$), 135.9 (C$_q$), 129.5 (CH), 128.8 (C$_q$), 128.3 (CH), 128.1 (CH), 126.4 (CH), 75.6 (CH), 71.6 (CH), 69.7 (CH), 69.6 (CH), 67.2 (CH), 62.6 (CH$_2$), 21.1 (CH$_3$), 20.9 (CH$_3$), 20.9 (CH$_3$), 20.9 (CH$_3$), 15.7 (CH$_3$).

IR (ATR): $\tilde{\nu}$ = 1737, 1548, 1213, 1047, 732, 654 cm^{-1}.

MS (ESI) *m/z* (relative intensity): 523 (100) [M+Na]$^+$, 501 (8) [M+H]$^+$.

HR-MS (ESI): m/z calcd for $C_{25}H_{29}N_2O_9^+$ [M+H]$^+$ 501.1868, found 501.1879.

(2*R*,3*R*,4*R*,5*R*,6*R*)-2-((Benzoyloxy)methyl)-6-(3-(5-methylpyrimidin-2-yl)phenyl)tetrahydro-2H-pyran-3,4,5-triyl-tribenzoate (6)

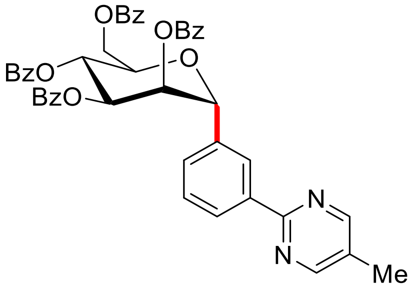

The general procedure was followed using 5-methyl-2-phenylpyrimidine (**1b**) (17.0 mg, 0.10 mmol), (2*R*,3*R*,4*S*,5*S*,6*R*)-2-[(benzoyloxy)methyl]-6-bromotetrahydro-2*H*-pyran-3,4,5-triyl-tribenzoate (**2a**) (131.6 mg, 0.20 mmol), $[RuCl_2(p\text{-cymene})]_2$ (3.1 mg, 5.0 mol %), NaOAc (16.4 mg, 0.2 mmol) in 1,4-dioxane (1.0 mL) at ambient temperature. Purification by column chromatography on silica gel (*n*-hexane/EtOAc: 10/1 to 3/1) yielded **6** (29.9 mg, 40%) as a sticky solid.

^{1}H NMR (400 MHz, $CDCl_3$) δ 8.79 (s, 1H), 8.58 (s, 2H), 8.45 (d, *J* = 7.8 Hz, 1H), 8.20 – 8.13 (m, 2H), 8.14 – 8.08 (m, 2H), 7.91 (ddd, *J* = 8.4, 3.1, 1.3 Hz, 4H), 7.83 (d, *J* = 7.4 Hz, 1H), 7.64 (d, *J* = 7.7 Hz, 1H), 7.64 – 7.55 (m, 2H), 7.47 (td, J = 7.3, 1.4 Hz, 2H), 7.42 (t, *J* = 7.7 Hz, 4H), 7.32 (td, *J* = 7.7, 3.5 Hz, 4H), 6.60 (t, *J* = 2.8 Hz, 1H), 6.18 (t, *J* = 9.4 Hz, 1H), 5.73 (dd, *J* = 9.6, 3.1 Hz, 1H), 5.53 (d, *J* = 2.5 Hz, 1H), 4.75 (dd, *J* = 12.1, 2.5 Hz, 1H), 4.59 (dd, *J* = 12.0, 5.6 Hz, 1H), 4.30 (ddd, *J* = 8.8, 5.6, 2.5 Hz, 1H), 2.35 (s, 3H).

^{13}C NMR (101 MHz, $CDCl_3$) δ 166.4 (C_q), 166.0 (C_q), 165.8 (C_q), 165.6 (C_q), 162.1 (C_q), 157.6 (CH), 139.0 (C_q), 135.9 (C_q), 133.6 (CH), 133.5 (CH), 133.4 (CH), 133.1 (CH), 130.1 (C_q), 130.1 (CH), 130.1 (CH), 130.0 (CH), 130.0 (CH), 129.7 (C_q), 129.2 (C_q), 129.1 (C_q), 128.7 (C_q), 128.6 (CH), 128.3 (CH), 128.2 (CH), 126.5 (CH), 76.5 (CH), 71.4 (CH), 71.0 (CH), 70.8 (CH), 67.7 (CH), 63.5 (CH_2), 15.7 (CH_3).

IR (ATR): $\tilde{\nu}$ = 1721, 1451, 1423, 1265, 1110, 1093, 906, 734, 709, 651 cm^{-1}.

MS (ESI) *m/z* (relative intensity): 771 (100) $[M+Na]^+$, 749 (10) $[M+H]^+$.

HR-MS (ESI): m/z calcd for $C_{45}H_{37}N_2O_9^+$ $[M+H]^+$ 749.25, found 749.26.

(2*R*,3*R*,4*R*,5*R*,6*R*)-2-(Acetoxymethyl)-6-(3-(5-ethylpyrimidin-2-yl)phenyl)tetrahydro-2*H*-pyran-3,4,5-triyl-triacetate (7)

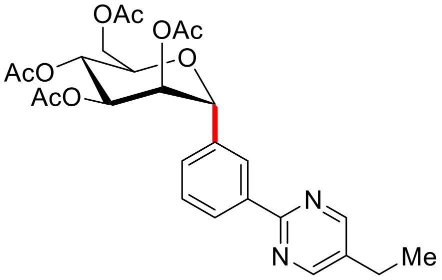

The general procedure was followed using 5-ethyl-2-phenylpyrimidine (**1c**) (18.4 mg, 0.10 mmol), (2*R*,3*R*,4*S*,5*S*,6*R*)-2-(acetoxymethyl)-6-bromotetrahydro-2*H*-pyran-3,4,5-triyl-triacetate (**2b**) (82.0 mg, 0.20 mmol), $[RuCl_2(p\text{-cymene})]_2$ (3.1 mg, 5.0 mol %), NaOAc (16.4 mg, 0.2 mmol) in 1,4-dioxane (1.0 mL) at ambient temperature. Purification by column chromatography on silica gel (*n*-hexane/EtOAc: 5/1 to 2/1) yielded **7** (18.5 mg, 36%) as a sticky solid.

^{1}H NMR (400 MHz, $CDCl_3$) δ 8.66 (s, 2H), 8.59 (d, *J* = 2.1 Hz, 1H), 8.40 (d, *J* = 7.5 Hz, 1H), 7.60 (dd, *J* = 7.7, 2.0 Hz, 1H), 7.55 (t, *J* = 7.7 Hz, 1H), 6.05 (t, *J* = 3.3 Hz, 1H), 5.34 (t, *J* = 8.5 Hz, 1H), 5.27 – 5.15 (m, 2H), 4.40 (dd, *J* = 12.0, 6.7 Hz, 1H), 4.18 (dd, *J* = 12.1, 2.8 Hz, 1H), 3.87 (ddd, *J* = 8.4, 6.7, 2.8 Hz, 1H), 2.69 (q, *J* = 7.6 Hz, 2H), 2.16 (s, 3H), 2.15 (s, 3H), 2.06 (s, 3H), 2.01 (s, 3H), 1.31 (t, *J* = 7.6 Hz, 3H).

^{13}C NMR (101 MHz, $CDCl_3$) δ 170.9 (C_q), 170.4 (C_q), 170.3 (C_q), 169.8 (C_q), 162.3 (C_q), 156.9 (CH), 138.8 (C_q), 135.9 (C_q), 134.7 (C_q), 129.5 (CH), 128.3 (CH), 128.2 (CH), 126.4 (CH), 75.7 (CH), 71.6 (CH), 69.7 (CH), 69.6 (CH), 67.2 (CH), 62.6 (CH_2), 23.6 (CH_2), 21.1 (CH_3), 20.9 (CH_3), 20.9 (CH_3), 20.9 (CH_3), 15.1 (CH_3).

IR (ATR): $\tilde{\nu}$ = 1737, 1423, 1367, 1211, 1048, 913, 733, 654 cm^{-1}.

MS (ESI) *m/z* (relative intensity): 537 (100) $[M+Na]^+$, 515 (58) $[M+H]^+$.

HR-MS (ESI): m/z calcd for $C_{26}H_{31}N_2O_9^+$ $[M+H]^+$ 515.2024, found 515.2044.

(2*R*,3*R*,4*R*,5*R*,6*R*)-2-(Acetoxymethyl)-6-(3-(5-methoxypyrimidin-2-yl)phenyl)tetrahydro-2*H*-pyran-3,4,5-triyl-triacetate (8)

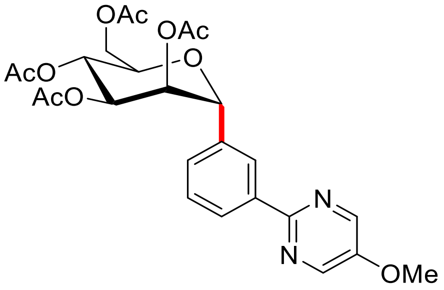

The general procedure was followed using 5-methoxy-2-phenylpyrimidine (**1d**) (18.4 mg, 0.10 mmol), (2*R*,3*R*,4*S*,5*S*,6*R*)-2-(acetoxymethyl)-6-bromotetrahydro-2*H*-pyran-3,4,5-triyl-triacetate (**2b**) (82.0 mg, 0.20 mmol), $[RuCl_2(p\text{-cymene})]_2$ (3.1 mg, 5.0 mol %), NaOAc (16.4 mg, 0.2 mmol) in 1,4-dioxane (1.0 mL) at ambient temperature. Purification by column chromatography on silica gel (*n*-hexane/EtOAc: 5/1 to 3/1) yielded **8** (20.6 mg, 40%) as a sticky solid.

^{1}H NMR (400 MHz, $CDCl_3$) δ 8.53 (t, J = 0.9 Hz, 1H), 8.48 (s, 2H), 8.37 – 8.32 (m, 1H), 7.60 – 7.55 (m, 1H), 7.55 – 7.50 (m, 1H), 6.05 (t, J = 3.2 Hz, 1H), 5.34 (t, J = 8.6 Hz, 1H), 5.25 – 5.10 (m, 2H), 4.40 (dd, J = 12.1, 6.6 Hz, 1H), 4.18 (dd, J = 12.1, 2.8 Hz, 1H), 3.97 (s, 3H), 3.86 (ddd, J = 9.1, 6.7, 2.8 Hz, 1H), 2.16 (s, 3H), 2.15 (s, 3H), 2.08 (s, 3H), 2.01 (s, 3H).

^{13}C NMR (101 MHz, $CDCl_3$) δ 170.9 (C_q), 170.4 (C_q), 170.3 (C_q), 169.8 (C_q), 157.5 (C_q), 152.3 (C_q), 143.6 (CH), 138.5 (C_q), 135.9 (C_q), 129.5 (CH), 127.8 (CH), 127.8 (CH), 126.1 (CH), 75.7 (CH), 71.5 (CH), 69.8 (CH), 69.6 (CH), 67.2 (CH), 62.6 (CH_2), 56.2 (CH_3), 21.2 (CH_3), 20.9 (CH_3), 20.9 (CH_3), 20.9 (CH_3).

IR (ATR): $\tilde{\nu}$ = 1744, 1454, 1424, 1368, 1278, 1224, 1049, 1014, 912, 796 cm^{-1}.

MS (ESI) *m/z* (relative intensity): 539 (100) $[M+Na]^+$, 749 (38) $[M+H]^+$.

HR-MS (ESI): m/z calcd for $C_{25}H_{29}N_2O_{10}^+$ $[M+H]^+$ 517.1817, found 517.1836.

(2*R*,3*R*,4*R*,5*R*,6*R*)-2-(Acetoxymethyl)-6-(3-(5-(methoxycarbonyl)pyrimidin-2-yl)phenyl)tetrahydro-2*H*-pyran-3,4,5-triyl-triacetate (9)

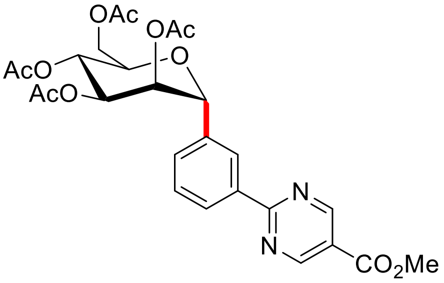

The general procedure was followed using methyl 2-phenylpyrimidine-5-carboxylate (**1e**) (21.4 mg, 0.10 mmol), (2*R*,3*R*,4*S*,5*S*,6*R*)-2-(acetoxymethyl)-6-bromotetrahydro-2*H*-pyran-3,4,5-triyl-triacetate (**2b**) (82.0 mg, 0.20 mmol), $[RuCl_2(p\text{-cymene})]_2$ (3.1 mg, 5.0 mol %), NaOAc (16.4 mg, 0.2 mmol) in 1,4-dioxane (1.0 mL) at ambient temperature. Purification by column chromatography on silica gel (*n*-hexane/EtOAc: 5/1 to 3/1) yielded **9** (10.9 mg, 20%) as a sticky solid.

^{1}H NMR (400 MHz, $CDCl_3$) δ 9.33 (s, 2H), 8.69 (dq, *J* = 1.7, 0.9 Hz, 1H), 8.52 (dq, *J* = 7.9, 1.0 Hz, 1H), 7.75 – 7.64 (m, 1H), 7.59 (t, *J* = 7.8 Hz, 1H), 6.02 (t, *J* = 3.4 Hz, 1H), 5.33 (t, *J* = 8.4 Hz, 1H), 5.22 (d, *J* = 3.2 Hz, 1H), 5.19 (t, *J* = 3.2 Hz, 1H), 4.43 (dd, *J* = 12.0, 6.9 Hz, 1H), 4.18 (dd, *J* = 12.0, 2.9 Hz, 1H), 4.00 (s, 3H), 3.92 – 3.80 (m, 1H), 2.16 (s, 3H), 2.15 (s, 3H), 2.09 (s, 3H), 2.02 (s, 3H).

^{13}C NMR (101 MHz, $CDCl_3$) δ 170.9 (C_q), 170.4 (C_q), 170.3 (C_q), 169.8 (C_q), 166.9 (C_q), 164.5 (C_q), 158.6 (CH), 137.6 (C_q), 136.3 (C_q), 129.8 (CH), 129.7 (CH), 129.3 (CH), 127.5 (CH), 121.9 (C_q), 75.3 (CH), 71.8 (CH), 69.6 (CH), 69.5 (CH), 67.2 (CH), 62.5 (CH_2), 52.8 (CH_3), 21.1 (CH_3), 21.0 (CH_3), 20.9 (CH_3), 20.9 (CH_3).

IR (ATR): $\tilde{\nu}$ = 1746, 1585, 1422, 1368, 1222, 914, 772 cm^{-1}.

MS (ESI) *m/z* (relative intensity): 567 (100) $[M+Na]^+$, 545 (40) $[M+H]^+$.

HR-MS (ESI): m/z calcd for $C_{26}H_{29}N_2O_{11}^+$ $[M+H]^+$ 545.1766, found 545.1776.

(2*R*,3*R*,4*R*,5*R*,6*R*)-2-(Acetoxymethyl)-6-(3-(5-chloropyrimidin-2-yl)phenyl)tetrahydro-2*H*-pyran-3,4,5-triyl-triacetate (10)

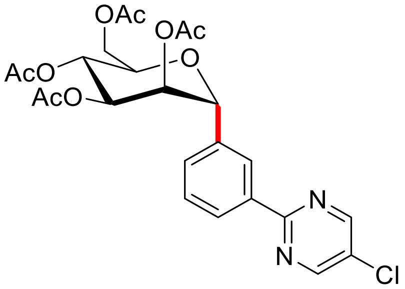

The general procedure was followed using 5-chloro-2-phenylpyrimidine (**1f**) (19.0 mg, 0.10 mmol), (2*R*,3*R*,4*S*,5*S*,6*R*)-2-(acetoxymethyl)-6-bromotetrahydro-2*H*-pyran-3,4,5-triyl-triacetate (**2b**) (82.0 mg, 0.20 mmol), $[RuCl_2(p\text{-cymene})]_2$ (3.1 mg, 5.0 mol %), NaOAc (16.4 mg, 0.2 mmol) in 1,4-dioxane (1.0 mL) at ambient temperature. Purification by column chromatography on silica gel (*n*-hexane/EtOAc: 5/1 to 3/1) yielded **10** (10.4 mg, 20%) as a sticky solid.

^{1}H NMR (400 MHz, $CDCl_3$) δ 8.76 (d, *J* = 0.6 Hz, 2H), 8.58 (t, *J* = 1.8 Hz, 1H), 8.46 – 8.36 (m, 1H), 7.68 – 7.61 (m, 1H), 7.56 (t, *J* = 7.7 Hz, 1H), 6.03 (t, *J* = 3.3 Hz, 1H), 5.34 (t, *J* = 8.5 Hz, 1H), 5.27 – 5.14 (m, 2H), 4.42 (dd, *J* = 12.1, 6.8 Hz, 1H), 4.18 (dd, *J* = 12.1, 2.8 Hz, 1H), 3.97 – 3.79 (m, 1H), 2.16 (s, 3H) ,2.15 (s, 3H), 2.08 (s, 3H), 2.02 (s, 3H).

^{13}C NMR (101 MHz, $CDCl_3$) δ 170.9 (C_q), 170.4 (C_q), 170.3 (C_q), 169.8 (C_q), 162.2 (C_q), 155.9 (CH), 137.5 (C_q), 136.2 (C_q), 129.8 (C_q), 129.7 (CH), 129.1 (CH), 128.5 (CH), 126.7 (CH), 75.5 (CH), 71.7 (CH), 69.6 (CH), 69.5 (CH), 67.2 (CH), 62.5 (CH_2), 21.1 (CH_3), 20.9 (CH_3), 20.9 (CH_3), 20.9 (CH_3).

IR (ATR): $\tilde{\nu}$ = 1744, 1536, 1416, 1221, 1048, 768, 733 cm^{-1}.

MS (ESI) *m/z* (relative intensity): 543 (100) $[M+Na]^+$.

HR-MS (ESI): m/z calcd for $C_{24}H_{26}ClN_2O_9^+$ $[M+Na]^+$ 543.1141, found 543.1154.

(2*R*,3*R*,4*R*,5*R*,6*R*)-2-(Acetoxymethyl)-6-(2-methyl-5-(pyrimidin-2-yl)phenyl)tetrahydro-2*H*-pyran-3,4,5-triyl-triacetate (11)

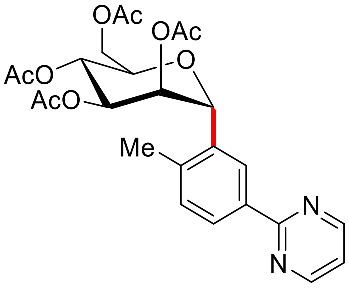

The general procedure was followed using 2-(*p*-tolyl)pyrimidine (**1g**) (17.0 mg, 0.10 mmol), (2*R*,3*R*,4*S*,5*S*,6*R*)-2-(acetoxymethyl)-6-bromotetrahydro-2*H*-pyran-3,4,5-triyl-triacetate (**2b**) (82.0 mg, 0.20 mmol), $[RuCl_2(p\text{-cymene})]_2$ (3.1 mg, 5.0 mol %), NaOAc (16.4 mg, 0.2 mmol) in 1,4-dioxane (1.0 mL) at ambient temperature. Purification by column chromatography on silica gel (*n*-hexane/EtOAc: 5/1 to 3/1) yielded **11** (32.0 mg, 64%) as a sticky solid.

^{1}H NMR (600 MHz, $CDCl_3$) δ 8.83 (d, *J* = 4.8 Hz, 2H), 8.62 (d, *J* = 1.8 Hz, 1H), 8.32 (dd, *J* = 7.9, 1.8 Hz, 1H), 7.33 (dd, *J* = 7.9, 0.9 Hz, 1H), 7.18 (t, *J* = 4.8 Hz, 1H), 5.92 (dd, *J* = 5.1, 3.3 Hz, 1H), 5.59 (dd, *J* = 7.3, 3.3 Hz, 1H), 5.33 – 5.17 (m, 2H), 4.51 (dd, *J* = 12.1, 7.1 Hz, 1H), 4.12 (dd, *J* = 12.1, 3.5 Hz, 1H), 3.84 (td, *J* = 6.7, 3.4 Hz, 1H), 2.50 (s, 3H), 2.14 (s, 3H), 2.08 (s, 3H), 2.07 (s, 3H), 2.05 (s, 3H).

^{13}C NMR (126 MHz, $CDCl_3$) δ 170.8 (C_q), 170.1 (C_q), 170.0 (C_q), 169.9 (C_q), 164.5 (C_q), 157.4 (CH), 141.1 (C_q), 135.9 (C_q), 134.4 (C_q), 131.8 (CH), 128.6 (CH), 127.2 (CH), 119.2 (CH), 72.7 (CH), 72.3 (CH), 69.7 (CH), 69.5 (CH), 67.9 (CH), 61.8 (CH_2), 21.0 (CH_3), 21.0 (CH_3), 20.9 (CH_3), 20.9 (CH_3), 20.1 (CH_3).

IR (ATR): $\tilde{\nu}$ = 1738, 1569, 1421, 1224, 1047, 798, 734 cm^{-1}.

MS (ESI) *m/z* (relative intensity): 523 (100) $[M+Na]^+$, 501 (22) $[M+H]^+$.

HR-MS (ESI): m/z calcd for $C_{25}H_{29}N_2O_8^+$ $[M+H]^+$ 501.1893, found 501.1868.

(2*R*,3*R*,4*R*,5*R*,6*R*)-2-(Acetoxymethyl)-6-(3-(5-fluoropyrimidin-2-yl)phenyl)tetrahydro-2*H*-pyran-3,4,5-triyl-triacetate (12)

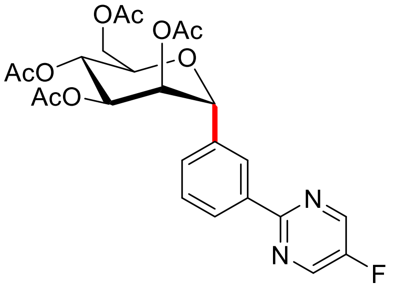

The general procedure was followed using 5-fluoro-2-phenylpyrimidine (**1h**) (17.4 mg, 0.10 mmol), (2*R*,3*R*,4*S*,5*S*,6*R*)-2-(acetoxymethyl)-6-bromotetrahydro-2*H*-pyran-3,4,5-triyl-triacetate (**2b**) (82.0 mg, 0.20 mmol), $[RuCl_2(p\text{-cymene})]_2$ (3.1 mg, 5.0 mol %), NaOAc (16.4 mg, 0.2 mmol) in 1,4-dioxane (1.0 mL) at ambient temperature. Purification by column chromatography on silica gel (*n*-hexane/EtOAc: 5/1 to 3/1) yielded **12** (30.2 mg, 60%) as a sticky solid.

^{1}H NMR (500 MHz, $CDCl_3$) δ 8.67 (d, J = 0.3 Hz, 2H), 8.56 (dq, J = 1.7, 1.1 Hz, 1H), 8.38 (dd, J = 7.7, 1.8 Hz, 1H), 7.62 (dd, J = 7.7, 2.1 Hz, 1H), 7.56 (t, J = 7.7 Hz, 1H), 6.03 (t, J = 3.4 Hz, 1H), 5.34 (t, J = 8.5 Hz, 1H), 5.21 (dd, J = 8.7, 3.2 Hz, 1H), 5.18 (d, J = 3.5 Hz, 1H), 4.41 (dd, J = 12.0, 6.7 Hz, 1H), 4.18 (dd, J = 12.1, 2.9 Hz, 1H), 3.89 – 3.83 (m, 1H), 2.16 (s, 3H), 2.15 (s, 3H), 2.08 (s, 3H), 2.02 (s, 3H).

^{19}F NMR (565 MHz, $CDCl_3$) δ -140.07.

^{13}C NMR (126 MHz, $CDCl_3$) δ 170.9 (C_q), 170.4 (C_q), 170.3 (C_q), 169.8 (C_q), 160.7 (d, $J_{C–F}$ = 6 Hz, C_q), 158.1 (C_q), 156.0 (C_q), 145.2 (d, $J_{C–F}$ = 21 Hz, CH), 137.7 (C_q), 136.1 (C_q), 129.6 (CH), 128.6 (CH), 128.6 (CH), 126.6 (CH), 75.5 (CH), 71.7 (CH), 69.7 (CH), 69.5 (CH), 67.2 (CH), 62.5 (CH_2), 21.1 (CH_3), 21.0 (CH_3), 20.9 (CH_3), 20.9 (CH_3).

IR (ATR): $\tilde{\nu}$ = 1745, 1424, 1225, 1051, 917, 456 cm^{-1}.

MS (ESI) *m/z* (relative intensity): 527 (100) $[M+Na]^+$, 505 (5) $[M+H]^+$.

HR-MS (ESI): m/z calcd for $C_{24}H_{26}N_2O_9F^+$ $[M+H]^+$ 505.1617, found 505.1612.

(2*R*,3*R*,4*R*,5*R*,6*R*)-2-(Acetoxymethyl)-6-(2-fluoro-5-(pyrimidin-2-yl)phenyl)tetrahydro-2*H*-pyran-3,4,5-triyl-triacetate (13)

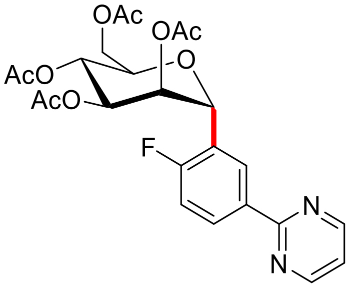

The general procedure was followed using 2-(4-fluorophenyl)pyrimidine (**1i**) (17.4 mg, 0.10 mmol), (2*R*,3*R*,4*S*,5*S*,6*R*)-2-(acetoxymethyl)-6-bromotetrahydro-2*H*-pyran-3,4,5-triyl-triacetate (**2b**) (82.0 mg, 0.20 mmol), $[RuCl_2(p\text{-cymene})]_2$ (3.1 mg, 5.0 mol %), NaOAc (16.4 mg, 0.2 mmol) in 1,4-dioxane (1.0 mL) at ambient temperature. Purification by column chromatography on silica gel (*n*-hexane/EtOAc: 5/1 to 3/1) yielded **13** (15.6 mg, 31%) as a sticky solid.

^{1}H NMR (600 MHz, $CDCl_3$) δ 8.81 (d, *J* = 4.8 Hz, 2H), 8.66 (dd, *J* = 7.2, 2.2 Hz, 1H), 8.45 (ddd, *J* = 8.7, 5.0, 2.2 Hz, 1H), 7.24 – 7.15 (m, 2H), 5.76 (dd, *J* = 6.6, 3.2 Hz, 1H), 5.42 (dd, *J* = 6.3, 3.2 Hz, 1H), 5.33 (d, *J* = 6.6 Hz, 1H), 5.19 (dd, *J* = 6.3, 5.0 Hz, 1H), 4.60 (dd, *J* = 12.0, 7.6 Hz, 1H), 4.26 (dd, *J* = 12.0, 4.1 Hz, 1H), 4.11 (dt, *J* = 7.8, 4.5 Hz, 1H), 2.15 (s, 3H), 2.13 (s, 3H), 2.10 (s, 3H), 2.00 (s, 3H).

^{19}F NMR (565 MHz, $CDCl_3$) δ -112.34.

^{13}C NMR (151 MHz, $CDCl_3$) δ 170.9 (C_q), 169.9 (C_q), 169.8 (C_q), 163.6 (C_q), 163.6 (C_q), 161.9 (C_q), 157.4 (CH), 134.4 (C_q), 134.4 (C_q), 130.7 (d, $J_{C\text{–}F}$ = 12 Hz, CH), 129.1 (d, $J_{C\text{–}F}$ = 5 Hz, CH), 123.9 (d, $J_{C\text{–}F}$ = 15 Hz, C_q), 119.3 (CH), 116.5 (d, $J_{C\text{–}F}$ = 23 Hz, CH), 73.3 (CH), 69.3 (d, $J_{C\text{–}F}$ = 3 Hz, CH), 68.8 (CH), 68.7 (CH), 68.1 (CH), 61.5 (CH_2), 21.1 (CH_3), 20.9 (CH_3), 20.9 (CH_3), 20.8 (CH_3).

IR (ATR): $\tilde{\nu}$ = 1737, 1557, 1421, 1208, 1046, 916, 802, 730 cm^{-1}. C24H25FN2O9, M+nH ,505.1

MS (ESI) *m/z* (relative intensity): 527 (100) $[M+Na]^+$, 505 (11) $[M+H]^+$.

HR-MS (ESI): m/z calcd for $C_{24}H_{26}N_2O_9F^+$ $[M+H]^+$ 505.1617, found 505.1636.

(2*R*,3*R*,4*R*,5*R*,6*R*)-2-(3-Acetylphenyl)-6-((benzoyloxy)methyl)tetrahydro-2*H*-pyran-3,4,5-triyl-tribenzoate (14)

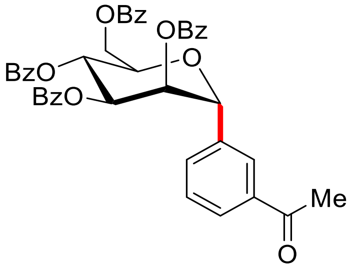

The general procedure was followed using (*E*/Z)-1-phenyl-*N*-(3,4,5-trimethoxyphenyl)ethan-1-imine (**1j**) (28.5 mg, 0.10 mmol), (2*R*,3*R*,4*S*,5*S*,6*R*)-2-[(benzoyloxy)methyl]-6-bromotetrahydro-2*H*-pyran-3,4,5-triyl-tribenzoate (**2a**) (131.6 mg, 0.20 mmol), [RuCl$_2$(*p*-cymene)]$_2$ (3.1 mg, 5.0 mol %), NaOAc (16.4 mg, 0.2 mmol) in 1,4-dioxane (1.0 mL) at ambient temperature. After 48 h, HCl aqueous solution (2M, 1.0 mL) was added at ambient temperature, and the resulting mixture was stirred for an additional 3 h, extracted with EtOAc (3 × 5 mL). The combined organic layers were dried over Na_2SO_4 and concentrated in *vacuo*. Purification by column chromatography on silica gel (*n*-hexane/EtOAc: 10/1 to 3/1) yielded **14** (10.4 mg, 20%) as a sticky solid.

^{1}H NMR (400 MHz, $CDCl_3$) δ 8.15 – 8.05 (m, 2H), 8.03 – 7.94 (m, 6H), 7.65 – 7.47 (m, 7H), 7.45 – 7.30 (m, 9H), 6.43 (dd, *J* = 3.9, 3.0 Hz, 1H), 6.14 (dd, *J* = 8.5, 7.6 Hz, 1H), 6.00 (d, *J* = 3.9 Hz, 1H), 5.72 (dd, *J* = 8.5, 3.0 Hz, 1H), 4.70 – 4.63 (m, 1H), 4.58 (dd, *J* = 12.3, 4.3 Hz, 1H), 4.06 (dt, *J* = 7.7, 3.8 Hz, 1H), 2.55 (s, 3H).

^{13}C NMR (101 MHz, $CDCl_3$) δ 203.9 (C_q), 166.2 (C_q), 166.2 (C_q), 165.6 (C_q), 165.4 (C_q), 141.1 (C_q), 133.8 (C_q), 133.6 (CH), 133.5 (CH), 133.1 (CH), 131.2 (CH), 130.1 (C_q), 130.0 (CH), 130.0 (CH), 130.0 (CH), 129.5 (C_q), 129.2 (C_q), 129.1 (CH), 129.1 (CH), 128.7 (CH), 128.6 (CH), 128.6 (CH), 128.5 (CH), 128.2 (CH), 127.5 (CH), 72.8 (CH), 72.5 (CH), 70.9 (CH), 70.1 (CH), 67.5 (CH), 62.2 (CH_2), 30.0 (CH_3).

IR (ATR): $\tilde{\nu}$ = 1720, 1601, 1263, 1092, 707, 686 cm^{-1}.

MS (ESI) *m/z* (relative intensity): 721 (100) [M+Na]$^+$, 699 (2) [M+H]$^+$.

HR-MS (ESI): m/z calcd for $C_{42}H_{35}O_{10}^+$ [M+H]$^+$ 699.2225, found 699.2226.

(2*S*,3*S*,4*R*,5*S*,6*S*)-2-Methyl-6-(3-(5-methylpyrimidin-2-yl)phenyl)tetrahydro-2*H*-pyran-3,4,5-triyl-tribenzoate (16)

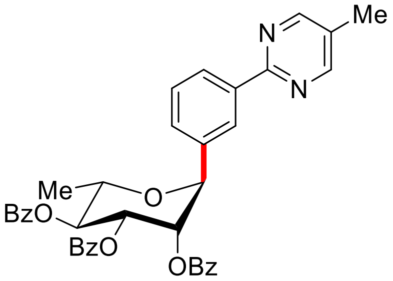

The general procedure was followed using 5-methyl-2-phenylpyrimidine (**1b**) (17.0 mg, 0.10 mmol), (3*R*,4*R*,5*S*,6*S*)-2-bromo-6-methyltetrahydro-2*H*-pyran-3,4,5-triyl-tribenzoate (**2i**) (107.6 mg, 0.20 mmol), [$RuCl_2$(*p*-cymene)$]_2$ (3.1 mg, 5.0 mol %), NaOAc (16.4 mg, 0.2 mmol) in 1,4-dioxane (1.0 mL) at ambient temperature. Purification by column chromatography on silica gel (*n*-hexane/EtOAc: 10/1 to 3/1) yielded **15** (29.5 mg, 47%) as a sticky solid.

^{1}H NMR (400 MHz, $CDCl_3$) δ 8.72 (d, *J* = 2.1, 1H), 8.68 (d, *J* = 0.8 Hz, 2H), 8.43 (dd, *J* = 7.8, 1.8 Hz, 1H), 8.16 – 8.07 (m, 2H), 7.99 – 7.85 (m, 4H), 7.79 (ddt, *J* = 7.6, 1.9, 1.0 Hz, 1H), 7.68 – 7.55 (m, 2H), 7.53 – 7.43 (m, 4H), 7.40 – 7.28 (m, 4H), 6.50 (t, *J* = 3.1 Hz, 1H), 5.78 (dd, *J* = 9.1, 8.3 Hz, 1H), 5.68 (dd, *J* = 9.1, 3.2 Hz, 1H), 5.43 (d, *J* = 3.0 Hz, 1H), 4.04 (dq, *J* = 8.4, 6.3 Hz, 1H), 2.36 (s, 3H), 1.48 (d, *J* = 6.3 Hz, 3H).

^{13}C NMR (101 MHz, $CDCl_3$) δ 166.0 (C_q), 165.9 (C_q), 165.8 (C_q), 162.3 (C_q), 157.6 (CH), 138.8 (C_q), 136.8 (C_q), 133.5 (CH), 133.4 (CH), 133.4 (CH), 130.1 (CH), 129.9 (CH), 129.8 (C_q), 129.6 (CH), 129.5 (C_q), 129.4 (C_q), 128.7 (C_q), 128.7 (CH), 128.5 (CH), 128.5 (CH), 128.4 (CH), 128.0 (CH), 126.5 (CH), 75.8 (CH), 72.5 (CH), 71.2 (CH), 70.9 (CH), 69.6 (CH), 17.8 (CH_3), 15.7 (CH_3).

IR (ATR): $\tilde{\nu}$ = 1737, 1373, 1233, 1043, 634, 607 cm^{-1}.

MS (ESI) *m/z* (relative intensity): 651 (100) $[M+Na]^+$, 629 (25) $[M+H]^+$.

HR-MS (ESI): m/z calcd for $C_{38}H_{33}N_2O_7^+$ $[M+H]^+$ 629.2282, found 629.2282.

(2*R*,3*S*,4*S*,5*S*)-2-(3-(5-Methylpyrimidin-2-yl)phenyl)tetrahydro-2*H*-pyran-3,4,5-triyl-tribenzoate (17)

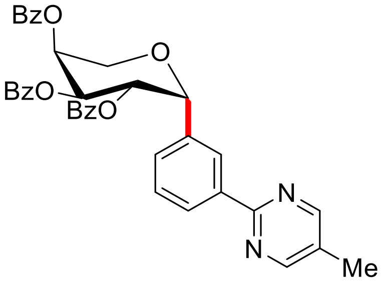

The general procedure was followed using 5-methyl-2-phenylpyrimidine (**1b**) (17.0 mg, 0.10 mmol), (2*R*,3*R*,4*S*,5*S*)-2-bromotetrahydro-2*H*-pyran-3,4,5-triyl-tribenzoate (**2j**) (104.8 mg, 0.20 mmol), $[RuCl_2(p\text{-cymene})]_2$ (3.1 mg, 5.0 mol %), NaOAc (16.4 mg, 0.2 mmol) in 1,4-dioxane (1.0 mL) at ambient temperature. Purification by column chromatography on silica gel (*n*-hexane/EtOAc: 10/1 to 3/1) yielded **16** (25.2 mg, 41%) as a sticky solid

^{1}H NMR (400 MHz, $CDCl_3$) δ 8.59 (d, J = 0.9 Hz, 2H), 8.49 (d, J = 1.8 Hz, 1H), 8.27 (d, J = 7.8 Hz, 1H), 8.24 – 8.17 (m, 2H), 8.06 – 7.95 (m, 2H), 7.92 – 7.84 (m, 2H), 7.76 – 7.64 (m, 1H), 7.62 – 7.52 (m, 3H), 7.55 – 7.46 (m, 2H), 7.42 – 7.30 (m, 5H), 6.05 – 5.94 (m, 1H), 5.75 (ddd, J = 10.8, 5.4, 3.1 Hz, 1H), 5.64 (dd, J = 3.9, 1.7 Hz, 1H), 5.34 – 5.29 (m, 1H), 4.44 (ddd, J = 10.8, 5.4, 1.2 Hz, 1H), 4.19 (t, J = 10.9 Hz, 1H), 2.33 (s, 3H).

^{13}C NMR (101 MHz, $CDCl_3$) δ 165.6 (C_q), 165.0 (C_q), 164.9 (C_q), 162.2 (C_q), 157.4 (CH), 137.7 (C_q), 137.2 (C_q), 133.8 (CH), 133.4 (CH), 133.4 (CH), 130.1 (CH), 130.1 (CH), 129.9 (CH), 129.7 (C_q), 129.5 (C_q), 129.2 (C_q), 128.9 (CH), 128.7 (CH), 128.6 (CH), 128.5 (CH), 128.5 (CH), 127.7 (CH), 126.2 (CH), 76.6 (CH), 71.7 (CH), 67.9 (CH), 66.5 (CH), 64.5 (CH_2), 15.7 (CH_3).

IR (ATR): $\tilde{\nu}$ = 1725, 1451, 1424, 1093, 1070, 1027, 784, 709 cm^{-1}.

MS (ESI) *m/z* (relative intensity): 637 (100) $[M+Na]^+$, 615 (46) $[M+H]^+$.

HR-MS (ESI): m/z calcd for $C_{37}H_{31}N_2O_7^+$ $[M+H]^+$ 615.2126, found 615.2138.

(2*R*,3*S*,4*R*,5*S*,6*R*)-2-(Acetoxymethyl)-6-(3-(5-methylpyrimidin-2-yl)phenyl)tetrahydro-2*H*-pyran-3,4,5-triyl-triacetate (19)

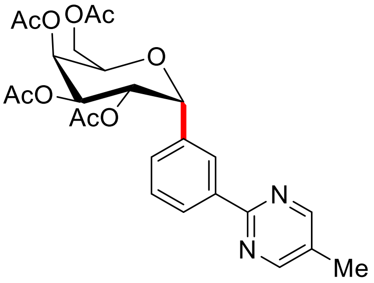

The general procedure was followed using 5-methyl-2-phenylpyrimidine (**1b**) (17.0 mg, 0.10 mmol), (2*R*,3*S*,4*S*,5*R*,6*R*)-2-(acetoxymethyl)-6-bromotetrahydro-2*H*-pyran-3,4,5-triyl-triacetate (**2k**) (82.0 mg, 0.20 mmol), $[RuCl_2(p\text{-cymene})]_2$ (3.1 mg, 5.0 mol %), NaOAc (16.4 mg, 0.2 mmol) in 1,4-dioxane (1.0 mL) at ambient temperature. Purification by column chromatography on silica gel (*n*-hexane/EtOAc: 5/1 to 2/1) yielded **17** (24.5 mg, 49%) as a sticky solid.

^{1}**H NMR** (400 MHz, $CDCl_3$) δ 8.63 (d, J = 0.8 Hz, 2H), 8.48 (d, J = 1.9 Hz, 1H), 8.35 (d, J = 7.8, 1H), 7.57 (d, J = 7.9 Hz, 1H), 7.46 (t, J = 7.8 Hz, 1H), 5.53 (dd, J = 5.0, 3.3 Hz, 1H), 5.44 (dd, J = 5.9, 3.2 Hz, 1H), 5.38 (d, J = 3.2 Hz, 1H), 5.36 (t, J = 3.1 Hz, 1H), 4.63 (dd, J = 12.1, 8.7 Hz, 1H), 4.32 (dt, J = 8.7, 4.4 Hz, 1H), 4.19 (dd, J = 12.2, 3.8 Hz, 1H), 2.35 (d, J = 0.8 Hz, 3H), 2.16 (s, 3H), 2.13 (s, 3H), 2.02 (s, 3H), 1.93 (s, 3H).

^{13}**C NMR** (101 MHz, $CDCl_3$) δ 171.0 (C_q), 170.0 (C_q), 169.6 (C_q), 169.5 (C_q), 162.2 (C_q), 157.5 (CH), 137.9 (C_q), 136.6 (C_q), 129.2 (CH), 128.7 (CH), 128.7 (C_q), 127.8 (CH), 127.1 (CH), 71.4 (CH), 71.1 (CH), 70.4 (CH), 68.0 (CH), 66.7 (CH), 60.3 (CH_2), 21.1 (CH_3), 21.0 (CH_3), 20.9 (CH_3), 20.8 (CH_3), 15.7 (CH_3).

IR (ATR): $\tilde{\nu}$ = 1737, 1372, 1233, 1043, 938, 847, 786, 634, 608 cm^{-1}.

MS (ESI) *m/z* (relative intensity): 523 (100) $[M+Na]^+$, 501 (21) $[M+H]^+$.

HR-MS (ESI): m/z calcd for $C_{25}H_{29}N_2O_9^+$ $[M+H]^+$ 501.1868, found 501.1873.

(2*S*,3*R*,4*R*,5*R*,6*S*)-2-Methyl-6-(3-(5-methylpyrimidin-2-yl)phenyl)tetrahydro-2*H*-pyran-3,4,5-triyl-tribenzoate (20)

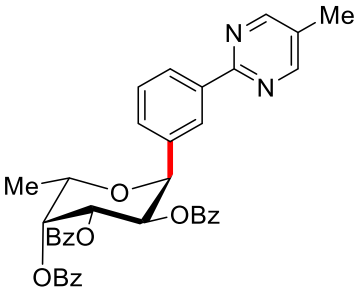

The general procedure was followed using 5-methyl-2-phenylpyrimidine (**1b**) (17.0 mg, 0.10 mmol), (3*S*,4*R*,5*R*,6*S*)-2-bromo-6-methyltetrahydro-2*H*-pyran-3,4,5-triyl-tribenzoate (**2l**) (107.6 mg, 0.20 mmol), $[RuCl_2(p\text{-cymene})]_2$ (3.1 mg, 5.0 mol %), NaOAc (16.4 mg, 0.2 mmol) in 1,4-dioxane (1.0 mL) at ambient temperature. Purification by column chromatography on silica gel (*n*-hexane/EtOAc: 10/1 to 4/1) yielded **18** (36.4 mg, 58%) as a sticky solid.

^{1}H NMR (400 MHz, $CDCl_3$) δ 8.80 (d, *J* = 1.8 Hz, 1H), 8.64 (s, 2H), 8.34 (d, *J* = 7.9 Hz, 1H), 8.10 – 8.06 (m, 2H), 8.05 – 8.01 (m, 2H), 7.99 – 7.94 (m, 2H), 7.82 – 7.78 (m, 1H), 7.63 – 7.56 (m, 1H), 7.55 – 7.51 (m, 1H), 7.51 – 7.43 (m, 4H), 7.41 – 7.32 (m, 4H), 6.05 (qd, *J* = 8.0, 3.6 Hz, 2H), 5.79 (t, *J* = 3.4 Hz, 1H), 5.67 (d, *J* = 3.9 Hz, 1H), 4.38 (qd, *J* = 6.7, 3.5 Hz, 1H), 2.35 (s, 3H), 1.45 (d, *J* = 6.7 Hz, 3H).

^{13}C NMR (101 MHz, $CDCl_3$) δ 165.9 (C_q), 165.6 (C_q), 165.5 (C_q), 162.3 (C_q), 157.5 (CH), 138.1 (C_q), 137.1 (C_q), 133.5 (CH), 133.4 (CH), 133.3 (CH), 130.1 (CH), 130.0 (CH), 129.9 (CH), 129.6 (CH), 129.5 (C_q), 129.4 (C_q), 128.9 (CH), 128.7 (CH), 128.6 (CH), 128.5 (CH), 127.7 (CH), 127.5 (CH), 72.2 (CH), 70.8 (CH), 70.5 (CH), 69.4 (CH), 68.5 (CH), 15.7 (CH_3), 15.5 (CH_3).

IR (ATR): $\tilde{\nu}$ = 1737, 1375, 1233, 1043, 938, 847, 634, 608 cm^{-1}.

MS (ESI) *m/z* (relative intensity): 651 (100) $[M+Na]^+$, 629 (15) $[M+H]^+$.

HR-MS (ESI): m/z calcd for $C_{38}H_{33}N_2O_7^+$ $[M+H]^+$ 629.2282, found 629.2278.

(2*R*,3*R*,4*R*,5*R*,6*R*)-2-(((4-Fluorobenzoyl)oxy)methyl)-6-(3-(pyrimidin-2-yl)phenyl)tetrahydro-2*H*-pyran-3,4,5-triyl-tris(4-fluorobenzoate) (21)

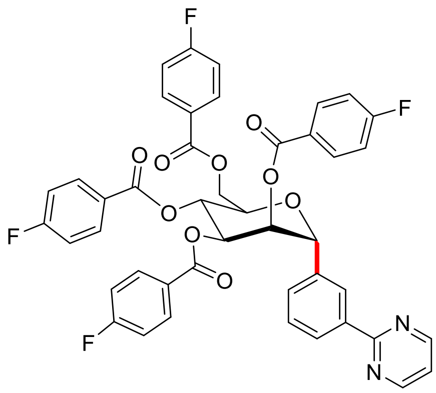

The general procedure was followed using 2-phenylpyrimidine (**1a**) (15.6 mg, 0.10 mmol), (2*R*,3*S*,4*S*,5*R*,6*R*)-2-bromo-6-(((4-fluorobenzoyl)oxy)methyl)tetrahydro-2*H*-pyran-3,4,5-triyl-tris(4-fluorobenzoate) (**2m**) (146.0 mg, 0.20 mmol), $[RuCl_2(p\text{-cymene})]_2$ (3.1 mg, 5.0 mol %), NaOAc (16.4 mg, 0.2 mmol) in 1,4-dioxane (1.0 mL) at ambient temperature. Purification by column chromatography on silica gel (*n*-hexane/EtOAc: 10/1 to 3/1) yielded **19** (27.4 mg, 34%) as a sticky solid.

^{1}H NMR (400 MHz, $CDCl_3$) δ 8.81 – 8.79 (m, 1H), 8.78 (d, J = 4.8 Hz, 2H), 8.50 (d, J = 7.8 Hz, 1H), 8.21 – 8.05 (m, 4H), 7.92 (ddd, J = 9.2, 8.1, 5.4 Hz, 4H), 7.82 (d, J = 7.7 Hz, 1H), 7.65 (t, J = 7.8 Hz, 1H), 7.22 (t, J = 4.8 Hz, 1H), 7.10 (td, J = 8.6, 6.0 Hz, 4H), 7.01 (td, J = 8.6, 1.8 Hz, 4H), 6.55 (t, J = 2.9 Hz, 1H), 6.06 (t, J = 9.4 Hz, 1H), 5.70 (dd, J = 9.4, 3.1 Hz, 1H), 5.51 (d, J = 2.6 Hz, 1H), 4.72 (dd, J = 12.0, 2.7 Hz, 1H), 4.59 (dd, J = 12.0, 5.9 Hz, 1H), 4.28 (ddd, J = 8.9, 5.9, 2.7 Hz, 1H).

^{19}F NMR (282 MHz, $CDCl_3$) δ -104.18, -104.21, -104.44, -105.13.

^{13}C NMR (101 MHz, $CDCl_3$) δ 167.5 (C_q), 167.4 (C_q), 167.3 (C_q), 165.2 (d, J =255 Hz, C_q), 164.9 (d, $J_{C\text{–}F}$ =255 Hz, C_q), 164.8 (d, $J_{C\text{–}F}$ =255 Hz, C_q), 164.5 (d, $J_{C\text{–}F}$ =255 Hz, C_q), 157.5 (CH), 139.0 (C_q), 135.7 (C_q), 132.7 (d, $J_{C\text{–}F}$ =9.4 Hz, CH), 132.6 (d, $J_{C\text{–}F}$ =9.4 Hz, CH), 132.6 (CH), 132.6 (d, $J_{C\text{–}F}$ =9.4 Hz, CH), 132.5 (d, $J_{C\text{–}F}$ =9.4 Hz, CH), 132.5 (CH), 132.4 (CH), 129.8 (CH), 128.7 (CH), 128.6 (CH), 126.6 (CH), 126.3 (d, $J_{C\text{–}F}$ = 3.0 Hz, C_q), 125.8 (d, $J_{C\text{–}F}$ = 2.9 Hz, C_q), 125.3 (d, J = 3.1 Hz, C_q), 125.1 (d, J = 3.0 Hz, C_q), 115.9 (d, J = 22.0 Hz, CH), 76.4 (CH), 71.3 (CH), 71.0 (CH), 70.8 (CH), 67.8 (CH), 63.5 (CH_2).

IR (ATR): $\tilde{\nu}$ = 1722, 1604, 1508, 1260, 1088, 906, 852, 731 cm^{-1}.

MS (ESI) *m/z* (relative intensity): 829 (100) $[M+Na]^+$, 807 (17) $[M+H]^+$.

HR-MS (ESI): m/z calcd for $C_{44}H_{31}F_4N_2O_9^+$ $[M+H]^+$ 807.1960, found 807.1957.

(2*R*,3*R*,4*R*,5*R*,6*R*)-2-(3-(Pyrimidin-2-yl)phenyl)-6-(((4-(trifluoromethyl)benzoyl)oxy)methyl)tetrahydro-2*H*-pyran-3,4,5-triyl-tris(4-(trifluoromethyl)benzoate) (22)

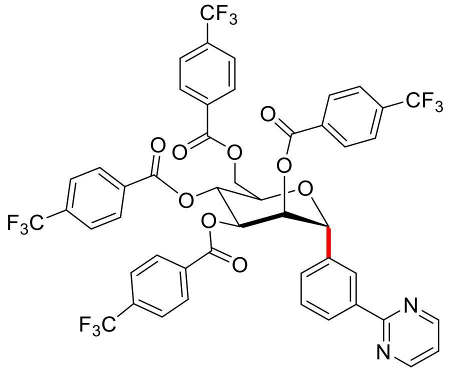

The general procedure was followed using 2-phenylpyrimidine (**1a**) (15.6 mg, 0.10 mmol), (2*R*,3*S*,4*S*,5*R*,6*R*)-2-bromo-6-(((4-(trifluoromethyl)benzoyl)oxy)methyl)tetrahydro-2*H*-pyran-3,4,5-triyl-tris(4-(trifluoromethyl)benzoate) (**2n**) (186.0 mg, 0.20 mmol), $[RuCl_2(p\text{-cymene})]_2$ (3.1 mg, 5.0 mol %), NaOAc (16.4 mg, 0.2 mmol) in 1,4-dioxane (1.0 mL) at ambient temperature. Purification by column chromatography on silica gel (*n*-hexane/EtOAc: 10/1 to 3/1) yielded **20** (37.2 mg, 37%) as a sticky solid.

^{1}H NMR (500 MHz, $CDCl_3$) δ 8.81 (s, 1H), 8.78 (d, *J* = 5.0 Hz, 2H), 8.54 (d, *J* = 7.7 Hz, 1H), 8.24 (d, *J* = 8.1 Hz, 2H), 8.17 (d, *J* = 8.0 Hz, 2H), 8.03 (d, *J* = 8.1 Hz, 2H), 7.99 (d, *J* = 8.1 Hz, 2H), 7.83 (d, *J* = 7.6 Hz, 1H), 7.73 – 7.65 (m, 5H), 7.64 – 7.58 (m, 4H), 7.24 (s, 1H), 6.64 (t, *J* = 2.6 Hz, 1H), 6.15 (t, *J* = 9.3 Hz, 1H), 5.76 (dd, *J* = 9.6, 2.9 Hz, 1H), 5.54 (d, *J* = 2.4 Hz, 1H), 4.81 (d, *J* = 11.8 Hz, 1H), 4.62 (dd, *J* = 12.1, 4.9 Hz, 1H), 4.31 (s, 1H).

^{19}F NMR (471 MHz, $CDCl_3$) δ -63.28, -63.30, -63.35, -63.36.

^{13}C NMR (126 MHz, $CDCl_3$) δ 165.0 (C_q), 164.8 (C_q), 164.6 (C_q), 164.4 (C_q), 164.2 (C_q), 157.5 (CH), 139.2 (C_q), 135.3 (C_q), 133.1 (C_q), 132.6 (C_q), 132.1 (C_q), 131.9 (C_q), 130.4 (CH), 130.0 (CH), 128.9 (CH), 128.6 (CH), 126.6 (CH), 125.9 (CH), 125.9 (CH), 125.8 (CH), 125.8 (CH), 125.7 (CH), 125.7 (CH), 125.7 (CH), 126.9 (q, J_{C-F} = 16 Hz, C_q), 124.6 (q, J_{C-F} = 14 Hz, C_q), 122.5 (q, J_{C-F} = 12 Hz, C_q), 120.2 (q, J_{C-F} = 13 Hz, C_q), 119.7 (CH), 76.5 (CH), 71.4 (CH), 71.1 (CH), 71.1 (CH), 68.0 (CH), 63.6 (CH_2).

IR (ATR): $\tilde{v}$ = 1720, 1568, 1411, 1325, 1278, 1097, 1067, 860, 772, 703 cm^{-1}.

MS (ESI) *m/z* (relative intensity): 1029 (100) $[M+Na]^+$, 1007 (8) $[M+H]^+$.

HR-MS (ESI): m/z calcd for $C_{48}H_{31}F_{12}N_2O_9^+$ $[M+H]^+$ 1007.1832, found 1007.1798.

(2*R*,3*R*,4*R*,5*R*,6*R*)-2-(((4-Methoxybenzoyl)oxy)methyl)-6-(3-(pyrimidin-2-yl)phenyl)tetrahydro-2*H*-pyran-3,4,5-triyl-tris(4-methoxybenzoate) (23)

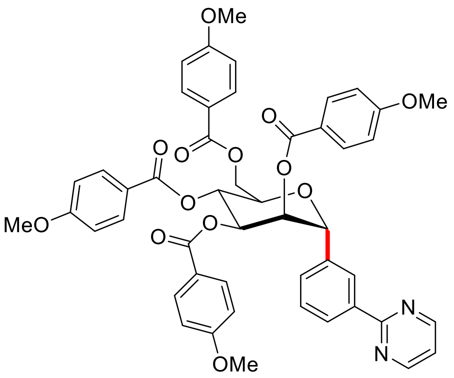

The general procedure was followed using 2-phenylpyrimidine (**1a**) (15.6 mg, 0.10 mmol), (2*R*,3*R*,4*R*,5*R*,6*R*)-2-(((4-methoxybenzoyl)oxy)methyl)-6-(3-(pyrimidin-2-yl)phenyl)tetrahydro-2*H*-pyran-3,4,5-triyl tris(4-methoxybenzoate) (**2o**) (155.6 mg, 0.20 mmol), $[RuCl_2(p\text{-cymene})]_2$ (3.1 mg, 5.0 mol %), NaOAc (16.4 mg, 0.2 mmol) in 1,4-dioxane (1.0 mL) at ambient temperature. Purification by column chromatography on silica gel (*n*-hexane/EtOAc: 10/1 to 3/1) yielded **21** (14.5 mg, 17%) as a sticky solid.

^{1}H NMR (600 MHz, $CDCl_3$) δ 8.82 – 8.81 (m, 1H), 8.78 (d, *J* = 4.8 Hz, 2H), 8.48 (dd, *J* = 7.8, 1.7 Hz, 1H), 8.14 – 8.09 (m, 2H), 8.07 – 8.03 (m, 2H), 77.88 – 7.83 (m, 5H), 7.63 (t, *J* = 7.8 Hz, 1H), 7.19 (t, *J* = 4.8 Hz, 1H), 6.92 – 6.87 (m, 4H), 6.82 – 6.77 (m, 4H), 6.51 (t, *J* = 2.9 Hz, 1H), 6.10 (t, *J* = 9.3 Hz, 1H), 5.67 (dd, *J* = 9.3, 3.1 Hz, 1H), 5.57 – 5.43 (m, 1H), 4.73 (dd, *J* = 11.9, 2.7 Hz, 1H), 4.56 (dd, *J* = 11.9, 5.9 Hz, 1H), 4.28 (ddd, *J* = 8.8, 5.8, 2.7 Hz, 1H), 3.87 (s, 3H), 3.87 (s, 3H), 3.79 (s, 3H), 3.78 (s, 3H).

^{13}C NMR (126 MHz, $CDCl_3$) δ 166.2 (C_q), 165.7 (C_q), 165.5, 165.3 (C_q), 164.5 (C_q), 163.8 (C_q), 163.8 (C_q), 163.7 (C_q), 163.5 (C_q), 157.5 (CH), 138.8 (C_q), 136.3 (C_q), 132.2 (CH), 132.1 (CH), 132.1 (CH), 132.1 (CH), 129.7 (CH), 128.8 (CH), 128.4 (CH), 126.8 (CH), 122.6 (C_q), 122.2 (C_q), 121.7 (C_q), 121.5 (C_q), 119.4 (CH), 113.9 (CH), 113.8 (CH), 113.8 (CH), 113.8 (CH), 76.4 (CH), 71.6 (CH), 70.7 (CH), 70.6 (CH), 67.5 (CH), 63.3 (CH_2), 55.6 (CH_3), 55.6 (CH_3), 55.6 (CH_3), 55.5 (CH_3).

IR (ATR): $\tilde{\nu}$ = 1714, 1604, 1511, 1410, 1251, 1167, 1094, 1029, 846, 767, 696 cm^{-1}.

MS (ESI) *m/z* (relative intensity): 877 (100) $[M+Na]^+$, 855 (8) $[M+H]^+$.

HR-MS (ESI): m/z calcd for $C_{48}H_{43}N_2O_{13}^+$ $[M+H]^+$ 855.2760, found 855.2774.

(2*R*,3*R*,4*R*,5*R*,6*R*)-2-(Acetoxymethyl)-5-(benzoyloxy)-6-(3-(pyrimidin-2-yl)phenyl)tetrahydro-2*H*-pyran-3,4-diyl-diacetate (24)

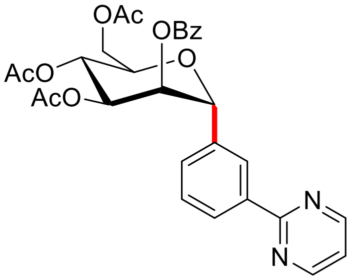

The general procedure was followed using 2-phenylpyrimidine (**1a**) (15.6 mg, 0.10 mmol), (2*R*,3*R*,4*S*,5*S*,6*R*)-2-(acetoxymethyl)-5-(benzoyloxy)-6-bromotetrahydro-2*H*-pyran-3,4-diyl-diacetate (**2p**) (94.4 mg, 0.20 mmol), $[RuCl_2(p\text{-cymene})]_2$ (3.1 mg, 5.0 mol %), NaOAc (16.4 mg, 0.2 mmol) in 1,4-dioxane (1.0 mL) at ambient temperature. Purification by column chromatography on silica gel (*n*-hexane/EtOAc: 5/1 to 2/1) yielded **22** (16.4 mg, 30%) as a sticky solid.

^{1}H NMR (400 MHz, $CDCl_3$) δ 8.82 (d, *J* = 4.8 Hz, 2H), 8.70 (q, *J* = 1.4 Hz, 1H), 8.50 – 8.38 (m, 1H), 8.18 – 8.07 (m, 2H), 7.72 – 7.66 (m, 1H), 7.64 – 7.55 (m, 2H), 7.53 – 7.43 (m, 2H), 7.22 (t, *J* = 4.8 Hz, 1H), 6.31 (t, *J* = 3.1 Hz, 1H), 5.49 (t, *J* = 8.8 Hz, 1H), 5.36 (d, *J* = 3.2 Hz, 1H), 5.32 (dd, *J* = 9.0, 3.0 Hz, 1H), 4.42 (dd, *J* = 12.0, 6.4 Hz, 1H), 4.25 (dd, *J* = 11.9, 2.8 Hz, 1H), 3.94 (ddd, *J* = 8.9, 6.3, 2.8 Hz, 1H), 2.18 (s, 3H), 2.04 (s, 3H), 2.02 (s, 3H).

^{13}C NMR (101 MHz, $CDCl_3$) δ 170.9 (C_q), 170.5 (C_q), 169.8 (C_q), 165.8 (C_q), 164.4 (C_q), 157.4 (CH), 138.7 (C_q), 136.0 (C_q), 133.6 (CH), 130.1 (CH), 129.7 (C_q), 129.6 (CH), 128.7 (CH), 128.7 (CH), 128.4 (CH), 126.7 (CH), 119.5 (CH), 75.9 (CH), 71.5 (CH), 70.3 (CH), 69.9 (CH), 67.2 (CH), 62.7 (CH_2), 20.9 (CH_3), 20.9 (CH_3).

IR (ATR): $\tilde{\nu}$ = 1747, 1556, 1411, 1222, 1096, 1047, 713 cm^{-1}.

MS (ESI) *m/z* (relative intensity): 571(100) $[M+Na]^+$, 549 (6) $[M+H]^+$.

HR-MS (ESI): m/z calcd for $C_{29}H_{29}N_2O_9^+$ $[M+H]^+$ 549.1868, found 549.1857.

(2*R*,3*R*,4*R*,5*R*,6R)-2-(Acetoxymethyl)-5-((4-fluorobenzoyl)oxy)-6-(3-(pyrimidin-2-yl)phenyl)tetrahydro-2*H*-pyran-3,4-diyl-diacetate (25)

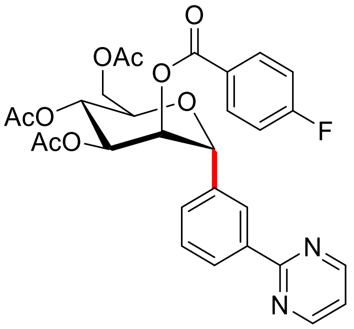

The general procedure was followed using 2-phenylpyrimidine (**1a**) (15.6 mg, 0.10 mmol), (2*R*,3*R*,4*S*,5*S*,6*R*)-2-(acetoxymethyl)-6-bromo-5-((4-fluorobenzoyl)oxy)tetrahydro-2*H*-pyran-3,4-diyl-diacetate (**2q**) (98.0 mg, 0.20 mmol), [RuCl$_2$(*p*-cymene)]$_2$ (3.1 mg, 5.0 mol %), NaOAc (16.4 mg, 0.2 mmol) in 1,4-dioxane (1.0 mL) at ambient temperature. Purification by column chromatography on silica gel (*n*-hexane/EtOAc: 5/1 to 2/1) yielded **23** (18.11 mg, 32%) as a sticky solid.

^{1}H NMR (400 MHz, CDCl$_3$) δ 8.82 (d, *J* = 4.8 Hz, 2H), 8.68 (t, *J* = 1.7 Hz, 1H), 8.53 – 8.35 (m, 1H), 8.24 – 8.04 (m, 2H), 7.80 – 7.65 (m, 1H), 7.58 (t, *J* = 7.8 Hz, 1H), 7.22 (t, *J* = 4.8 Hz, 1H), 7.15 (dd, *J* = 8.7, 6.7 Hz, 2H), 6.27 (t, *J* = 3.2 Hz, 1H), 5.46 (t, *J* = 8.7 Hz, 1H), 5.38 – 5.31 (m, 1H), 5.35 – 5.28 (m, 1H), 4.42 (dd, *J* = 12.0, 6.5 Hz, 1H), 4.24 (dd, *J* = 12.0, 2.8 Hz, 1H), 3.94 (ddd, *J* = 8.9, 6.4, 2.8 Hz, 1H), 2.17 (s, 3H), 2.05 (s, 3H), 2.02 (s, 3H).

^{19}F NMR (377 MHz, CDCl$_3$) δ -104.58.

^{13}C NMR (101 MHz, CDCl$_3$) δ 170.9 (C$_q$), 170.4 (C$_q$), 169.8 (C$_q$), 165.0 (C$_q$), 164.9 (C$_q$), 164.4 (C$_q$), 157.4 (CH), 138.7 (C$_q$), 135.9 (C$_q$), 132.8 (d, J_{C-F} = 7 Hz, CH), 129.6 (CH), 128.6 (CH), 128.4 (CH), 126.7 (CH), 125.9 (d, J_{C-F} = 3 Hz, C$_q$), 119.6 (CH), 115.9 (d, J_{C-F} = 10 Hz, CH), 75.7 (CH), 71.5 (CH), 70.5 (CH), 69.9 (CH), 67.2 (CH), 62.7 (CH$_2$), 20.9 (CH$_3$), 20.9 (CH$_3$).

IR (ATR): $\tilde{v}$ = 1749, 1569, 1556, 1274, 1223, 1110, 1048, 917, 765 cm^{-1}.

MS (ESI) *m/z* (relative intensity): 589 (100) [M+Na]$^+$, 567 (4) [M+H]$^+$, 605 (8) [M+K]$^+$.

HR-MS (ESI): m/z calcd for $C_{29}H_{28}FN_2O_9^+$ [M+H]$^+$ 567.1773, found 567.1792.

(2*R*,3*R*,4*R*,5*R*,6*R*)-2-((((*S*)-2-(7-Methoxynaphthalen-2-yl)propanoyl)oxy)methyl)-6-(3-(pyrimidin-2-yl)phenyl)tetrahydro-2*H*-pyran-3,4,5-triyl-triacetate (28)

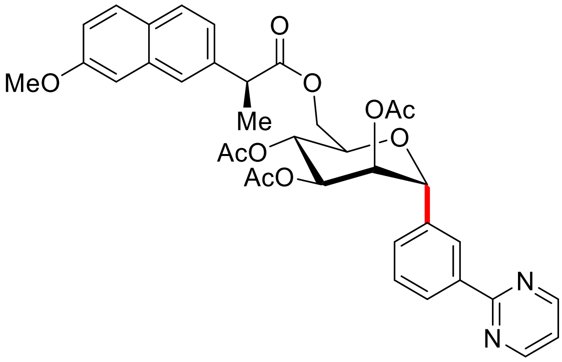

The general procedure was followed using 2-phenylpyrimidine (**1a**) (15.6 mg, 0.10 mmol), (2*R*,3*S*,4*S*,5*R*,6*R*)-2-bromo-6-((((*S*)-2-(7-methoxynaphthalen-2-yl)propanoyl)oxy)methyl)tetrahydro-2*H*-pyran-3,4,5-triyl-triacetate (**2r**) (116.0 mg, 0.20 mmol), $[RuCl_2(p\text{-cymene})]_2$ (3.1 mg, 5.0 mol %), NaOAc (16.4 mg, 0.2 mmol) in 1,4-dioxane (1.0 mL) at ambient temperature. Purification by column chromatography on silica gel (*n*-hexane/EtOAc: 5/1 to 2/1) yielded **25** (57.1 mg, 87%) as a sticky solid.

^{1}H NMR (400 MHz, $CDCl_3$) δ 8.81 (d, *J* = 4.8 Hz, 2H), 8.62 (d, *J* = 2.0 Hz, 1H), 8.42 (dt, *J* = 7.7, 1.5 Hz, 1H), 7.70 (d, *J* = 1.9 Hz, 1H), 7.64 (t, *J* = 8.7 Hz, 1H), 7.51 (d, *J* = 7.6 Hz, 2H), 7.47 – 7.39 (m, 1H), 7.19 (t, *J* = 4.8 Hz, 2H), 7.13 – 7.03 (m, 1H), 5.96 (dd, *J* = 4.1, 2.9 Hz, 2H), 5.36 – 5.17 (m, 1H), 5.13 (d, *J* = 4.0 Hz, 2H), 4.39 (dd, *J* = 12.0, 7.6 Hz, 1H), 4.24 (dd, *J* = 12.0, 2.9 Hz, 1H), 3.98 (q, *J* = 5.7 Hz, 1H), 3.93 – 3.85 (m, 4H), 2.06 (s, 6H), 1.99 (s, 3H), 1.57 (d, *J* = 7.2 Hz, 3H).

^{13}C NMR (101 MHz, $CDCl_3$) δ 174.4 (C_q), 170.3 (C_q), 170.2 (C_q), 169.8 (C_q), 164.5 (C_q), 157.8 (C_q), 157.4 (CH), 138.5 (C_q), 136.3 (C_q), 135.5 (C_q), 133.9 (C_q), 129.4 (CH), 129.4 (CH), 129.1 (C_q), 128.9 (CH), 128.4 (CH), 127.3 (CH), 126.7 (CH), 126.5 (CH), 126.2 (CH), 119.5 (CH), 119.0 (CH), 105.7 (CH), 74.9 (CH), 71.9 (CH), 69.5 (CH), 69.5 (CH), 67.5 (CH), 62.9 (CH_2), 55.4 (CH_3), 45.5 (CH), 21.0 (CH_3), 20.9 (CH_3), 18.6 (CH_3).

IR (ATR): $\tilde{\nu}$ = 1745, 1251, 1217, 901, 724, 649 cm^{-1}.

MS (ESI) *m/z* (relative intensity): 679 (100) $[M+Na]^+$, 657 (60) $[M+H]^+$.

HR-MS (ESI): m/z calcd for $C_{36}H_{37}N_2O_{10}^+$ $[M+H]^+$ 657.2443, found 657.2441.

(2*R*,3*R*,4*R*,5*R*,6*R*)-2-(((2-(4-(2,2-Dichlorocyclopropyl)phenoxy)-2-methylpropanoyl)oxy)methyl)-6-(3-(pyrimidin-2-yl)phenyl)tetrahydro-2*H*-pyran-3,4,5-triyl-triacetate (29)

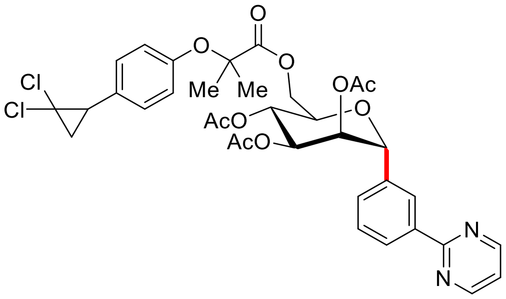

The general procedure was followed using 2-phenylpyrimidine (**1a**) (15.6 mg, 0.10 mmol), (2*R*,3*S*,4*S*,5*R*,6*R*)-2-bromo-6-(((2-(4-(2,2-dichlorocyclopropyl)phenoxy)-2-methylpropanoyl)oxy)methyl)tetrahydro-2*H*-pyran-3,4,5-triyl-triacetate (**2s**) (127.6 mg, 0.20 mmol), $[RuCl_2(p\text{-cymene})]_2$ (3.1 mg, 5.0 mol %), NaOAc (16.4 mg, 0.2 mmol) in 1,4-dioxane (1.0 mL) at ambient temperature. Purification by column chromatography on silica gel (*n*-hexane/EtOAc: 5/1 to 2/1) yielded **26** (24.28 mg, 34%) as a sticky solid.

^{1}H NMR (400 MHz, $CDCl_3$) δ 8.81 (d, *J* = 4.8 Hz, 2H), 8.59 (d, *J* = 1.9 Hz, 1H), 8.51 – 8.38 (m, 1H), 7.61 (d, *J* = 7.7 Hz, 1H), 7.52 (t, *J* = 7.7 Hz, 1H), 7.20 (td, *J* = 4.8, 0.8 Hz, 1H), 7.06 – 6.95 (m, 2H), 6.82 (dd, *J* = 8.7, 1.1 Hz, 2H), 5.95 (dd, *J* = 4.3, 3.0 Hz, 1H), 5.38 – 5.22 (m, 2H), 5.16 (d, *J* = 4.3 Hz, 1H), 4.58 (dd, *J* = 12.0, 7.0 Hz, 1H), 4.29 (ddd, *J* = 12.0, 2.9, 1.5 Hz, 1H), 3.93 (dt, *J* = 7.1, 3.8 Hz, 1H), 2.82 – 2.69 (m, 1H), 2.09 (d, *J* = 1.2 Hz, 3H), 2.07 (d, *J* = 1.0 Hz, 3H), 2.04 (s, 3H), 1.89 (dd, *J* = 10.7, 7.4 Hz, 1H), 1.72 (ddd, *J* = 8.4, 7.3, 1.1 Hz, 1H), 1.61 (s, 6H).

^{13}C NMR (101 MHz, $CDCl_3$) δ 173.9 (C_q), 170.2 (C_q), 170.2 (C_q), 169.8 (C_q), 164.4 (C_q), 157.5 (CH), 154.9 (C_q), 138.6 (C_q), 136.3 (C_q), 129.7 (CH), 129.4 (CH), 129.1 (CH), 128.5 (C_q), 128.5 (CH), 128.5 (CH), 126.8 (CH), 119.6 (CH), 119.5 (CH), 119.5 (CH), 79.4 (C_q), 77.2 (CH), 74.8 (CH), 72.0 (CH), 69.5 (CH), 69.4 (CH), 67.4 (CH), 63.0 (CH_2), 61.0 (C_q), 35.0 (CH), 26.0 (CH_2), 25.6 (CH_3), 25.5 (CH_3), 21.0 (CH_3), 20.9 (CH_3).

IR (ATR): $\tilde{\nu}$ = 1737, 1511, 1411, 1245, 1212, 1116, 1046, 911, 730 cm^{-1}.

MS (ESI) *m/z* (relative intensity): 737 (100) $[M+Na]^+$, 715 (34) $[M+H]^+$.

HR-MS (ESI): m/z calcd for $C_{35}H_{37}Cl_2N_2O_{10}^+$ $[M+H]^+$ 715.1820, found 715.1825.

2*R*,3*R*,4*R*,5*R*,6*R*)-2-((((*S*)-2-(4-Isobutylphenyl)propanoyl)oxy)methyl)-6-(3-(pyrimidin-2-yl)phenyl)tetrahydro-2*H*-pyran-3,4,5-triyl-triacetate (30)

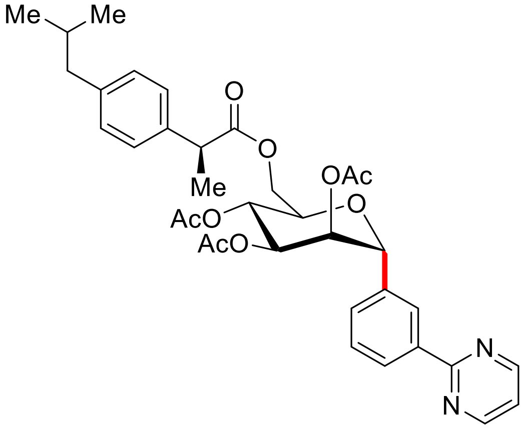

The general procedure was followed using 2-phenylpyrimidine (**1a**) (15.6 mg, 0.10 mmol), (2*R*,3*S*,4*S*,5*R*,6*R*)-2-bromo-6-((((*S*)-2-(4-isobutylphenyl)propanoyl)oxy)methyl)tetrahydro-2*H*-pyran-3,4,5-triyl-triacetate (**2t**) (111.2 mg, 0.20 mmol), [RuCl$_2$(*p*-cymene)]$_2$ (3.1 mg, 5.0 mol %), NaOAc (16.4 mg, 0.2 mmol) in 1,4-dioxane (1.0 mL) at ambient temperature. Purification by column chromatography on silica gel (*n*-hexane/EtOAc: 5/1 to 2/1) yielded **27** (55.00 mg, 87%) as a sticky solid.

^{1}H NMR (400 MHz, CDCl$_3$) δ 8.82 (d, *J* = 4.7 Hz, 2H), 8.64 (s, 1H), 8.44 (d, *J* = 6.7 Hz, 1H), 7.61 – 7.48 (m, 2H), 7.25 – 7.17 (m, 3H), 7.08 – 6.97 (m, 2H), 5.99 (dd, *J* = 3.8, 2.9 Hz, 1H), 5.32 – 5.17 (m, 2H), 5.14 (d, *J* = 3.8 Hz, 1H), 4.33 (dd, *J* = 12.0, 7.5 Hz, 1H), 4.25 (dd, *J* = 11.9, 3.0 Hz, 1H), 3.88 (td, *J* = 7.5, 3.0 Hz, 1H), 3.81 (q, *J* = 7.2 Hz, 1H), 2.40 (d, *J* = 7.1 Hz, 2H), 2.12 (s, 3H), 2.07 (s, 3H), 2.00 (s, 3H), 1.80 (dp, *J* = 13.6, 6.8 Hz, 1H), 1.48 (d, *J* = 7.2 Hz, 3H), 0.87 (d, *J* = 6.6 Hz, 6H).

^{13}C NMR (101 MHz, CDCl$_3$) δ 174.5 (C$_q$), 170.3 (C$_q$), 170.3 (C$_q$), 169.8 (C$_q$), 164.5 (C$_q$), 157.5 (CH), 140.7 (C$_q$), 138.6 (C$_q$), 137.5 (C$_q$), 136.2 (C$_q$), 129.5 (CH), 129.5 (CH), 128.9 (CH), 128.4 (CH), 127.4 (CH), 126.7 (CH), 119.5 (CH), 75.1 (CH), 71.9 (CH), 69.6 (CH), 69.5 (CH), 67.4 (CH), 62.9 (CH$_2$), 45.2 (CH), 45.2 (CH$_2$), 30.3 (CH), 22.5 (CH$_3$), 21.1 (CH$_3$), 20.9 (CH$_3$), 20.9 (CH$_3$), 18.5 (CH$_3$).

IR (ATR): $\tilde{\nu}$ = 1736, 1568, 1555, 1367, 1411, 1212, 1046, 911, 731 cm^{-1}.

MS (ESI) *m/z* (relative intensity): 655 (100) [M+Na]$^+$, 633 (40) [M+H]$^+$.

HR-MS (ESI): m/z calcd for $C_{35}H_{41}N_2O_9^+$ [M+H]$^+$ 633.2807, found 633.2815.

(2*R*,3*R*,4*R*,5*R*,6R)-2-(((Cyclopropanecarbonyl)oxy)methyl)-6-(3-(pyrimidin-2-yl)phenyl)tetrahydro-2*H*-pyran-3,4,5-triyl-triacetate (31)

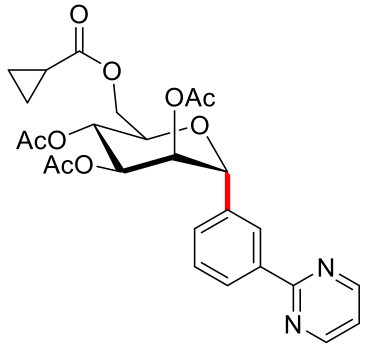

The general procedure was followed using 2-phenylpyrimidine (**1a**) (15.6 mg, 0.10 mmol), (2R,3*S*,4*S*,5*R*,6*R*)-2-bromo-6-(((cyclopropanecarbonyl)oxy)methyl)tetrahydro-2*H*-pyran-3,4,5-triyl-triacetate (**2u**) (87.2 mg, 0.20 mmol), [$RuCl_2$(*p*-cymene)$]_2$ (3.1 mg, 5.0 mol %), NaOAc (16.4 mg, 0.2 mmol) in 1,4-dioxane (1.0 mL) at ambient temperature. Purification by column chromatography on silica gel (*n*-hexane/EtOAc: 5/1 to 2/1) yielded **28** (26.12 mg, 51%) as a sticky solid.

^{1}H NMR (400 MHz, $CDCl_3$) δ 8.81 (d, *J* = 4.9 Hz, 2H), 8.63 (d, *J* = 2.1 Hz, 1H), 8.43 (d, *J* = 7.7 Hz, 1H), 7.64 (dd, *J* = 7.9, 2.2 Hz, 1H), 7.56 (t, *J* = 7.7 Hz, 1H), 7.21 (t, *J* = 4.8 Hz, 1H), 6.02 (t, *J* = 3.4 Hz, 1H), 5.33 (t, *J* = 8.3 Hz, 1H), 5.23 (dd, *J* = 8.6, 3.2 Hz, 1H), 5.19 (d, *J* = 3.7 Hz, 1H), 4.37 (dd, *J* = 12.0, 6.7 Hz, 1H), 4.23 (dd, *J* = 12.0, 3.0 Hz, 1H), 3.89 (ddd, *J* = 8.0, 6.7, 3.0 Hz, 1H), 2.15 (s, 3H), 2.08 (s, 3H), 2.02 (s, 3H), 1.72 (tt, *J* = 8.1, 4.8 Hz, 1H), 1.05 (dd, *J* = 4.6, 3.1 Hz, 2H), 0.93 – 0.81 (m, 2H).

^{13}C NMR (101 MHz, $CDCl_3$) δ 174.7 (C_q), 170.4 (C_q), 170.3 (C_q), 169.8 (C_q), 164.4 (C_q), 157.4 (CH), 138.6 (C_q), 136.1 (C_q), 129.5 (CH), 128.8 (CH), 128.4 (CH), 126.7 (CH), 119.5 (CH), 75.3 (CH), 71.7 (CH), 69.6 (CH), 69.5 (CH), 67.3 (CH), 62.5 (CH_2), 21.1 (CH_3), 20.9 (CH_3), 13.0 (CH), 8.8 (CH_2), 8.8 (CH_2).

IR (ATR): $\tilde{\nu}$ = 1746, 1569, 1555, 1410, 1367, 1211, 1170, 1045, 911, 729 cm^{-1}.

MS (ESI) *m/z* (relative intensity): 535 (100) $[M+Na]^+$, 513 (17) $[M+H]^+$.

HR-MS (ESI): m/z calcd for $C_{26}H_{29}N_2O_9^+$ $[M+H]^+$ 513.1868, found 513.1890.

(2*R*,3*R*,4*R*,5*R*,6*R*)-2-(((Cyclopentanecarbonyl)oxy)methyl)-6-(3-(pyrimidin-2-yl)phenyl)tetrahydro-2*H*-pyran-3,4,5-triyl-triacetate (32)

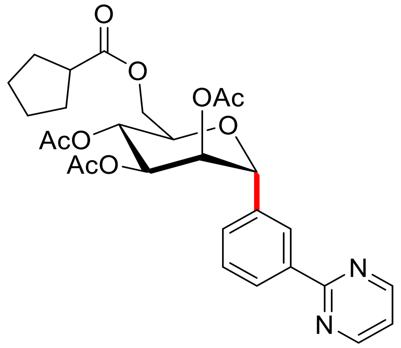

The general procedure was followed using 2-phenylpyrimidine (**1a**) (15.6 mg, 0.10 mmol), (2*R*,3*S*,4*S*,5*R*,6*R*)-2-bromo-6-(((cyclopentanecarbonyl)oxy)methyl)tetrahydro-2*H*-pyran-3,4,5-triyl-triacetate (**2v**) (92.81 mg, 0.20 mmol), $[RuCl_2(p\text{-cymene})]_2$ (3.1 mg, 5.0 mol %), NaOAc (16.4 mg, 0.2 mmol) in 1,4-dioxane (1.0 mL) at ambient temperature. Purification by column chromatography on silica gel (*n*-hexane/EtOAc: 5/1 to 2/1) yielded **29** (29.17 mg, 54%) as a sticky solid.

^{1}H NMR (400 MHz, $CDCl_3$) δ 8.82 (d, *J* = 4.9 Hz, 2H), 8.61 (d, *J* = 2.0 Hz, 1H), 8.47 – 8.38 (m, 1H), 7.64 (dq, *J* = 7.7, 1.1 Hz, 1H), 7.55 (t, *J* = 7.7 Hz, 1H), 7.22 (t, *J* = 4.8 Hz, 1H), 6.03 (t, *J* = 3.4 Hz, 1H), 5.34 (t, *J* = 8.4 Hz, 1H), 5.23 (dd, *J* = 8.6, 3.1 Hz, 1H), 5.19 (d, *J* = 3.8 Hz, 1H), 4.39 (dd, *J* = 12.0, 6.6 Hz, 1H), 4.22 (dd, *J* = 12.1, 2.9 Hz, 1H), 3.87 (ddd, *J* = 8.1, 6.6, 3.0 Hz, 1H), 2.83 (p, *J* = 8.0 Hz, 1H), 2.14 (s, 3H), 2.08 (s, 3H), 2.02 (s, 3H), 1.98 – 1.89 (m, 1H), 1.88 – 1.74 (m, 3H), 1.73 – 1.60 (m, 2H), 1.56 – 1.50 (m, 2H).

^{13}C NMR (101 MHz, $CDCl_3$) δ 176.6 (C_q), 170.4 (C_q), 170.3 (C_q), 169.8 (C_q), 164.5 (C_q), 157.4 (CH), 138.6 (C_q), 136.2 (C_q), 129.5 (CH), 128.9 (CH), 128.4 (CH), 126.8 (CH), 119.5 (CH), 75.3 (CH), 71.8 (CH), 69.7 (CH), 69.5 (CH), 67.2 (CH), 62.2 (CH_2), 43.9 (CH), 30.1 (CH_2), 30.0 (CH_2), 26.0 (CH_2), 26.0 (CH_2), 21.1 (CH_3), 21.0 (CH_3), 20.9 (CH_3).

IR (ATR): $\tilde{\nu}$ = 1728, 1555, 1411, 1367, 1210, 1151, 1038, 912, 734 cm^{-1}.

MS (ESI) *m/z* (relative intensity): 541 (100) $[M+H]^+$, 563 (42) $[M+Na]^+$.

HR-MS (ESI): m/z calcd for $C_{28}H_{33}N_2O_9^+$ $[M+H]^+$ 541.2181, found 541.2202.

(2*R*,3*R*,4*R*,5*R*,6*R*)-2-(((Cyclohexanecarbonyl)oxy)methyl)-6-(3-(pyrimidin-2-yl)phenyl)tetrahydro-2*H*-pyran-3,4,5-triyl-triacetate (33)

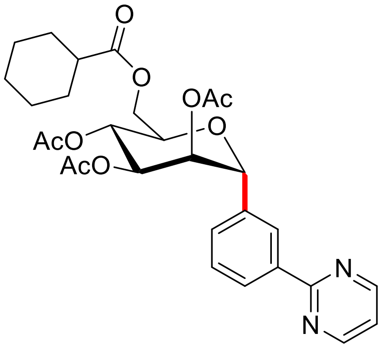

The general procedure was followed using 2-phenylpyrimidine (**1a**) (15.6 mg, 0.10 mmol), (2*R*,3*S*,4*S*,5*R*,6*R*)-2-bromo-6-(((cyclohexanecarbonyl)oxy)methyl)tetrahydro-2*H*-pyran-3,4,5-triyl-triacetate (**2w**) (95.62 mg, 0.20 mmol), $[RuCl_2(p\text{-cymene})]_2$ (3.1 mg, 5.0 mol %), NaOAc (16.4 mg, 0.2 mmol) in 1,4-dioxane (1.0 mL) at ambient temperature. Purification by column chromatography on silica gel (*n*-hexane/EtOAc: 5/1 to 2/1) yielded **30** (21.61 mg, 39%) as a sticky solid.

^{1}H NMR (400 MHz, $CDCl_3$) δ 8.83 (d, *J* = 4.9 Hz, 2H), 8.60 (d, *J* = 2.0 Hz, 1H), 8.43 (dq, *J* = 7.7, 0.9 Hz, 1H), 7.64 (dd, *J* = 7.7, 2.0 Hz, 1H), 7.55 (t, *J* = 7.8 Hz, 1H), 7.22 (t, *J* = 4.8 Hz, 1H), 6.02 (t, *J* = 3.4 Hz, 1H), 5.34 (t, *J* = 8.3 Hz, 1H), 5.23 (dd, *J* = 8.6, 3.1 Hz, 1H), 5.19 (d, *J* = 3.7 Hz, 1H), 4.38 (dd, *J* = 12.1, 6.4 Hz, 1H), 4.22 (dd, *J* = 12.1, 3.0 Hz, 1H), 3.87 (ddd, *J* = 8.0, 6.3, 3.0 Hz, 1H), 2.39 (tt, *J* = 11.3, 3.7 Hz, 1H), 2.14 (s, 3H), 2.08 (s, 3H), 2.02 (s, 3H), 1.99 – 1.87 (m, 2H), 1.78 – 1.65 (m, 2H), 1.67 – 1.62 (m, 1H), 1.53 – 1.40 (m, 2H), 1.34 – 1.16 (m, 3H).

^{13}C NMR (101 MHz, $CDCl_3$) δ 175.8 (C_q), 170.3 (C_q), 170.3 (C_q), 169.8 (C_q), 164.4 (C_q), 157.5 (CH), 138.6 (C_q), 136.2 (C_q), 129.5 (CH), 128.9 (CH), 128.4 (CH), 126.8 (CH), 119.5 (CH), 75.3 (CH), 71.8 (CH), 69.7 (CH), 69.5 (CH), 67.1 (CH), 62.0 (CH_2), 43.2 (CH), 29.1 (CH_2), 29.0 (CH_2), 25.9 (CH_2), 25.6 (CH_2), 25.6 (CH_2), 21.1 (CH_3), 20.9 (CH_3), 20.9 (CH_3).

IR (ATR): $\tilde{\nu}$ = 1746, 1555, 1410, 1245, 1213, 1047, 911, 731 cm^{-1}.

MS (ESI) *m/z* (relative intensity): 677 (100) $[M+Na]^+$, 555 (9) $[M+H]^+$.

HR-MS (ESI): m/z calcd for $C_{29}H_{35}N_2O_9^+$ $[M+H]^+$ 555.2337, found 555.2332.

(2*R*,3*R*,4*R*,5*R*,6*R*)-2-((Butyryloxy)methyl)-6-(3-(pyrimidin-2-yl)phenyl)tetrahydro-2*H*-pyran-3,4,5-triyl-triacetate (34)

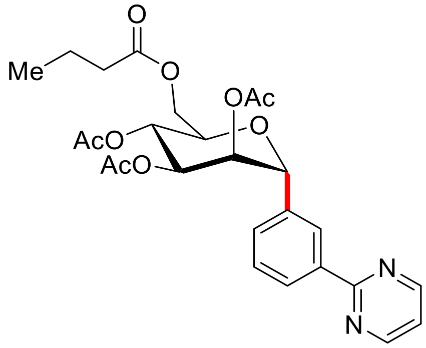

The general procedure was followed using 2-phenylpyrimidine (**1a**) (15.6 mg, 0.10 mmol), (2*R*,3*S*,4*S*,5*R*,6R)-2-bromo-6-((butyryloxy)methyl)tetrahydro-2*H*-pyran-3,4,5-triyl-triacetate (**2x**) (87.6 mg, 0.20 mmol), $[RuCl_2(p\text{-cymene})]_2$ (3.1 mg, 5.0 mol %), NaOAc (16.4 mg, 0.2 mmol) in 1,4-dioxane (1.0 mL) at ambient temperature. Purification by column chromatography on silica gel (*n*-hexane/EtOAc: 5/1 to 2/1) yielded **31** (21.08 mg, 41%) as a sticky solid.

^{1}H NMR (400 MHz, $CDCl_3$) δ 8.82 (d, *J* = 4.9 Hz, 2H), 8.62 (tt, *J* = 1.6, 0.7 Hz, 1H), 8.52 – 8.36 (m, 1H), 7.63 (dd, *J* = 7.7, 2.0 Hz, 1H), 7.56 (t, *J* = 7.7 Hz, 1H), 7.22 (t, *J* = 4.8 Hz, 1H), 6.03 (t, *J* = 3.4 Hz, 1H), 5.33 (t, *J* = 8.4 Hz, 1H), 5.22 (dd, *J* = 8.7, 3.1 Hz, 1H), 5.19 (d, *J* = 3.7 Hz, 1H), 4.48 – 4.35 (m, 1H), 4.21 (dd, *J* = 12.1, 2.9 Hz, 1H), 3.87 (ddd, *J* = 8.1, 6.7, 2.9 Hz, 1H), 2.39 (td, *J* = 7.5, 6.2 Hz, 2H), 2.15 (s, 3H), 2.08 (s, 3H), 2.02 (s, 3H), 1.75 – 1.63 (m, 2H), 0.94 (t, *J* = 7.4 Hz, 3H).

^{13}C NMR (101 MHz, $CDCl_3$) δ 173.5 (C_q), 170.4 (C_q), 170.3 (C_q), 169.8 (C_q), 164.4 (C_q), 157.4 (CH), 138.6 (C_q), 136.1 (C_q), 129.5 (CH), 128.8 (CH), 128.4 (CH), 126.7 (CH), 119.5 (CH), 75.4 (CH), 71.8 (CH), 69.7 (CH), 69.5 (CH), 67.2 (CH), 62.2 (CH_2), 36.1 (CH_2), 21.1 (CH_3), 20.9 (CH_3), 20.9 (CH_3), 18.4 (CH_2), 13.8 (CH_3).

IR (ATR): $\tilde{\nu}$ = 1737, 1555, 1411, 1368, 1214, 1047, 700 cm^{-1}.

MS (ESI) *m/z* (relative intensity): 537 (100) $[M+Na]^+$, 515 (40) $[M+H]^+$.

HR-MS (ESI): m/z calcd for $C_{26}H_{31}N_2O_9^+$ $[M+H]^+$ 515.2024, found 515.2020.

(2*R*,3*R*,4*R*,5*R*,6*R*)-2-((Pentanoyloxy)methyl)-6-(3-(pyrimidin-2-yl)phenyl)tetrahydro-2*H*-pyran-3,4,5-triyl-triacetate (35)

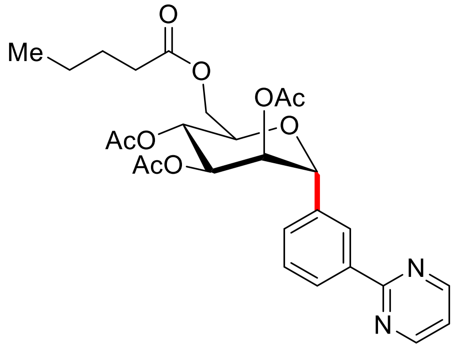

The general procedure was followed using 2-phenylpyrimidine (**1a**) (15.6 mg, 0.10 mmol), (2*R*,3*S*,4*S*,5*R*,6*R*)-2-bromo-6-((pentanoyloxy)methyl)tetrahydro-2*H*-pyran-3,4,5-triyl-triacetate (**2y**) (90.4 mg, 0.20 mmol), $[RuCl_2(p\text{-cymene})]_2$ (3.1 mg, 5.0 mol %), NaOAc (16.4 mg, 0.2 mmol) in 1,4-dioxane (1.0 mL) at ambient temperature. Purification by column chromatography on silica gel (*n*-hexane/EtOAc: 5/1 to 2/1) yielded **32** (20.60 mg, 39%) as a sticky solid.

^{1}H NMR (400 MHz, $CDCl_3$) δ 8.82 (d, J = 4.8 Hz, 2H), 8.62 (d, J = 2.0 Hz, 1H), 8.48 – 8.40 (m, 1H), 7.63 (dd, J = 7.6, 1.9 Hz, 1H), 7.56 (t, J = 7.7 Hz, 1H), 7.22 (t, J = 4.8 Hz, 1H), 6.02 (t, J = 3.4 Hz, 1H), 5.33 (t, J = 8.3 Hz, 1H), 5.23 (dd, J = 8.6, 3.1 Hz, 1H), 5.19 (d, J = 3.7 Hz, 1H), 4.42 (dd, J = 12.1, 6.8 Hz, 1H), 4.20 (dd, J = 12.0, 2.9 Hz, 1H), 3.88 (ddd, J = 8.0, 6.7, 2.9 Hz, 1H), 2.48 – 2.31 (m, 2H), 2.15 (s, 3H), 2.08 (s, 3H), 2.02 (s, 3H), 1.68 – 1.57 (m, 2H), 1.40 – 1.30 (m, 2H), 0.89 (t, J = 7.3 Hz, 3H).

^{13}C NMR (101 MHz, $CDCl_3$) δ 173.7 (C_q), 170.4 (C_q), 170.3 (C_q), 169.8 (C_q), 164.4 (C_q), 157.4 (CH), 138.6 (C_q), 136.1 (C_q), 129.5 (CH), 128.8 (CH), 128.4 (CH), 126.8 (CH), 119.5 (CH), 75.4 (CH), 71.8 (CH), 69.7 (CH), 69.5 (CH), 67.3 (CH), 62.2 (CH_2), 34.0 (CH_2), 27.0 (CH_2), 22.4 (CH_2), 21.1 (CH_3), 20.9 (CH_3), 20.9 (CH_3), 13.9 (CH_3).

IR (ATR): $\tilde{\nu}$ = 1746, 1556, 1369, 1221, 1170, 909, 731 cm^{-1}.

MS (ESI) *m/z* (relative intensity): 551 (100) $[M+Na]^+$, 529 (50) $[M+H]^+$.

HR-MS (ESI): m/z calcd for $C_{27}H_{33}N_2O_9^+$ $[M+H]^+$ 529.2181, found 529.2189.

References

(1) (a) Simonetti, M.; Cannas, D. M.; Just-Baringo, X.; Vitorica-Yrezabal, I. J.; Larrosa, I., Cyclometallated ruthenium catalyst enables late-stage directed arylation of pharmaceuticals. *Nat. Chem.* **2018**, *10*, 724-731; (b) McArthur, G.; Docherty, J. H.; Hareram, M. D.; Simonetti, M.; Vitorica-Yrezabal, I. J.; Douglas, J. J.; Larrosa, I., An air- and moisture-stable ruthenium precatalyst for diverse reactivity. *Nat. Chem.* **2024**, *16*, 1141-1150; (c) Korvorapun, K.; Kuniyil, R.; Ackermann, L., Late-Stage Diversification by Selectivity Switch in meta-C–H Activation: Evidence for Singlet Stabilization. *ACS Catal.* **2020**, *10*, 435-440.

(2) (a) Doyle, L. M.; O'Sullivan, S.; Di Salvo, C.; McKinney, M.; McArdle, P.; Murphy, P. V., Stereoselective Epimerizations of Glycosyl Thiols. *Org. Lett.* **2017**, *19*, 5802-5805; (b) Johnston, B. D.; Pinto, B. M., Synthesis of thio-linked disaccharides by 1-->2 intramolecular thioglycosyl migration: oxacarbenium versus episulfonium ion intermediates. *J. Org. Chem.* **2000**, *65*, 4607-17; (c) Zhao, G.; Yao, W.; Mauro, J. N.; Ngai, M.-Y., Excited-State Palladium-Catalyzed 1,2-Spin-Center Shift Enables Selective C-2 Reduction, Deuteration, and Iodination of Carbohydrates. *J. Am. Chem. Soc.* **2021**, *143*, 1728-1734; (d) Cicchillo, R. M.; Norris, P., A convenient synthesis of glycosyl chlorides from sugar hemiacetals using triphosgene as the chlorine source. *Carbohydrate Research* **2000**, *328*, 431-434.

(3) Wu, J.; Kaplaneris, N.; Pöhlmann, J.; Michiyuki, T.; Yuan, B.; Ackermann, L., Remote C−H Glycosylation by Ruthenium(II) Catalysis: Modular Assembly of meta-C-Aryl Glycosides. *Angew. Chem. Int. Ed.* **2022**, *61*, e202208620.

(4) Gandeepan, P.; Koeller, J.; Korvorapun, K.; Mohr, J.; Ackermann, L., Visible-Light-Enabled Ruthenium-Catalyzed meta-C−H Alkylation at Room Temperature. *Angew. Chem. Int. Ed.* **2019**, *58*, 9820-9825.

(5) (a) Michiyuki, T.; Maksso, I.; Ackermann, L., Photo-Induced Ruthenium-Catalyzed C−H Arylation Polymerization at Ambient Temperature. *Angew. Chem. Int. Ed.* **2024**, *63*, e202400845; (b) Korvorapun, K.; Struwe, J.; Kuniyil, R.; Zangarelli, A.; Casnati, A.; Waeterschoot, M.; Ackermann, L., Photo-Induced Ruthenium-Catalyzed C−H Arylations at Ambient Temperature. *Angew. Chem. Int. Ed.* **2020**, *59*, 18103-18109; (c) Cismesia, M. A.; Yoon, T. P., Characterizing chain processes in visible light photoredox catalysis. *Chem. Sci.* **2015**, *6*, 5426-5434.

(6) Gaussian 16, R. A., M. J. Frisch, G. W. Trucks, H. B. Schlegel, G. E. Scuseria, M. A. Robb, J. R. Cheeseman, G. Scalmani, V. Barone, G. A. Petersson, H. Nakatsuji, X. Li, M. Caricato, A. V. Marenich, J. Bloino, B. G. Janesko, R. Gomperts, B. Mennucci, H. P. Hratchian, J. V. Ortiz, A. F. Izmaylov, J. L. Sonnenberg, D. Williams-Young, F. Ding, F. Lipparini, F. Egidi, J. Goings, B. Peng, A. Petrone, T. Henderson, D. Ranasinghe, V. G. Zakrzewski, J. Gao, N. Rega, G. Zheng, W. Liang, M. Hada, M. Ehara, K. Toyota, R. Fukuda, J. Hasegawa, M. Ishida, T. Nakajima, Y. Honda, O. Kitao, H. Nakai, T. Vreven, K. Throssell, J. A. Montgomery, Jr., J. E. Peralta, F. Ogliaro, M. J. Bearpark, J. J. Heyd, E. N. Brothers, K. N. Kudin, V. N. Staroverov, T. A. Keith, R. Kobayashi, J. Normand, K. Raghavachari, A. P. Rendell, J. C. Burant, S. S. Iyengar, J. Tomasi, M. Cossi, J. M. Millam, M. Klene, C. Adamo, R. Cammi, J. W. Ochterski, R. L. Martin, K. Morokuma, O. Farkas, J. B. Foresman, D. J. Fox, Gaussian, Inc., Wallingford CT, **2016**.

(7) (a) Adamo, C.; Barone, V., Toward reliable density functional methods without adjustable parameters: The PBE0 model. *J. Chem. Phys.* **1999**, *110*, 6158-6170; (b) Ernzerhof, M.; Scuseria, G. E., Assessment of the Perdew–Burke–Ernzerhof exchange-correlation functional. *J. Chem. Phys.* **1999**, *110*, 5029-5036.

(8) (a) Grimme, S.; Ehrlich, S.; Goerigk, L., Effect of the damping function in dispersion corrected density functional theory. *J. Comput. Chem.* **2011**, *32*, 1456-1465; (b) Grimme, S.; Antony, J.; Ehrlich, S.; Krieg, H., A consistent and accurate ab initio parametrization of density functional dispersion correction (DFT-D) for the 94 elements H-Pu. *J. Chem. Phys.* **2010**, *132*.

(9) (a) Weigend, F., Accurate Coulomb-fitting basis sets for H to Rn. *Phys. Chem. Chem. Phys.* **2006**, *8*, 1057-1065; (b) Weigend, F.; Ahlrichs, R., Balanced basis sets of split valence, triple zeta valence and quadruple zeta valence quality for H to Rn: Design and assessment of accuracy. *Phys. Chem. Chem. Phys.* **2005**, *7*, 3297-3305; (c) Schäfer, A.; Huber, C.; Ahlrichs, R., Fully optimized contracted Gaussian

basis sets of triple zeta valence quality for atoms Li to Kr. *J. Chem. Phys.* **1994**, *100*, 5829-5835; (d) Schäfer, A.; Horn, H.; Ahlrichs, R., Fully optimized contracted Gaussian basis sets for atoms Li to Kr. *J. Chem. Phys.* **1992**, *97*, 2571-2577.
(10) (a) Martin, J. M. L.; Sundermann, A., Correlation consistent valence basis sets for use with the Stuttgart – Dresden – Bonn relativistic effective core potentials: The atoms Ga – Kr and In – Xe. *J. Chem. Phys.* **2001**, *114*, 3408-3420; (b) Dolg, M.; Wedig, U.; Stoll, H.; Preuss, H., Energy - adjusted ab initio pseudopotentials for the first row transition elements. *J. Chem. Phys.* **1987**, *86*, 866-872.
(11) (a) Caldeweyher, E.; Ehlert, S.; Hansen, A.; Neugebauer, H.; Spicher, S.; Bannwarth, C.; Grimme, S., A generally applicable atomic-charge dependent London dispersion correction. *J. Chem. Phys.* **2019**, *150*; (b) Caldeweyher, E.; Bannwarth, C.; Grimme, S., Extension of the D3 dispersion coefficient model. *J. Chem. Phys.* **2017**, *147*, 034112.
(12) Marenich, A. V.; Cramer, C. J.; Truhlar, D. G., Universal Solvation Model Based on Solute Electron Density and on a Continuum Model of the Solvent Defined by the Bulk Dielectric Constant and Atomic Surface Tensions. *J. Phys. Chem. B* **2009**, *113*, 6378-6396.
(13) C. Y. Legault, C., 1.0b; Université de Sherbrooke: (Quebec) Canada, http://www.cylview.org.

^{1}H and ^{13}C NMR Spectra

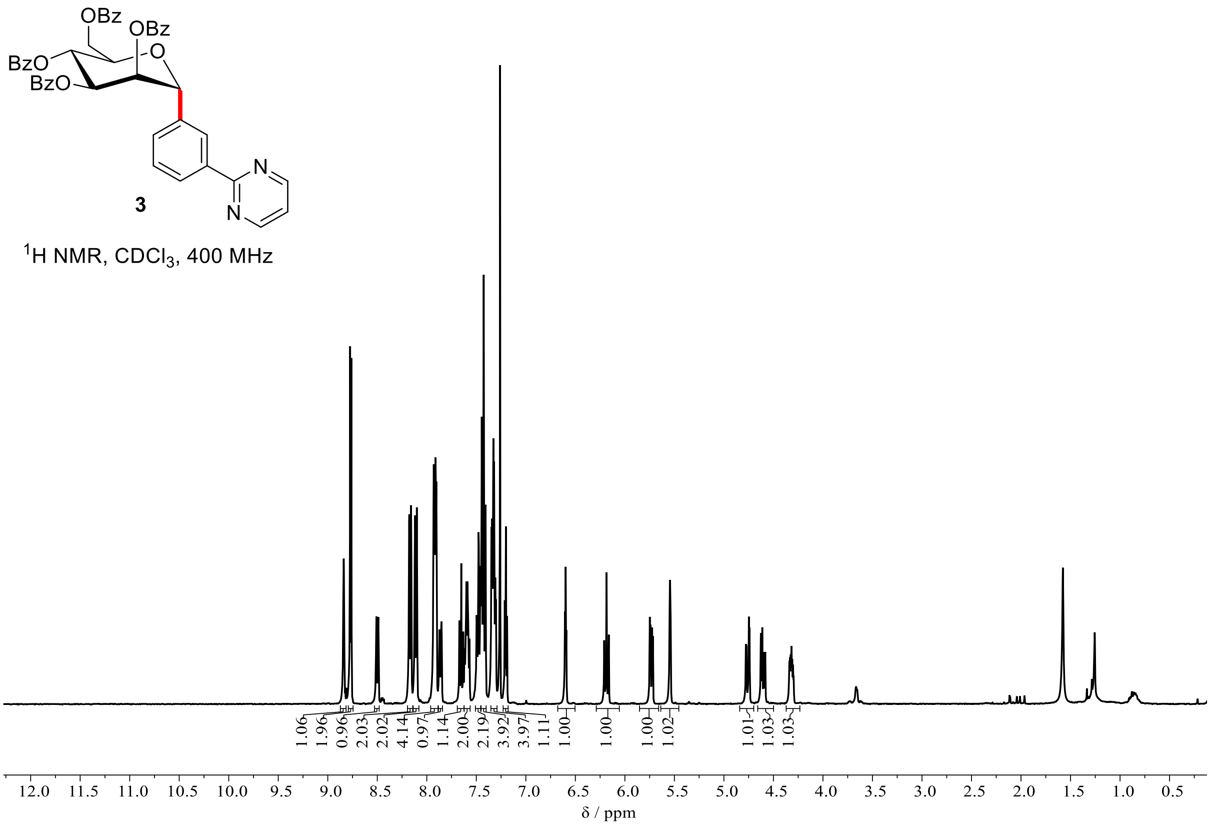

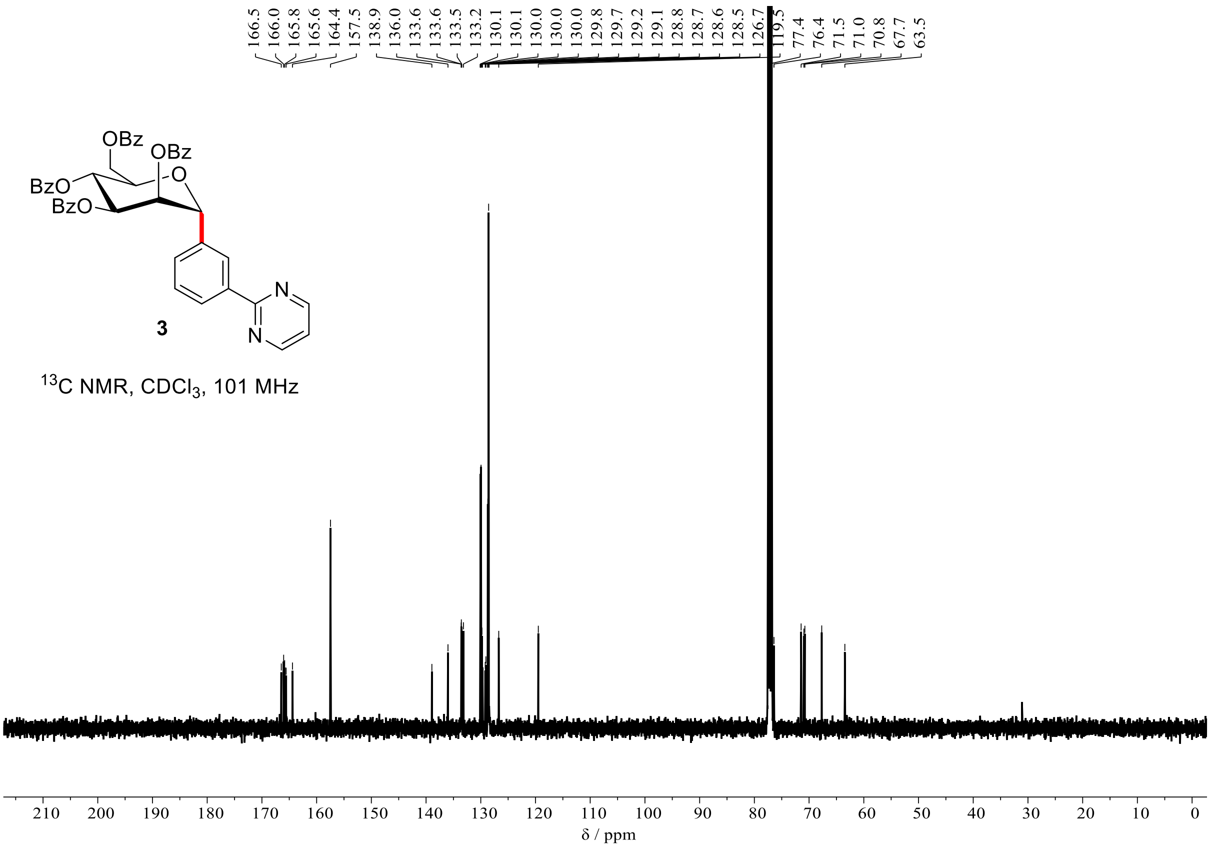

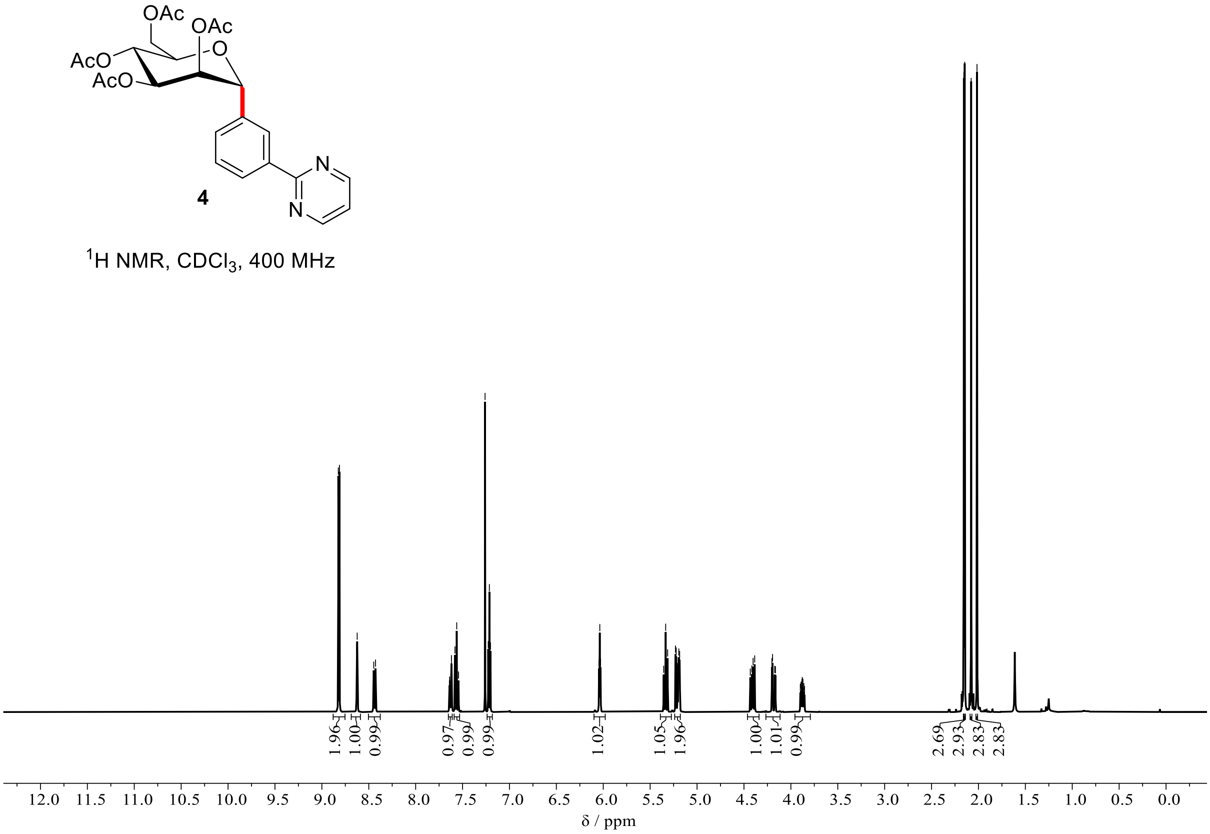
OAc
OAc
AcO
AcO
O
N
N
4
1H NMR, CDCl3, 400 MHz
1.96
1.00
0.99
0.97
0.99
0.99
1.02
1.05
1.96
1.00
1.01
0.99
2.69
2.93
2.83
2.83
δ / ppm

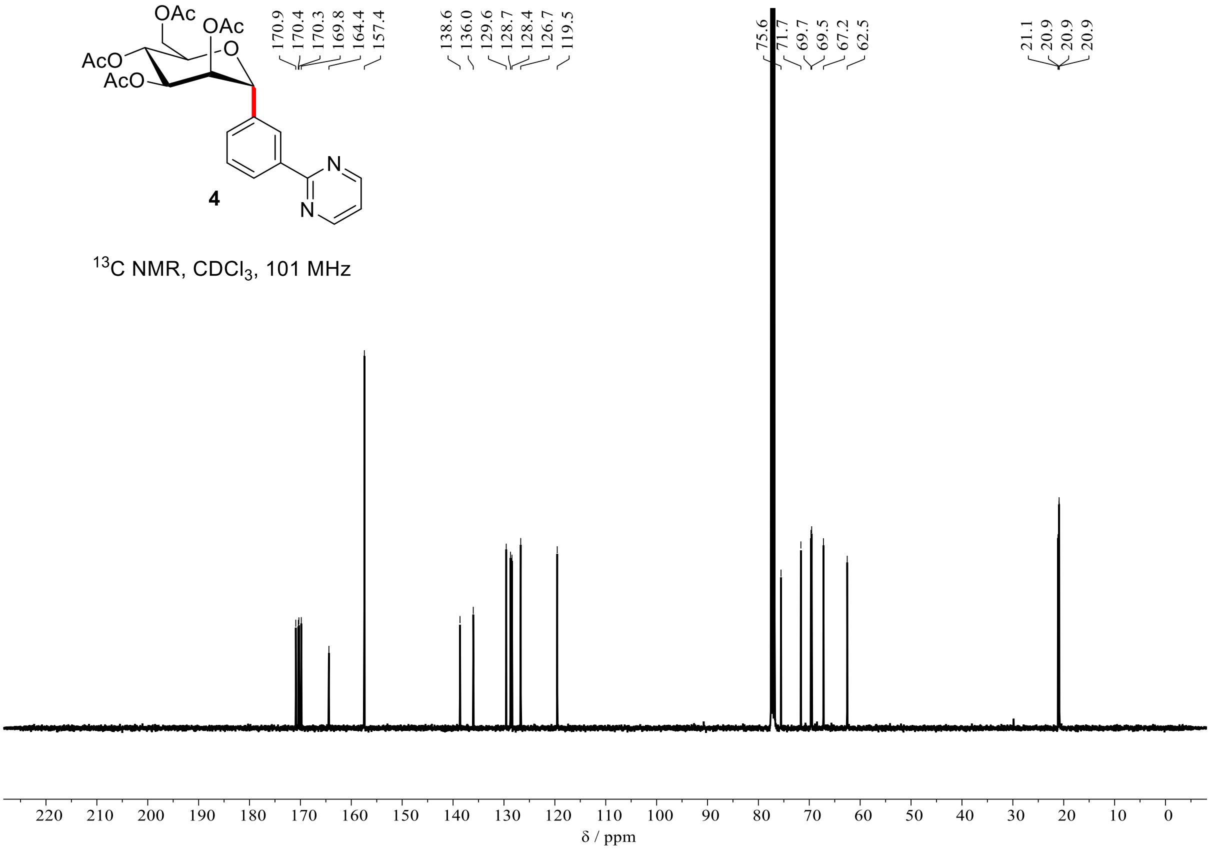
OAc
OAc
AcO
AcO
O
N
N
4
13C NMR, CDCl3, 101 MHz
170.9
170.4
170.3
169.8
164.4
157.4
138.6
136.0
129.6
128.7
128.4
126.7
119.5
75.6
71.7
69.7
69.5
67.2
62.5
21.1
20.9
20.9
20.9
δ / ppm

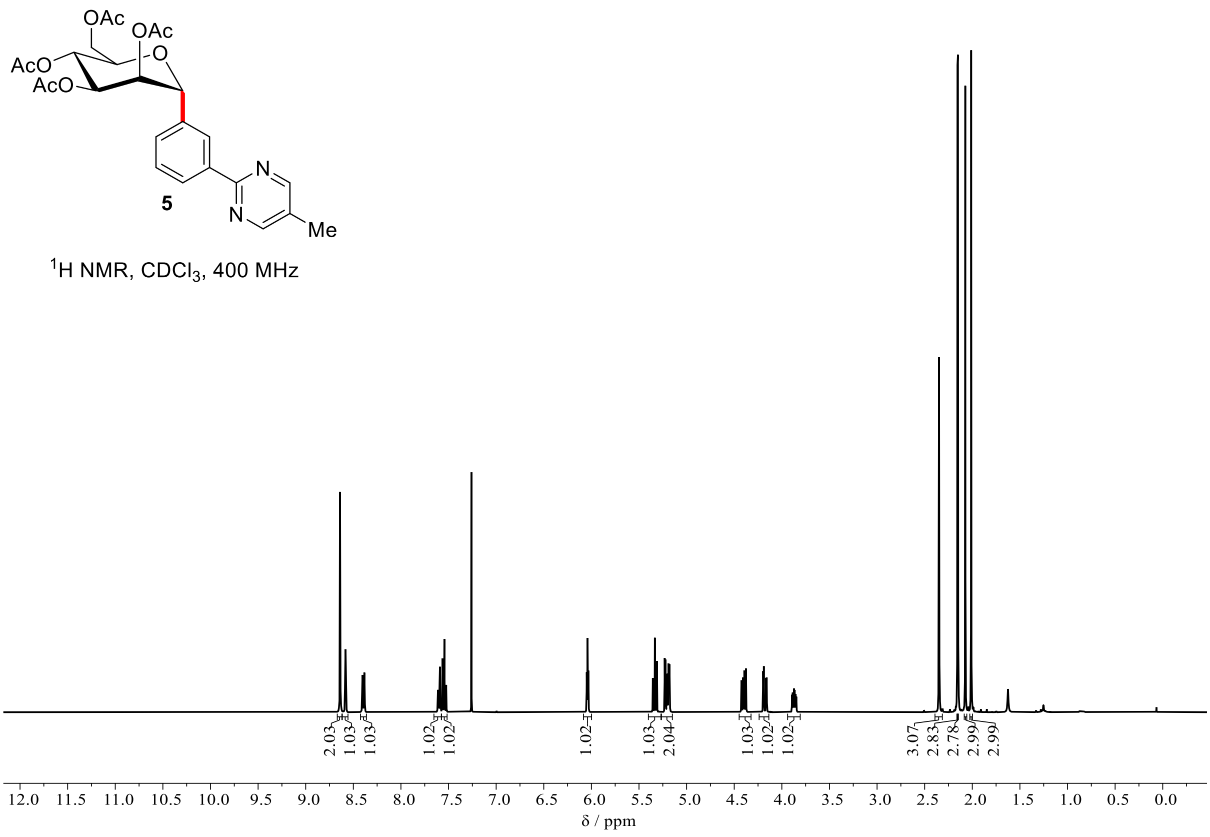

OAc
OAc
AcO
AcO
O
N
N
Me
5
^{1}H NMR, CDCl$_3$, 400 MHz
2.03 1.03 1.03 1.02 1.02 1.02 1.03 2.04 1.03 1.02 1.02 3.07 2.83 2.78 2.99 2.99
12.0 11.5 11.0 10.5 10.0 9.5 9.0 8.5 8.0 7.5 7.0 6.5 6.0 5.5 5.0 4.5 4.0 3.5 3.0 2.5 2.0 1.5 1.0 0.5 0.0
δ / ppm

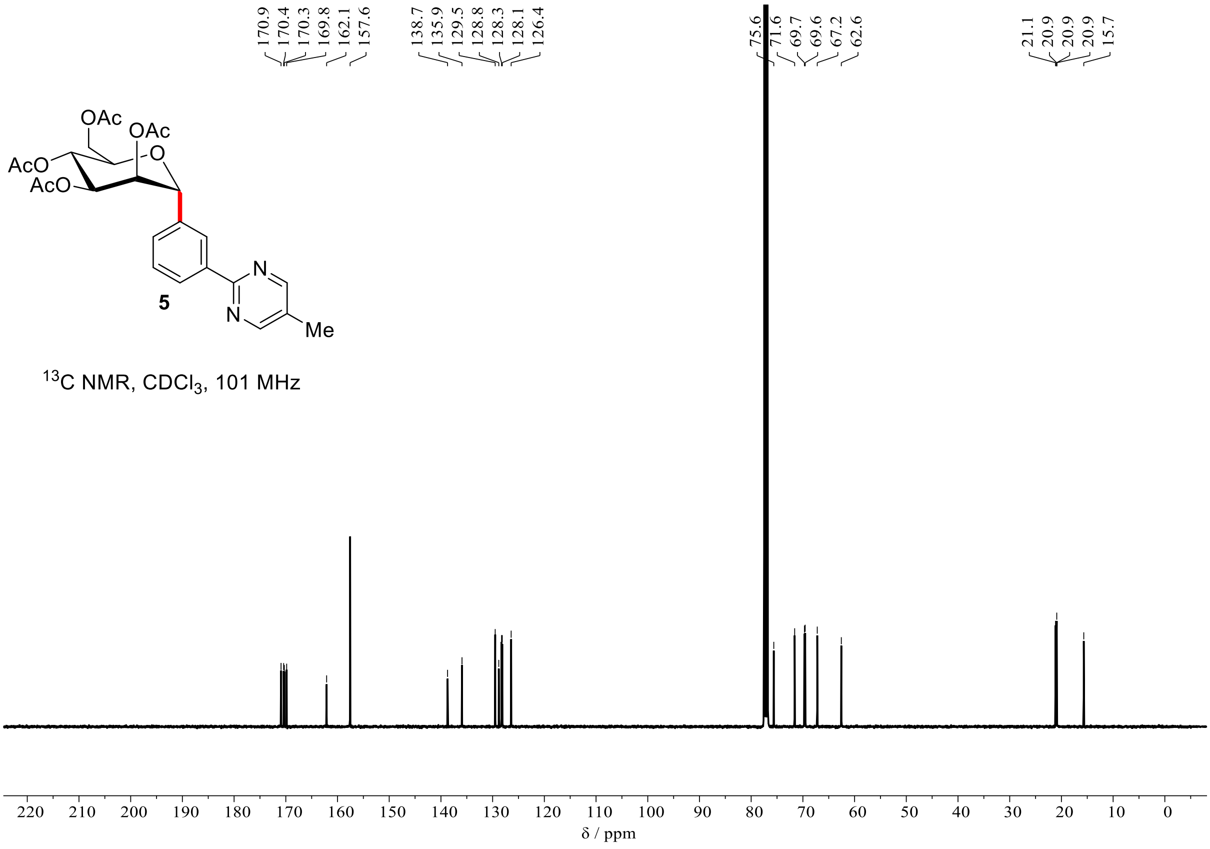

170.9 170.4 170.3 169.8 162.1 157.6 138.7 135.9 129.5 128.8 128.3 128.1 126.4 75.6 71.6 69.7 69.6 67.2 62.6 21.1 20.9 20.9 20.9 15.7
OAc
OAc
AcO
AcO
O
N
N
Me
5
^{13}C NMR, CDCl$_3$, 101 MHz
220 210 200 190 180 170 160 150 140 130 120 110 100 90 80 70 60 50 40 30 20 10 0
δ / ppm

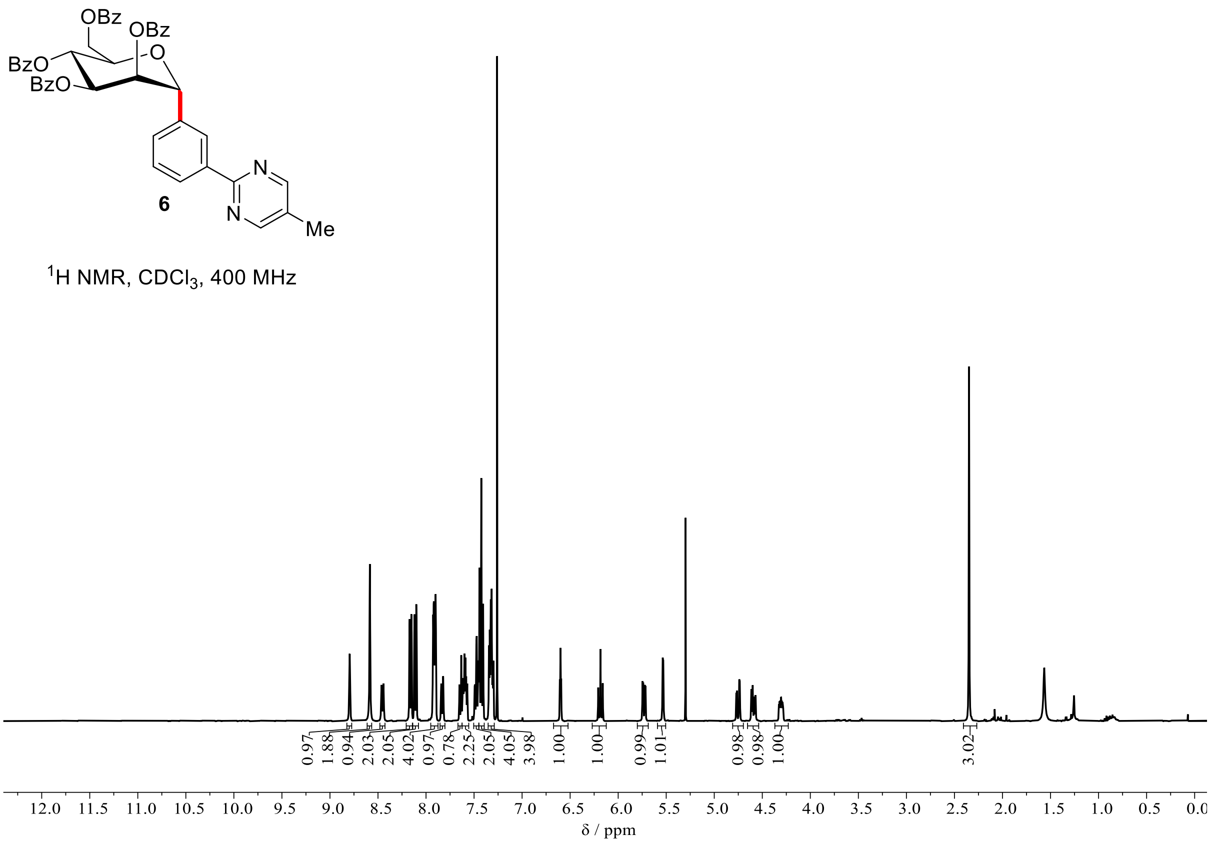
OBz
OBz
BzO
BzO
O
N
N
6
Me
1H NMR, CDCl3, 400 MHz
0.97
1.88
0.94
2.03
2.05
4.02
0.97
0.78
2.25
2.05
4.05
3.98
1.00
1.00
0.99
1.01
0.98
0.98
1.00
3.02
12.0 11.5 11.0 10.5 10.0 9.5 9.0 8.5 8.0 7.5 7.0 6.5 6.0 5.5 5.0 4.5 4.0 3.5 3.0 2.5 2.0 1.5 1.0 0.5 0.0
δ / ppm

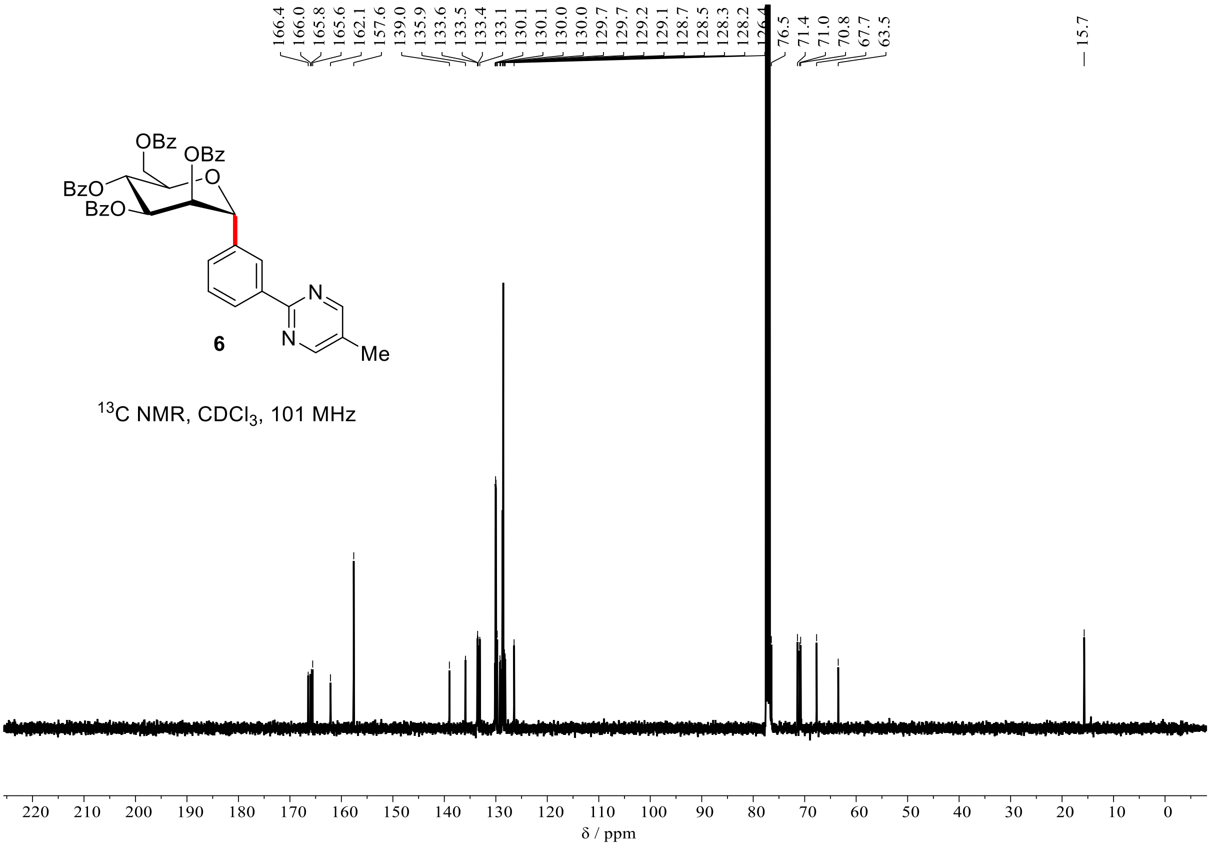
166.4
166.0
165.8
165.6
162.1
157.6
139.0
135.9
133.6
133.5
133.4
133.1
130.1
130.1
130.0
130.0
129.7
129.7
129.2
129.1
128.7
128.5
128.3
128.2
126.4
76.5
71.4
71.0
70.8
67.7
63.5
15.7
OBz
OBz
BzO
BzO
O
N
N
6
Me
13C NMR, CDCl3, 101 MHz
220 210 200 190 180 170 160 150 140 130 120 110 100 90 80 70 60 50 40 30 20 10 0
δ / ppm

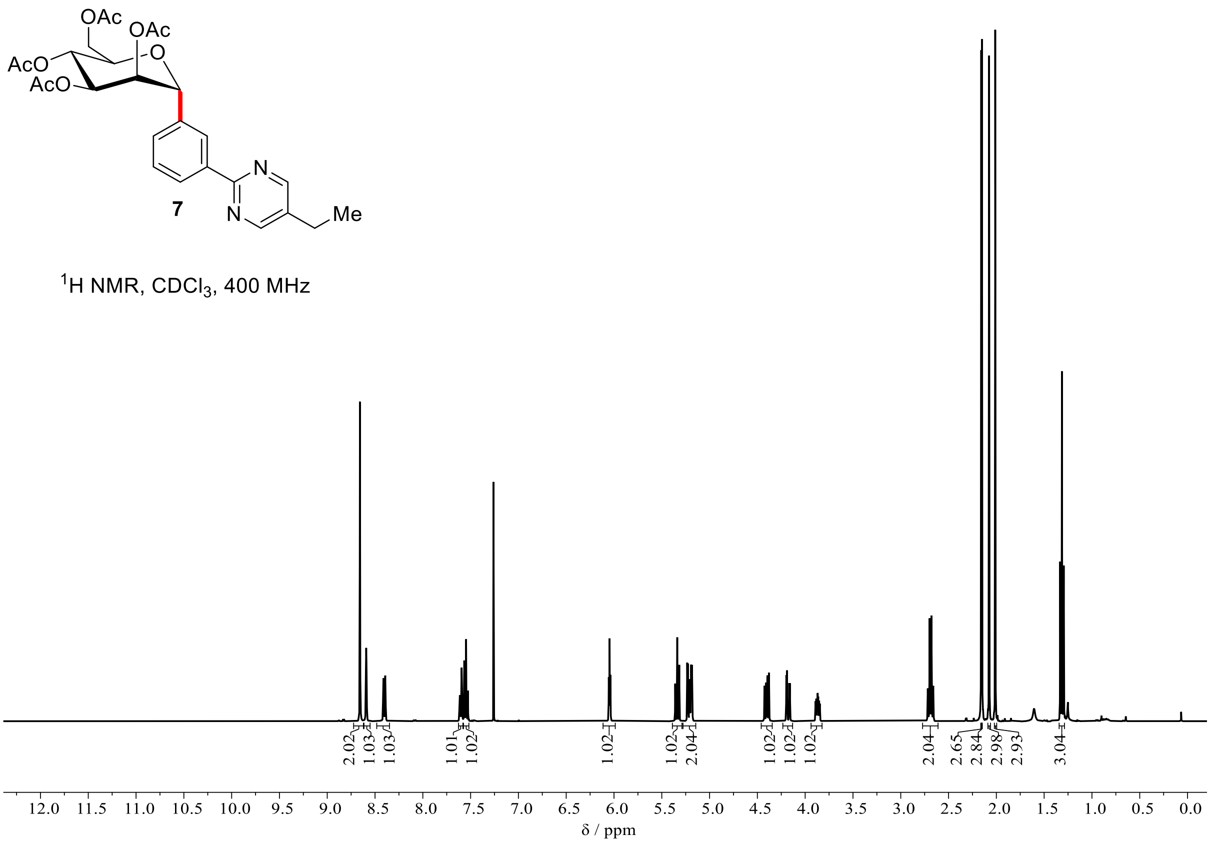
OAc
OAc
AcO
AcO
O
N
N
7
Me
1H NMR, CDCl3, 400 MHz
2.02
1.03
1.03
1.01
1.02
1.02
1.02
2.04
1.02
1.02
1.02
2.04
2.65
2.84
2.98
2.93
3.04
12.0 11.5 11.0 10.5 10.0 9.5 9.0 8.5 8.0 7.5 7.0 6.5 6.0 5.5 5.0 4.5 4.0 3.5 3.0 2.5 2.0 1.5 1.0 0.5 0.0
δ / ppm

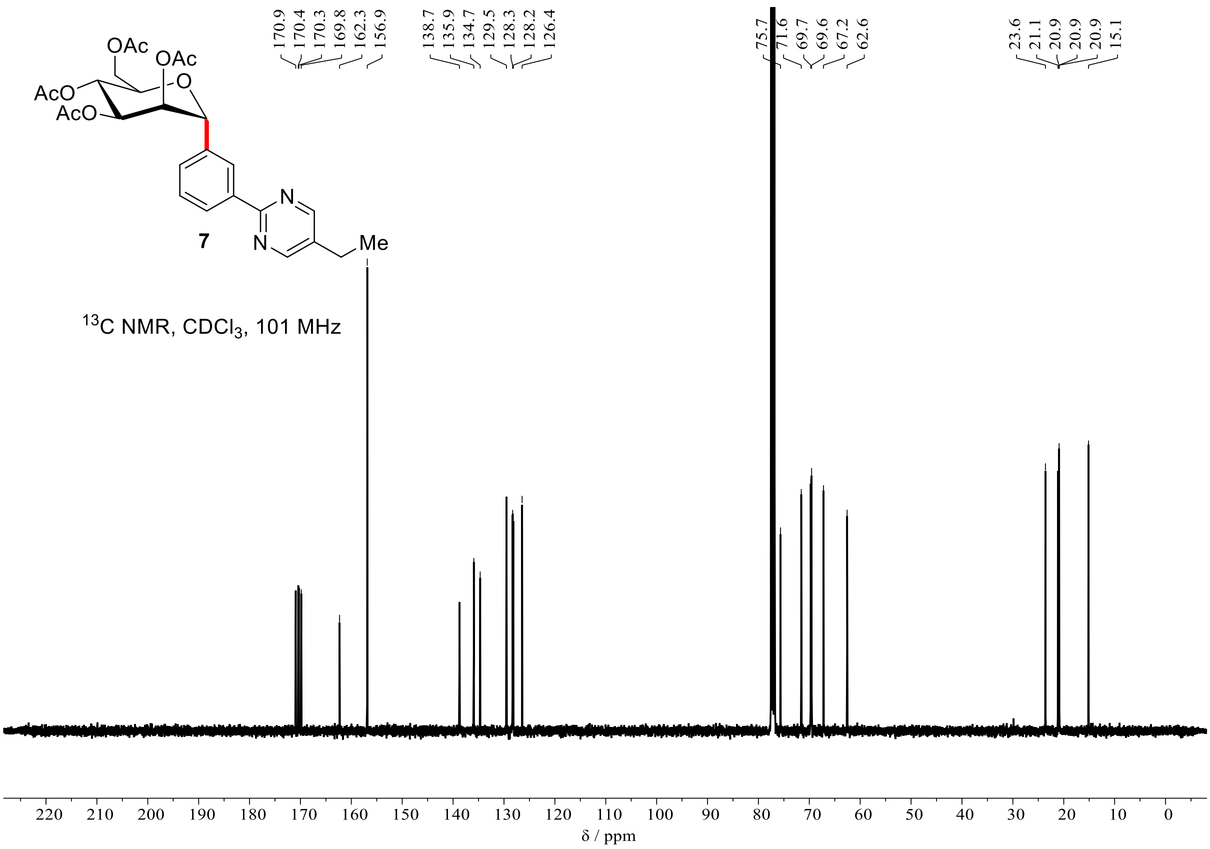
170.9
170.4
170.3
169.8
162.3
156.9
138.7
135.9
134.7
129.5
128.3
128.2
126.4
75.7
71.6
69.7
69.6
67.2
62.6
23.6
21.1
20.9
20.9
20.9
15.1
OAc
OAc
AcO
AcO
O
N
N
7
Me
13C NMR, CDCl3, 101 MHz
220 210 200 190 180 170 160 150 140 130 120 110 100 90 80 70 60 50 40 30 20 10 0
δ / ppm

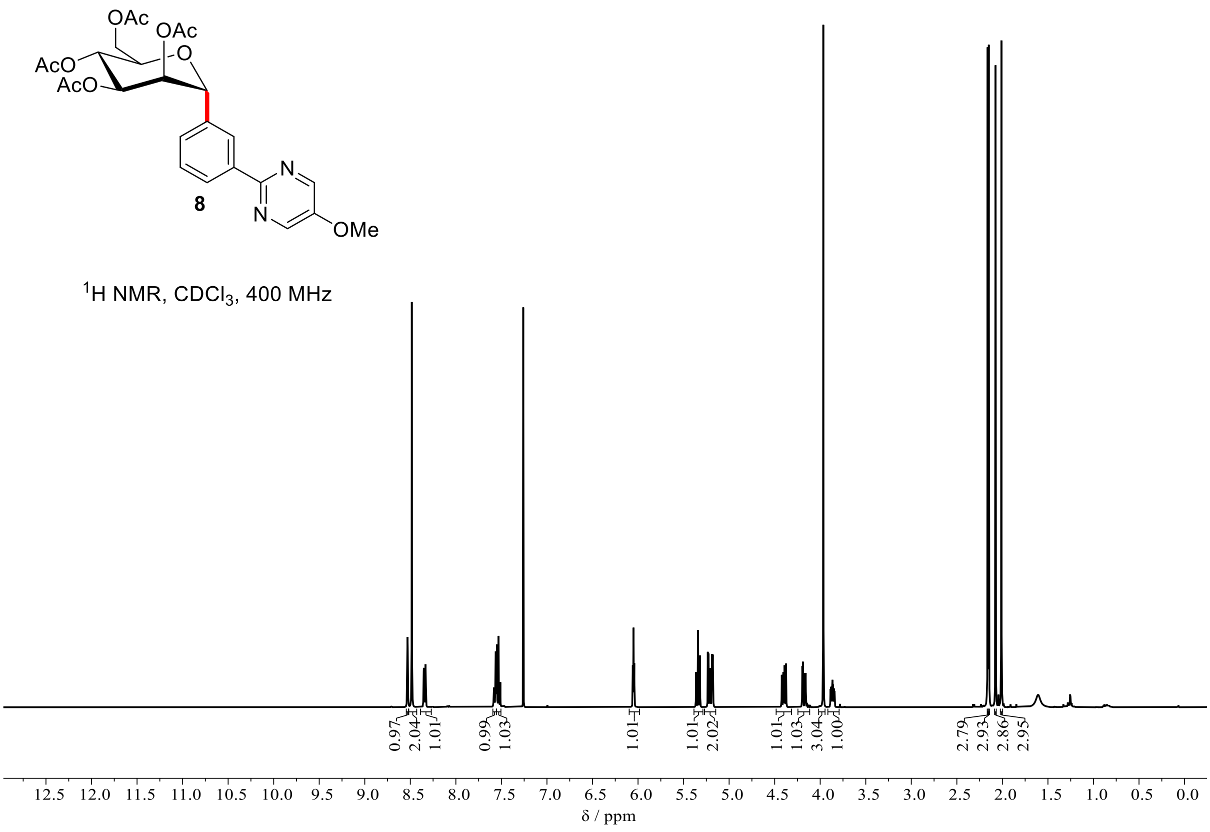
OAc
OAc
AcO
AcO
O
N
N
8
OMe
^{1}H NMR, CDCl$_3$, 400 MHz
0.97
2.04
1.01
0.99
1.03
1.01
1.01
2.02
1.01
1.03
3.04
1.00
2.79
2.93
2.86
2.95
δ / ppm

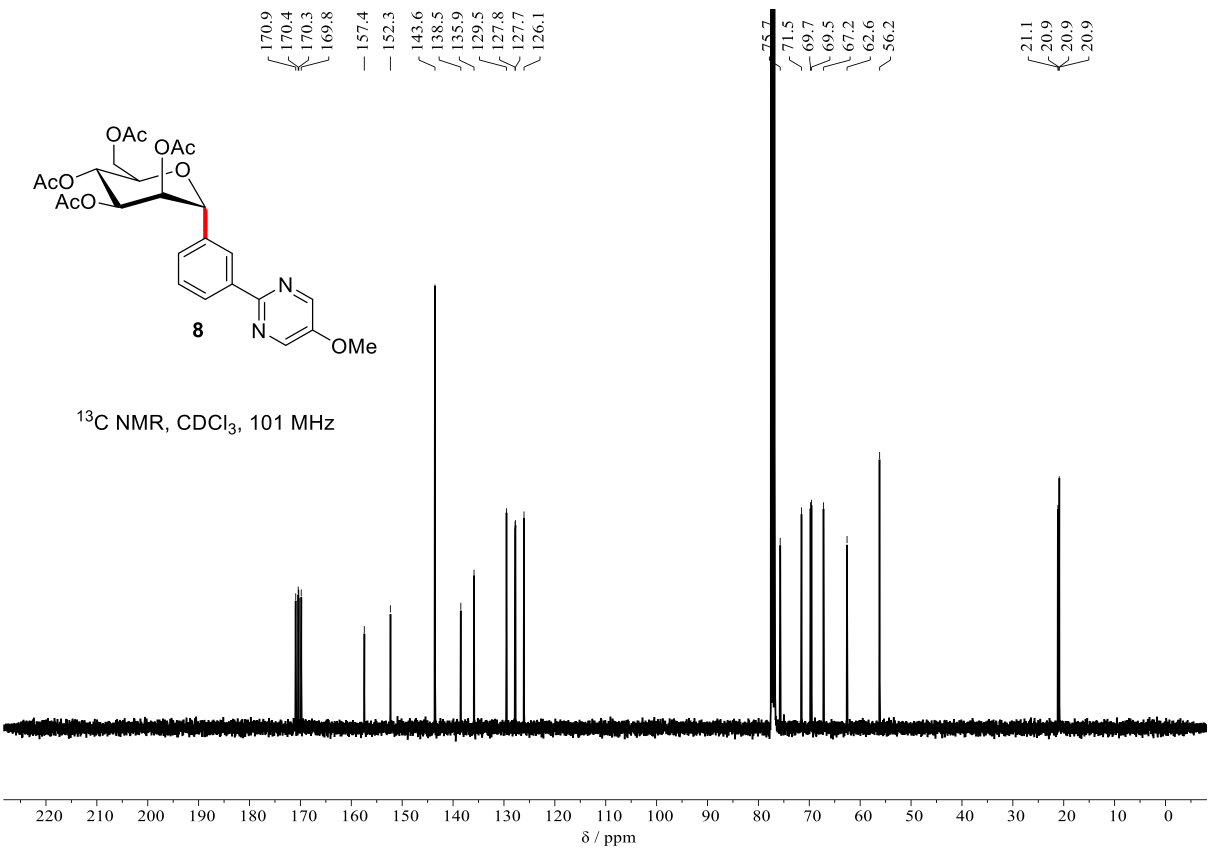
170.9
170.4
170.3
169.8
157.4
152.3
143.6
138.5
135.9
129.5
127.8
127.7
126.1
75.7
71.5
69.7
69.5
67.2
62.6
56.2
21.1
20.9
20.9
20.9
8
OMe
^{13}C NMR, CDCl$_3$, 101 MHz
δ / ppm

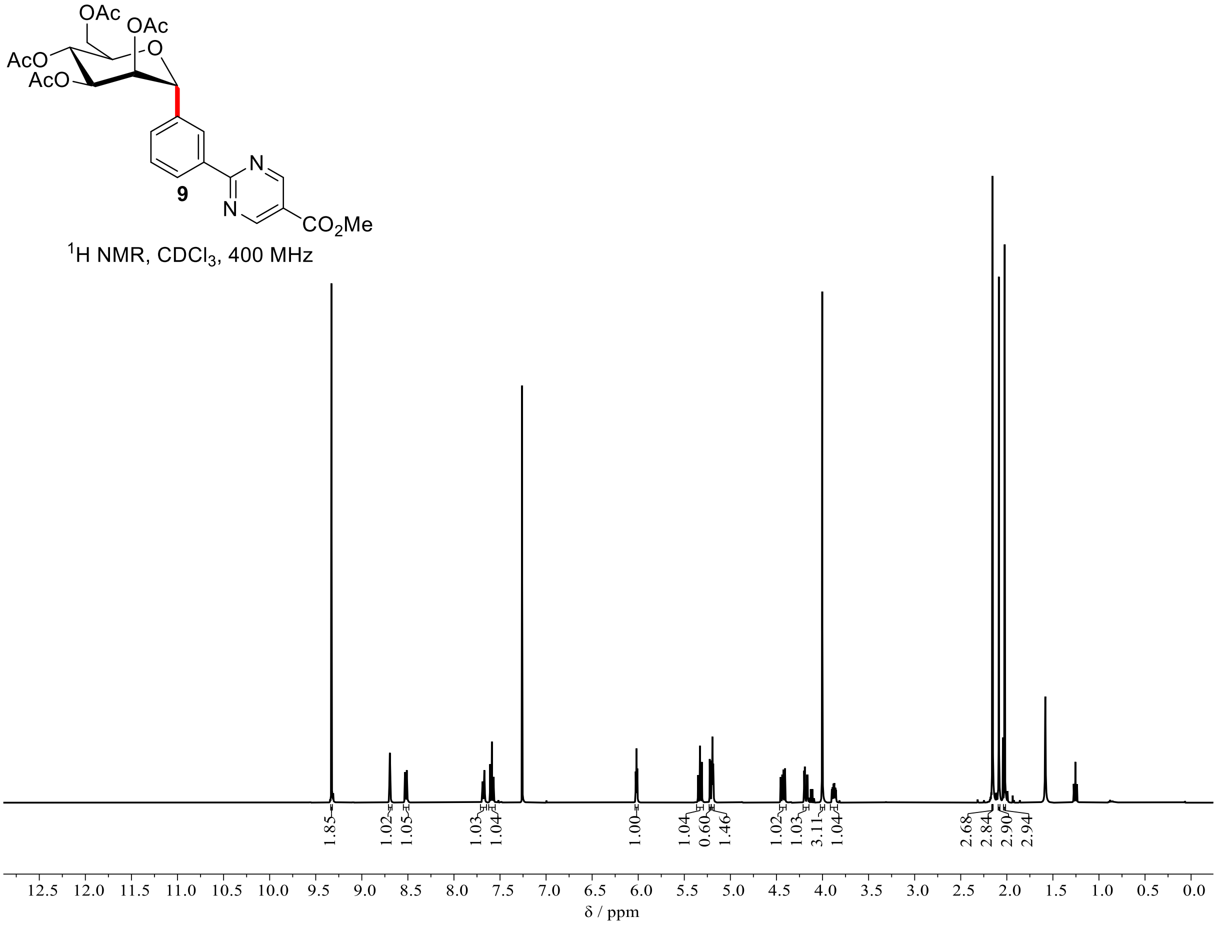

^{1}H NMR, $CDCl_3$, 400 MHz

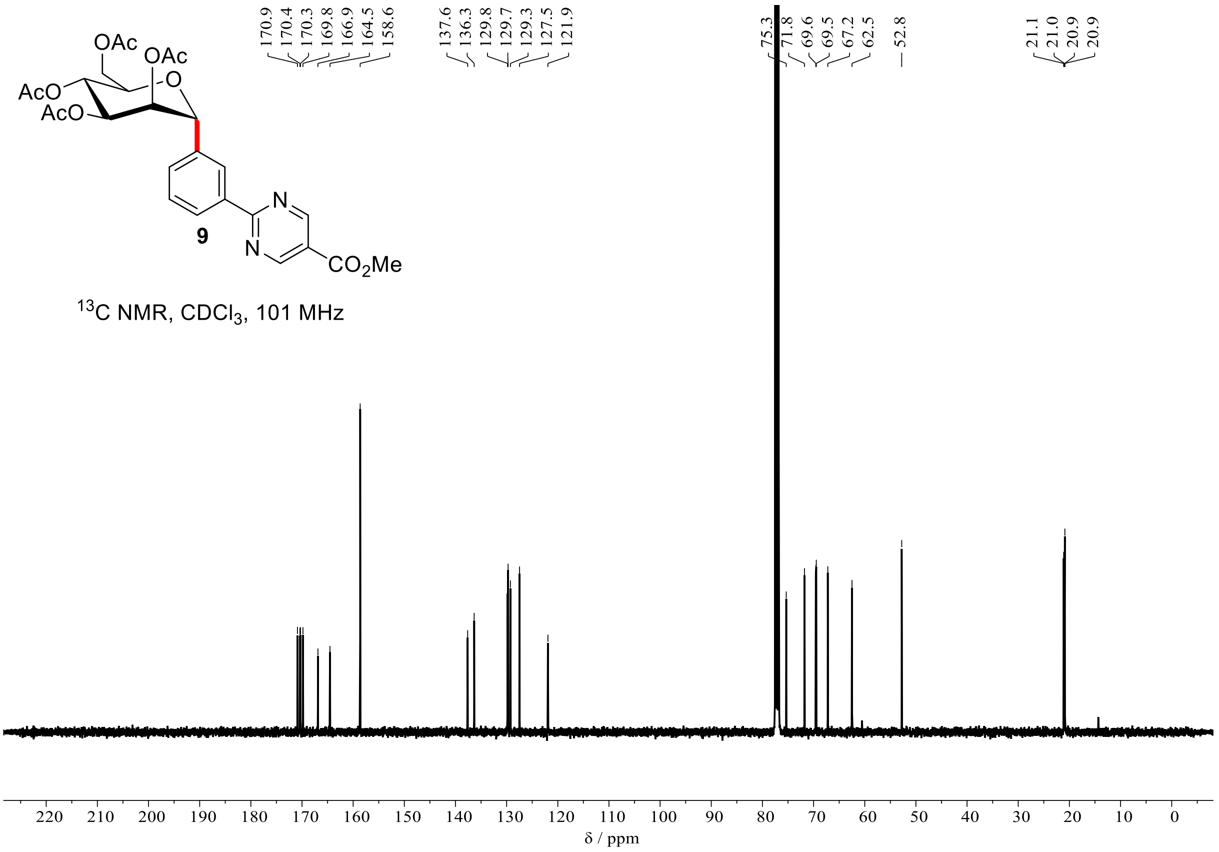

^{13}C NMR, $CDCl_3$, 101 MHz

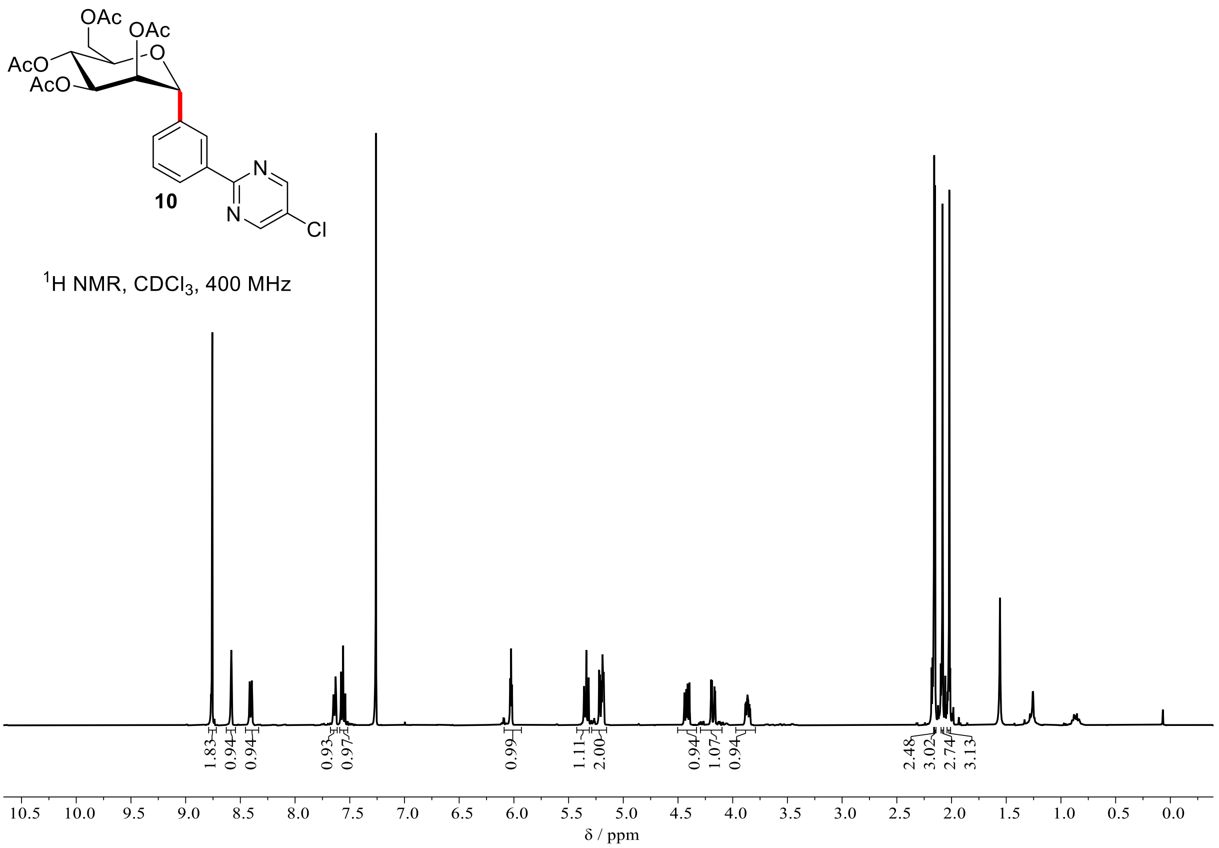
OAc
OAc
AcO
AcO
O
N
N
Cl
10
^{1}H NMR, CDCl$_3$, 400 MHz
1.83
0.94
0.94
0.93
0.97
0.99
1.11
2.00
0.94
1.07
0.94
2.48
3.02
2.74
3.13
10.5 10.0 9.5 9.0 8.5 8.0 7.5 7.0 6.5 6.0 5.5 5.0 4.5 4.0 3.5 3.0 2.5 2.0 1.5 1.0 0.5 0.0
δ / ppm

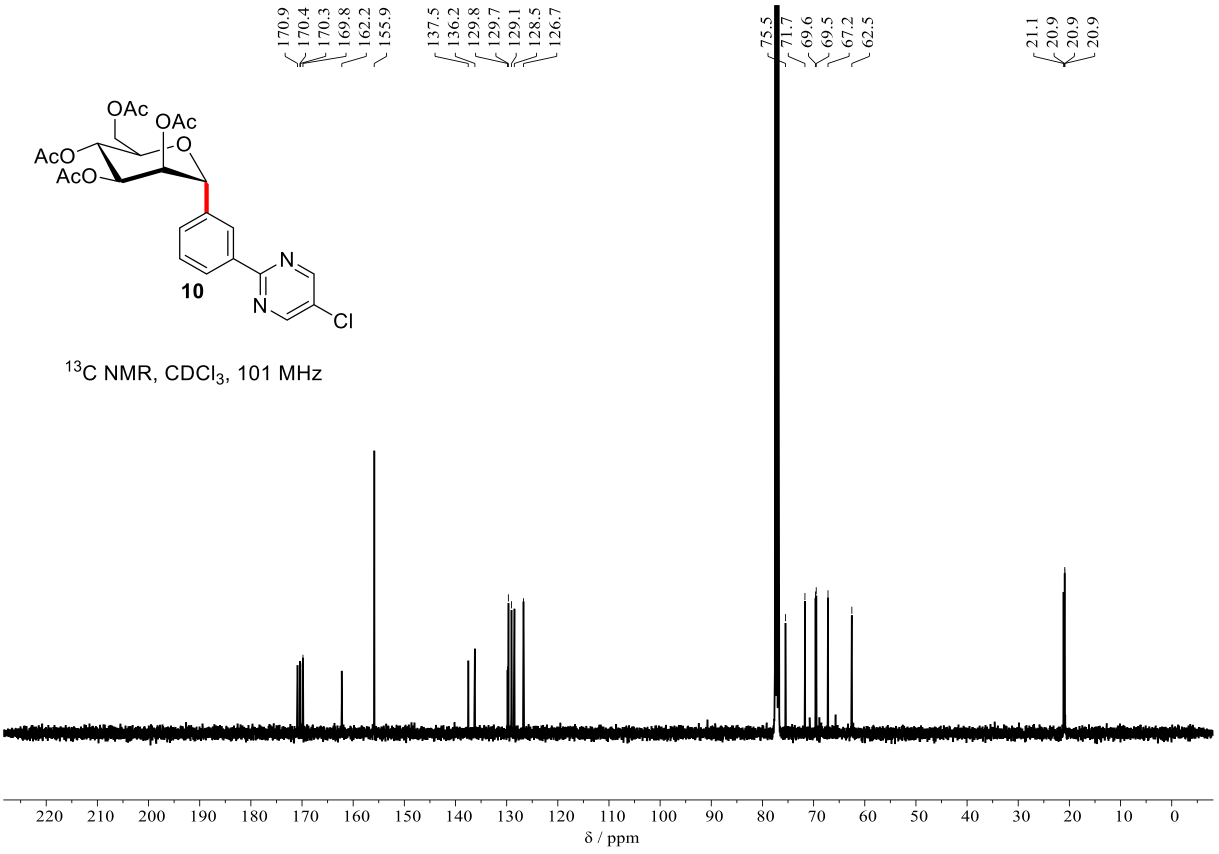
170.9
170.4
170.3
169.8
162.2
155.9
137.5
136.2
129.8
129.7
129.1
128.5
126.7
75.5
71.7
69.6
69.5
67.2
62.5
21.1
20.9
20.9
20.9
OAc
OAc
AcO
AcO
O
N
N
Cl
10
^{13}C NMR, CDCl$_3$, 101 MHz
220 210 200 190 180 170 160 150 140 130 120 110 100 90 80 70 60 50 40 30 20 10 0
δ / ppm

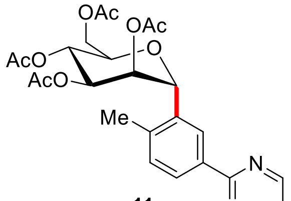

11

^{1}H NMR, $CDCl_3$, 600 MHz

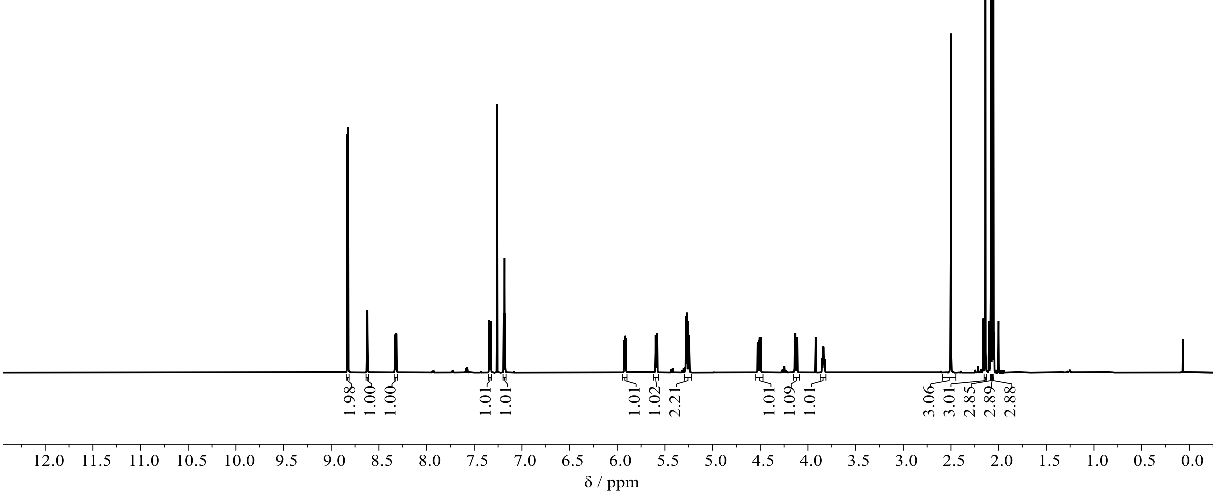

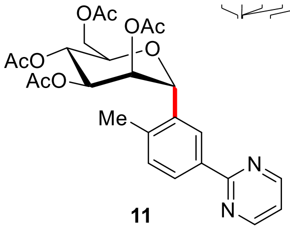

11

^{13}C NMR, $CDCl_3$, 126 MHz

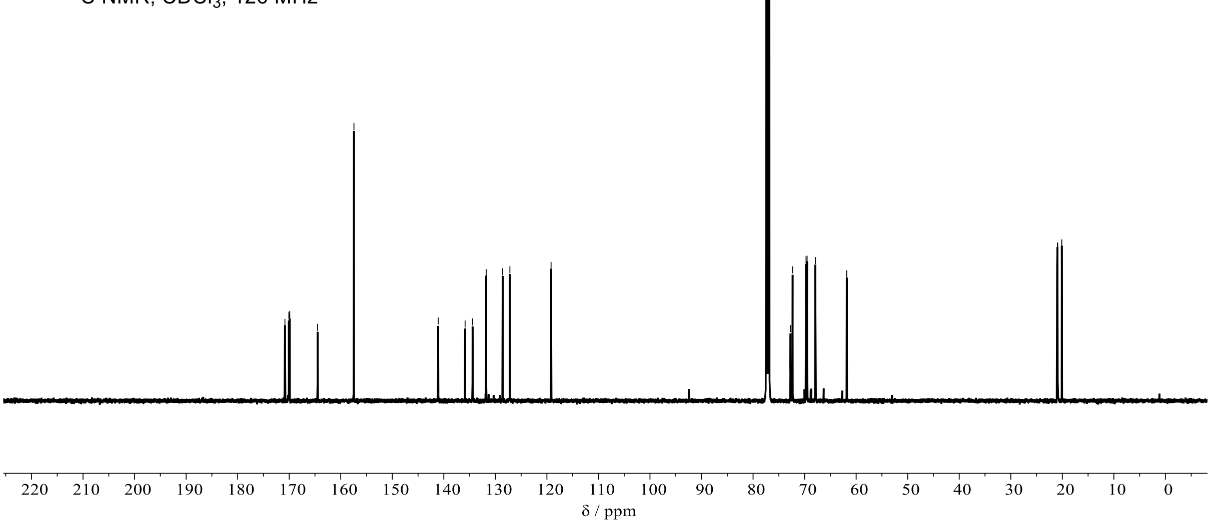

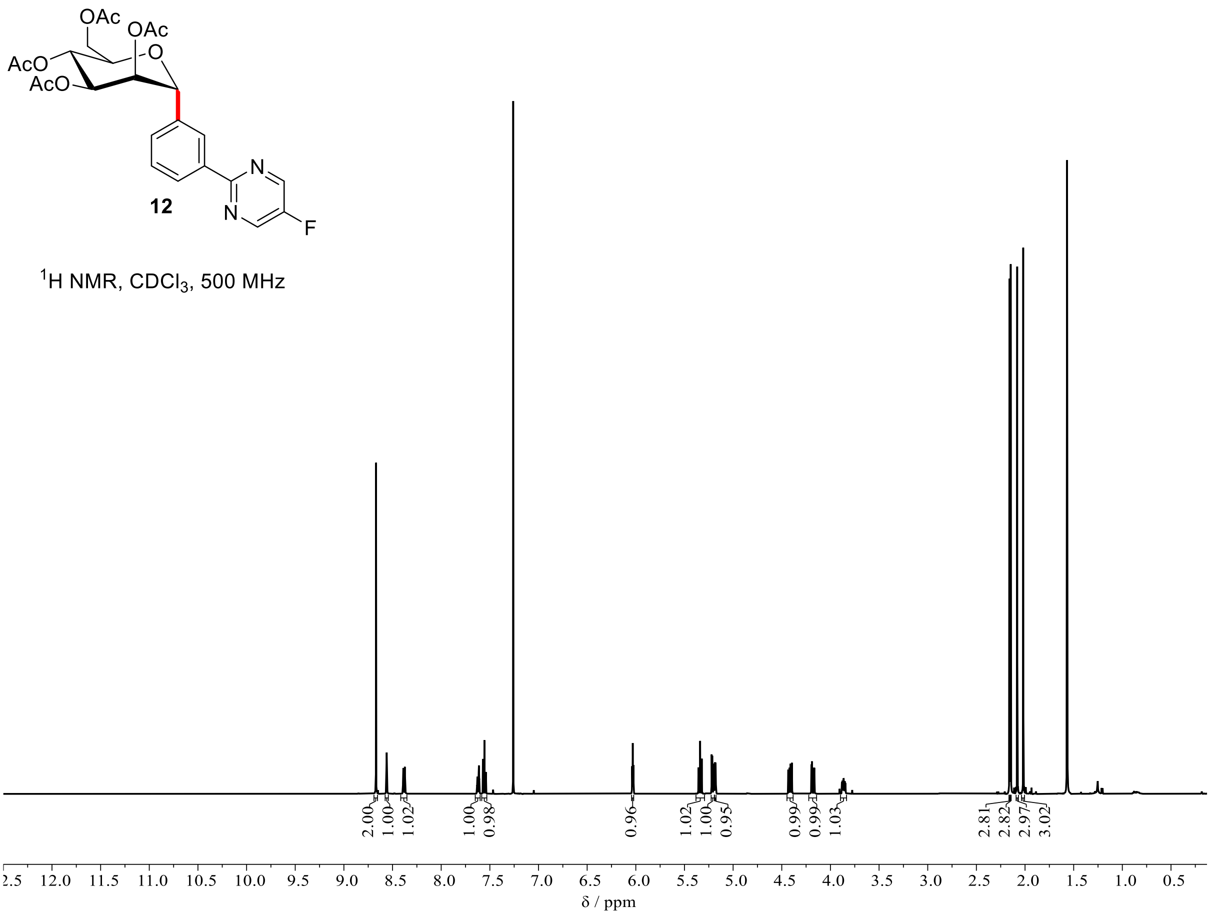
OAc
OAc
AcO
AcO
O
N
N
F
12
1H NMR, CDCl3, 500 MHz
2.00
1.00
1.02
1.00
0.98
0.96
1.02
1.00
0.95
0.99
0.99
1.03
2.81
2.82
2.97
3.02
δ / ppm

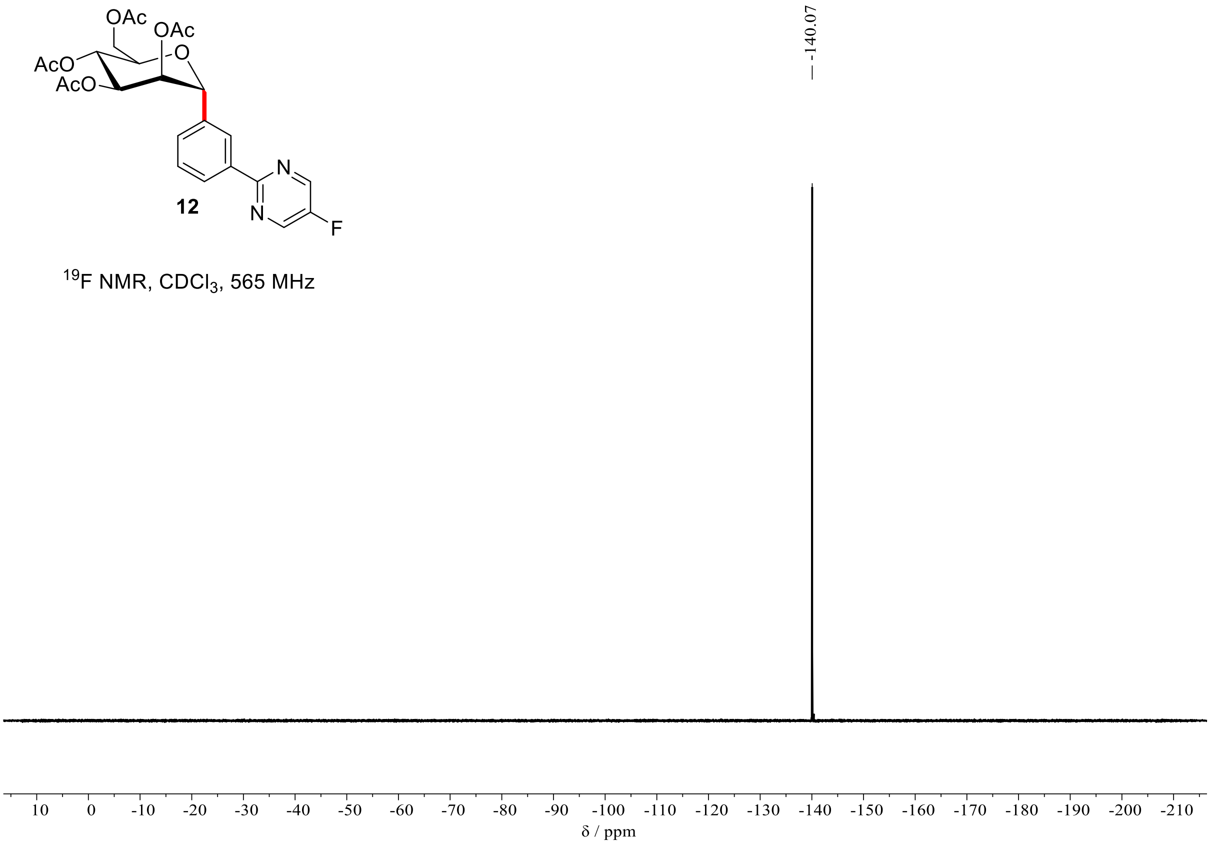
OAc
OAc
AcO
AcO
O
N
N
F
12
19F NMR, CDCl3, 565 MHz
-140.07
δ / ppm

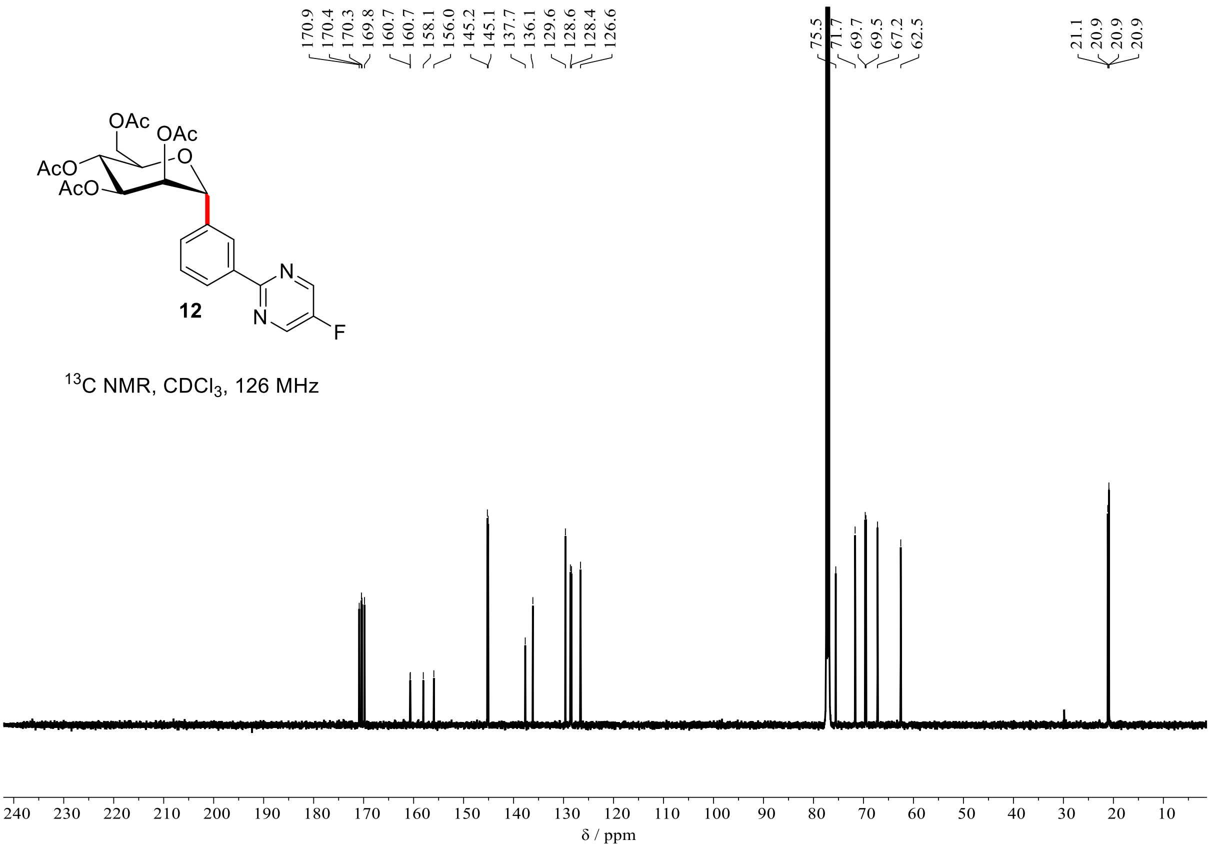
170.9
170.4
170.3
169.8
160.7
160.7
158.1
156.0
145.2
145.1
137.7
136.1
129.6
128.6
128.4
126.6
75.5
71.7
69.7
69.5
67.2
62.5
21.1
20.9
20.9
20.9
OAc
OAc
AcO
AcO
O
N
N
F
12
^{13}C NMR, $CDCl_3$, 126 MHz
240 230 220 210 200 190 180 170 160 150 140 130 120 110 100 90 80 70 60 50 40 30 20 10
δ / ppm

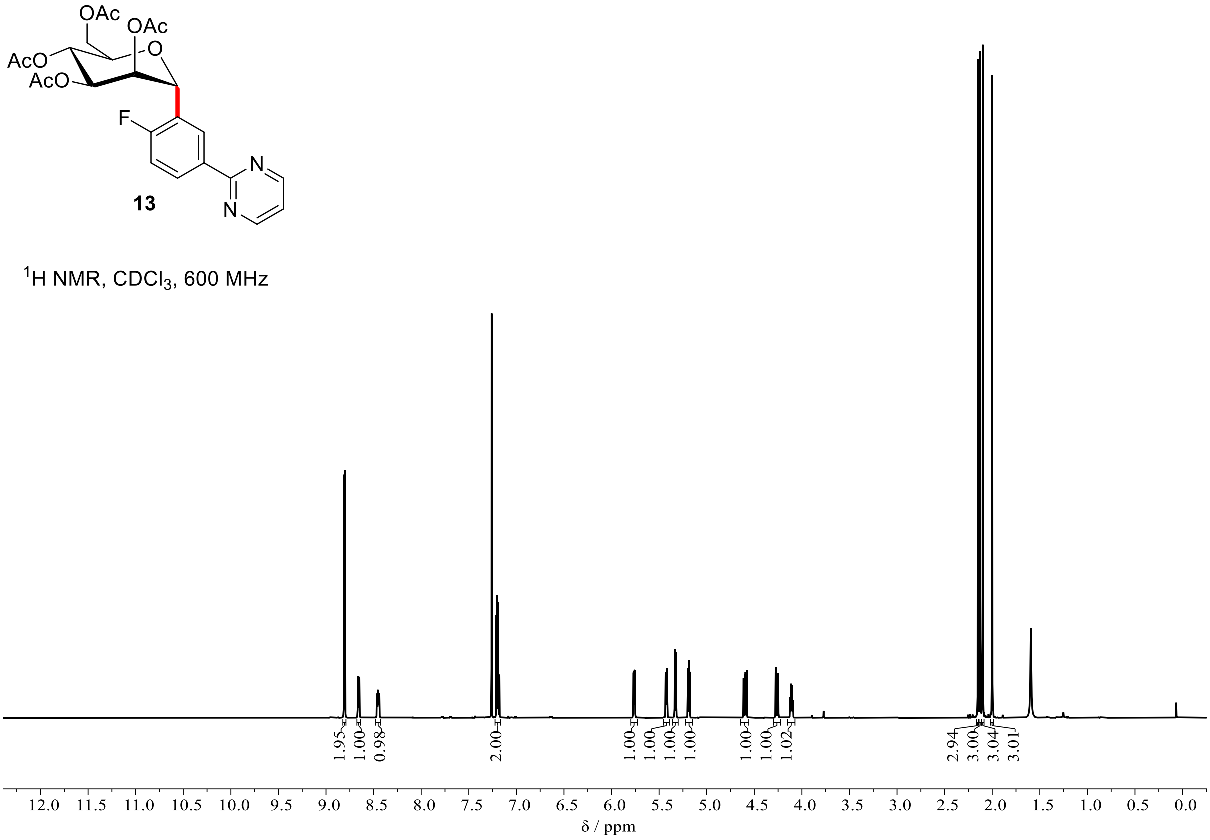
OAc
OAc
AcO
AcO
O
F
N
N
13
^{1}H NMR, $CDCl_3$, 600 MHz
1.95
1.00
0.98
2.00
1.00
1.00
1.00
1.00
1.00
1.00
1.02
2.94
3.00
3.04
3.01
12.0 11.5 11.0 10.5 10.0 9.5 9.0 8.5 8.0 7.5 7.0 6.5 6.0 5.5 5.0 4.5 4.0 3.5 3.0 2.5 2.0 1.5 1.0 0.5 0.0
δ / ppm

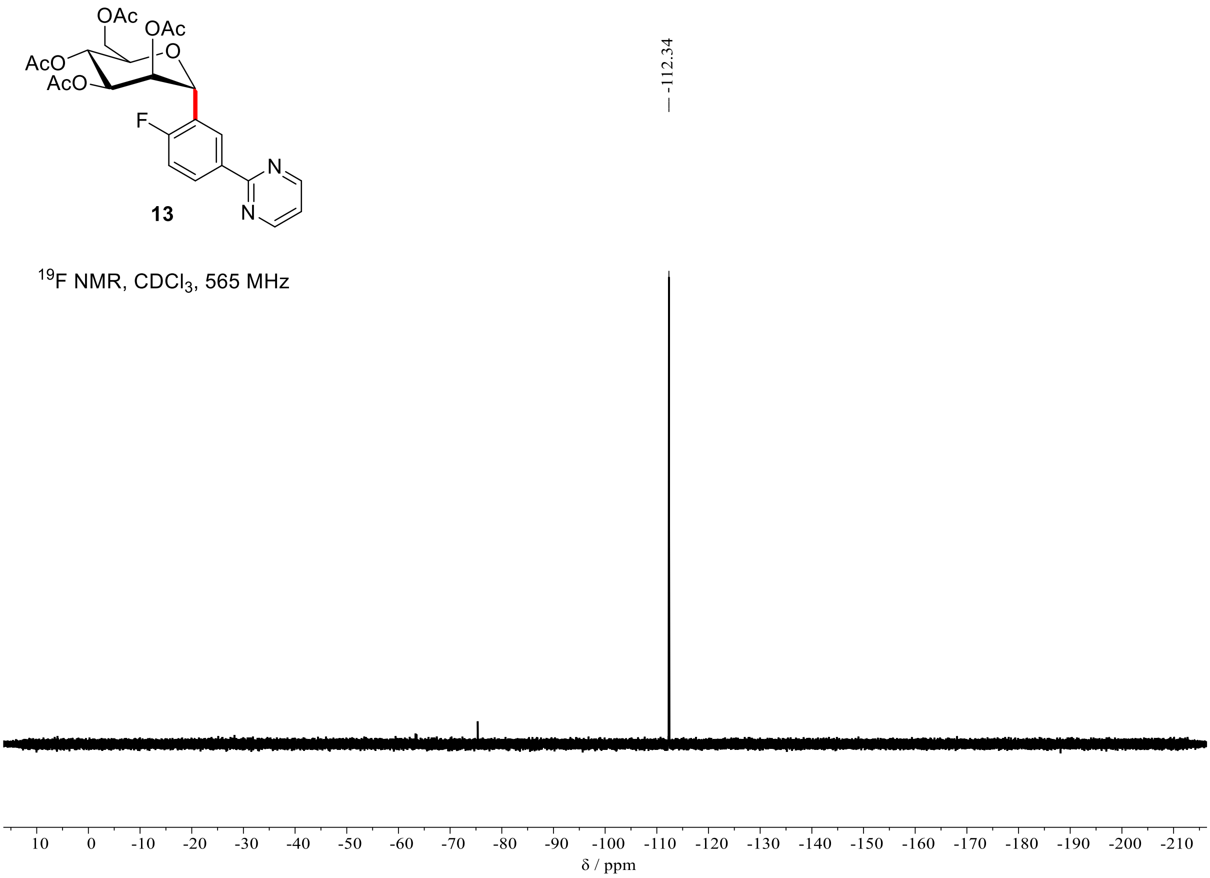
OAc
OAc
AcO
AcO
O
F
N
N
13
19F NMR, CDCl3, 565 MHz
-112.34
δ / ppm

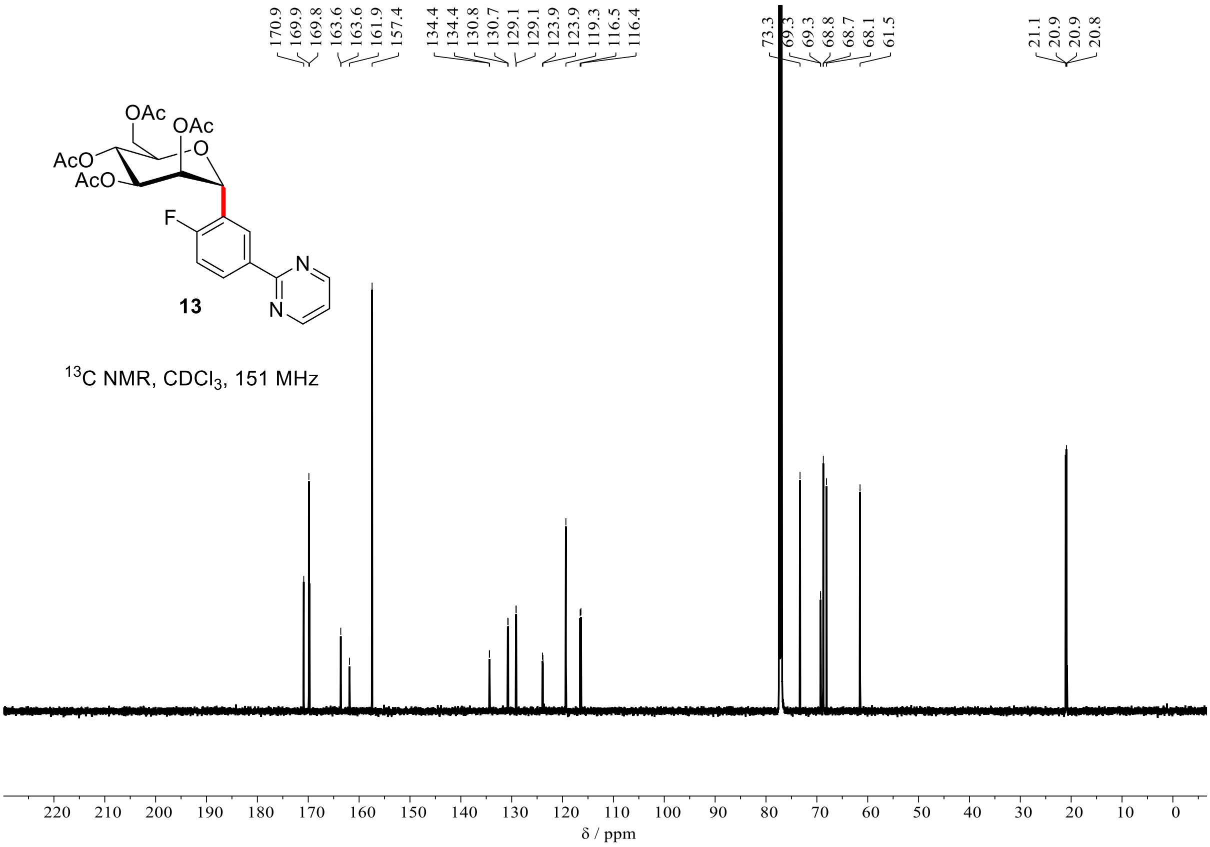
170.9
169.9
169.8
163.6
163.6
161.9
157.4
134.4
134.4
130.8
130.7
129.1
129.1
123.9
123.9
119.3
116.5
116.4
73.3
69.3
69.3
68.8
68.7
68.1
61.5
21.1
20.9
20.9
20.8
OAc
OAc
AcO
AcO
O
F
N
N
13
13C NMR, CDCl3, 151 MHz
δ / ppm

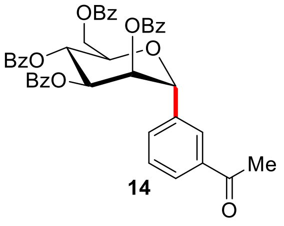

14

^{1}H NMR, $CDCl_3$, 400 MHz

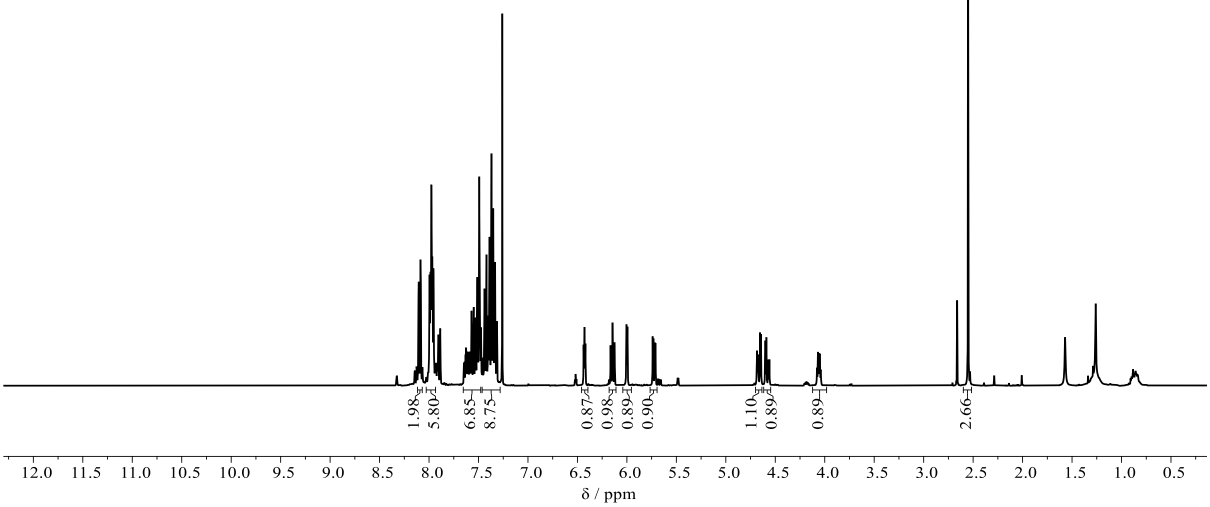

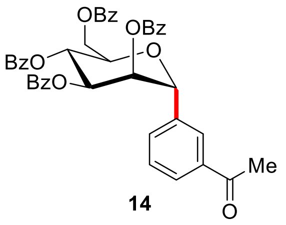

14

^{13}C NMR, $CDCl_3$, 101 MHz

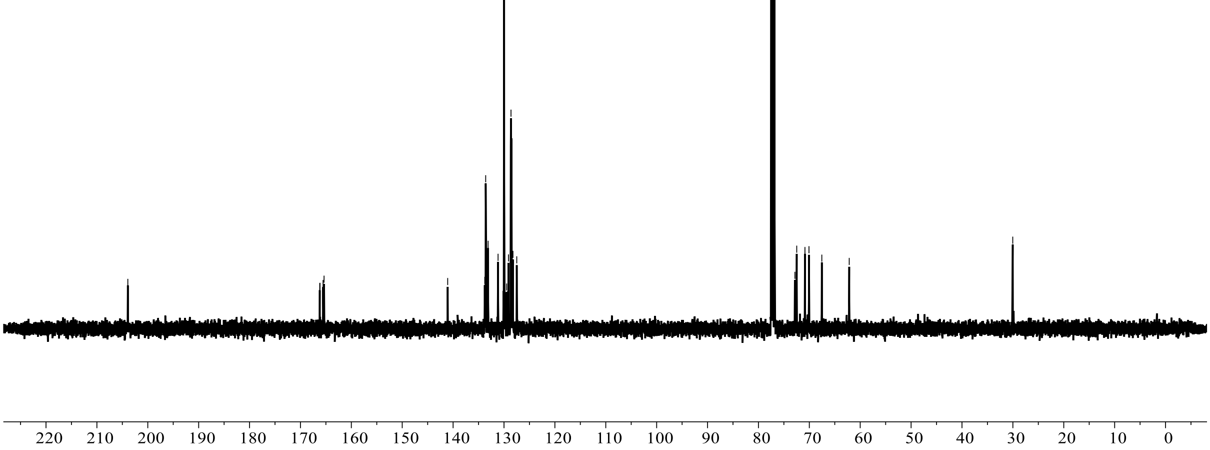

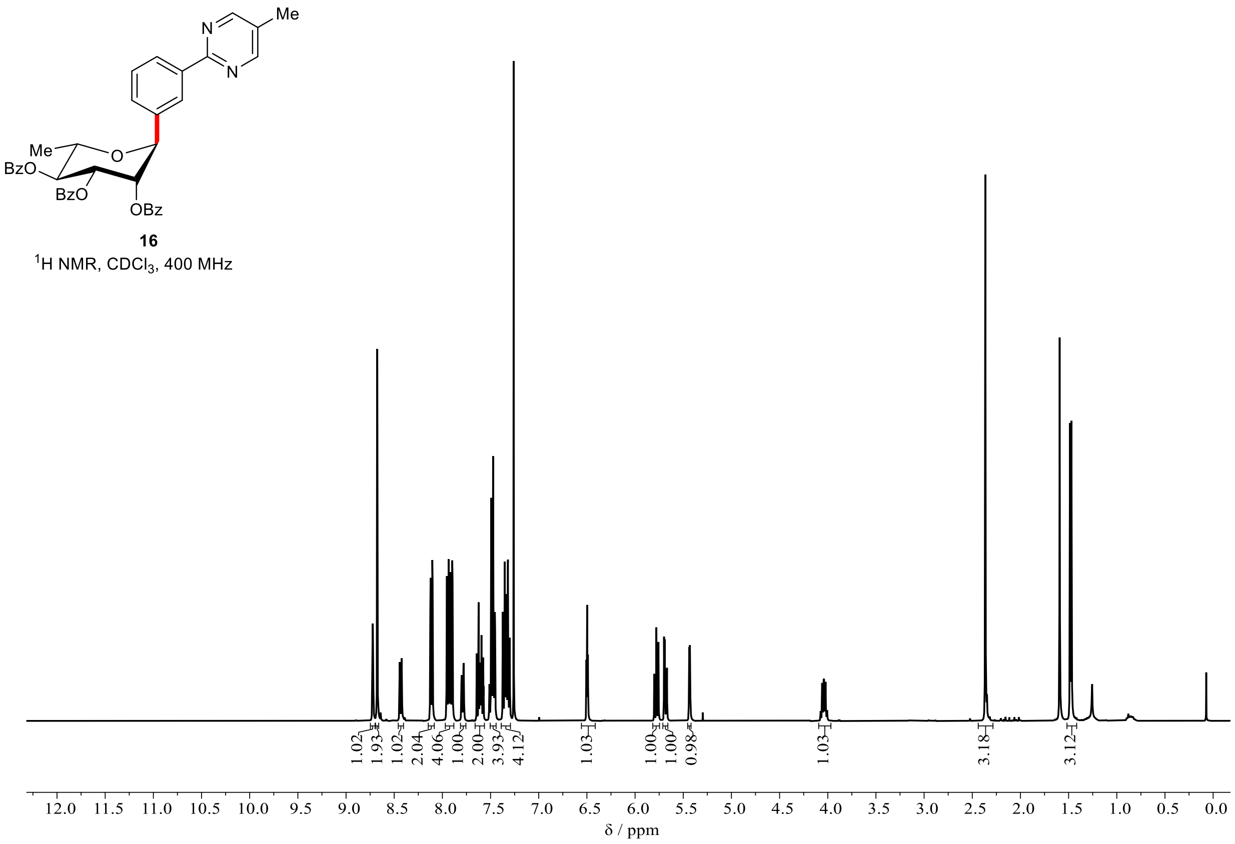

Me
N
N
Me
O
BzO
BzO
OBz
16
^{1}H NMR, CDCl$_3$, 400 MHz
1.02
1.93
1.02
2.04
4.06
1.00
1.00
2.00
3.93
4.12
1.03
1.00
1.00
0.98
1.03
3.18
3.12
12.0 11.5 11.0 10.5 10.0 9.5 9.0 8.5 8.0 7.5 7.0 6.5 6.0 5.5 5.0 4.5 4.0 3.5 3.0 2.5 2.0 1.5 1.0 0.5 0.0
δ / ppm

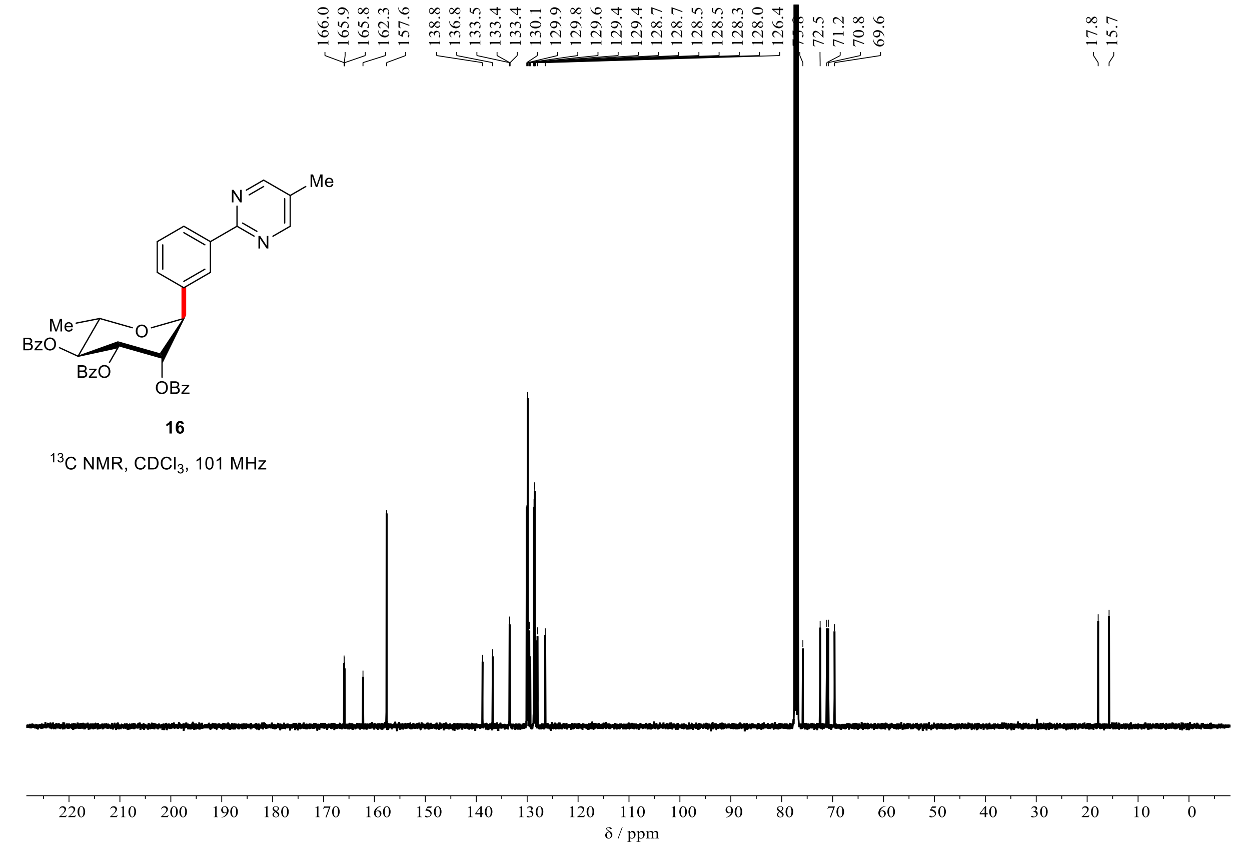

166.0
165.9
165.8
162.3
157.6
138.8
136.8
133.5
133.4
133.4
130.1
129.9
129.8
129.6
129.4
129.4
128.7
128.7
128.5
128.5
128.3
128.0
126.4
72.5
71.2
70.8
69.6
17.8
15.7
Me
N
N
Me
O
BzO
BzO
OBz
16
^{13}C NMR, CDCl$_3$, 101 MHz
220 210 200 190 180 170 160 150 140 130 120 110 100 90 80 70 60 50 40 30 20 10 0
δ / ppm

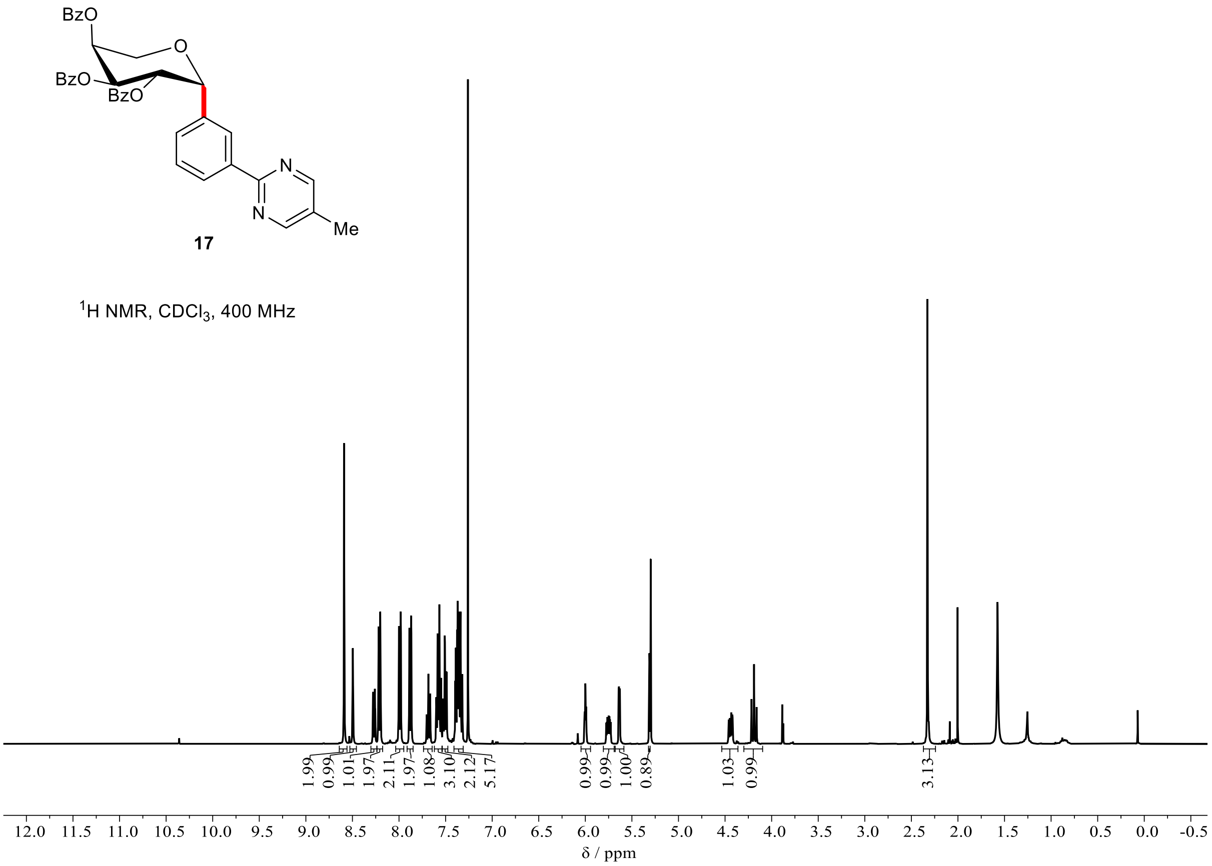
BzO
O
BzO
BzO
N
N
Me
17
1H NMR, CDCl3, 400 MHz
1.99 0.99 1.01 1.97 2.11 1.97 1.08 3.10 2.12 5.17
0.99 0.99 1.00 0.89
1.03 0.99
3.13
12.0 11.5 11.0 10.5 10.0 9.5 9.0 8.5 8.0 7.5 7.0 6.5 6.0 5.5 5.0 4.5 4.0 3.5 3.0 2.5 2.0 1.5 1.0 0.5 0.0 -0.5
δ / ppm

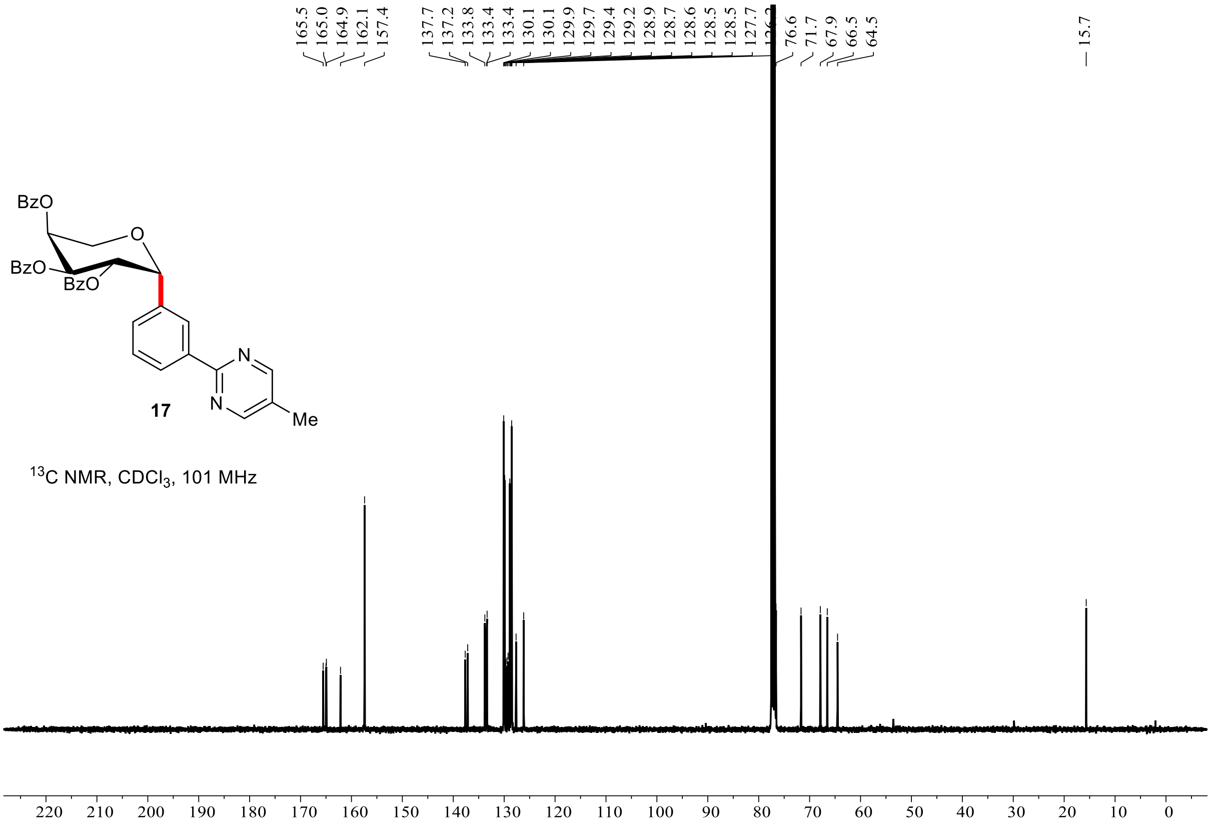
165.5 165.0 164.9 162.1 157.4
137.7 137.2 133.8 133.4 133.4 130.1 130.1 129.9 129.7 129.4 129.2 128.9 128.7 128.6 128.5 128.5 127.7
76.6 71.7 67.9 66.5 64.5
15.7
BzO
O
BzO
BzO
N
N
Me
17
13C NMR, CDCl3, 101 MHz
220 210 200 190 180 170 160 150 140 130 120 110 100 90 80 70 60 50 40 30 20 10 0
δ / ppm

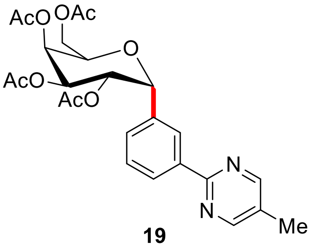

19

^{1}H NMR, $CDCl_3$, 400 MHz

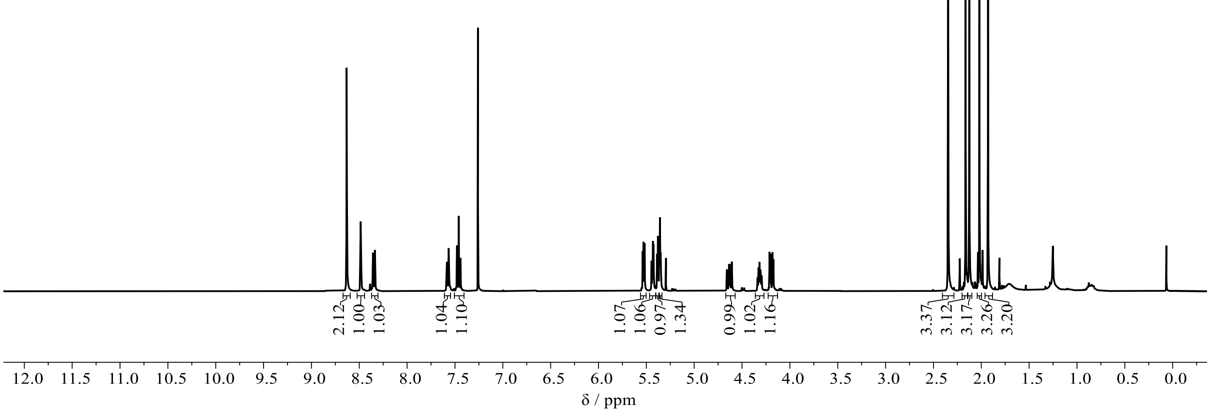

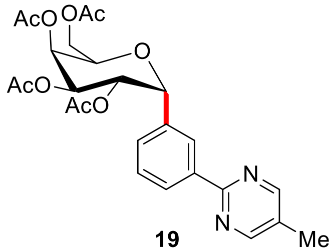

19

^{13}C NMR, $CDCl_3$, 101 MHz

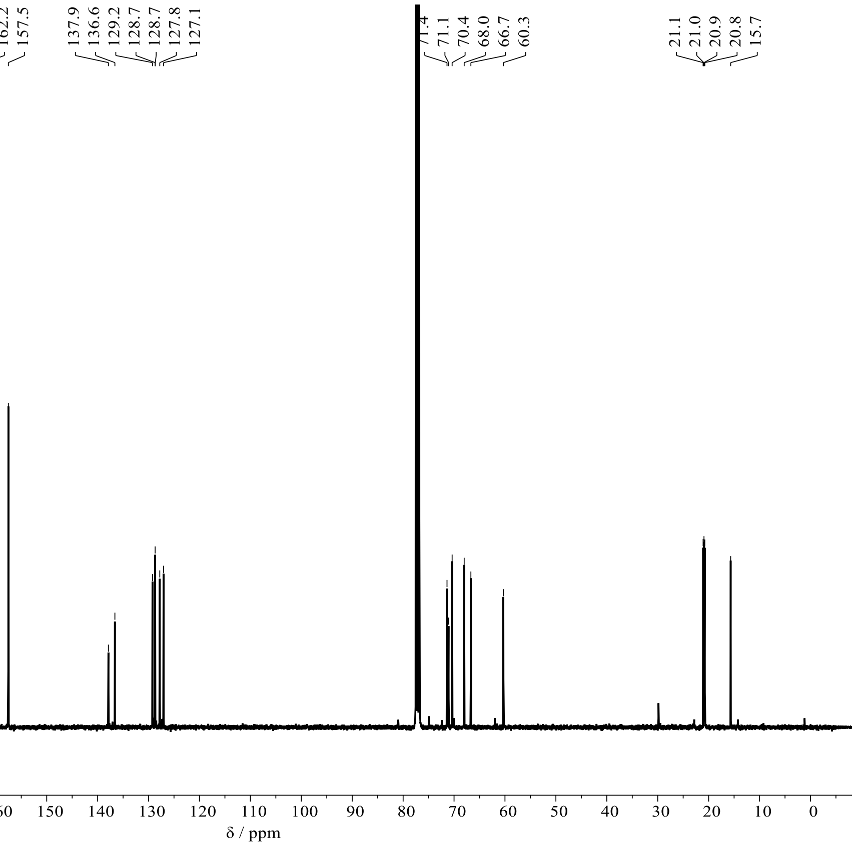

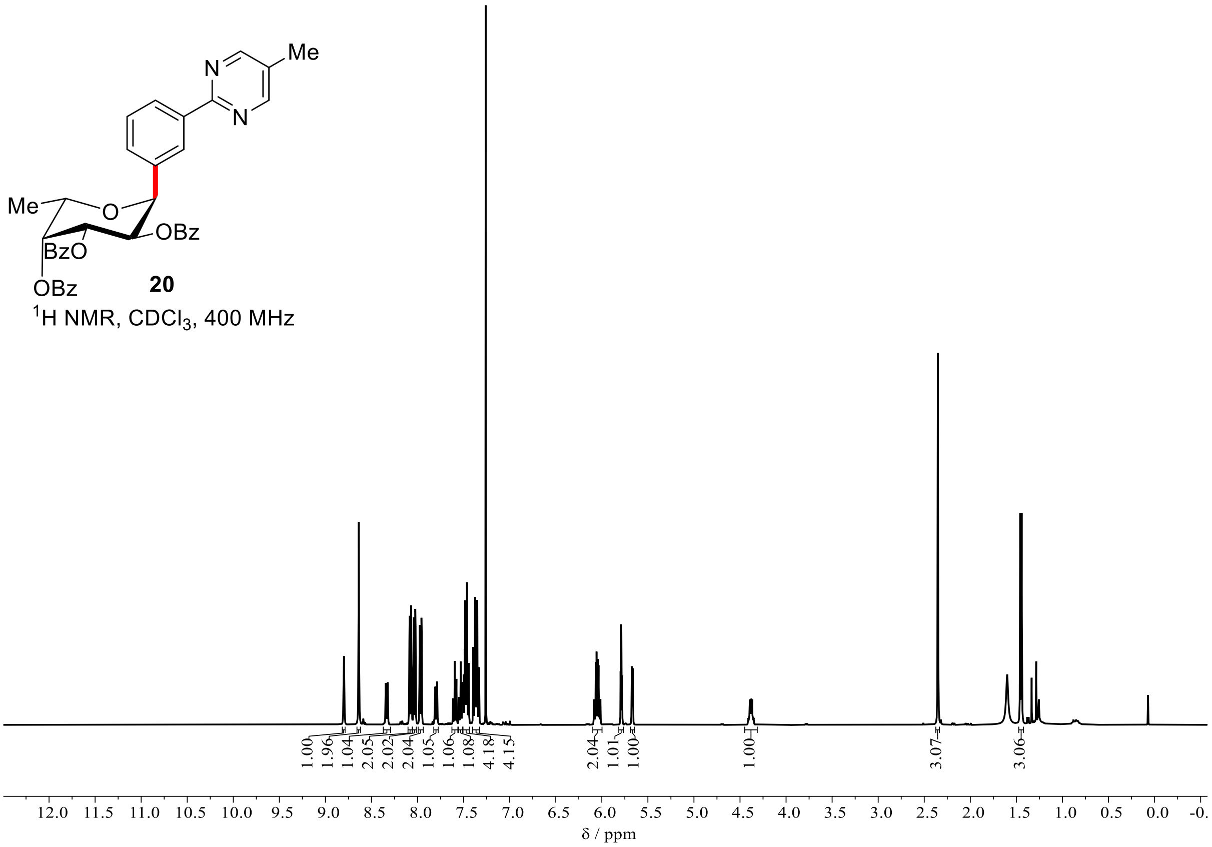
Me
N
N
Me
O
OBz
BzO
OBz
20
1H NMR, CDCl3, 400 MHz
1.00 1.96 1.04 2.05 2.02 2.04 1.05 1.06 1.08 4.18 4.15
2.04 1.01 1.00
1.00
3.07
3.06
12.0 11.5 11.0 10.5 10.0 9.5 9.0 8.5 8.0 7.5 7.0 6.5 6.0 5.5 5.0 4.5 4.0 3.5 3.0 2.5 2.0 1.5 1.0 0.5 0.0 -0.
δ / ppm

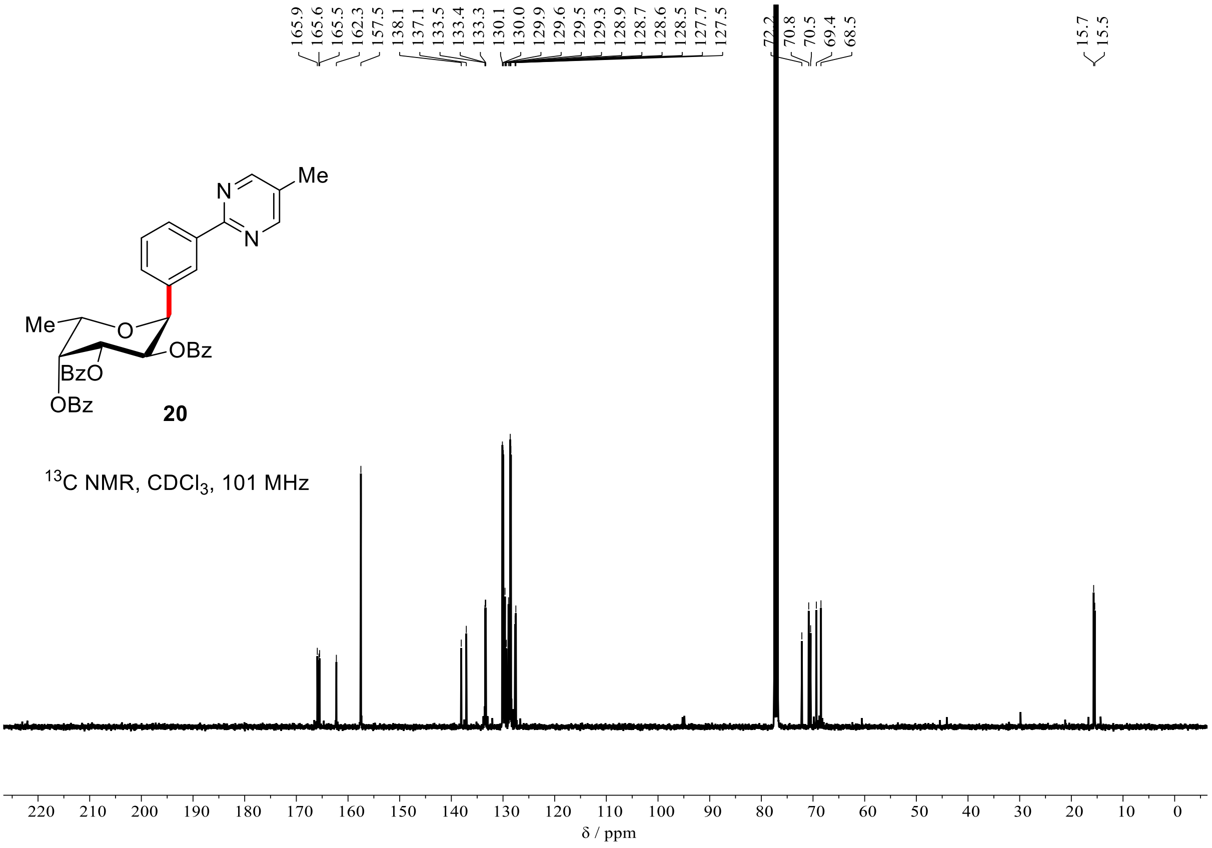
165.9 165.6 165.5 162.3 157.5 138.1 137.1 133.5 133.4 133.3 130.1 130.0 129.9 129.6 129.5 129.3 128.9 128.7 128.6 128.5 127.7 127.5
72.2 70.8 70.5 69.4 68.5
15.7 15.5
Me
N
N
Me
O
OBz
BzO
OBz
20
13C NMR, CDCl3, 101 MHz
220 210 200 190 180 170 160 150 140 130 120 110 100 90 80 70 60 50 40 30 20 10 0
δ / ppm

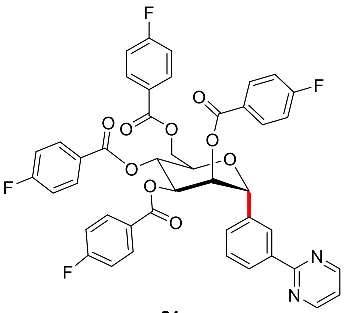

21
^{1}H NMR, CDCl$_3$, 400 MHz

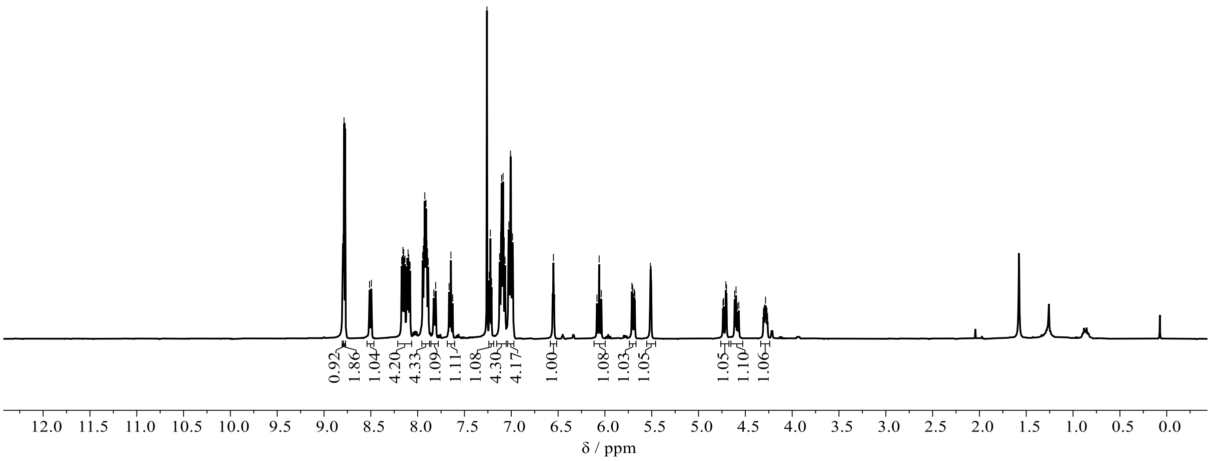

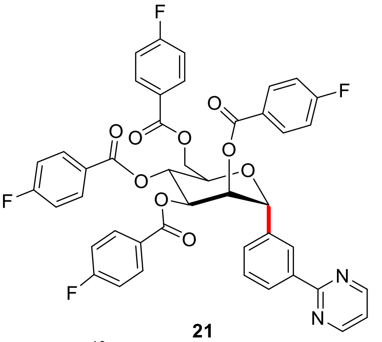

21
^{19}F NMR, CDCl$_3$, 282 MHz

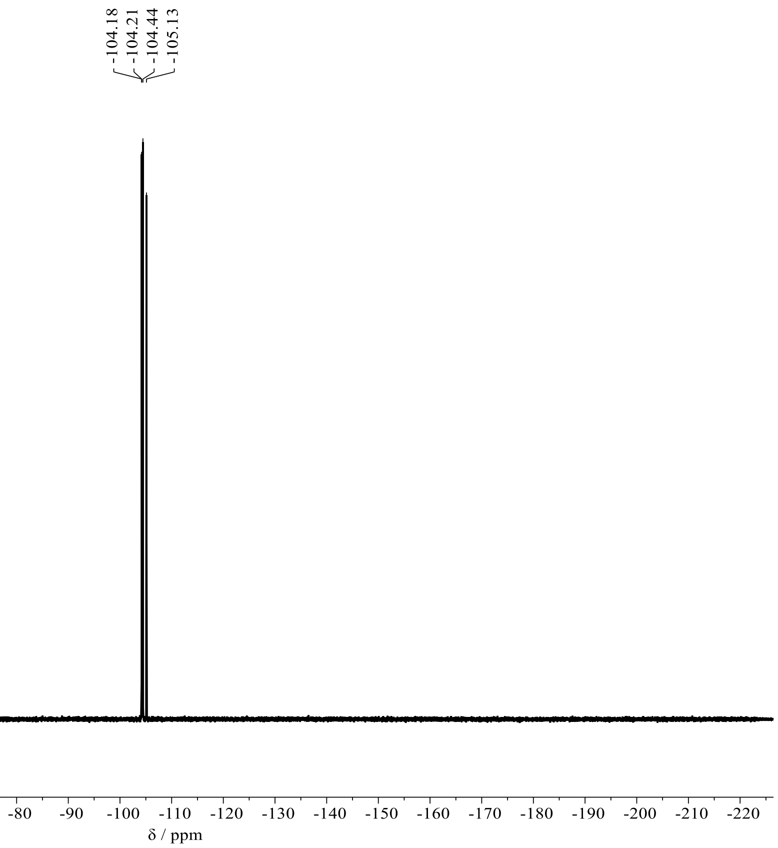

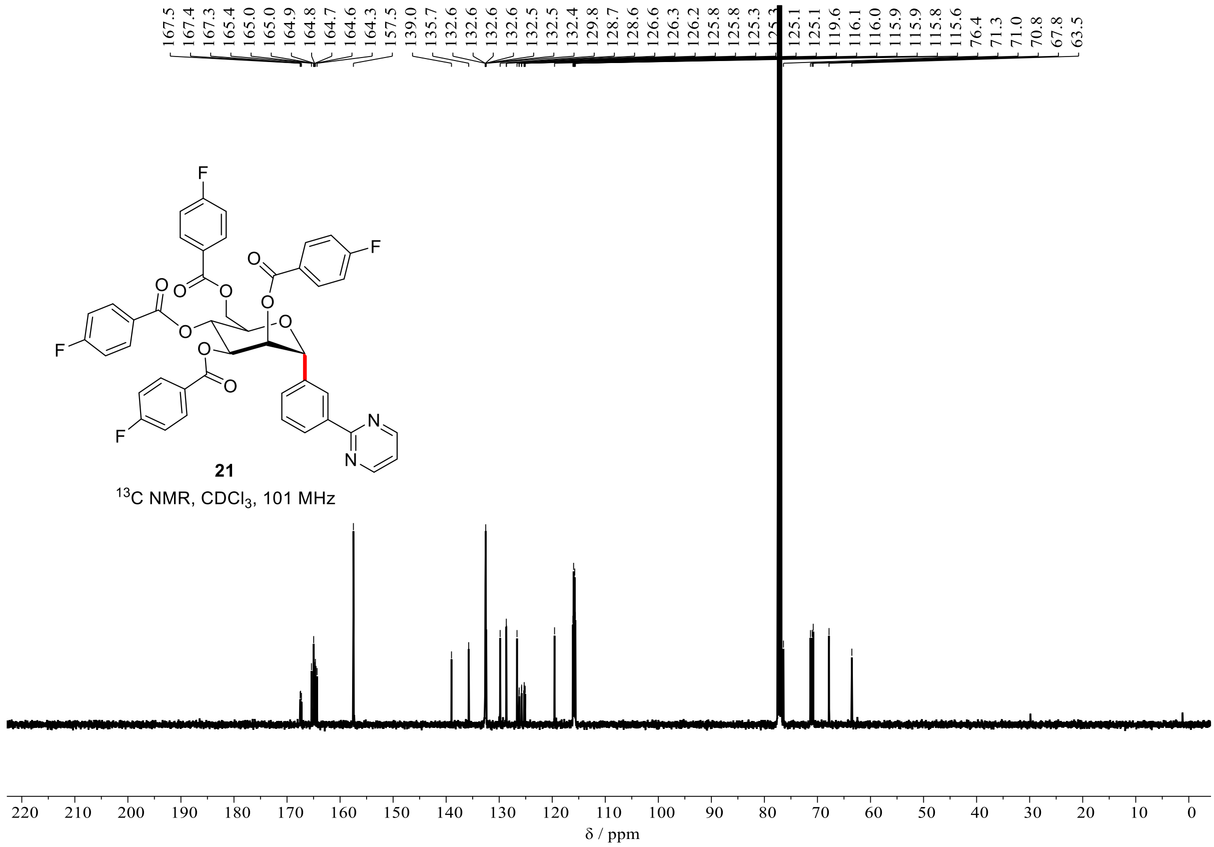
167.5 167.4 167.3 165.4 165.0 164.9 164.8 164.7 164.6 164.3 157.5 139.0 135.7 132.6 132.6 132.6 132.6 132.5 132.5 132.4 129.8 128.7 128.6 126.6 126.3 126.2 125.8 125.8 125.3 125.3 125.1 125.1 119.6 116.1 116.0 115.9 115.9 115.8 115.6 76.4 71.3 71.0 70.8 67.8 63.5
21
^{13}C NMR, $CDCl_3$, 101 MHz
δ / ppm

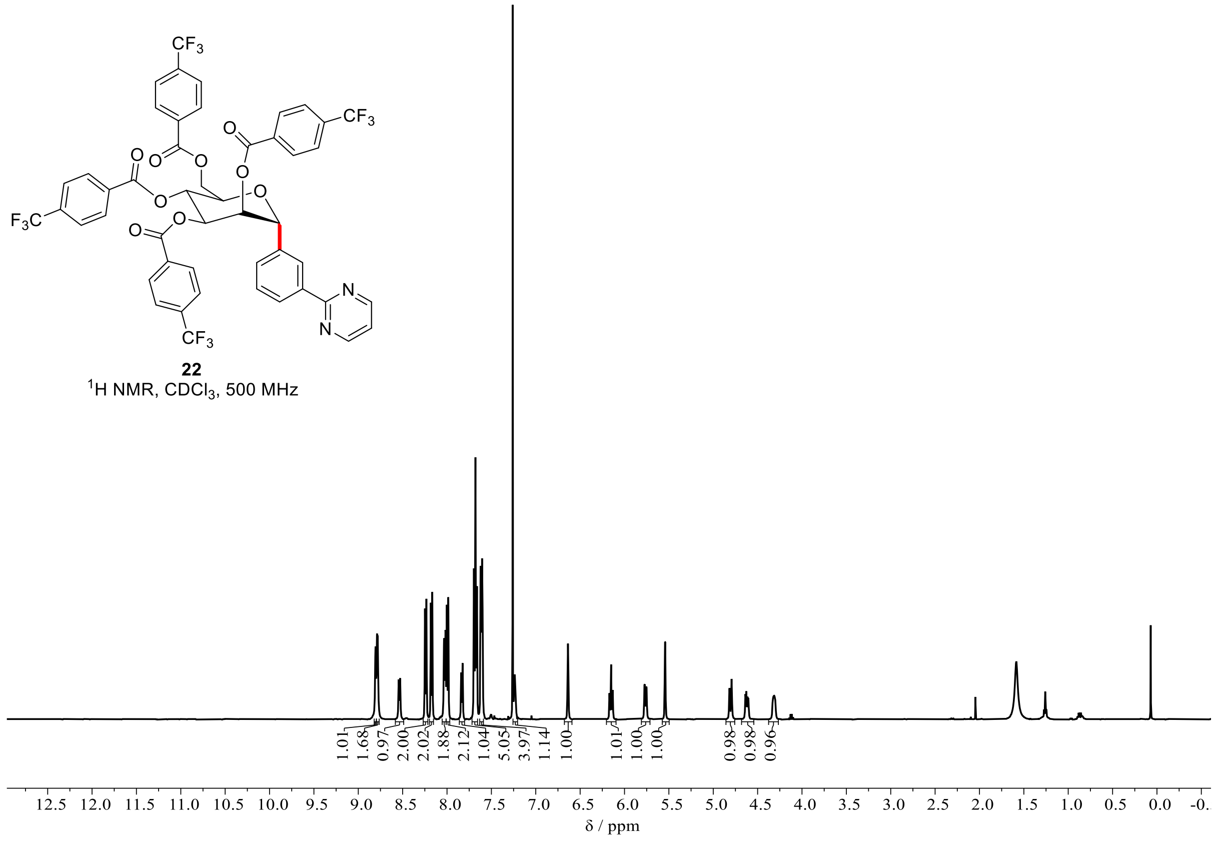
22
^{1}H NMR, $CDCl_3$, 500 MHz
1.01 1.68 0.97 2.00 2.02 1.88 2.12 1.04 5.05 3.97 1.14 1.00 1.01 1.00 1.00 0.98 0.98 0.96
δ / ppm

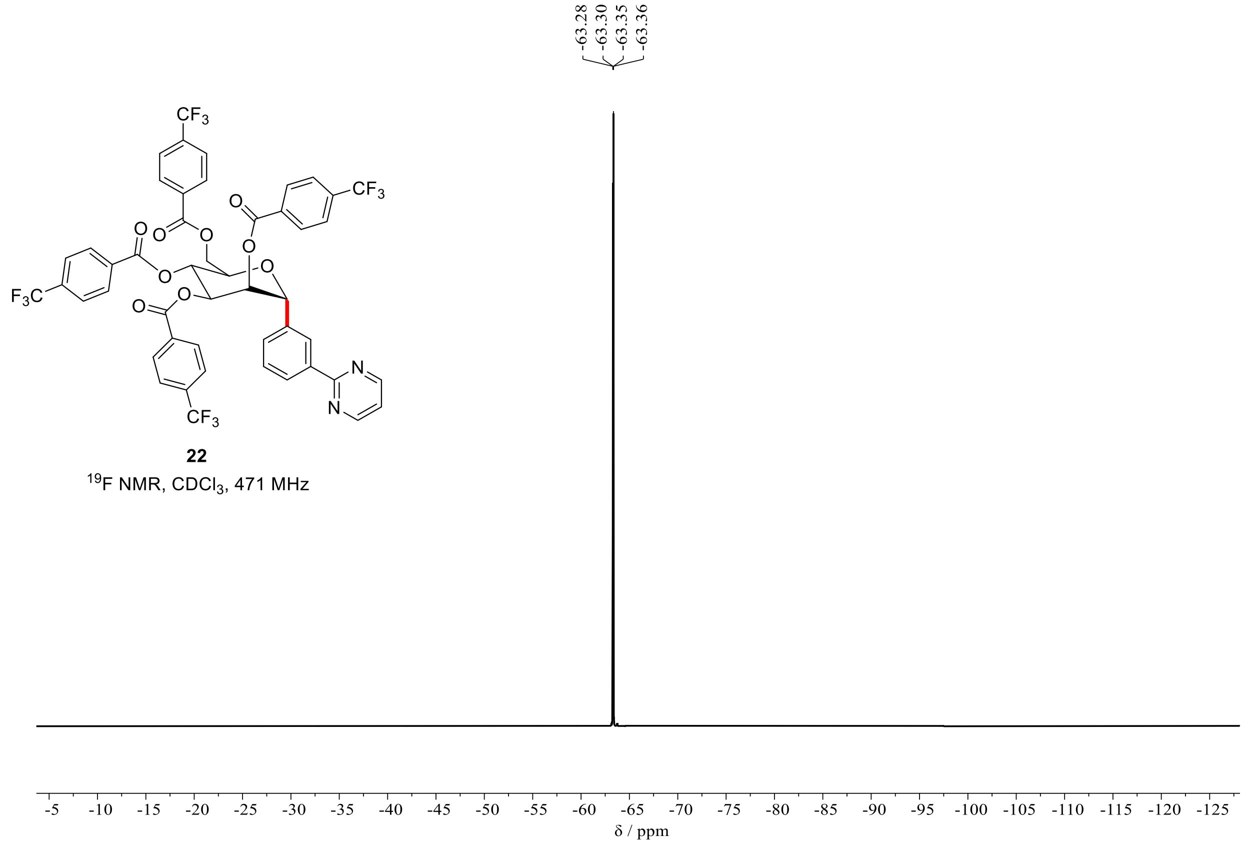
-63.28
-63.30
-63.35
-63.36
CF3
O
O
O
F3C
O
O
O
O
O
O
N
N
22
19F NMR, CDCl3, 471 MHz
δ / ppm

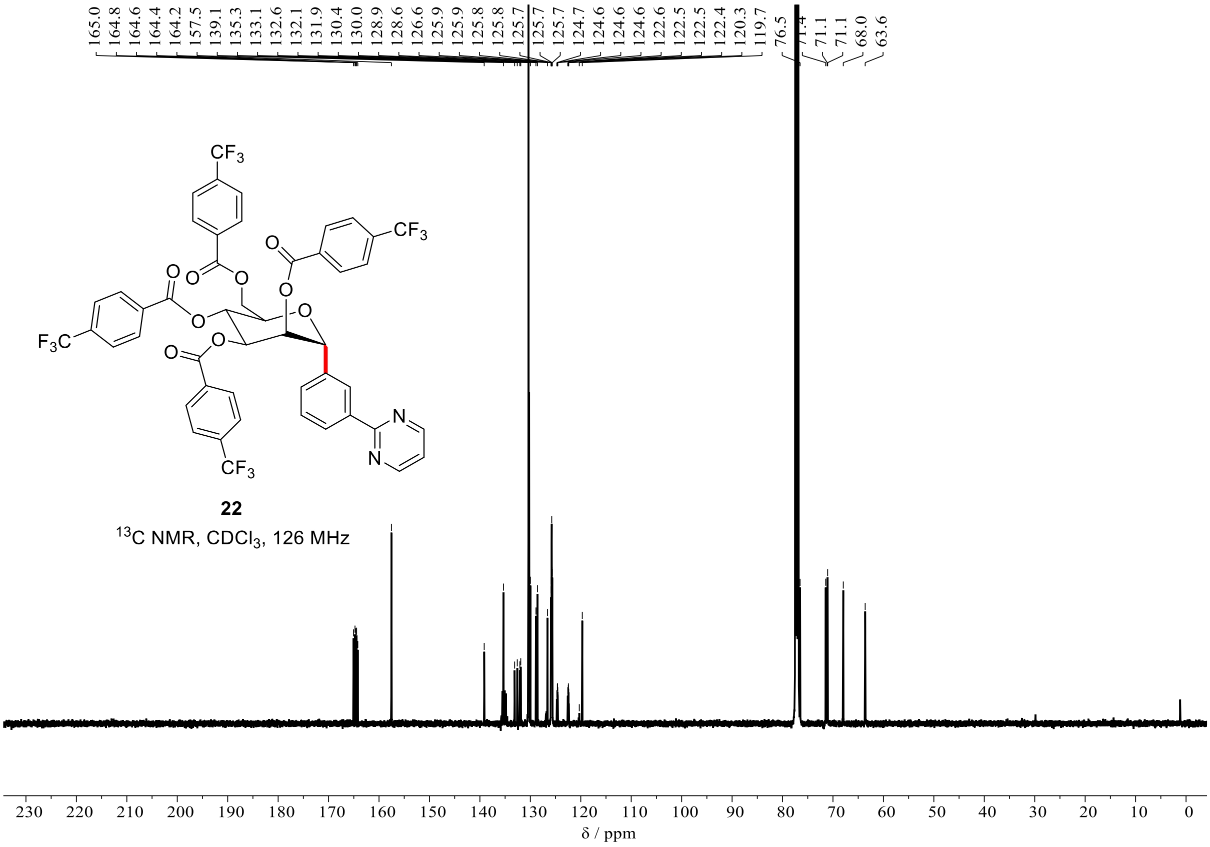
165.0
164.8
164.6
164.4
164.2
157.5
139.1
135.3
133.1
132.6
132.1
131.9
130.4
130.0
128.9
128.6
126.6
125.9
125.9
125.8
125.8
125.7
125.7
125.7
124.7
124.6
124.6
124.6
122.6
122.5
122.5
122.4
120.3
119.7
76.5
71.4
71.1
71.1
68.0
63.6
22
13C NMR, CDCl3, 126 MHz
δ / ppm

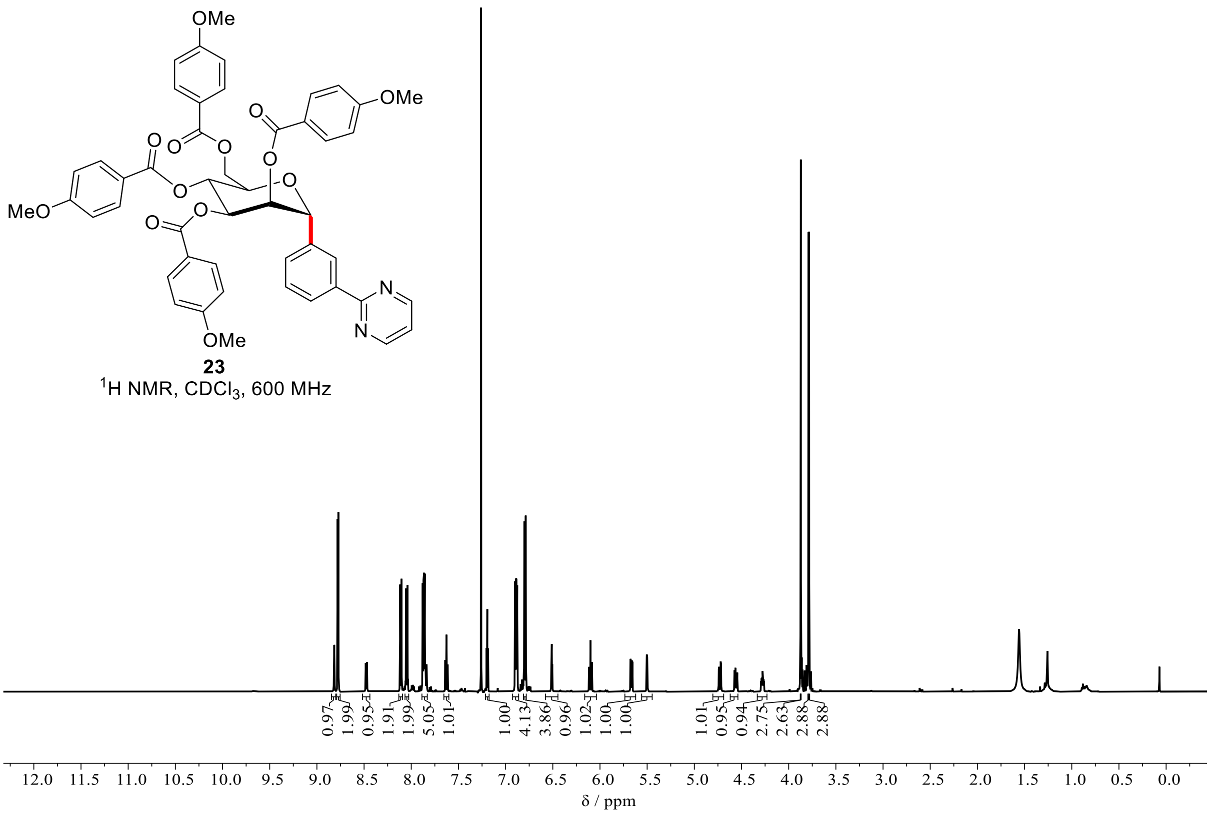
OMe
OMe
MeO
O
O
N
N
OMe
23
^{1}H NMR, CDCl$_3$, 600 MHz
0.97
1.99
0.95
1.91
1.99
5.05
1.01
1.00
4.13
3.86
0.96
1.02
1.00
1.00
1.01
0.95
0.94
2.75
2.63
2.88
2.88
12.0 11.5 11.0 10.5 10.0 9.5 9.0 8.5 8.0 7.5 7.0 6.5 6.0 5.5 5.0 4.5 4.0 3.5 3.0 2.5 2.0 1.5 1.0 0.5 0.0
δ / ppm

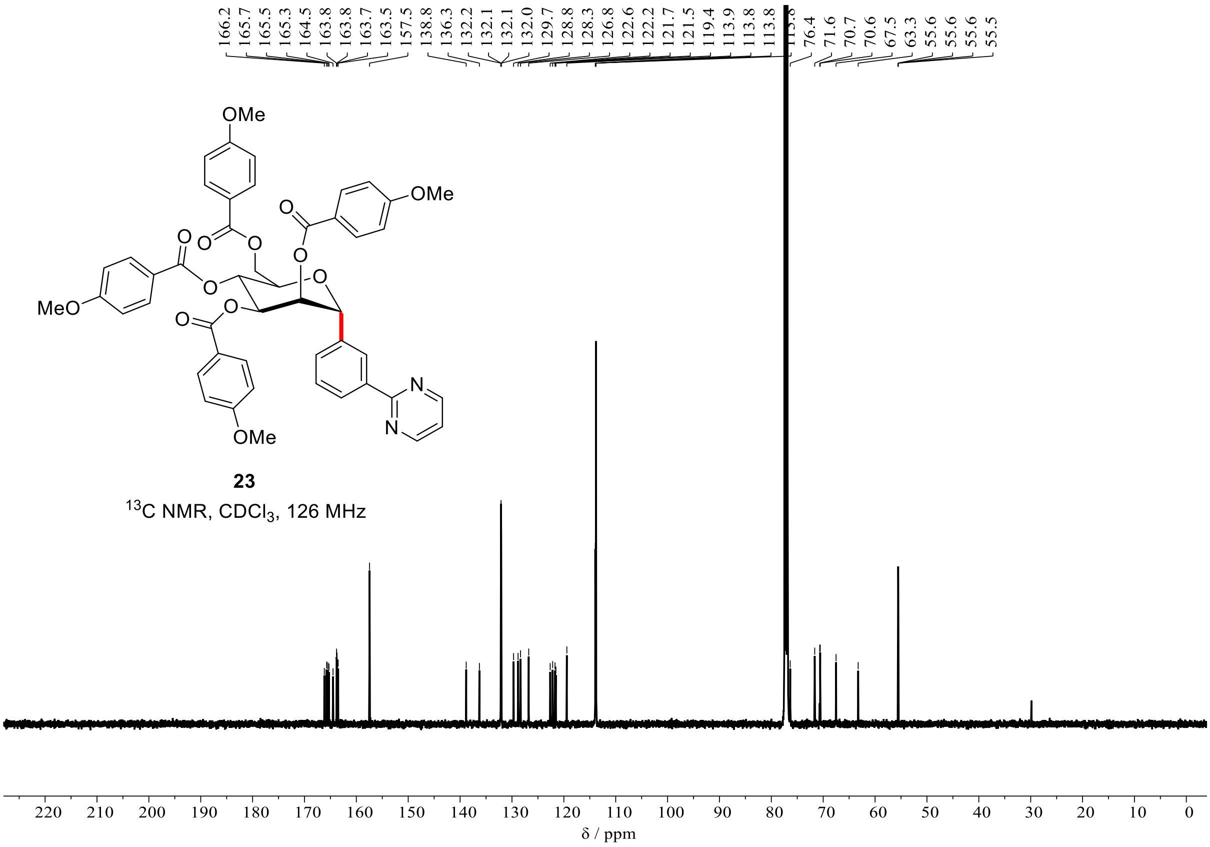
166.2
165.7
165.5
165.3
164.5
163.8
163.8
163.7
163.5
157.5
138.8
136.3
132.2
132.1
132.1
132.0
129.7
128.8
128.3
126.8
122.6
122.2
121.7
121.5
119.4
113.9
113.8
113.8
76.4
71.6
70.7
70.6
67.5
63.3
55.6
55.6
55.6
55.5
OMe
OMe
MeO
O
O
N
N
OMe
23
^{13}C NMR, CDCl$_3$, 126 MHz
220 210 200 190 180 170 160 150 140 130 120 110 100 90 80 70 60 50 40 30 20 10 0
δ / ppm

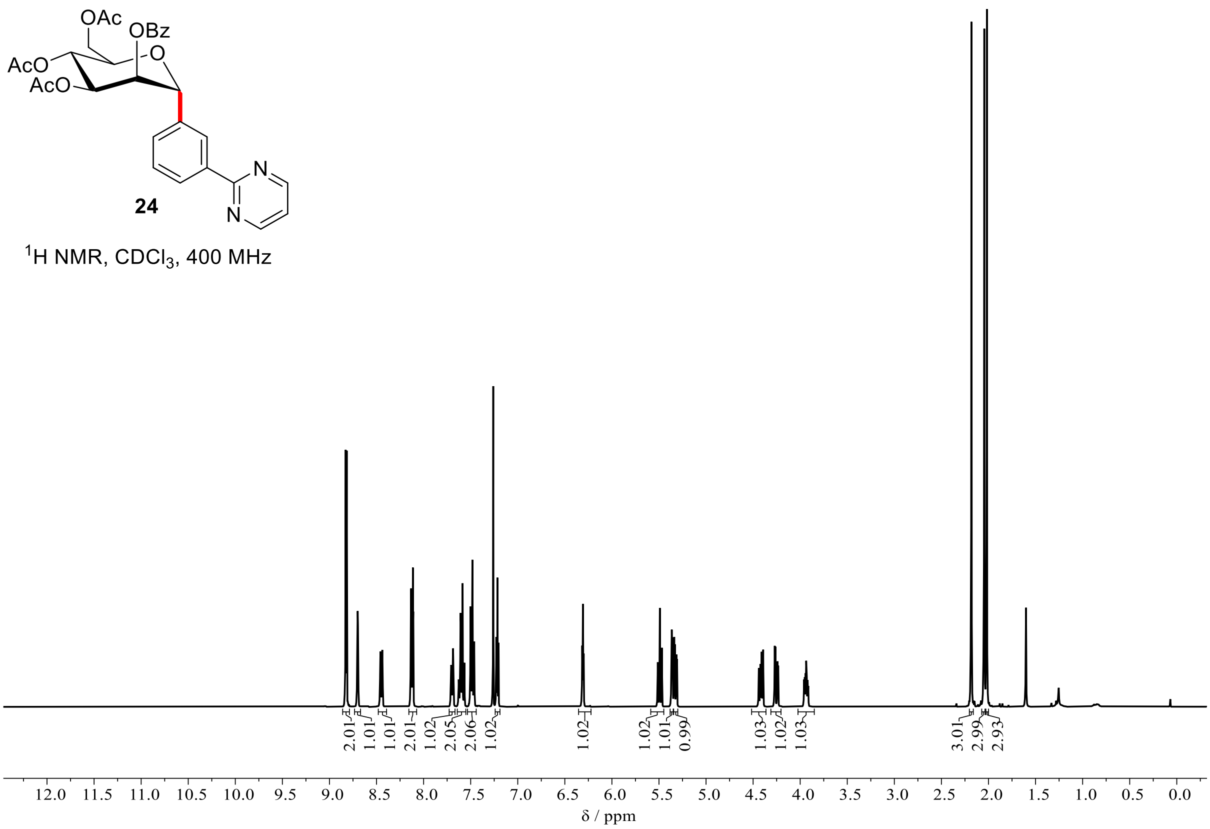
OAc
OBz
AcO
AcO
O
N
N
24
1H NMR, CDCl3, 400 MHz
2.01
1.01
1.01
2.01
1.02
2.05
2.06
1.02
1.02
1.02
1.01
0.99
1.03
1.02
1.03
3.01
2.99
2.93
δ / ppm

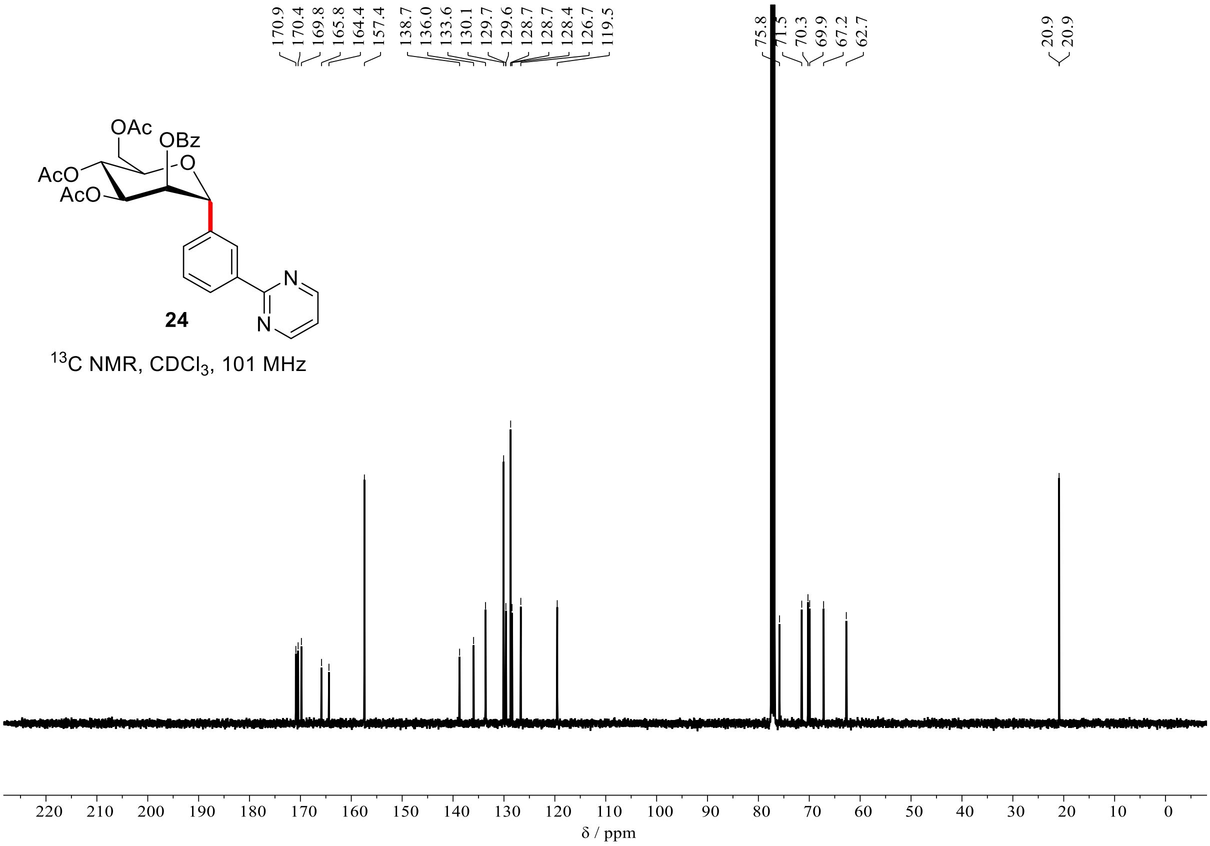
170.9
170.4
169.8
165.8
164.4
157.4
138.7
136.0
133.6
130.1
129.7
129.6
128.7
128.7
128.4
126.7
119.5
75.8
71.5
70.3
69.9
67.2
62.7
20.9
20.9
OAc
OBz
AcO
AcO
O
N
N
24
13C NMR, CDCl3, 101 MHz
δ / ppm

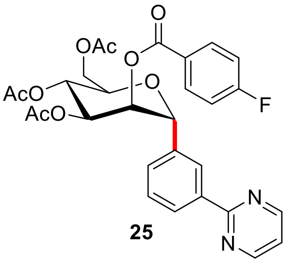

25

^{1}H NMR, $CDCl_3$, 400 MHz

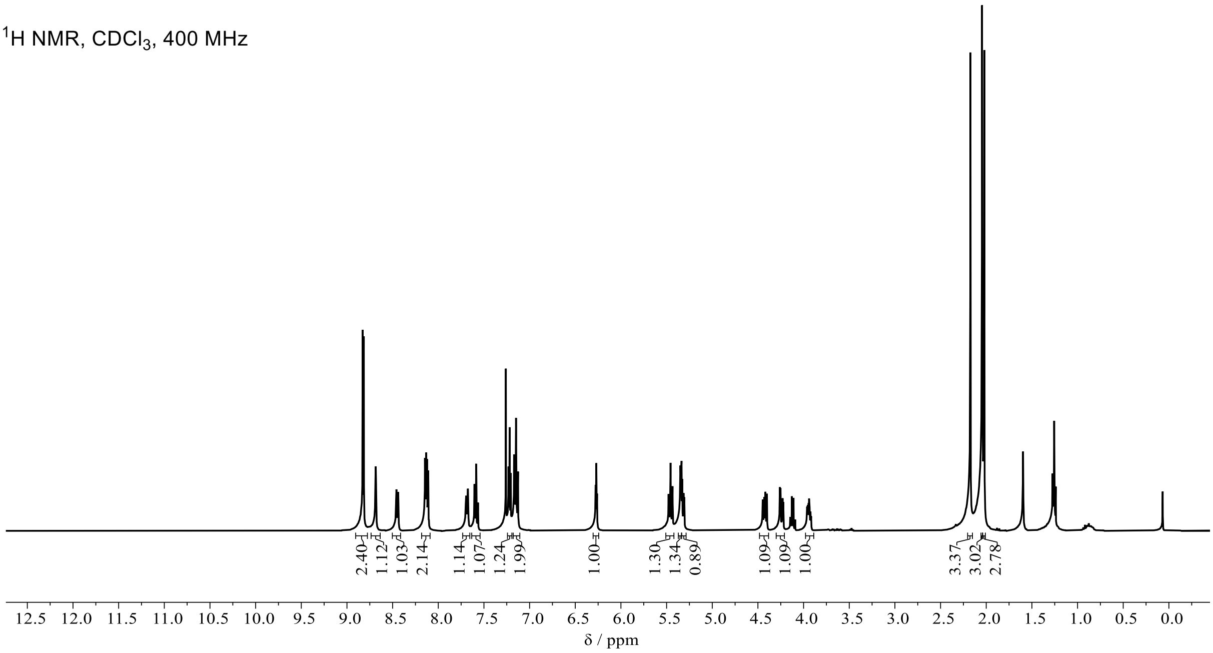

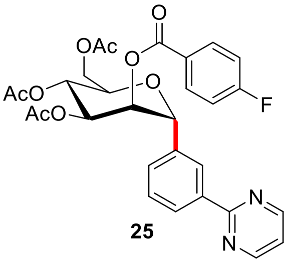

25

^{19}F NMR, $CDCl_3$, 377 MHz

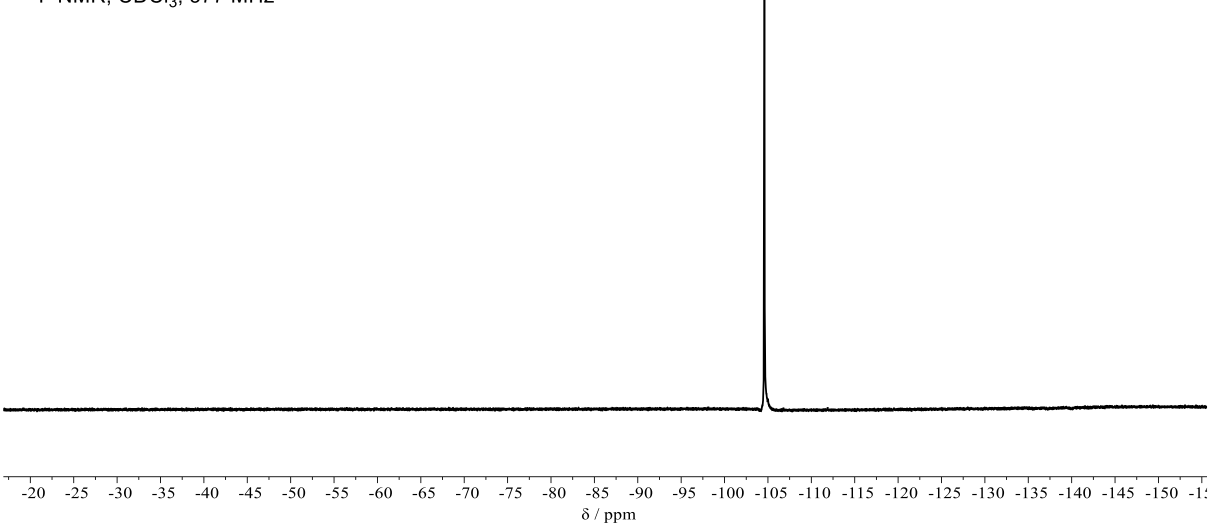

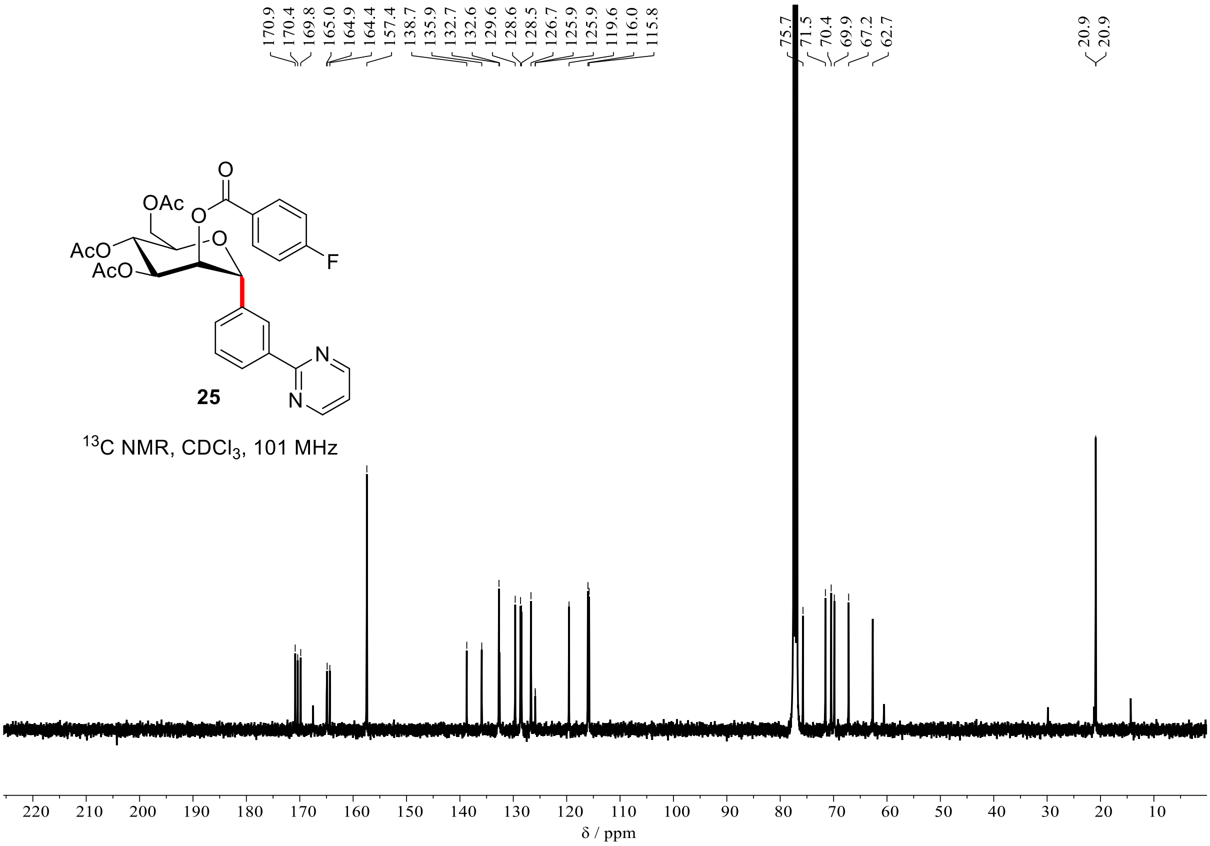
170.9
170.4
169.8
165.0
164.9
164.4
157.4
138.7
135.9
132.7
132.6
129.6
128.6
128.5
126.7
125.9
125.9
119.6
116.0
115.8
75.7
71.5
70.4
69.9
67.2
62.7
20.9
20.9
OAc
O
AcO
AcO
F
N
N
25
^{13}C NMR, CDCl$_3$, 101 MHz
δ / ppm

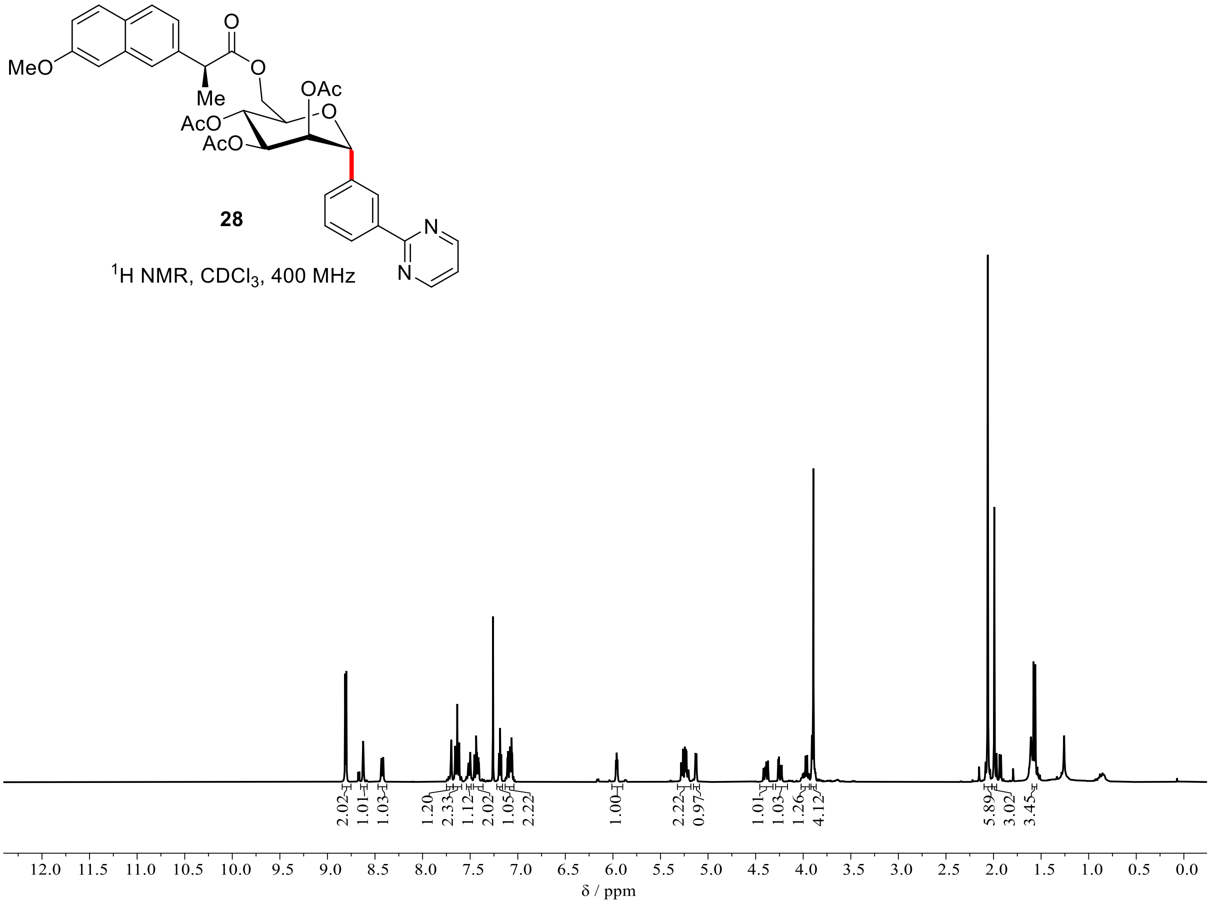
MeO
O
Me
OAc
AcO
AcO
N
N
28
^{1}H NMR, CDCl$_3$, 400 MHz
2.02
1.01
1.03
1.20
2.33
1.12
2.02
1.05
2.22
1.00
2.22
0.97
1.01
1.03
1.26
4.12
5.89
3.02
3.45
δ / ppm

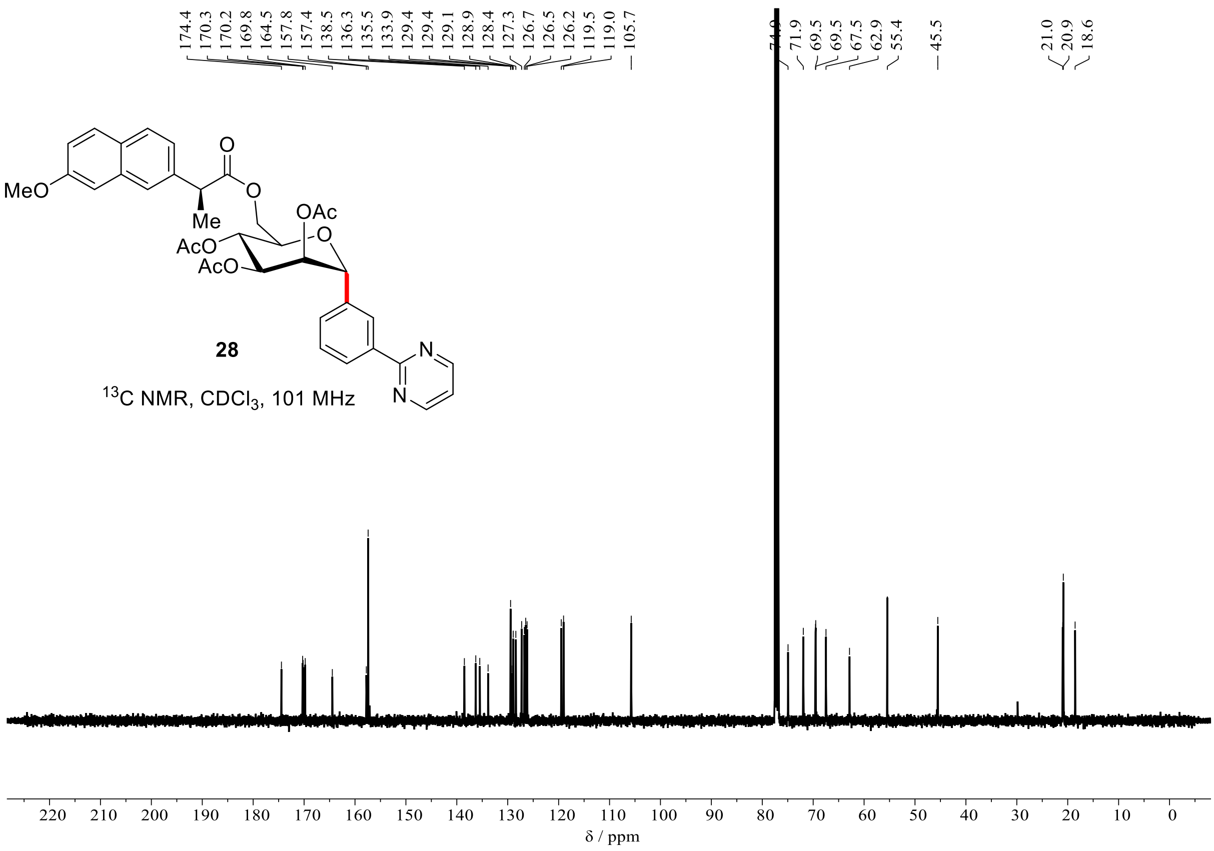

MeO
Me
OAc
AcO
AcO
O
N
N
28
^{13}C NMR, CDCl$_3$, 101 MHz
174.4
170.3
170.2
169.8
164.5
157.8
157.4
138.5
136.3
135.5
133.9
129.4
129.4
129.1
128.9
128.4
127.3
126.7
126.5
126.2
119.5
119.0
105.7
71.9
69.5
69.5
67.5
62.9
55.4
45.5
21.0
20.9
18.6
δ / ppm

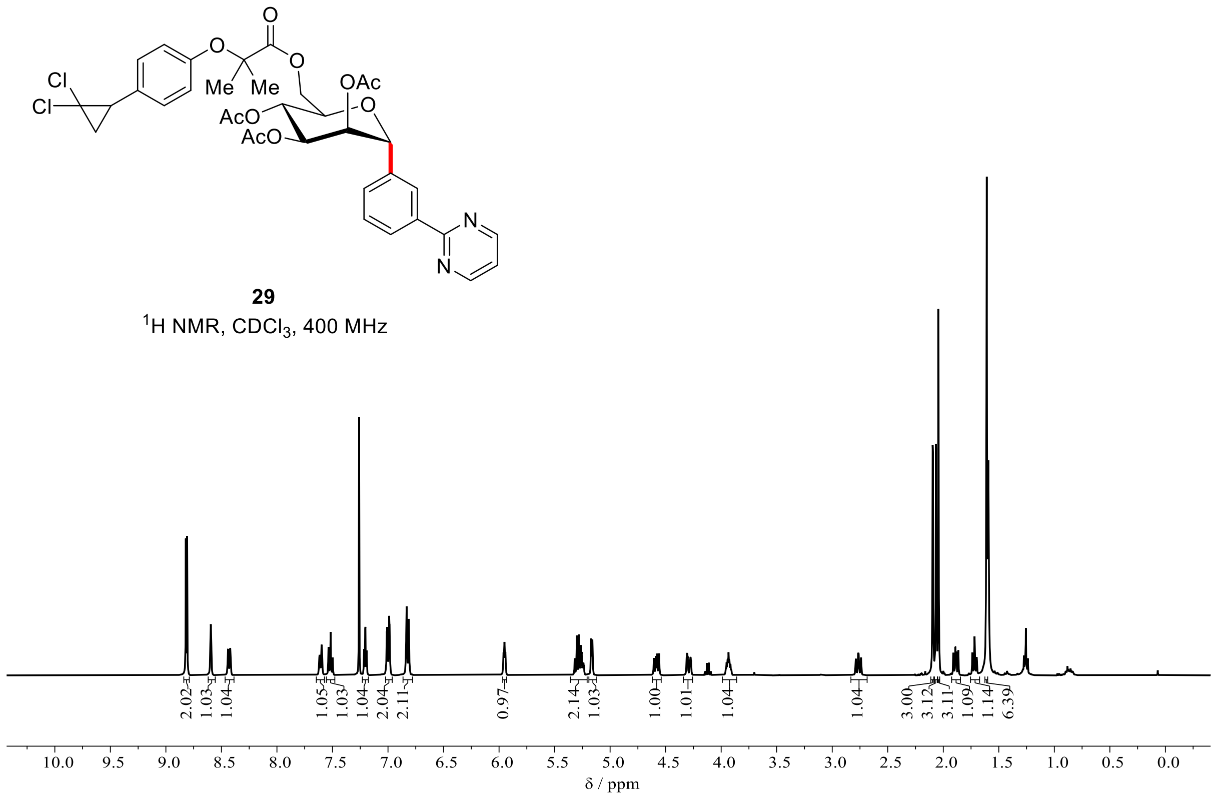

Cl
Cl
O
Me
Me
OAc
AcO
AcO
O
N
N
29
^{1}H NMR, CDCl$_3$, 400 MHz
2.02
1.03
1.04
1.05
1.03
1.04
2.04
2.11
0.97
2.14
1.03
1.00
1.01
1.04
1.04
3.00
3.12
3.11
1.09
1.14
6.39
δ / ppm

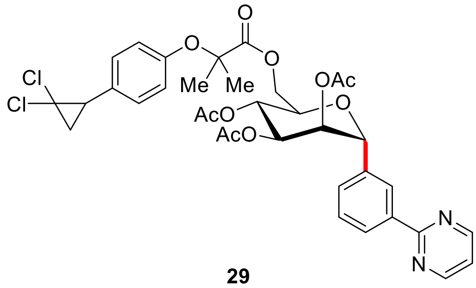

29

^{13}C NMR, $CDCl_3$, 101 MHz

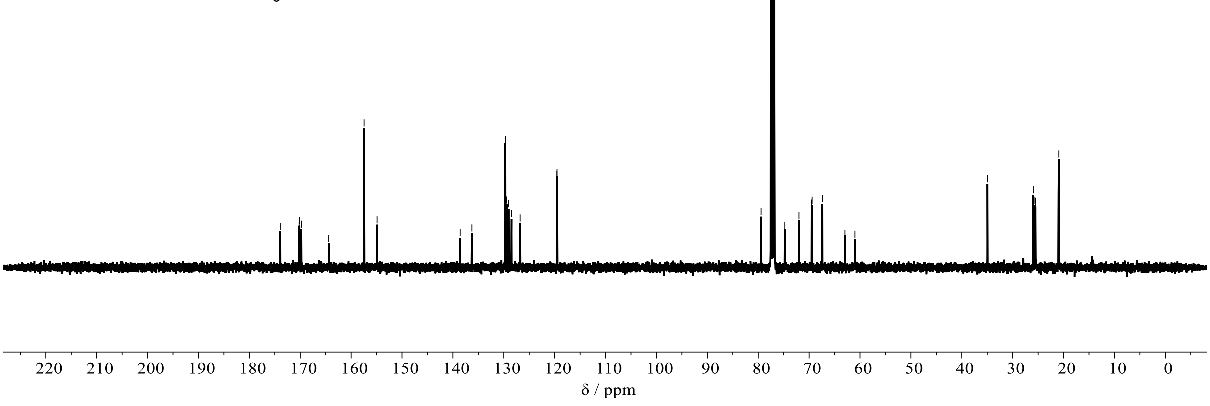

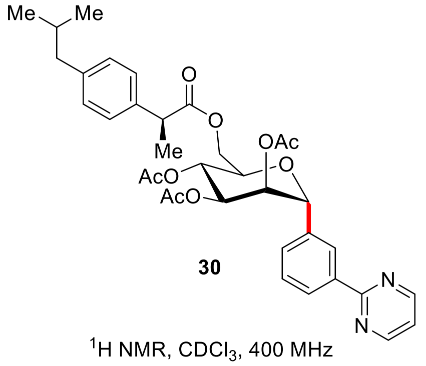

30

^{1}H NMR, $CDCl_3$, 400 MHz

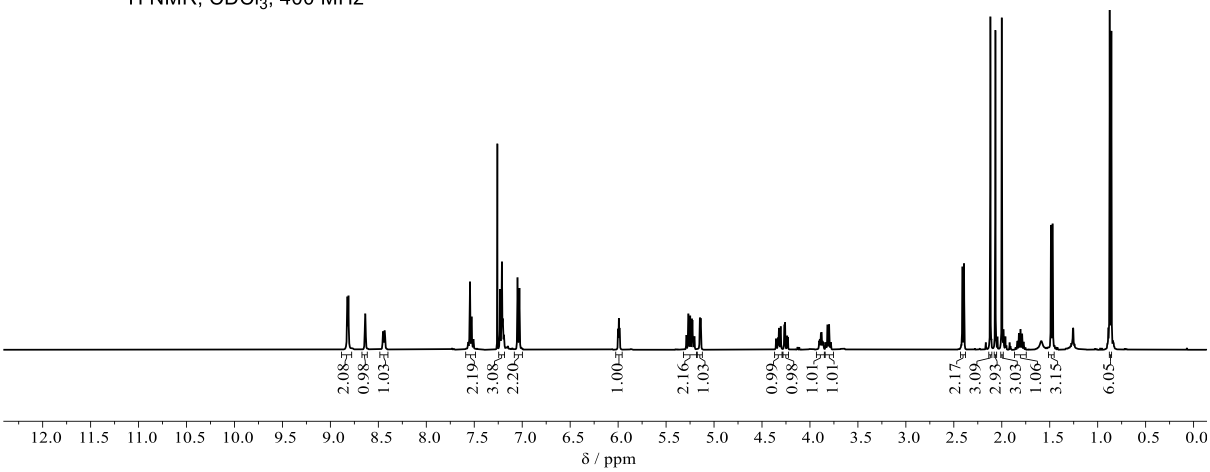

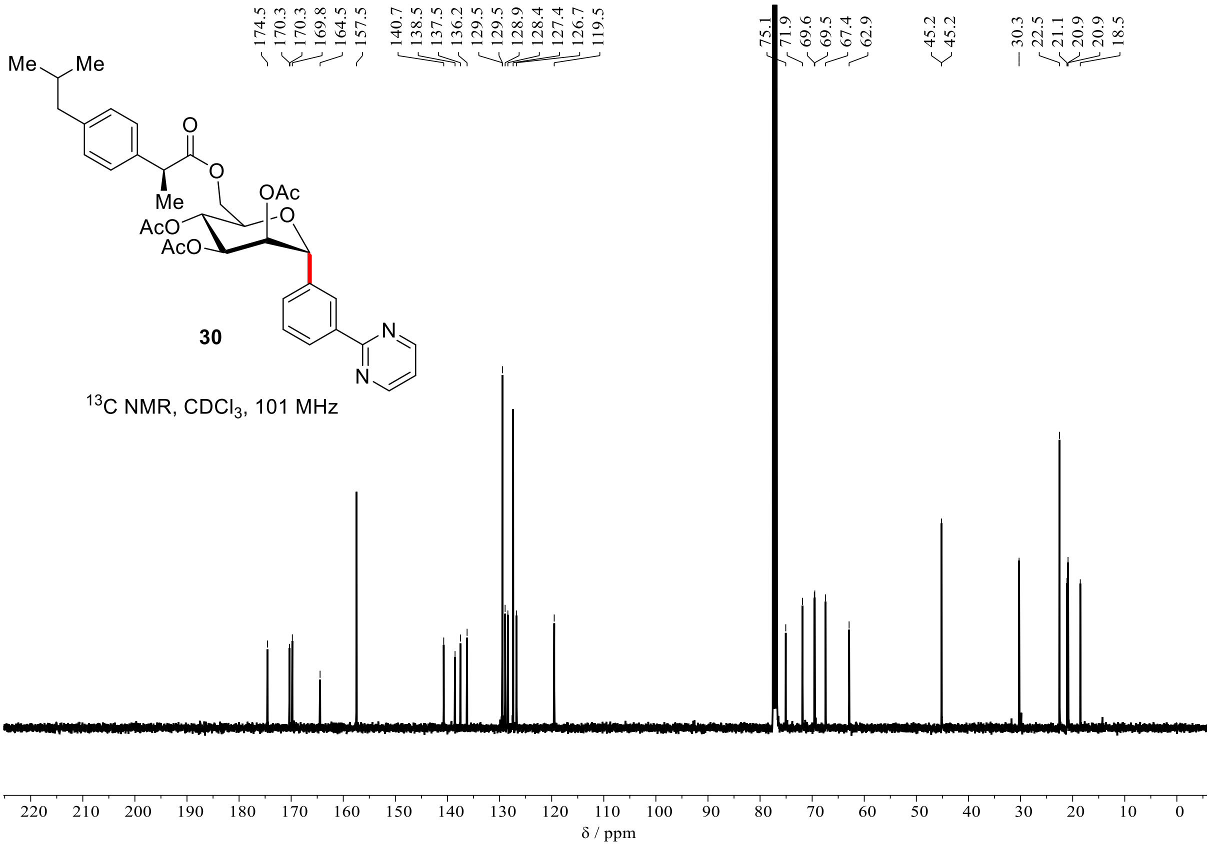
174.5
170.3
170.3
169.8
164.5
157.5
140.7
138.5
137.5
136.2
129.5
129.5
128.9
128.4
127.4
126.7
119.5
75.1
71.9
69.6
69.5
67.4
62.9
45.2
45.2
30.3
22.5
21.1
20.9
20.9
18.5
Me
Me
O
O
Me
OAc
O
AcO
AcO
N
N
30
^{13}C NMR, CDCl$_3$, 101 MHz
220 210 200 190 180 170 160 150 140 130 120 110 100 90 80 70 60 50 40 30 20 10 0
δ / ppm

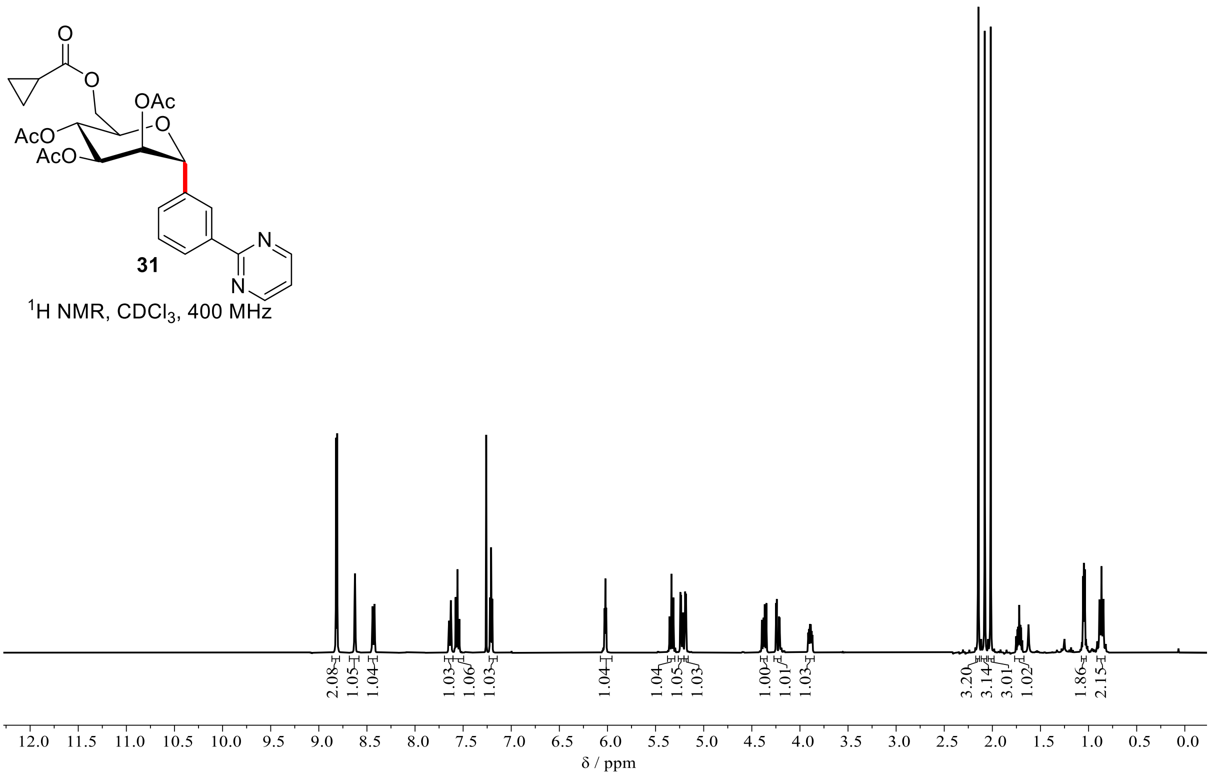
O
O
OAc
O
AcO
AcO
N
N
31
^{1}H NMR, CDCl$_3$, 400 MHz
2.08
1.05
1.04
1.03
1.06
1.03
1.04
1.04
1.05
1.03
1.00
1.01
1.03
3.20
3.14
3.01
1.02
1.86
2.15
12.0 11.5 11.0 10.5 10.0 9.5 9.0 8.5 8.0 7.5 7.0 6.5 6.0 5.5 5.0 4.5 4.0 3.5 3.0 2.5 2.0 1.5 1.0 0.5 0.0
δ / ppm

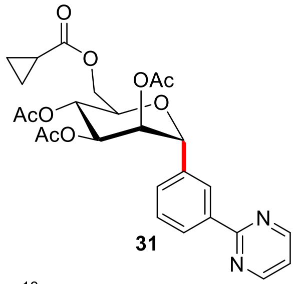

31

^{13}C NMR, $CDCl_3$, 101 MHz

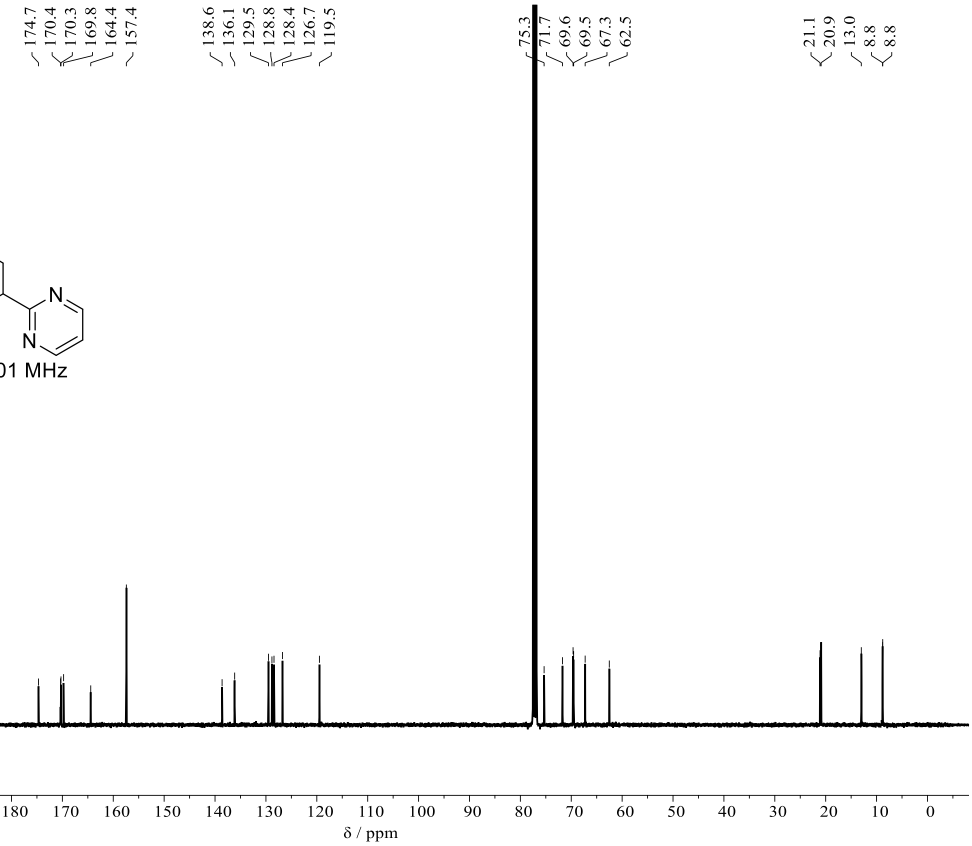

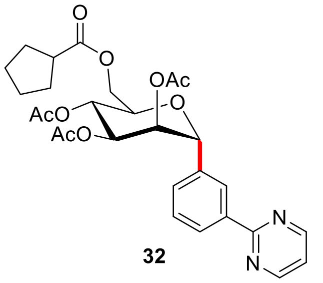

32

^{1}H NMR, $CDCl_3$, 400 MHz

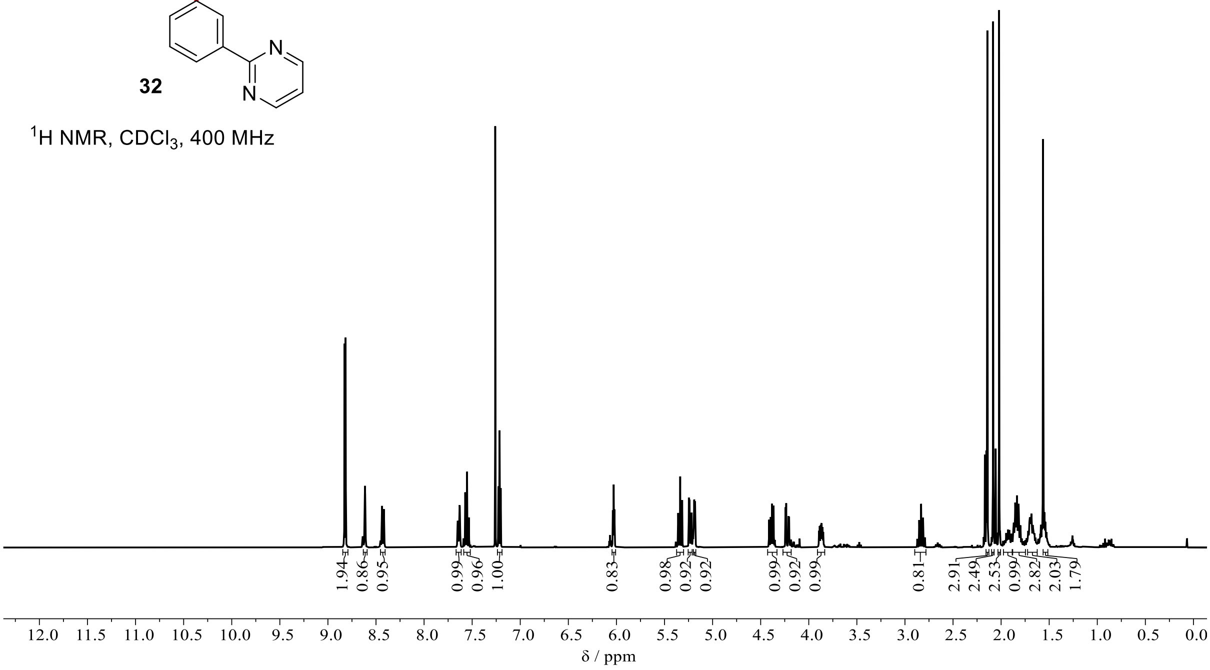

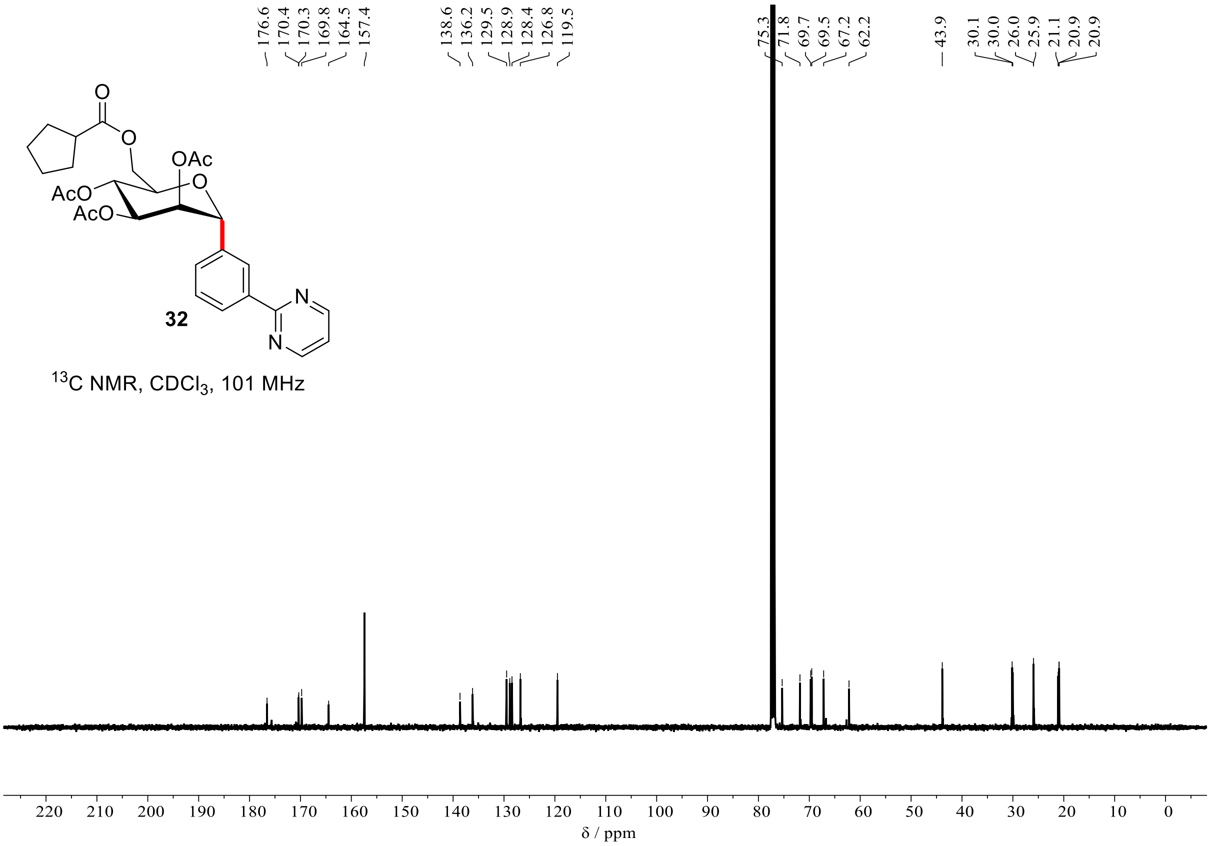
176.6
170.4
170.3
169.8
164.5
157.4
138.6
136.2
129.5
128.9
128.4
126.8
119.5
75.3
71.8
69.7
69.5
67.2
62.2
43.9
30.1
30.0
26.0
25.9
21.1
20.9
20.9
OAc
AcO
AcO
O
N
N
32
13C NMR, CDCl3, 101 MHz
δ / ppm

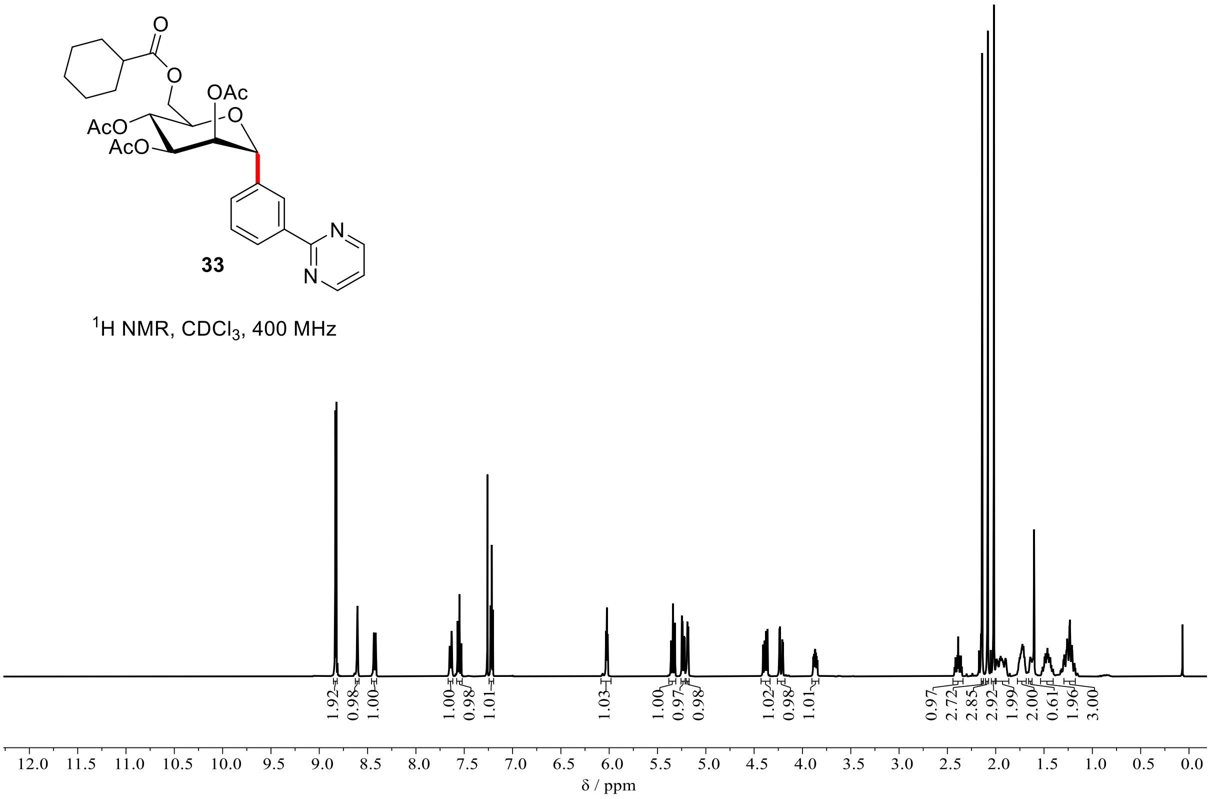
OAc
AcO
AcO
O
N
N
33
1H NMR, CDCl3, 400 MHz
1.92
0.98
1.00
1.00
0.98
1.01
1.03
1.00
0.97
0.98
1.02
0.98
1.01
0.97
2.72
2.85
2.92
1.99
2.00
0.61
1.96
3.00
δ / ppm

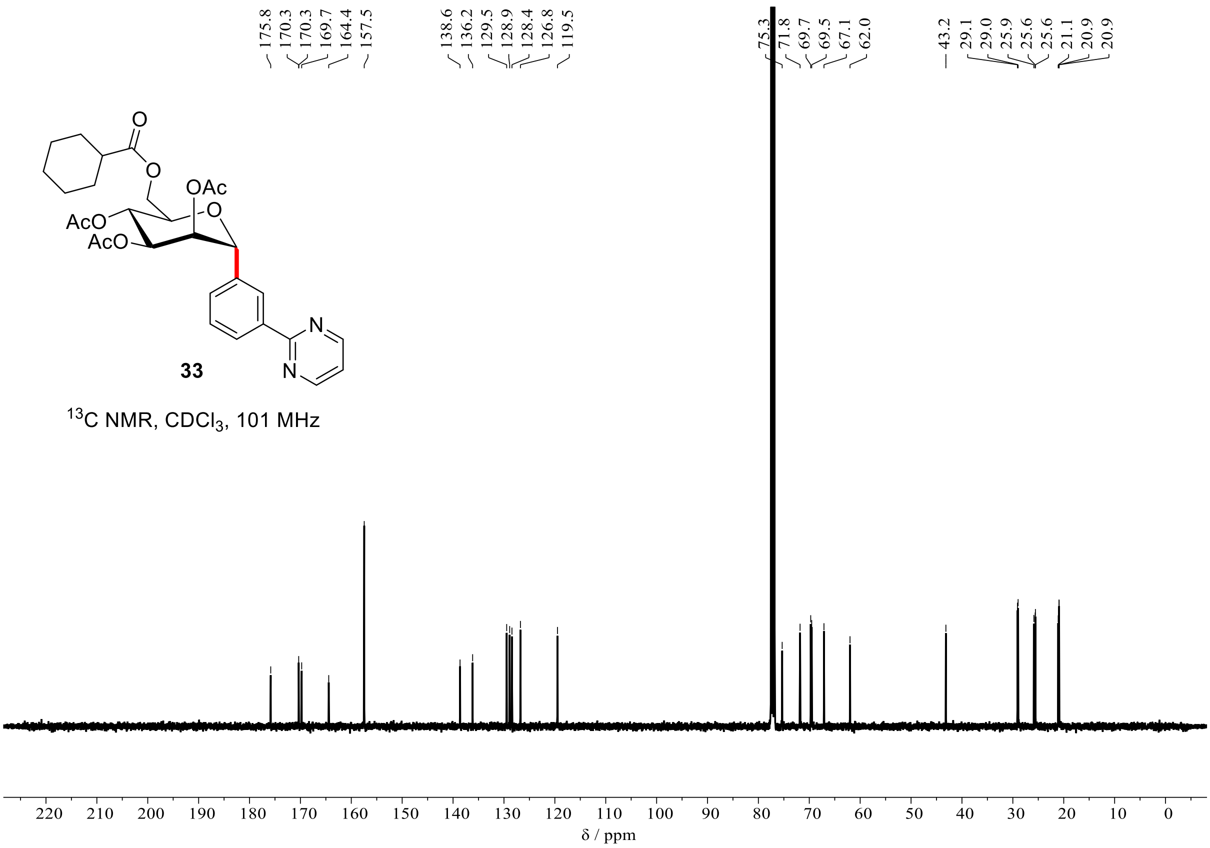
OAc
AcO
AcO
O
N
N
33
^{13}C NMR, CDCl$_3$, 101 MHz
175.8
170.3
170.3
169.7
164.4
157.5
138.6
136.2
129.5
128.9
128.4
126.8
119.5
75.3
71.8
69.7
69.5
67.1
62.0
43.2
29.1
29.0
25.9
25.6
25.6
21.1
20.9
20.9
δ / ppm

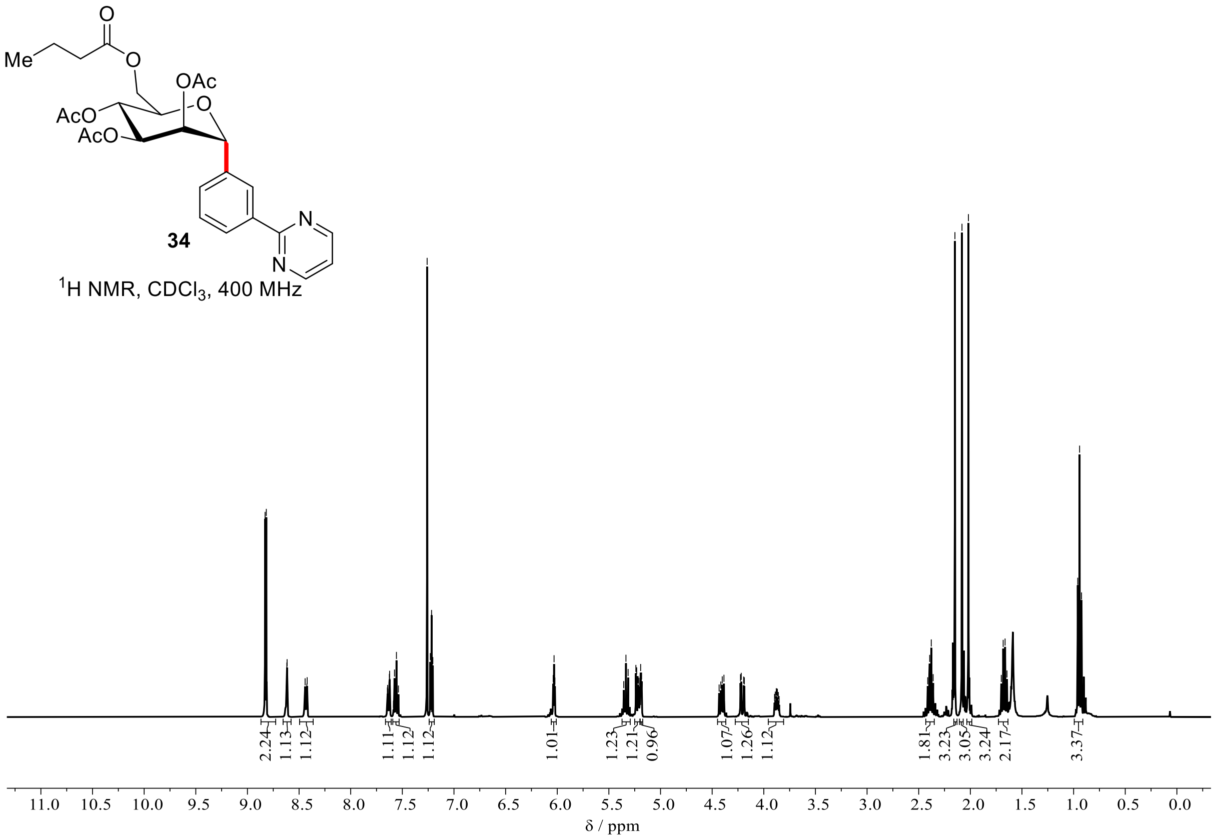
Me
OAc
AcO
AcO
O
N
N
34
^{1}H NMR, CDCl$_3$, 400 MHz
2.24
1.13
1.12
1.11
1.12
1.12
1.01
1.23
1.21
0.96
1.07
1.26
1.12
1.81
3.23
3.05
3.24
2.17
3.37
δ / ppm

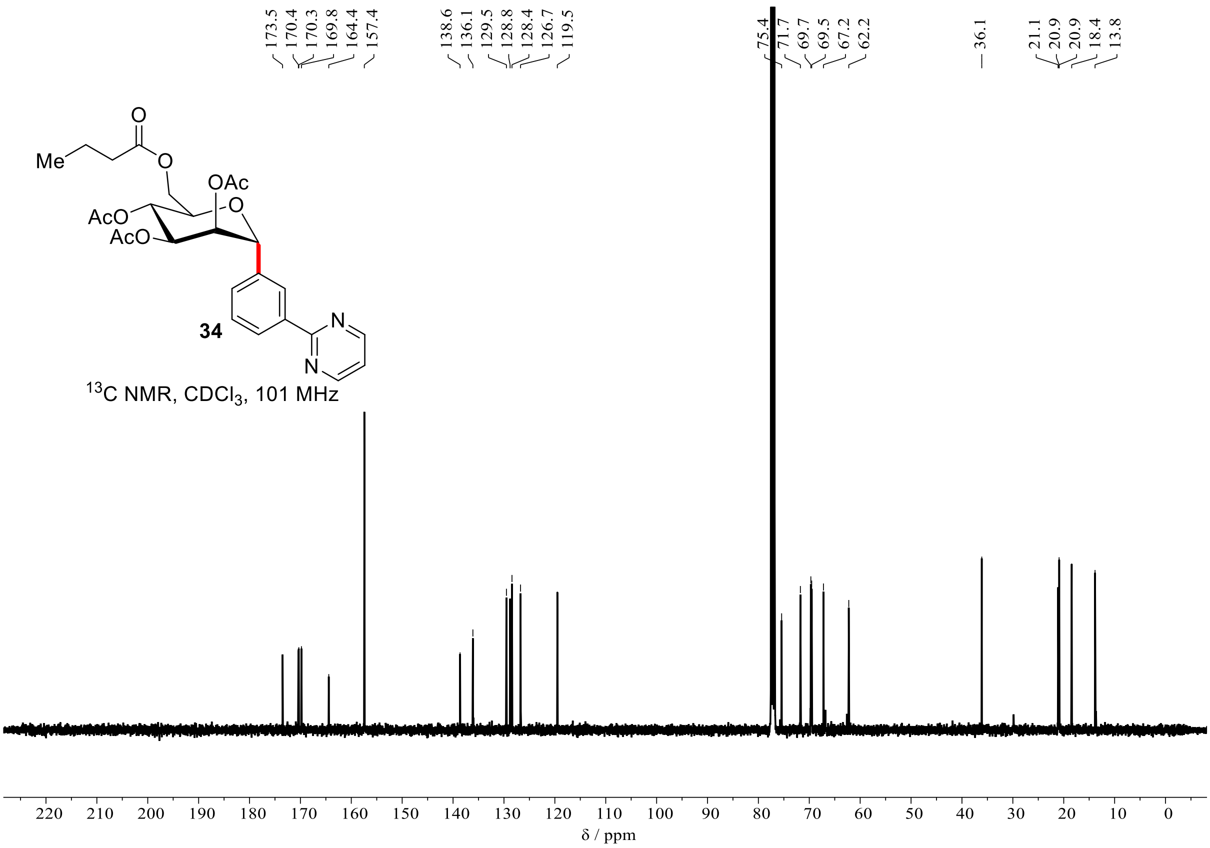
173.5
170.4
170.3
169.8
164.4
157.4
138.6
136.1
129.5
128.8
128.4
126.7
119.5
75.4
71.7
69.7
69.5
67.2
62.2
36.1
21.1
20.9
20.9
18.4
13.8
Me
O
O
OAc
O
AcO
AcO
N
N
34
^{13}C NMR, CDCl$_3$, 101 MHz
δ / ppm

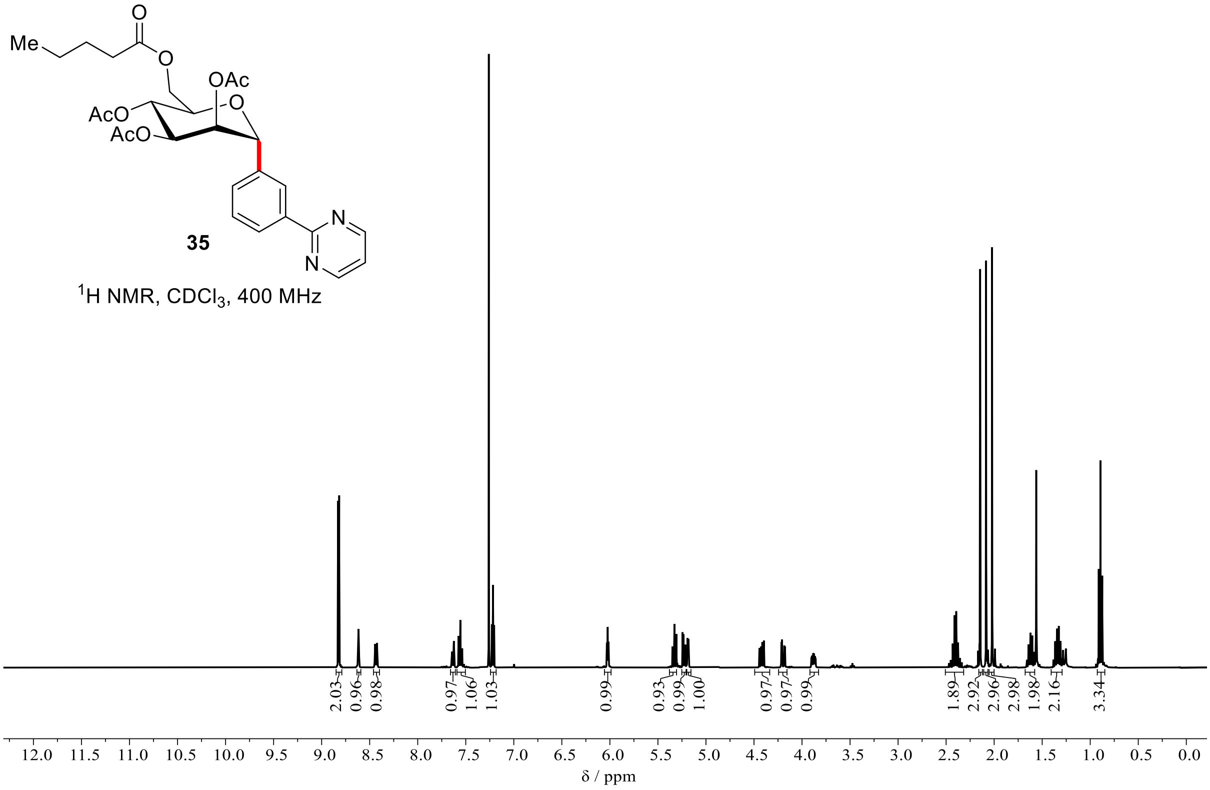
Me
O
O
OAc
O
AcO
AcO
N
N
35
^{1}H NMR, CDCl$_3$, 400 MHz
2.03
0.96
0.98
0.97
1.06
1.03
0.99
0.93
0.99
1.00
0.97
0.97
0.99
1.89
2.92
2.96
2.98
1.98
2.16
3.34
δ / ppm

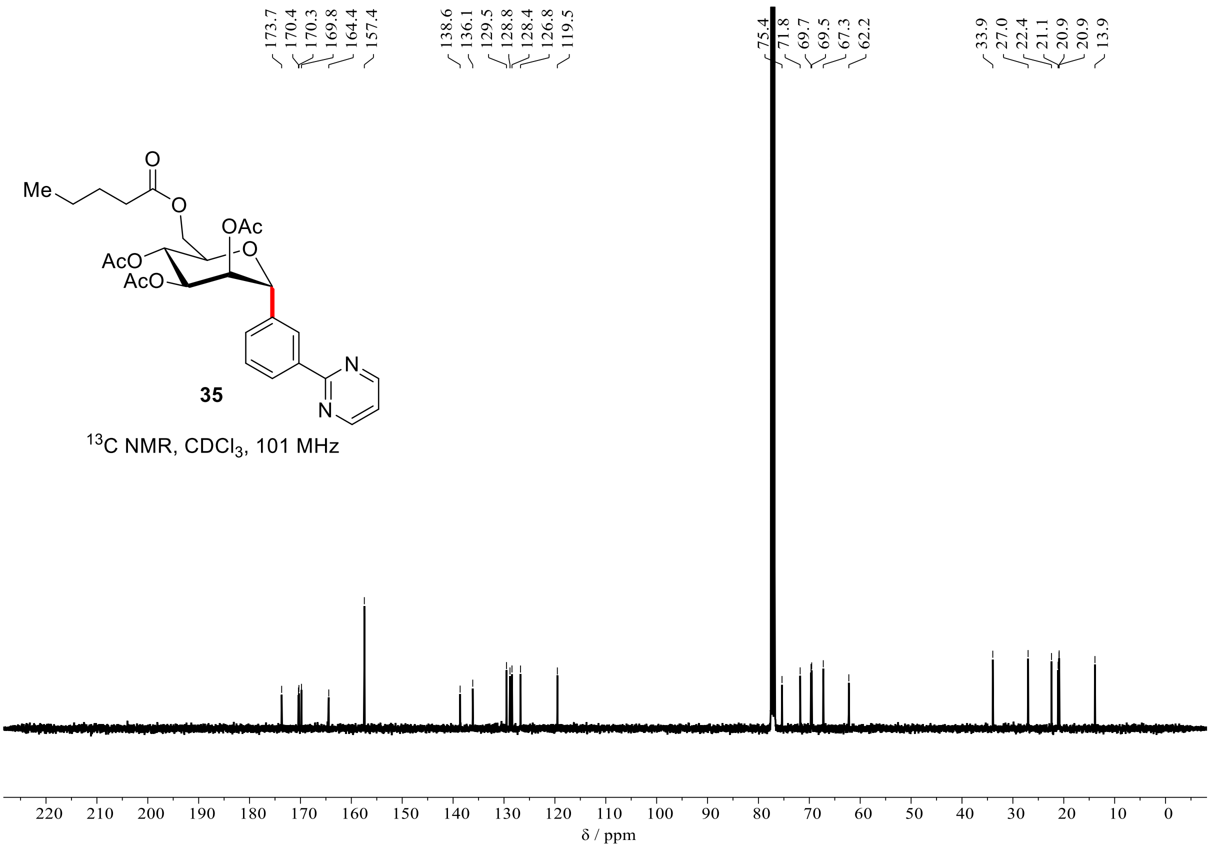
173.7
170.4
170.3
169.8
164.4
157.4
138.6
136.1
129.5
128.8
128.4
126.8
119.5
75.4
71.8
69.7
69.5
67.3
62.2
33.9
27.0
22.4
21.1
20.9
20.9
13.9
Me
O
O
OAc
AcO
O
AcO
N
N
35
^{13}C NMR, CDCl$_3$, 101 MHz
220 210 200 190 180 170 160 150 140 130 120 110 100 90 80 70 60 50 40 30 20 10 0
δ / ppm
